# Targeting structural features of viral genomes with a nano-sized supramolecular drug†‡

**DOI:** 10.1039/d1sc00933h

**Published:** 2021-04-05

**Authors:** Lazaros Melidis, Iain B. Styles, Michael J. Hannon

**Affiliations:** Physical Sciences for Health Centre, University of Birmingham Edgbaston Birmingham B15 2TT UK; School of Computer Science, University of Birmingham Edgbaston Birmingham B15 2TT UK; Centre of Membrane Proteins and Receptors, The Universities of Birmingham and Nottingham The Midlands UK; Alan Turing Institute London UK; School of Chemistry, University of Birmingham Edgbaston Birmingham B15 2TT UK m.j.hannon@bham.ac.uk

## Abstract

RNA targeting is an exciting frontier for drug design. Intriguing targets include functional RNA structures in structurally-conserved untranslated regions (UTRs) of many lethal viruses. However, computational docking screens, valuable in protein structure targeting, fail for inherently flexible RNA. Herein we harness MD simulations with Markov state modeling to enable nanosize metallo-supramolecular cylinders to explore the dynamic RNA conformational landscape of HIV-1 TAR untranslated region RNA (representative for many viruses) replicating experimental observations. These cylinders are exciting as they have unprecedented nucleic acid binding and are the first supramolecular helicates shown to have anti-viral activity *in cellulo*: the approach developed in this study provides additional new insight about how such viral UTR structures might be targeted with the cylinder binding into the heart of an RNA-bulge cavity, how that reduces the conformational flexibility of the RNA and molecular details of the insertion mechanism. The approach and understanding developed represents a new roadmap for design of supramolecular drugs to target RNA structural motifs across biology and nucleic acid nanoscience.

## Abstract 2

MD simulations and Markov state modeling explore induced fit binding of metallo-helicates to bulges in dynamic TAR RNA, reproduce experimental data, show how RNA conformational flexibility is reduced, and give mechanistic insight into insertion.

## Graphical abstract

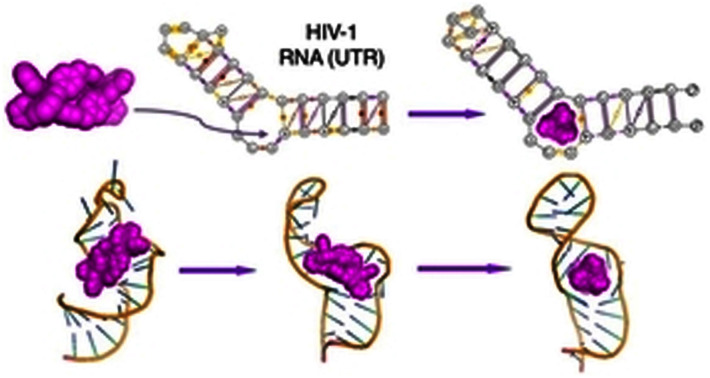

## Introduction

Infectious disease represents one of the greatest current threats to humans as demonstrated by the frequency of recent lethal viral outbreaks: 4 out of the 10 greatest threats identified by the World Health Organization are viral related. While vaccines offer long-term eradication or suppression, they are bespoke to the disease and their development and implementation across a global population is slow. There is therefore a pressing need for a new generation of drugs that could hold an emerging disease at bay while bespoke solutions are created; broad-acting anti-viral agents having different molecular designs and molecular targets, offering a diverse platform that maximizes the potential preventative effect against new diseases.

Modern drug research tends to focus primarily on the protein targets as the effectors of disease. However, to target broad classes of disease, drugs that target the nucleic acids^1–5^ (DNA, RNA) of the infectious agents are of particular interest with RNA increasingly recognized as a druggable target.^6,7^ The rapid emergence of infections, and subsequent rapid evolution of viral genetic sequences, means that drugs that target a specific sequence are unsuitable. However, agents that target a specific nucleic-acid structure could be much more interesting. In particular, the untranslated regions (UTR) at both 3′ and 5′ ends of many viral genomes are not only highly structured but often share common structural elements^7–10^ that are functionally essential and so conserved as the virus evolves (drifts) genetically.^10,11^ Indeed, structure-affecting mutations in the UTR have been used to create live attenuated or inactivated vaccine strains.^12,13^ UTRs have been mostly studied in RNA viruses, such as HIVs,^7,10,14,15^ coronaviruses,^16–18^ dengue,^11,12,19,20^ zika^21^ and other flaviviruses^22^ and, in every studied case, functional involvement of the UTR has been shown in either initiation of replication^16,19^ (by recruiting proteins or by direct interaction with the ribosome) or regulation of the replication cycle. The most studied example is the retrovirus HIV-1 which contains a bulge in the first stem loop of the 5′ UTR of its RNA genome,^23–30^ the structure and dynamics of which are crucial for initiation of viral replication. Similar bulges are found in UTRs of other RNA viruses including coronaviruses and SARS-COV-2. These UTR structures represent exciting potential anti-viral targets.

Structure-based recognition of RNA (and DNA) by drugs is still very much in its infancy.^4,31–34^ The molecular structural information needed for such recognition is not yet available for most viruses, and crystal structures of drugs bound to RNA structures are rare (and not necessarily representative); new molecular-level understanding of such binding is a critical need. Structural studies on RNA are further complicated by the inherent flexibility of RNA molecules, which requires an understanding of their dynamics not just their ground state conformation. Consequently, simple molecular docking will not suffice; by contrast molecular dynamics potentially allows the energy landscape and structural flexibility to be probed. Herein we employ molecular dynamics to explore in detail, for the first time, a nano-scale drug inserting into a bulge in a UTR viral RNA, replicating experimental observations and gaining fundamental new insight into the dynamics of the RNA and of the drug entry process; crucial intelligence to inform design of new UTR-structure-targeting drugs. The nano-scale drugs studied are supramolecular cylinders, which not only have unprecedented RNA bulge-binding ability but are the first in class of metallo-supramolecular architectures to show potent anti-viral activity in cellular assays.^35^ There is a growing interest in the application of metallo-supramolecular architectures in biology.^36–41^

## Results and discussion

**Fig. 1:**
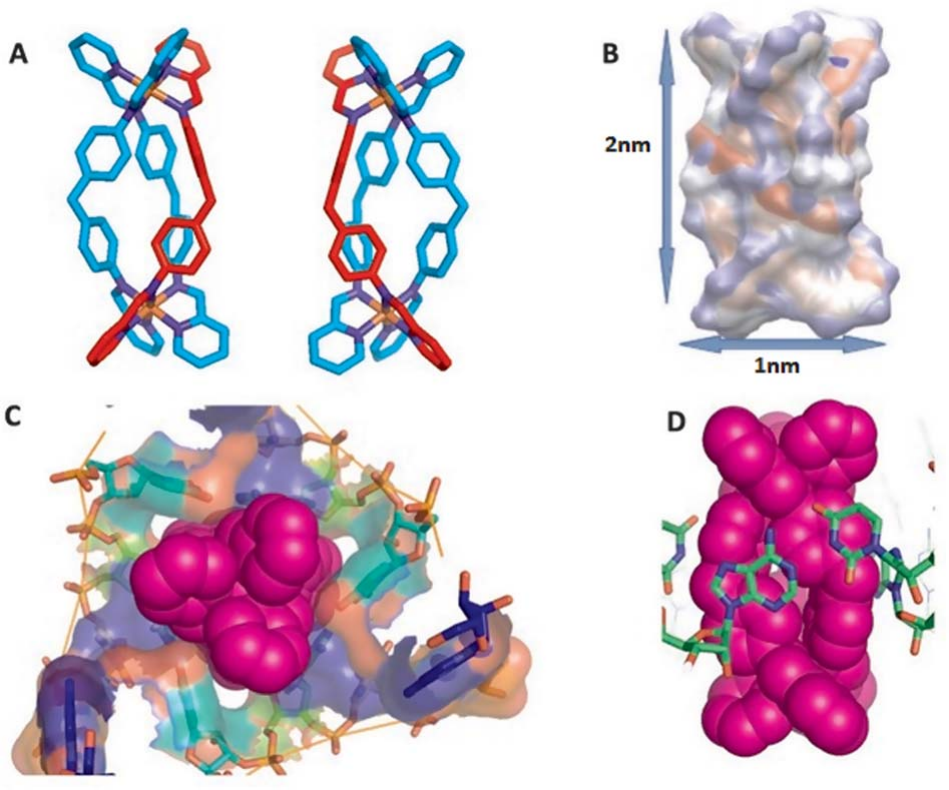
(A) *P* and *M* enantiomers of the iron cylinder [Fe_2_L_3_]^4+^ optimized by DFT. (B) Distribution of partial charges for *P* enantiomer as calculated by ssb-d-D3/Def2-SVP level of DFT theory, visualized by VMD, and also showing approximate cylinder size. (C) Surface of the RNA 3-way junction cavity stabilized by the *M* enantiomer of the cylinder from the crystal structure pdb 4JIY.^43^ (D) Stacking of RNA bases to the cylinder in the centre of the 3-way junction in pdb 4JIY.^43^ Analogous stacking is also seen with cylinders located at the terminal base pairs of the strands (see ESI part B‡). Hydrogen are omitted for clarity in A, C and D.

As a suitable UTR structure for our studies we chose HIV-1 TAR RNA which is both experimentally well described and representative of wider viral UTR structural motifs. As a drug we chose a nanoscale metallo-supramolecular cylinder because it is unique as a nano-drug that has previously been crystallographically characterised when bound within an RNA cavity (a perfect three-way junction (3WJ)) (Fig. 1).^42,43^ It is also unique in threading through an RNA cavity, interacting with all of the internal structure. These cylinders also bind bulge structures in RNA, prevent TAT protein from recognizing the binding site in the TAR sequence of HIV^35,44^ and arrest HIV replication in mammalian cells.^35^ The strong evidence of binding and in-cell efficacy, makes this an ideal test-bed to investigate whether molecular dynamics simulations can identify the processes that underpin the kinetics of targeting highly flexible RNA strands. At the same time, it provides a suitable challenging size of drug, and one with large, nanoscale, 3-dimensional molecular surfaces whose match and strong binding to the 3D shape of RNA structural motifs should collapse the RNA's conformational landscape to a non-functional (impotent) state. The cylinder exists in two enantiomeric forms, both of which bind RNA bulges. Experimental X-ray crystal structures are also available for unbound cylinders;^45,46^ the calculated DFT structures herein are almost identical.

### Simulations of RNAs (uncomplexed)

For multi-microsecond simulations, classical MD forcefields describing the dynamics of both RNA and DNA have until very recently^47–52^ been found to be unsatisfactory – over such timescales they induced structures not seen experimentally. With longer simulations being available, the conformational space sampled can deviate further from the absolute minimum energy point and explore the importance of non-covalent interaction dynamics as pi-stacking and hydrogen bonding.^53–57^ However new forcefields^50–52^ have become available and we show now that the Rochester-Mathews forcefield^51,53^ can be used to simulate RNA over long timescales, reproducibly, not only for free RNA but for drug–bound complexes. The Rochester-Mathews forcefield is publicly available giving it the potential to be accessed and implemented by all. It uses the same underpinning level of DFT theory as that applied to metal-containing cylinder coordination compounds creating an overall consistency. Moreover, there are ways to accurately model NMR ensembles of RNA structures without the need of extensive MD simulations.^58^ Collectively we accumulated over 200 μs of simulated time; such long and data-rich simulations on a flexible RNA system, brought new challenges in analysis. We address these by applying Markov state modeling^59,60^ to the problem and show that this enables us to identify stable and metastable conformations among the millions of frames.

Overall we have performed 123 simulations of at least 1 μs and up to 10 μs, overall ∼200 μs including several shorter runs with varying initial conditions. To analyse the vast volume of data, over 200 000 000 coordinate frames, we employ the PyEmma workflow^60^ and Markov State Modelling (MSM). This involves reducing the dimensionality by choosing appropriate features of the simulation and identifying macrostates of each simulations using MSM and extracting those metastable structures with Perron-cluster cluster analysis (PCCA). Those extracted structures and the whole simulation are also presented in the Leontis–Westholf^61^ nomenclature using Barnaba.^62^ A detailed explanation of this workflow is included in ESI.‡

To confirm the ability of the forcefield^50–52^ to conserve structural features of viral stem-loop RNAs (as observed, dynamically, in NMR), and to establish the effectiveness of our approach to analysis, we first explored the dynamics of poliovirus stem loop (pdb: 2GRW)^63^ coxsackievirus stem loop (pdb: 1RFR)^64^ and HIV2-TAR (pdb: 1AJU)^65^ RNA with no bound drugs. The simulations reliably reproduced NMR observations for the stem loops (including regions of non-Watson–Crick pairing) and the predicted effect of a small bound ligand on the HIV2 TAR. Indeed for poliovirus stem-loop, the MD simulations reveal and explain features that are observed in the NMR structural data, but have not previously been satisfactorily captured in the deposited conformations, and for HIV2-TAR shows how the ligand-free RNA structure deviates from the conformation of the bound state, demonstrating the effect a binding molecule can have on an RNA structure: a detailed analysis of these free RNA simulations is included in ESI.‡

#### HIV1-TAR

**Fig. 2:**
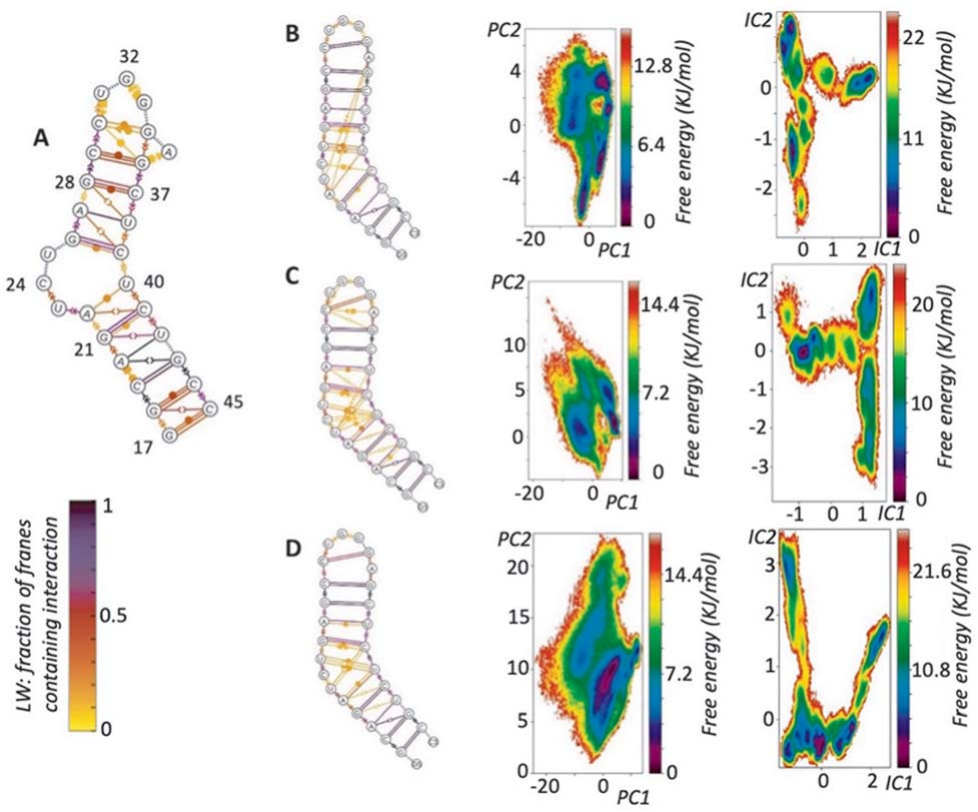
(A) Summary of the 1ANR 20 NMR solutions presented in Leontis Westhof (LW) nomenclature. (B) LW nomenclature of 10 μs simulation and PCA and TICA free energy surfaces, demonstrating: how the simulation reproduces 1ANR NMR structure but also reveals transient pairings (LW yellow) not well defined by (but nevertheless noted in) NMR; the greater richness of information in TICA analysis over PCA; the many conformations (TICA minima) that are accessible in the simulation at this temperature (310 K). (C) LW nomenclature of 6 μs simulation and PCA and TICA free energy surfaces. (D) Combined results of seven 2 μs simulations (see ESI Methods‡) starting with different NMR solutions.

We now turned to a more in depth study of the dynamics of our test UTR stem-loop, the HIV-1 TAR RNA. While in the coxsackievirus, poliovirus and HIV-2 simulations we had focused on the proposed ground state of the RNA as the starting point for the simulations, now we expanded our attention beyond the ground state to look also at other conformations within the experimentally suggested (NMR; pdb 1ANR) structures. In an effort to avoid introducing biases and acceleration methods to the simulation we chose to explore the conformation landscape by starting simulations from different local minima as described in the original HIV-1 TAR NMR solution structure.^66^ There are 20 NMR solutions proposed and we started from five such minima (first, third, fourth, seventh and twelfth). For each of these higher energy solutions a 2 μs simulation retained the characteristics consistent with the NMR structure and did not deviate into unnatural (loosely bound) conformations. From each starting point similar features can be observed as the simulation proceeds which indicates that the forcefield can reproduce transitions within the landscape of a few μs per solution. These unbiased MD simulations capture the conformational changes of the RNA across the energy landscape for the first time, and clearly reveal the variation possible in the RNA structure and the range of conformations sampled (and which a drug could encounter and sample). Importantly, time-lagged independent component analysis (TICA) of the trajectories (Fig. 2) revealed a broad energy minimum in the ground state which shows that small perturbations in the conformation have minimal effect on the energy. Moreover, a single 10 μs long simulation (as well as an independent 6 μs long simulation) of the ground state reveals the conformational richness near to the minimum. These observations highlight the limitation of a simple docking approach for flexible RNAs.

Across the simulations, the helical regions remain relatively stable with strong WC base pairing. The only stem base pair not retaining the WC pairing is A22:U40, which often drifts apart as the U40 retains strong stacking with C39. It is often the case that U40 seems to be in the 2^nd^ rather than the first stem.

While a variety of transient base-pairings of all types were observed in the bulge region, as expected from the experimental NMR observations, no new stable base pairings were observed apart from that between C30 and G33/4 which is not observed by NMR but is observed in gel electrophoresis. On the un-bulged strand stacking is strong and continuous, but this is a lot less evident on the bulge strand. Of the three bulged nucleotides, U23 and C24 are more likely to stack whereas U25 is the most likely to be fully outside the helix and can even create long range interactions with the loop nucleotides (G33 and A35) creating a transient folding up of the second stem. Such a folding was not observed in the HIV-2 TAR simulation.

The loop region is characterised by limited stacking between bases and common WC pairing between C30 and G34. Transient non-WC pairing can include C30 *cis* or *trans* WC/Hoogsteen to A35.

Examining the runs starting from the different local energy minima; the first simulation starting from 1anr1 identified 3 distinct states, that can be recognised even by the PCA analysis. All are energetically and conformationally close together as seen by the RMSD and ERMSD. PCCA analysis shows one to be in much higher occupancy, clearly the ground state. A second simulation also starting from 1anr1 sampled a wider conformational space. Base pairing of stems was retained although stacking between C19:G43 and A20:U21 was not, although it is observed in the NMR. After that, stacking does continue all the way to the loop. At the loop a few different conformations were sampled that mostly gave rise to the different MSM states identified. C30 base pairs with either G33 or G34. In the bulge region U40 is stacked strongly with C39 but not always to C41 and transient, short lived pairing takes place between all bulge residues and either of C39 and U40, with pairing types including both sugar and Hoogsteen edges as well as in the trans position. MSM analysis gave 6 different states.

The simulation starting from the third NMR solution, 1anr3, yielded 5 local minima in the TiCA projections and CK test allowed for 5 states in MSM analysis. Overall, stacking and pairing throughout the stems is conserved and transient pairing within the bulge region is similar to that of the previous run. Most importantly the second state is very reminiscent of the ground state.

As we planned to apply significant external forces to the RNA structure, by introducing the cylinder into the system, we also tested the behaviour of the forcefield with higher NMR energy solutions. An experimental analysis of higher energy RNA conformations (when in the presence of a bound ligand) has been discussed by Orlovsky *et al.*^67^ In that work, 3 nucleotide bulges are observed to adopt multiple conformations; we replicate these multiple conformations in our simulations (Fig. 2B–D, ESI‡) providing further experimental validation of our model. Going up the energy ladder from the starting conformation one might expect to encounter more structures that deviate significantly from the ground state. Nevertheless starting from the fourth solution, 1anr4, most of the important structural features were retained. Pairing and stacking remains consistent with the exception of the U23 to C24 stacking. PCA revealed 3 stationary points which become 5 with TiCA. Also notable is that from this state up, examining the first 4 TiCA vectors instead of just two showed much higher diversity. In the loop, pairing C30:G34 is seen again, as well as the usual transient non-traditional pairing, but now interactions between U23 and U38 and trans Hoogsteen to sugar between U23 and C39 are observed. Stacking of U40 to C39 remains strong but stacking of U23 to C24 was less prevalent.

The seventh, 1anr7, and twelfth, 1anr12, structures are quite different from the ground state and this brings challenges for the simulation: specifically, the loss of A helix structures which is characterised by the overall elongation of G17 to G33 distance can be testing to any forcefield. Nevertheless, starting from 1anr7, the stacking and pairing remains consistent. PCA identified 2 states whereas TiCA suggested 6 states and the CK test is also passed with 6 states. The first 4 states are reminiscent of the ground state with different loop configurations, namely sugar to Hoogsteen between C30 and A35, or less often trans WC to Hoogsteen. In the other two states, U25, which generally points outside the bulge can create temporary long-range interactions with loop residue G33.

Starting from 1anr12, which is also very elongated with a sharp backbone kink in the bulge area, also retrieved most of the properties of the ground state. Pairing and stacking remain consistent for the stems. In the loop the common C30 to G34 pairing is stable along with a transient Hoogsteen to sugar between A35 and C30. In the bulge region stacking between C39 and U40 is strong and most of the transient non traditional base pairings are also seen. PCA revealed 2 states whereas TiCA revealed 5.

The results demonstrate that the forcefield can satisfactorily retain characteristics of the structure as described by the NMR experimental constrains.

In addition to the unbound 1anr structure, there are some TAR RNA structures with various different bound drugs, and so for comparison we also explored as a starting point one such structure (the only solution of pdb; 1UUI)^68^ from which we had removed the drug. The structure, after removing the ligand, has some differences with the 1anr structure: pairing on the stems is the same, but stacking is disturbed before the bulge, probably since U23 is WC paired with A27.

When using this as the starting point for a 2 μs simulation, the loop folded back onto the bulge (from which the ligand had been removed) forming interactions from U23 and C24 to A35, and the stem remained folded for much of the simulation. The bulge stacking did not return to the transient pairings seen in the earlier simulations. PCA analysis of the simulation revealed 3 states and TiCA 6, which was also passed the CK test on with MSM with the sixth state being ground state of this run. The simulation demonstrates how ligand binding can modify the structure and dynamics of the TAR RNA and again highlights that docking, while a useful guide, may miss key features and opportunities. The Rochester forcefield^52^ behaved well for every case of RNA molecular dynamics, even in cases outside the ground state of the structure in question.

### Cylinders binding to HIV1-TAR

#### Docking studies

Disney has recently used docking to screen libraries of small molecules binding to RNAs including TAR.^3,52^ We initially undertook simple docking calculations as described in methods using all 20 structures from pdb; 1anr TAR RNA NMR study. The results are dominated by different forms of bulge region binding. While the two enantiomers do show slightly different binding energies, the Autodock Vina^69^ as other docking software (used as it is one of few that allow incorporation of first row d-block metal centres) as other docking software tends to underestimate the electrostatic contribution when a charged molecule is involved. Nevertheless the docking scores are high compared to other small molecule drugs assessed by this method reflecting the larger available surface of the cylinder.

It is interesting to compare the results of docking with overall results of subsequent MD simulations. In particular in the MD simulations, capping of the open terminal bases is a transient, but relatively stable (more than 2 μs) location seen with both enantiomers. Although only a local minimum in the interaction of cylinders with TAR it highlights the limitations of docking in targeting nucleic acids because, across all 20 NMR solutions of TAR RNA, the terminal bases are coplanar only in one (the ninth). Consequently only in this structure solution does the docking reveal the end capping as a potential binding site. So docking outcomes are constrained by the rigid RNA structure(s) used in the docking, whereas in reality – as we shall see – RNAs are highly fluxional and dynamic molecules that access much structural space. Thus while such simple docking studies are valuable for high throughput screening they might be more suited to small molecules where the molecule is less likely to have a major effect on RNA conformation. For the larger cylinders the size of the binding surface means that induced conformational change is more likely and so more sophisticated MD can offer greater insight into the interaction. Crucially, while the docking showed bulge region binding, bulge insertion by the cylinder was not observed.

#### Molecular dynamics simulations

To examine the interaction between TAR and the cylinders, simulations (112) started with the cylinder (DFT optimized – Fig. 1A and B) in random places 1 nm away from the RNA as well as from sites identified by docking studies with initial TAR conformations derived from multiple experimental 1ANR solutions examined earlier.

The size of the cylinder restricts how rapidly it will move between sites (local minima) in the simulations' timescale. Consequently a single simulation would fail to explore all binding sites and conformations. Instead we take the quite different approach of using multiple simulations (1–10 μs) from different starting points which allows the cylinder to explore a much greater range of RNA conformations and to encounter multiple potential binding sites. By combining this with Markov state modelling analysis we are now able to explore effectively the dynamic conformational landscape of the TAR RNA – cylinder complex.

The simulations show the cylinder moving up, down and around the DNA exploring different sites and positions, and moving between them, until it ultimately inserts into the 3-base bulge. Such a dynamic exploration of different positions is what is anticipated for such a polycation with a sophisticated RNA polyanion in these timescales. There are a number of different, kinetically-accessible, positions that the cylinder explores and occupies transiently *en route*, of which some represent local minima with longer residence times (though still transient) and are identified from the MSM analysis (Fig. S8 and S9‡). We and we will describe these briefly before turning to the 3-base bulge that is the ultimate binding site.

#### Transient end-stacking interactions

**Fig. 3:**
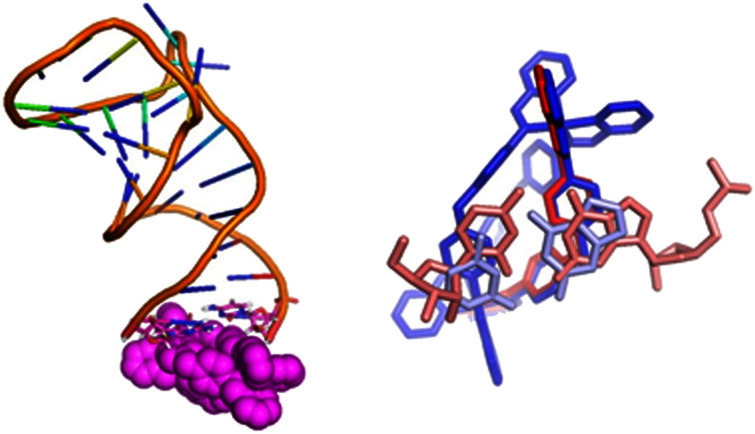
Left: end-capping of the cylinder observed in an MD simulation. Right: the end-stacking experimentally observed in crystal structure 4JIY^43^ (red), overlain with that observed in an MD simulation followed by DFT optimisation (blue).

Often the cylinder (both enantiomers) found a local minimum, which it occupied for at least 1 μs at a time, and in which it capped the terminal G17:C45 bases (Fig. 3). Some RNA forcefields have been suggested to over-emphasise base-stacking.^70,71^ However, in this RNA system this binding position is among the most accessible kinetically and, since such cylinder binding has also been observed in X-ray crystal structures,^42,43^ it demonstrates that the simulation is replicating an experimentally validated binding location. To assess how well the forcefield and the parameterisation (now including the cylinder) reproduces this binding as captured by the crystal structure we extracted the G17:C45 bases and the cylinder from a frame of the longest lived position and we then optimised that structure at the ssb-d-D3/LANL2DZ (DFT and semi-empirical (PM7)) and superimposed it on the binding mode extracted from a crystal structure. The overlap (Fig. 3) is extremely good, implying that the forcefield is working as desired, and that the crystallographically observed binding is reproduced. This end capping is to some extent a feature of using a shortened oligonucleotide both in these simulations and in X-ray crystal structures: it certainly does demonstrate the affinity of the cylinder for extended planar pi-surfaces, but such end capping sites are not so common in biologically active RNAs.

#### Transient groove interactions

The cylinder is commonly observed exploring the RNA grooves, primarily the groove of the first stem. The residence time for the *M* enantiomer on average is longer than for the *P* implying that the *M* enantiomer may have a higher affinity for the grooves although the kinetics were not adequately sampled to quantify difference.

#### Transient loop interactions

The cylinder can take advantage of unpaired open bases of the loop and interact transiently there (also seen in simulations with the coxsackievirus stem), but this is less commonly observed in the simulation compared to other locations. Loops are a common feature in RNA structures (and indeed in non-canonical DNA structures such as G-quadruplexes and i-motifs) but seem not to be a particular target for the cylinder, consistent with our experimental observations.

#### Transient interactions in the bulge area

**Fig. 4:**
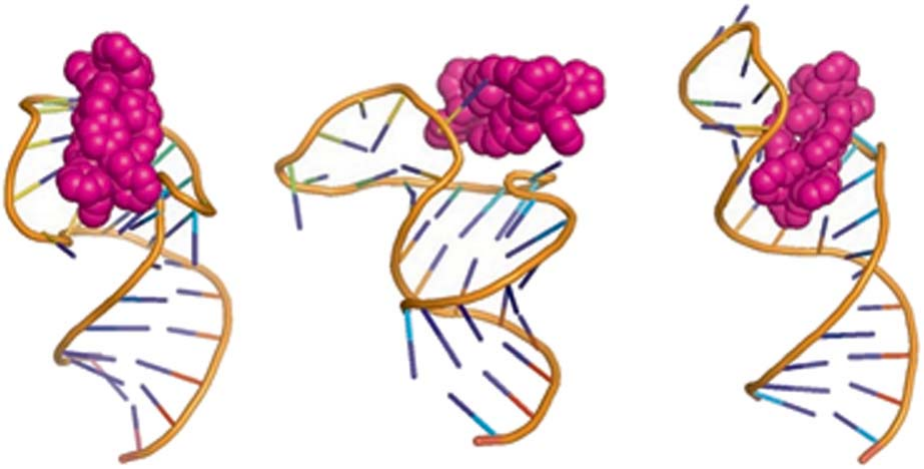
Exemplar bulge-binding interactions observed in the simulations, *en route* to bulge insertion, including bridging from bulge to loop. The right hand figure has the cylinder in the position where a cyclic peptide has been observed to bind to TAR.

The cylinder is most frequently found around or on the bulge (Fig. 4) in the simulation (and as confirmed by experimental data^35,44^), with *M* and *P* being very similar in their preference for this location. RNA conformations that involve the loop bridging to the bulge (U25) can be stabilised for longer (compared to free TAR), with the cylinder sitting on top of the bridge or mediating stacking. In the absence of the bridge, the cylinder can also sit between the bulge and the opposite RNA strand, in a position in which it opens up the base pairing protecting the TAT binding site. In the case that the cylinder sits on the bulge nucleotides, it stabilises the transient base pairing and dislocates the counter ions that would normally reside there which leads to an overall elongated structure of the RNA with minimal helicity.

In this context it is noteworthy that Keene and Collins have explored the binding of a dinuclear ruthenium polypyridyl agent (but of quite different shape to the cylinders) to a TAR-like RNA and proposed that it might bind around the groove near the bulge.^72,73^ Given that the bulge-area is the most frequent location for the cylinder prior to bulge-insertion, it seems likely that this region could also be a preferred area of binding for other dinuclear complexes that cannot insert inside the bulge; for example differently shaped metallo-helices have been reported to not remain bound to TAR in electrophoresis,^74^ in contrast to the bulge-inserting cylinders herein,^35,44^ and might be more loosely associated outside the bulge.

#### Bulge insertion

**Fig. 5:**
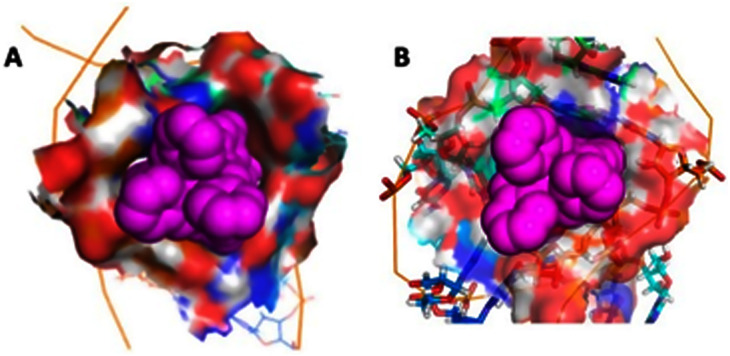
The bulge insertion mode: the surface of the RNA cavity shows the extremely high contact surface for (A) the *M* enantiomer and (B) the *P* enantiomer, and the similarity to each other and to the 3WJ-binding (compare Fig. 1C).

For both *M* and *P* enantiomers, insertion into the bulge is observed; once in the bulge the cylinder is strongly bound and remains there. In this unique binding mode, the cylinder sits in a V-shaped cleft (Fig. 5) that resembles the 3WJ structure (Fig. 1C). The effect of the binding is to restrict/collapse the conformational flexibility of the RNA, prevent the transient loop–bulge interactions and lessen the helicity of the stems. It is striking that, although this is the most stable binding mode in simulations, it fails to be identified in docking studies from any of the 20 1ANR solutions, because docking does not account for RNA flexibility. The bulge insertion and its effects are consistent with and explain both experimental RNase A footprinting results^44^ and the ability of this cylinder to remain bound in electrophoresis when other metallo-helices do not.^74^

The MD simulations also provide intriguing molecular-level insight into how an insertion is possible:

#### Entry mechanism for *M* enantiomer (Movies S1, S2; ‡Fig. 6A–E)

**Fig. 6:**
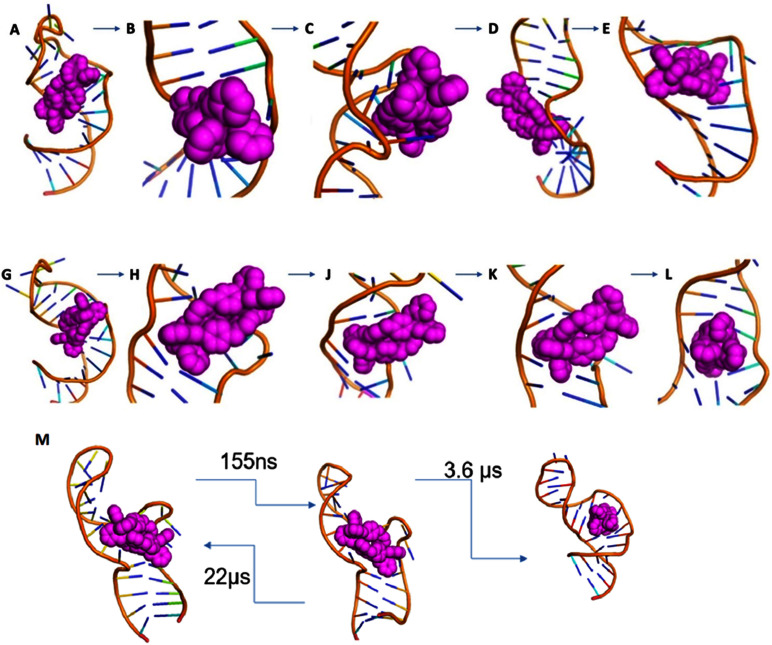
(A–E) Entry of *M* enantiomer: (A) starting position of *M* cylinder on 1ANR1. (B) Cylinder rotates to split the U25 G26 and (C) aligns in parallel to the G26:C39 base pair (order of microseconds). (D) After relaxation of the backbone (order of microseconds), (E) the cylinder is inserted into the cavity (order of nanoseconds). In contrast to the *P* cylinder the *M* cylinder splits the C39 U40 and makes contact transiently stacking the 3 nucleotides of the bulge. (G–L) Entry of *P* enantiomer: (G) starting position of *P* cylinder on 1anr1. (H) Cylinder splits the CU nucleotides at the non-bulged strand and (J) pushes the AU base pair (order of microseconds). (K) The bp opens and the cylinder aligns parallel to the GC base pair (order of nanoseconds) and (L) after the AU closes the *P* cylinder is in the centre of the bulge. (M) Transition timescales for the *M* cylinder between states.

The cylinder first associates with the RNA outside the bulge (Fig. 6A and B). It interacts with the two base pairs at the bulge; A22–U40 and G26–C39. The G26–C39 base pair stacks onto a pair of phenyls (drawn from different strands of the cylinder; Fig. 6C). The A22–U40 pair is transient and we see it both paired and unpaired and interacting (stacking) with the cylinder with the U40 having a particular tendency to stack on a phenyl even when not paired (Fig. 6C and D). From here the mechanism of entry proceeds by two very similar processes, differing primarily in whether the A22–U40 is paired during entry or not. The entry process seems to be quicker when A22–U40 is paired, but entry can take place without this pairing (Fig. 6E). The stacking of the paired bases A22–U40, along with the stacking of paired G26–C39 to the cylinder is effectively a V-shaped cleft about the cylinder and is reminiscent of the stacking observed in the 3WJ structure. The bulge itself is initially folded (rather than open) (Fig. 6D) and neutralised by sodium cations, implicating the kinetic contribution of the ionic environment.

As the simulation proceeds, the sodium cations leave and the bulge opens. U25 and C24 are flipped out and stack with each other. The cylinder remains stacked in the V-shaped cleft afforded by U40 (or U40–A22) and C39–G26. The cylinder starts to slide around placing its pyridyls into the bulge; these pyridyls initially encounter the sugar of U23. U25 and C24 swing back and forth with U25 also encountering the pyridyls and transiently stacking with pyridyls as does A22. The crucial point of insertion involves the cylinder stacked with G26–C39, twisting around and inserting through the centre of the bulge (Fig. 6E). It does so facilitated by transient stacking interactions with U25, A22 and C41 which help to guide it into the cavity. With the cylinder now in the cavity, U40–A22 stack onto a pair of the cylinder phenyls, and so (re-)form the V-shaped cleft (now U40–A22; C39–G26) that is similar to two sides of the 3WJ structure. This process has been replicated in 5 independent simulations.

The MSM analysis of this entry process shows just two principal states; once the cylinder has moved from its location just outside and starts to open and enter the bulge, the energy landscape drops rapidly down into the final position where the cylinder is fully inserted and where it remains (Fig. 6M, S147 and ESI Table 5‡).

#### Entry mechanism for *P* enantiomer (Movies S3, S4; ‡Fig. 6G–L)

In the case of the *P* enantiomer from the same starting position (Fig. 6G), the entry mechanism is different but has similar features. The cylinder splits the U25 G26 bulge nucleotides and still stacks the G26–C39 base pair while stabilizing it (Fig. 6H and J). On the other side, the cylinder pyridyls press upon the A22:U40 base pair (Fig. 6K). Within 3 ns the base pair opens, the cylinder stacking aligns to G26–C39 and the A22–U40 base pair re-forms, now enclosing the cylinder in the bulge pocket (Fig. 6L). For the rest of the simulation the cylinder resides in the familiar triangle only this time it is splitting nucleotides U25 and G26 as opposed to C39 and U40 with the *M* enantiomer. U40 now plays a supportive role in stacking the cylinder phenyls and its base pairing with A22 becomes transient. This mechanism has been replicated in 4 independent simulations.

It is instructive that both cylinder enantiomers slide into the cleft down the RNA bases and locate in the V-shaped cleft of the bulge which is similar to that in the 3WJ (Fig. 5). The longer range effect of the insertion is that the helicity of the second stem is disturbed which is consistent with the experimentally observed increased cutting of the C30:U31 by RNAase A.^44^

This bulge insertion is a fascinating illustration of how a three dimensional nano-size agent might target the interior of an RNA structural feature, not by hydrogen bonding to the bases but rather by using its external pi-surfaces to recognize the surfaces inside the structure. To that extent the structure resembles a three-dimensional version of intercalation, and in that context it is notable that Barton has shown that the ‘light-switch’ intercalator [Ru(bpy)_2_(dppz)]^2+^, which doesn't intercalate into duplex RNA, can bind at RNA mismatch sites,^75^ where it is proposed to do so by insertion, with extrusion of the mispaired bases. The organic intercalator ethidium has been proposed to bind one-base bulges in RNA,^76^ and metal complexes bearing a ‘phi’ intercalator suggested to bind near the TAR bulge from cleavage experiments, though that is not yet well understood at a structural level.^77–80^ This insertion of a three-dimensional structure represents a unique and exciting approach to target RNA structures.

### Considerations regarding free energy landscape of RNA-cylinder complex

The simulations suggest that the binding interaction between the cylinder and the TAR-RNA should be characterised as an “induced fit” interaction, meaning that the cylinder does not recognise the bulge cavity in the traditional lock-key manner but rather it induces the precise conformation of the RNA. This complicates the free energy landscape estimation. Although we do get an idea of the landscape using TiCA and PCA we do not believe that the space is sufficiently sampled and therefore MSM probabilities only reflect the sampled space. Mmpbsa techniques cannot be used as removing the cylinder from the final complex exposes a large hydrophobic cavity and an RNA structure that is not in a minimum. Therefore in this paper we have focused on the kinetics and mechanics of the binding process and not on the free energy estimation of the binding. However, in other systems, metadynamics and transition path sampling (TPS) have previously been applied to study the interaction of metal complexes with nucleic acids and proteins.^81,82^

## Methods

### DFT of cylinders

Density functional theory optimisation of the two cylinders were performed in Nwchem 6.8.1 (ref. 83) with SSB-D,^84^ becke97-d,^85^ and TPSSh^86^ with D3 dispersion correction^87^ for the first two and D3BJ for the last with of Def2-SVP basis set. The optimisation was performed under tight driver criteria and increased grid to xfine settings for convergence. Partial charge distribution on atomic positions was calculated with the ESP module under overall restrain of charge. Visualisation of the charge distribution at the surface was done in VMD 1.9.2 (ref. 88) on surface after converting the nwchem output .molden and .esp files to mol2.

### Docking

Autodock vina^69^ was used to create pdbqt files for all solutions of pdb 1ANR as well as the first solutions of coxackievirus stem loop and HIV-2. The cylinder structure after DFT optimisation was entered as a ligand – the searching box was big enough to contain the entire molecule and the cylinder (at least 20 Å away from the biomolecule). Exhaustiveness was set to 1000. Additional docking to just the terminal bases, specifying the docking box to the first 3 base pairs showed that only 2 out of the 20 solutions allowed for capping-mode docking.

### Molecular dynamics simulations

Parametrisation of supramolecular cylinder: already DFT optimised geometries of the cylinders were split into 5 residues (3 ligands and 2 metal ions) that were fed to MCPB.py^89^ that generated parameters for the metal centres at the wB97XD9/6-31G*^86^ level of theory using Gaussian09 (ref. 90) as well as partial charges using RESP. The coordinate and parameter files were converted to gromacs using ParmEd (http://parmed.github.io/ParmEd/html/index.html).

Preparation of parameters with AMBER99SB^66^ was achieved with pdb2gmx program of GROMACS 2019.2 (ref. 91) whereas for the ROC forcefield^51^ it was achieved using tleap program of Amber18 (ref. 92) and the files provided in ref. 51. The parameters and coordinates were then converted to gromacs using Parmed.

In all systems, unless otherwise stated, the RNA was put in a dodecahedral box with edges at least 1.5 nm from the solute filled with TIP3P water. Initial minimisation was carried to at least 500 kJ mol^−1^ nm^−1^ or 50 000 steps followed by heating and NVT equilibration for 1000 ps using V-rescale modified Berendsen thermostat, coupling the cylinder with the RNA at 310 K. All simulations use 2 fs time step and Parrinello–Rahman pressure coupling and PME electrostatics at 1.0 nm cut-off. Attempts to run the simulation with a 4 fs time step led quickly to blow up of the system, although 3 fs time step was more stable.

After completion the compressed trajectories (.xtc) were analysed to remove periodic boundary conditions and rotations using gromacs' trjconv program. After removing the water the trajectories were analysed with pyemma2.5.6 and pyemma 2.5.7,^60^ barnaba.^62^ Free energy calculations used g_mmpbsa.^93^

We also explored simulations for the ruthenium cylinder (total 17.3 μs) in place of the iron cylinder. The ruthenium cylinder behaved analogously in its binding, though its movement was slower due to the increased molecular mass.

### Simulation analysis

To analyse the simulations and identify different micro-states on the energy landscape of each run, we followed the Pyemma workflow.^60^ The workflow involves principal component analysis, time dependent component analysis, and Markov state modeling and Perron cluster cluster analysis.

To identify the best features to apply the workflow to, we explored a variety of potential different features to see which best captured the kinetic variance that occurred during the simulations:

1. Position of centre of mass (COM) of each residue is a low dimensional and relatively efficient way to capture different states, including simulations that involve one or more cylinders.

2. Taking advantage of the fact that each residue has an atom named N3, which is away from the backbone, we created a matrix of distances between these N3 atoms, which although high in dimensionality captures nearly all the kinetic variance. For the cylinder simulations, we also added the distances of the metal ions (Fe or Ru) and the resulting matrix can capture adequately the kinetics of the system during the simulation.

3. The distances between the phosphorus atoms in the backbone.

Of these approaches 2 proved the most useful and was applied to all the simulations.

For each simulation, Principal Component Analysis (PCA) was carried and the projections between the first 4 PCs are plotted, followed by time-lagged independent component analysis (TICA) for lag times 1 to 5000 steps. The lag time for which the fewer number of TICA dimensions were necessary to capture 95% of the kinetic variance was chosen for further analysis. The number of clusters was chosen by examining the convergence with regards to VAMP2 as described the original paper and http://www.emma-project.org/latest/index.html. Lag times for MSM model were chosen from the convergence at timescales of identified processes. Only models that used all of the states and could pass the Chapman–Kolmogorov test were continued to Perron-cluster cluster analysis (PCCA)which led to extraction of states with certain probability and structure in pdb format. Not all simulations were long enough to produce an appropriate Markov state model, and it should be noted that the Markov state models as used here are meant to describe or sum up the particular simulations and not the whole system.

The extracted state and the full length of the simulation were analysed with Barnaba:^62^ all long production molecular dynamics runs, as well as states identified by PCCA, were analysed using barnaba resulting in 2D Leontis/Westhof classification^61^ of base interactions as well as E-RMSD as defined by barnaba software, RMSD and J-couplings.

## Conclusions

This study provides an unprecedented platform to inform design of agents that target different important RNA structural motifs found in nucleic acid nanoscience and biology, such as this bulge cavity present in the UTR of many different viruses. We show that MD simulations, in conjunction with Markov state modeling, allow the dynamic conformational landscape of RNA to be probed and thus different and more relevant binding modes and capabilities of a potential drug to be identified; by contrast, docking to rigid RNA structures is not sufficient to guide such drug designs. The simulations provide crucial new information, not readily accessible by experiment: they show insertion of the cylinders into the cavity of the RNA bulge in a similar binding to that seen for RNA 3-way junctions; they not only provide insight into the ultimate bound structure but also its wider effect on RNA conformation reducing the RNA conformational flexibility once the cavity is bound; and, for the first time, they provide insight about the molecular mechanism through which a drug might enter a cavity in the RNA UTR, involving stacking on and sliding down bases and base pairs. Together these new molecular insights and the combined modelling and analysis approaches that have enabled them and can be more widely applied, will transform understanding of how to create supramolecular drugs that insert effectively into RNA cavities and can guide new designs against a spectrum of critical RNA viruses that threaten human well-being.

## Author contributions

DFT calculations, MD simulations and MSM analyses were designed and undertaken by LM. MJH conceived the project, which was supervised by MJH and IS. All authors analysed the data and discussed the results, and MJH and LM drafted the paper. All authors commented on the manuscript and contributed to the final version of the manuscript.

## Conflicts of interest

There are no conflicts to declare.

## Supplementary Material

SC-012-D1SC00933H-s001

SC-012-D1SC00933H-s002

SC-012-D1SC00933H-s003

SC-012-D1SC00933H-s004

SC-012-D1SC00933H-s005

## References

[cit1] Velagapudi S. P., Cameron M. D., Haga C. L., Rosenberg L. H., Lafitte M., Duckett D. R., Phinney D. G., Disney M. D. (2006). Proc. Natl. Acad. Sci. U.S.A..

[cit2] Rizvi N. F., Smith G. F. (2017). Bioorg. Med. Chem. Lett.

[cit3] Disney M. D., Angelbello A. J. (2016). Acc. Chem. Res..

[cit4] Disney M. D., Dwyer B. G., Childs-Disney J. L. (2018). Cold Spring Harbor Perspect. Biol..

[cit5] Zaman G. J. R., Michiels P. J. A., Van Boeckel C. A. A. (2003). Drug Discov. Today.

[cit6] Li C. H., Chen Y. (2013). Int. J. Biochem. Cell Biol..

[cit7] Smirnova V., Terenin I. M., Khutornenko A., Andreev D. E., Dmitriev S. E., Shatsky I. N. (2016). Biochimie.

[cit8] De Nova-Ocampo M., Soliman M. C., Espinosa-Hernández W. (2019). Mol. Biol. Rep..

[cit9] Gilmore J., Deguchi K. and Takeyasu K., Nanoimaging of RNA Molecules with Atomic Force Microscopy. in Microscopy and imaging Science: Practical approaches to Applied Reseach and education, ed. A. Méndez-Vilas, Formatex Research Center, 2017, pp. 300–306

[cit10] Comandur R., Olson E. D., Musier-Forsyth K. (2017). RNA.

[cit11] Villordo S. M., Filomatori C. V., Sánchez-Vargas I., Blair C. D., Gamarnik A. V. (2015). PLoS Pathog..

[cit12] E Alvarez D., De Lella Ezcurra Fucito A. L. S., Gamarnik A. V. (2005). Virology.

[cit13] Kelly E. J., Hadac E. M., Greiner S., Russell S. J. (2008). Nat. Med..

[cit14] Damgaard C. K., Andersen E. S., Knudsen B., Gorodkin J., Kjems J. (2004). J. Mol. Biol..

[cit15] Boeras I., Seufzer B., Brady S., Rendahl A., Heng X., Boris-Lawrie K. (2017). Sci. Rep..

[cit16] Yang D., Leibowitz J. L. (2015). Virus Res..

[cit17] Hsue B., Masters P. S. (1997). J. Virol..

[cit18] Li L., Kang H., Liu P., Makkinje N., Williamson S. T., Leibowitz J. L., Giedroc D. P. (2008). J. Mol. Biol..

[cit19] Liao K. C., Chuo V., Ng W. C., Neo S. P., Pompon J., Gunaratne J., Ooi E. E., Garcia-Blanco M. A. (2018). RNA.

[cit20] de Borba L., Villordo S. M., Marsico F. L., Carballeda J. M., Filomatori C. V., Gebhard L. G., Pallarés H. M., Lequime S., Lambrechts L., Sánchez Vargas I., Blair C. D., Gamarnik A. V. (2019). mBio.

[cit21] Fleming A. M., Ding Y., Alenko A., Burrows C. J. (2016). ACS Infect. Dis..

[cit22] Ng W. C., Soto-Acosta R., Bradrick S. S., Garcia-Blanco M. A., Ooi E. E. (2017). Viruses.

[cit23] Sethaphong. L., Singh A., Marlowe A. E., Yingling Y. G. (2010). J. Phys. Chem. C.

[cit24] Kulinski T., Olejniczak M., Huthoff H., Bielecki L., Pachulska-Wieczorek K., Das A. T., Berkhout B., Adamiak R. W. (2003). J. Biol. Chem..

[cit25] Do T. N., Ippoliti E., Carloni P., Varani G., Parrinello M. (2012). J. Chem. Theory Comput..

[cit26] Pascale L., Azoulay S., Di Giorgio A., Zenacker L., Gaysinski M., Clayette P., Patino N. (2013). Nucleic Acids Res..

[cit27] Maity D., Kumar S., Curreli F., Debnath A. K., Hamilton A. D. (2019). Chem.–Eur. J..

[cit28] Selby M. J., Bain E. S., Luciw P. A., M Peterlin B. (1989). Genes Dev..

[cit29] Nifosi R. (2000). Nucleic Acids Res..

[cit30] Musiani F., Rossetti G., Capece L., Gerger T. M., Micheletti C., Varani G., Carloni P. (2014). J. Am. Chem. Soc..

[cit31] Krüger D. M., Bergs J., Kazemi S., Gohlke H. (2011). ACS Med. Chem. Lett..

[cit32] Ennifar E., Paillart J. C., Bodlenner A., Walter P., Weibel J. M., Aubertin A. M., Pale P., Dumas P., Marquet R. (2006). Nucleic Acids Res..

[cit33] Dong H., Ray D., Ren S., Zhang B., Puig-Basagoiti F., Takagi Y., Ho C. K., Li H., Shi P. Y. (2007). J. Virol..

[cit34] Ling H., Fabbri M., Calin G. A. (2013). Nat. Rev. Drug Discovery.

[cit35] Cardo L., Nawroth I., Cail P. J., McKeating J. A., Hannon M. J. (2018). Sci. Rep..

[cit36] Sepehrpour H., Fu W., Sun Y., Stang P. J. (2019). J. Am. Chem. Soc..

[cit37] Casini A., Woods B., Wenzel M. (2017). Inorg. Chem..

[cit38] Pöthig A., Casini A. (2019). Theranostics.

[cit39] Woods B., Silva R. D. M., Schmidt C., Wragg D., Cavaco M., Neves V., Ferreira V. F. C., Gano L., Morais T. S., Mendes F., Correia J. D. G., Casini A. (2021). Bioconjugate Chem..

[cit40] Han J., Räder A. F. B., Reichart F., Aikman B., Wenzel M. N., Woods B., Weinmüller M., Ludwig B. S., Stürup S., Groothuis G. M. M., Permentier H. P., Bischoff R., Kessler H., Horvatovich P., Casini A. (2018). Bioconjugate Chem..

[cit41] Cardo L., Hannon M. J. (2018). Met. Ions Life Sci..

[cit42] Oleksi A., Blanco A. G., Boer R., Usón I., Aymamí J., Rodger A., Hannon M. J., Coll M. (2006). Angew. Chem. Int. Ed..

[cit43] Phongtongpasuk S., Paulus S., Schnabl J., Sigel R. K., Spingler B., Hannon M. J., Freisinger E. (2013). Angew. Chem. Int. Ed..

[cit44] Malina J., Hannon M. J., Brabec V. (2016). Sci. Rep..

[cit45] Kerckhoffs J. M., Peberdy J. C., Meistermann I., Childs L. J., Isaac C. J., Pearmund C. R., Reudegger V., Khalid S., Alcock N. W., Hannon M. J., Rodger A. (2007). Dalton Trans..

[cit46] Pascu G. I., Hotze A. C. G., Sanchez-Cano C., Kariuki B. M., Hannon M. J. (2007). Angew. Chem. Int. Ed..

[cit47] Šponer J., Bussi G., Krepl M., Banáš P., Bottaro S., Cunha R. A., Gil-Ley A., Pinamonti G., Poblete S., Jurečka P., Walter N. G., Otyepka M. (2018). Chem. Rev..

[cit48] Cesari A., Bottaro S., Lindorff-Larsen K., Banáš P., Šponer J., Bussi G. (2019). J. Chem. Theory Comput..

[cit49] Tan D., Piana S., Dirks R. M., Shaw D. E. (2018). Proc. Natl. Acad. Sci. U.S.A..

[cit50] Vangaveti S., Ranganathan S. V., Chen A. A. (2017). Wiley Interdiscip. Rev.: RNA.

[cit51] Aytenfisu A. H., Spasic A., Grossfield A., Stern H. A., Mathews D. H. (2017). J. Chem. Theory Comput..

[cit52] Angelbello A. J., Benhamou R. I., Rzuczek S. G., Choudhary S., Tang Z., Chen J. L., Roy M., Wang K. W., Yildirim I., Jun A. S., Thornton C. A., Disney M. D. (2021). Cell Chem. Biol..

[cit53] Mathews D. H. (2019). Methods.

[cit54] Dans P. D., Gallego D., Balaceanu A., Darré L., Gómez H., Orozco M. (2019). Chem.

[cit55] Gresh N., Sponer J. E., Devereux M., Gkionis K., De Courcy B., Piquemal J. P., Sponer J. (2015). J. Phys. Chem. B.

[cit56] Zgarbová M., Otyepka M., Šponer J., Lankaš F., Jurečka P. (2014). J. Chem. Theory Comput..

[cit57] Pinamonti G., Paul F., Noe F., Rodriguez A., Bussi G. (2019). J. Chem. Phys..

[cit58] Shi H., Rangadurai A., Abou Assi H., Roy R., Case D. A., Herschlag D., Yesselman J. D., Al-Hashimi H. M. (2020). Nat. Commun..

[cit59] Copperman J. and Zuckerman D., Accelerated estimation of long-timescale kinetics by combining weighted ensemble simulation with Markov modelmicrostatesusing non-Markovian theory, 2019, ArXiv.1903.04673, 10.1016/j.bpj.2019.11.1099

[cit60] Scherer M. K., Trendelkamp-Schroer B., Paul F., Pérez-Hernández G., Hoffmann M., Plattner N., Wehmeyer C., Prinz J. H., Noé F. (2015). J. Chem. Theory Comput..

[cit61] Leontis N. B. (2002). Nucleic Acids Res..

[cit62] Bottaro S., Bussi G., Pinamonti G., Reißer S., Boomsma W., Lindorff-Larsen K. (2019). RNA.

[cit63] Zoll J. A. N., Tessari M., Van Kuppeveld F. J. M., Melchers W. J. G., Heus H. A. (2007). RNA.

[cit64] Ohlenschläger O., Wöhnert J., Bucci E., Seitz S., Häfner S., Ramachandran R., Zell R., Görlach M. (2004). Structure.

[cit65] Dayie K. T., Brodsky A. S., Williamson J. R. (2002). J. Mol. Biol..

[cit66] Aboul-ela F., Karn J., Varani G. (1996). Nucleic Acids Res..

[cit67] Orlovsky N. I., Al-Hashimi H. M., Oas T. G. (2020). J. Am. Chem. Soc..

[cit68] Davis B., Afshar M., Varani G., Murchie A. I. H., Karn J., Lentzen G., Drysdale M. J., Bower J., Potter A. J., Aboul-Ela F. (2004). J. Mol. Biol..

[cit69] Trott O., Olson A. J. (2019). J. Comput. Chem..

[cit70] Ditzler M. A., Otyepka M., Šponer J., Walter N. G. (2010). Acc. Chem. Res..

[cit71] Bermejo G. A., Clore G. M., Schwieters C. D. (2016). Structure.

[cit72] Buck D. P., Spillane C. B., Collins J. G., Keene F. R. (2008). Mol. Biosyst..

[cit73] Spillane C. B., Smith J. A., Buck D. P., Collins J. G., Keene F. R. (2007). Dalton Trans..

[cit74] Malina J., Scott P., Brabec V. (2020). Chem.–Eur. J..

[cit75] McConnell A. J., Song H., Barton J. K. (2013). Inorg. Chem..

[cit76] Ratmeyer L. S., Vinayak R., Zon G., Wilson W. D. (1992). J. Med. Chem..

[cit77] Alberti E., Zampakou M., Donghi D. (2016). J. Inorg. Biochem..

[cit78] Neenhold H. R., Rana T. M. (1995). Biochemistry.

[cit79] Lim A. C., Barton J. K. (1997). Bioorg. Med. Chem..

[cit80] Carter P. J., Cheng C. C., Thorp H. H. (1998). J. Am. Chem. Soc..

[cit81] Bernardi R. C., Melo M. C. R., Schulten K. (2015). Biochim. Biophys. Acta, Gen. Subj..

[cit82] Wragg D., de Almeida A., Bonsignore R., Kühn F. E., Leoni S., Casini A. (2018). Angew. Chem. Int. Ed..

[cit83] Aprá E., Bylaska E. J., de Jong W. A., Govind N., Kowalski K., Straatsma T. P., Valiev M., van Dam H. J. J., Alexeev Y., Anchell J., Anisimov V., Aquino F. W., Atta-Fynn R., Autschbach J., Bauman N. P., Becca J. C., Bernholdt D. E., Bhaskaran-Nair K., Bogatko S., Borowski P., Boschen J., Brabec J., Bruner A., Cauët E., Chen Y., Chuev G. N., Cramer C. J., Daily J., Deegan M. J. O., Dunning Jr T. H., Dupuis M., Dyall K. G., Fann G. I., Fischer S. A., Fonari A., Früchtl H., Gagliardi L., Garza J., Gawande N., Ghosh S., Glaesemann K., Götz A. W., Hammond J., Helms V., Hermes E. D., Hirao K., Hirata S., Jacquelin M., Jensen L., Johnson B. G., Jónsson H., Kendall R. A., Klemm M., Kobayashi R., Konkov V., Krishnamoorthy S., Krishnan M., Lin Z., Lins R. D., Littlefield R. J., Logsdail A. J., Lopata K., Ma W., Marenich A. V., Martin del Campo J., Mejia-Rodriguez D., Moore J. E., Mullin J. M., Nakajima T., Nascimento D. R., Nichols J. A., Nichols P. J., Nieplocha J., Otero-de-la-Roza A., Palmer B., Panyala A., Pirojsirikul T., Peng B., Peverati R., Pittner J., Pollack L., Richard R. M., Sadayappan P., Schatz G. C., Shelton W. A., Silverstein D. W., Smith D. M. A., Soares T. A., Song D., Swart M., Taylor H. L., Thomas G. S., Tipparaju V., Truhlar D. G., Tsemekhman K., Van Voorhis T., Vázquez-Mayagoitia A., Verma P., Villa O., Vishnu A., Vogiatzis K. D., Wang D. D., Weare J. H., Williamson M. J., Windus T. L., Woliński K., Wong A. T., Wu Q., Yang C., Yu Q., Zacharias M., Zhang Z., Zhao Y., Harrison R. J. (2020). J. Chem. Phys..

[cit84] Swart M., Solà M., Bickelhaupt F. M. (2009). J. Chem. Phys..

[cit85] Varbanov H. P., Jakupec M. A., Roller A., Jensen F., Galanski M., Keppler B. K. (2013). J. Med. Chem..

[cit86] Kepp K. P. (2016). Inorg. Chem..

[cit87] Grimme S., Antony J., Schwabe T., Mück-Lichtenfeld C. (2007). Org. Biomol. Chem..

[cit88] Humphrey W., Dalke A., Schulten K. (1996). J. Mol. Graph..

[cit89] Li P., Merz K. M. (2016). J. Chem. Inf. Model..

[cit90] Frisch M. J., Trucks G. W., Schlegel H. B., Scuseria G. E., Robb M. A., Cheeseman J. R., Scalmani G., Barone V., Petersson G. A., Nakatsuji H., Li X., Caricato M., Marenich A., Bloino J., Janesko B. G., Gomperts R., Mennucci B., Hratchian H. P., Ortiz J. V., Izmaylov A. F., Sonnenberg J. L., Williams-Young D., Ding F., Lipparini F., Egidi F., Goings J., Peng B., Petrone A., Henderson T., Ranasinghe D., Zakrzewski V. G., Gao J., Rega N., Zheng G., Liang W., Hada M., Ehara M., Toyota K., Fukuda R., Hasegawa J., Ishida M., Nakajima T., Honda Y., Kitao O., Nakai H., Vreven T., Throssell K., Montgomery Jr J. A., Peralta J. E., Ogliaro F., Bearpark M., Heyd J. J., Brothers E., Kudin K. N., Staroverov V. N., Keith T., Kobayashi R., Normand J., Raghavachari K., Rendell A., Burant J. C., Iyengar S. S., Tomasi J., Cossi M., Millam J. M., Klene M., Adamo C., Cammi R., Ochterski J. W., Martin R. L., Morokuma K., Farkas O., Foresman J. B. and Fox D. J., Gaussian 09, Gaussian, Inc., Wallingford CT, 2016

[cit91] Abraham M. J., Murtola T., Schulz R., Pall S., Smith J. C., Hess B., Lindahl E. (2015). SoftwareX.

[cit92] Salomon-Ferrer R., Case D. A., Walker R. C. (2013). Wiley Interdiscip. Rev.: Comput. Mol. Sci..

[cit93] Kumari R., Kumar R., Lynn A. (2014). J. Chem. Inf. Model..

[cit94] Thompson S. J., Thompson S. E. M. and Cazier J.-B., CaStLeS (Compute and Storage for the Life Sciences): a collection of compute and storage resources for supporting research at the University of Birmingham, 10.5281/zenodo.3250616

