# Supplementary material for: Targeting structural features of viral genomes with a nano-sized supramolecular drug
Source: Chem Sci. 2021 Apr 5;12(20):7174–84. doi: 10.1039/d1sc00933h (PMC8153246; doi:10.1039/d1sc00933h)
Supplement: SC-012-D1SC00933H-s005 [file SC-012-D1SC00933H-s005.pdf]

Electronic Supplementary Material (ESI) for Chemical Science.
This journal is © The Royal Society of Chemistry 2021

Supplementary information

Targeting structural features of Viral genomes with a nano-sized supramolecular drug

*Larry Melidis,[1] Iain B. Styles[1,2] and Michael J. Hannon[1,3] **

[1] Physical Sciences for Health Centre, University of Birmingham, Edgbaston, Birmingham B15 2TT, UK.

[2] School of Computer Science, University of Birmingham, Edgbaston, Birmingham B15 2TT, UK.

[3] School of Chemistry, University of Birmingham, Edgbaston, Birmingham B15 2TT, UK.

*Correspondence to: m.j.hannon@bham.ac.uk

Contents

		Figures	Tables	Page
Section A:	Additional Results and Discussion	S1-S9		2
Section B:	Approach used to analyse the simulation results	S10-S173	T1-8	13
References				125

Part A: Additional Results and Discussion

1. DFT of supramolecular cylinders containing Fe and Ru

The first task was to create a computational model of the metallo-supramolecular cylinders, with suitable charge distributions, that could then be used in the MD simulations. We considered two different metal cylinders, one containing iron(II) and the other ruthenium(II), but otherwise identical (Main text Figure 1).

Both metal centres have a d^6 electronic configuration and exhibit a preference for octahedral coordination; although ruthenium(II) is slightly larger than iron(II) (metal ligand bonds of ~2.1Å instead of~2.0Å in Fe) this has only small effects on the overall dimensions of the cylinder, which are of the order of 2nm in length by 1nm in diameter - similar in size to the alpha helix of zinc finger units in proteins. The cylinders are helical and exist in two enantiomeric forms, exhibiting a right handed (P) or left handed (M) twist[17], and the binding of both enantiomers will be discussed. Although the binding of enantiomers to chiral RNA will be different, biophysical studies show both to bind in the same location with similar affinities. (Both enantiomers of both cylinders bind strongly and displace the TAT peptide from the TAR RNA).[9] X-ray crystal structures are available for both cylinders (Fe and Ru) and these were used as starting point for DFT calculations.[1,7] The parameterisation are explained in methods. The calculated DFT and experimental X-ray crystal structures are almost identical, and as expected RuCyl and FeCyl have very similar distribution of charges at the surface of the molecule (Fig. S1).

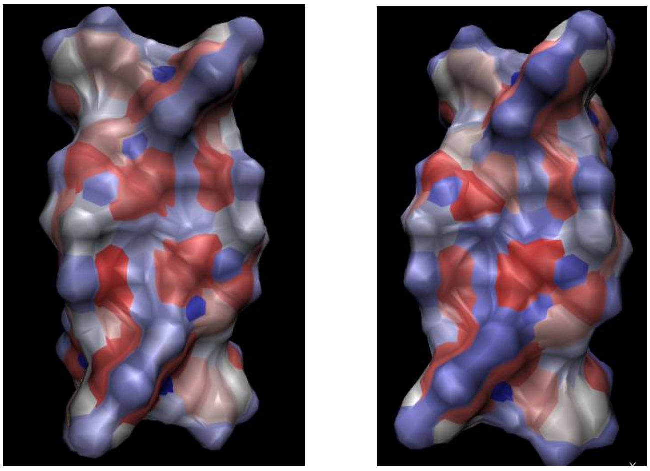

Fig. S1 Surface charge distribution in Fe cylinder (left) and Ru cylinder (right)

2. Docking of cylinders to TAR RNA

Disney has recently used docking to screen libraries of small molecules binding to RNAs including TAR [18]. We initially undertook simple docking calculations as described in methods using all 20 structures from pdb;1anr TAR RNA NMR study. The results are dominated by different forms of bulge region binding. While the two enantiomers do show slightly different binding energies, the Autodock Vina as other docking software (used as it is one of few that allow incorporation of first row d-block metal centres) tend to underestimate the electrostatic contribution when a charged molecule is involved. Nevertheless the docking scores are high compared to other small molecule drugs assessed by this method reflecting the larger available surface of the cylinder.

It is interesting to compare the results of docking with overall results of subsequent MD simulations. In particular in the MD simulations, capping of the open terminal bases is seen with both enantiomers and is relatively stable binding mode (more than 2μs). Although this binding mode is a local minimum in the interaction of cylinders with TAR it highlights the limitations of

docking in targeting Nucleic Acids because, across all 20 NMR solutions of TAR RNA, the terminal bases are coplanar only in one (the 9th). Consequently only in this structure solution does the docking reveal the end capping binding mode as a potential binding site. So docking outcomes are constrained by the rigid RNA structure(s) used in the docking, whereas in reality – as we shall see – RNAs are highly fluxional and dynamic molecules that access much structural space. Thus while such simple docking studies are valuable for high throughput screening they might be more suited to small molecules where the molecule is less likely to have a major effect on RNA conformation. For the larger cylinders the size of the binding surface means that induced conformational change is more likely and so more sophisticated MD can offer greater insight into the interaction. Crucially, while the docking showed bulge region binding, bulge insertion by the cylinder was not observed.

a)

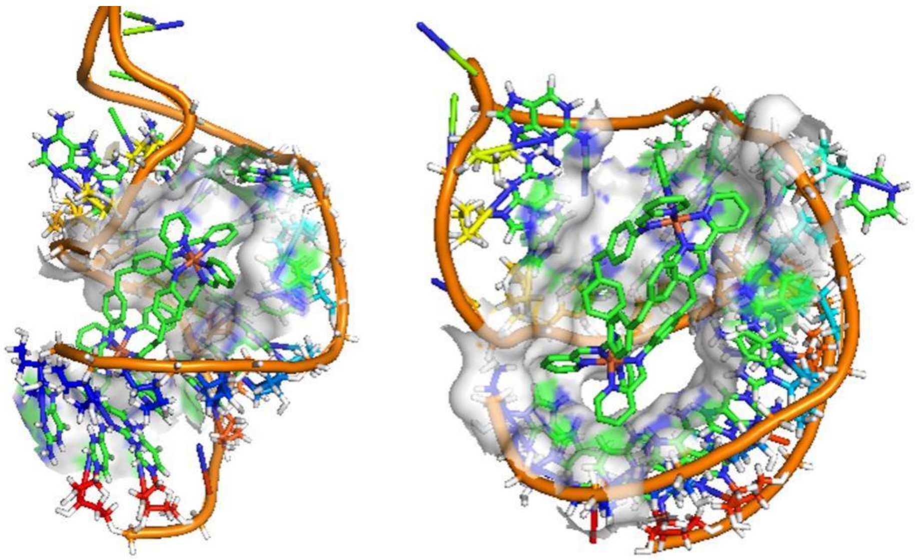

b)

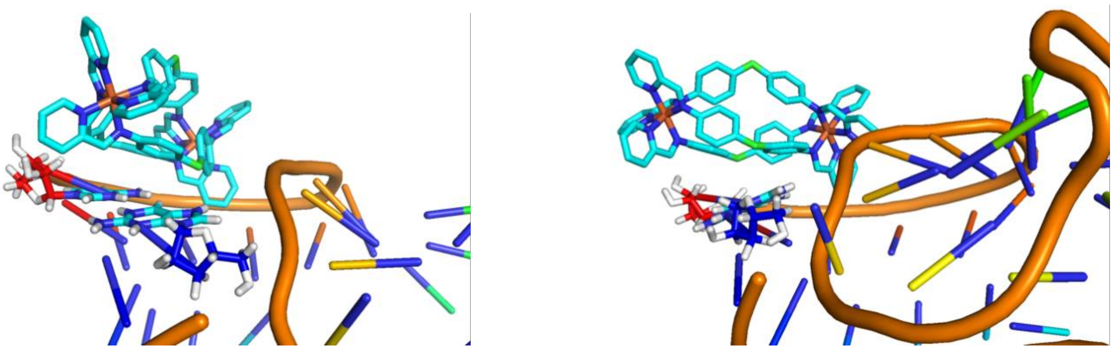

Fig. S2 (a) Docking of M cylinder on the 8th solution (left) with binding score of -15.4 kcal/mol and docking of P cylinder on the 9th solution (right) with binding score of -14.8 kcal/mol. (b) M cylinder in capping binding mode after docking on 9th solution (top) binding score of -9.6 kcal/mol . P cylinder in capping binding mode on the 9th solution (-7.8kcal/mol)

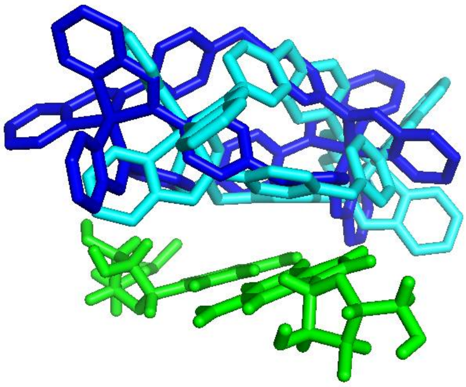

Fig. S3 Capping of the cylinder: end-stacking observed in docking results of the 9th NMR solution with the P (blue) and M(cyan) cylinders.

3. Molecular dynamics simulations of unbound RNAs

To initiate our MD simulations, we needed first to establish whether the new (Rochester-Matthews) force-field recently tested for small RNAs (<10 residues)[19] would reproduce the experimental observations for longer and more complex RNA structures. As there is a growing interest in RNA-targeting more broadly, and to have confidence in the approach, we wished to verify on more than one RNA structure, and not solely on the target TAR RNA structure that is the focus herein. Aside from TAR RNA, we undertook simulations (2 microseconds each) of coxsackievirus stem loop and polio virus stem loop. These were selected as they have mismatched pairing on a single stem, allowing us to probe the approach against a dynamically complex yet potentially drug-able site, and because they have established NMR structures to which we could compare our results.) Overall we have performed over 70 simulations of at least 1μs and up to 10μs overall 200μs including several shorter runs with varying initial conditions.

3a. Coxsackievirus stem-loop The first simulation was of the Coxsackievirus D stem (pdb: 1RFR). This structure forms part of a three stem array (a frequent RNA motif), and the D-stem is the most conserved of the component parts. The sequence contains both Watson-Crick and non-Watson-Crick parings and a loop and is thus an interesting first challenge. Two simulation runs of 2μs each were performed with 50mM ionic strength (NaCl). This is, to the best of our knowledge the first example of this force-field being applied to model Coxsackievirus stem.

The overall structure dynamics are consistent with the NMR observations, showing a well defined structure in the mismatch region (U6,U8,U23-U25). U24 is the only of these residues that has a stable WC bonding with the opposite C7, and not a transient pairing with the -1 and +1 of the opposite strand. The bases of the tetraloop comprise the relatively stable wobble base pair of U13 and G18, but also G17 which shows transient trans bonding with C14 either as trans Sugar/WC or trans WC. This has also been identified by NMR. In this loop region the stacking between A15 and C14 is largely preserved as is the overall stacking between the U residues and the G; these are likewise as described in NMR. The formation of an electronegative region , identified by NMR, is also seen in the MD simulation with a large number of Na cations constantly clustered in that location. An aspect not observed in the NMR analysis is a transient non-traditional pairing of A12-A20, which leaves U11 as temporary bulge. This changes the overall structure of that region to a ladder-like (less helical) stacking and changes the overall distance between the loop and the base of the stem.

Principle components analysis was not able to resolve states by projection of the first two components although slight differentiation can be seen in projections that include the third. TiCA using lag time of 200steps (2ns), as expected proved more revealing and describes a more diverse landscape with at least 4 distinct points in the first two projections and many more in projections including the 3rd and 4th components. Based on projection between the first two components and a lag time for Markov model of 800 steps four clean (distinct) states have been identified, the 4th

being the most stable. These four states, and the microstates that underpin them, are all consistent with the reported NMR and give confidence in our approach.

3b. Polio stem-loop We next turned to the Polio Y-stem CU variant (pdb: 2GRW). This structure is shorter than the Coxsackie D-stem, and contains a single mismatch flanked by Watson-Crick paired bases, giving the opportunity to explore the approach with a structure where a single nucleotide bulge might arise. An analogous 5 μs simulation was run again at 50mM ionic strength, with the NMR ground state as the starting structure. The Y-stem of the poliovirus shows similar dynamics of the loop structure CGAAAG to that in the coxsackievirus (UCACGG) despite the UG wobble now being a Watson crick CG. The simulation is sufficiently long to identify problematic pairing between C5 and U18, explaining the observed absence of imino and amino proton NMR signals for these resonances (despite that pairing being proposed in the NMR structure). Strong stacking of the A2-U20 to C4-G19 and C5,U18 to U6:A17 is also stable throughout the simulation and consistent with the NMR data. The C4 G19 residues do not have typical A-RNA torsion angles because the stacking with the adjacent (problematic) pair is less extensive. G19 can transiently base pair with A3 and stack with C5 and in a similar way U18 can base pair to U6. Finally, in the loop region A13 is transiently both trans or cis Sugar/WC paired with G10. Strong stacking patterns all the way to the very stable C9:G14 base pair help the stability of the loop.

PCA showed a dominant and a very minor cluster although the TiCA analysis, with a lag time of 2ns, resolved those peaks to four. Based on projection between the first two components and a lag time for Markov model of 400steps, four clean states have been identified, the 4th being the most stable. It is striking that the MD simulations reveal and explain features that are observed in the NMR structural data, but have not previously been satisfactorily captured. This gives further confidence in this approach.

3c. HIV2-TAR As a final step before addressing the HIV-1 TAR target, we next checked the applicability of the simulations to the closely related HIV-2 TAR (pdb: 1AJU) which differs solely in the size of the bulge (2 bases rather than 3 bases). A 3μs simulation was undertaken starting from pdb:1aju after removing the bound ligand argininamide. The results are useful as an initial understanding of the behaviour of bulges. The bulge region is rich in different transient pairings, and more specifically between residues A22,U23,U25,U40 and U40 C41, including Hoogsteen sugar bonding. At the loop, A35 is transiently involved in cis Hoogsteen -Sugar Edge with G34 C30 or G32. C29 has strong binding with G34, as it has been observed with HIV1 TAR. Stacking on both stems is strong and only breaks at A22<<U23. U24 is less involved in pairing than other residues and often bulges out of helix/stacking. The simulation was not able to sample the G26 U23 interaction that is described at the NMR, which is likely a result of the ligand binding between A22 and U23.

3d. HIV1-TAR The pdb: 1anr structure is the only drug-free HIV-1 TAR structure. There are 20 NMR solutions proposed that lie at different local minima on the landscape. We started with five such minima (first, third, fourth, seventh and twelfth). In each case the 2μs simulation retained the characteristics consistent with the NMR structure and did not deviate into unnatural conformations. From each starting point similar features can be observed which indicates that the forcefield can reproduce transitions within the landscape within a few μs.

The helical regions remain relatively stable with strong WC base pairing. The only stem base pair not retaining the WC pairing is A22:U40, which often drifts apart as the U40 retains strong stacking with C39. It is often the case that U40 seems to be in the 2nd rather than the first stem.

While a variety of transient base-pairings of all types were observed in the bulge region, as expected from the experimental NMR observations, no new stable base pairings were observed apart from that between C30 and G33/4 which is not observed by NMR but is observed in gel electrophoresis. On the un-bulged strand stacking is strong and continuous, but this is a lot less evident on the bulge strand. Of the three bulged nucleotides, U23 and C24 are more likely to stack

whereas U25 is the most likely to be fully outside the helix and can even create long range interactions with the loop nucleotides (G33 and A35) creating a transient folding up of the second stem. Such a folding was not observed in the HIV-2 TAR simulation.

The loop region is characterised by limited stacking between bases and common WC pairing between C30 and G34. Transient non-WC pairing can include C30 cis or trans WC/Hoogsteen to A35.

Examining the runs individually; The first simulation starting from 1anr1 identified 3 distinct states, that can be recognised even by the PCA analysis. All are energetically and conformationally close together as seen by the RMSD and ERMSD. PCCA analysis shows one to be in much higher occupancy, clearly the ground state. A second simulation also starting from 1anr1 sampled a wider conformational space. Base pairing of stems was retained although stacking between C19:G43 and A20:U21 was not, although it is observed in the NMR. After that stacking does continue all the way to the loop. At the loop a few different conformations were sampled that mostly gave rise to the different MSM states identified. C30 base pairs with either G33 or G34. In the bulge region U40 is stacked strongly with C39 but not always to C41 and transient, short lived pairing takes place between all bulge residues and either of C39 and U40, with pairing types including both sugar and Hoogsteen edges as well as in the trans position. MSM analysis gave 6 different states.

The simulation starting from 1anr3 yielded 5 local minima in the TiCA projections and CK test allowed for 5 states in MSM analysis. Overall, stacking and pairing throughout the stems is conserved and transient pairing within the bulge region is similar to that of the previous run. Most importantly the 2nd state is very reminiscent of the ground state.

Going up the energy ladder from the starting conformation one might expect to encounter more structures that deviate significantly from the ground state. Nevertheless starting from the 4th solution 1anr4, most of the important structural features were retained. Pairing and stacking remains consistent with the exception of the U23 to C24 stacking. PCA revealed 3 stationary points which become 5 with TiCA. Also notable is that from this state up, examining the first 4 TiCA vectors instead of just two showed much higher diversity. In the loop, pairing C30:G34 is seen again, as well as the usual transient non traditional pairing, but now interactions between U23 and U38 and trans Hoogsteen to sugar between U23 and C39 are observed. Stacking of U40 to C39 remains strong but stacking of U23 to C24 was less prevalent.

The 1anr7 and 1anr12 structures are quite different from the ground state and this brings challenges for the simulation: Specifically the loss of A helix structures which is characterised by the overall elongation of G17 to G33 distance can be testing to any forcefield. Nevertheless, starting from 1anr7, the stacking and pairing remains consistent. PCA identified 2 states whereas TiCA suggested 6 states and the CK test is also passed with 6 states. The first 4 states are reminiscent of the ground state with different loop configurations, namely sugar to Hoogsteen between C30 and A35, or less often trans WC to Hoogsteen. In the other two states, U25, which generally points outside the bulge can create temporary long range interactions with loop residue G33.

Starting from 1anr12, which is also very elongated with a sharp backbone kink in the bulge area, also retrieved most of the properties of the ground state. Pairing and stacking remain consistent for the stems. In the loop the common C30 to G34 pairing is stable along with a transient Hoogsteen to sugar between A35 and C30. In the bulge region stacking between C39 and U40 is strong and most of the transient non traditional base pairings are also seen. PCA revealed 2 states whereas TiCA revealed 5.

In addition to the unbound 1anr structure, there are some TAR RNA structures with various different bound drugs, so for comparison we also explored as a starting point one such structure (the only solution of pdb;1UUI) from which we had removed the drug. The structure of, after removing the ligand, has some differences with the 1anr structure: Pairing on the stems is the same, but stacking is disturbed before the bulge, probably since U23 is WC paired with A27.

When using this as the starting point for a 2μs simulation, the loop folded back onto the bulge (from which the ligand had been removed) forming interactions from U23 and C24 to A35, and the stem remained folded for much of the simulation. The bulge stacking did not return to the transient

pairings seen in the earlier simulations. PCA analysis of the simulation revealed 3 states and TiCA 6, which was also passed the CK test on with MSM with the 6th state being ground state of this run.

The simulation demonstrates how ligand binding can modify the structure and dynamics of the TAR RNA and again highlights that docking, while a useful guide, may miss key features and opportunities.

3e. Consideration of the forcefields The Rochester forcefield[19] seems to behave well for every case of RNA molecular dynamics, even in cases outside the ground state of the structure in question. That can give more trust for ligand binding molecular dynamics which by definition can transition the conformation to a non ground one. The tendency to drive the conformation towards the ground state even in a couple of microseconds might be a bias of the forcefield, or a true frequency sampling of the ground state.

We also explored the alternative (older) forcefield, AMBER99SB[10] to see whether our selected forcefield was indeed more effective. An issue with this alternate forcefield is that starting from structures other than the ground state generally resulted in drifting towards unnatural solutions even within 1 [illegible]s. As has been previously reported[20], the older forcefield behaves admirably when starting structure is the first solution 1anr1. Pairing and stacking of stems are consistent, up to C24, U40 is stacked more often to C39 than C41, U25 is transiently WC paired with U40 and C30 to G34. However PCA analysis showed 6 peaks which is significantly higher than expected for a ground state. Where things break down is when the simulation starts from the seventh state 1anr7: Stacking is completely lost as well as the base pairing of the first stem.

4. Simulations of Cylinder binding to the coxsackievirus and polio RNA stem-loops

With the RNA simulations, showing the different conformational space that the RNA could access and the results validated by the observed NMR data, we now turned to explore the binding of the enantiomers of the two metallo-supramolecular cylinders, using the iron(II) cylinders.

To get an initial view of where cylinder might potentially bind to a stem-loop, and to test/establish analysis methods, we performed initial simulations of the M and P enantiomers of the cylinder with the coxsackievirus and poliovirus stem-loops.

4a. Coxsackievirus stem-loop Two P enantiomer cylinders were added, placed away from the RNA, and each quickly settled to a binding mode that they retained till the end of 2 μs simulation. One was located on the groove and loop region assisted by stacking of the loop nucleotides, and the second lower on the groove but moving through the simulation up and down the groove (Fig. S3).

For the M enantiomers, one settled at the groove also interacting with the loop nucleotides and the other capped the open base pair at the base of the stem (Fig. S3).

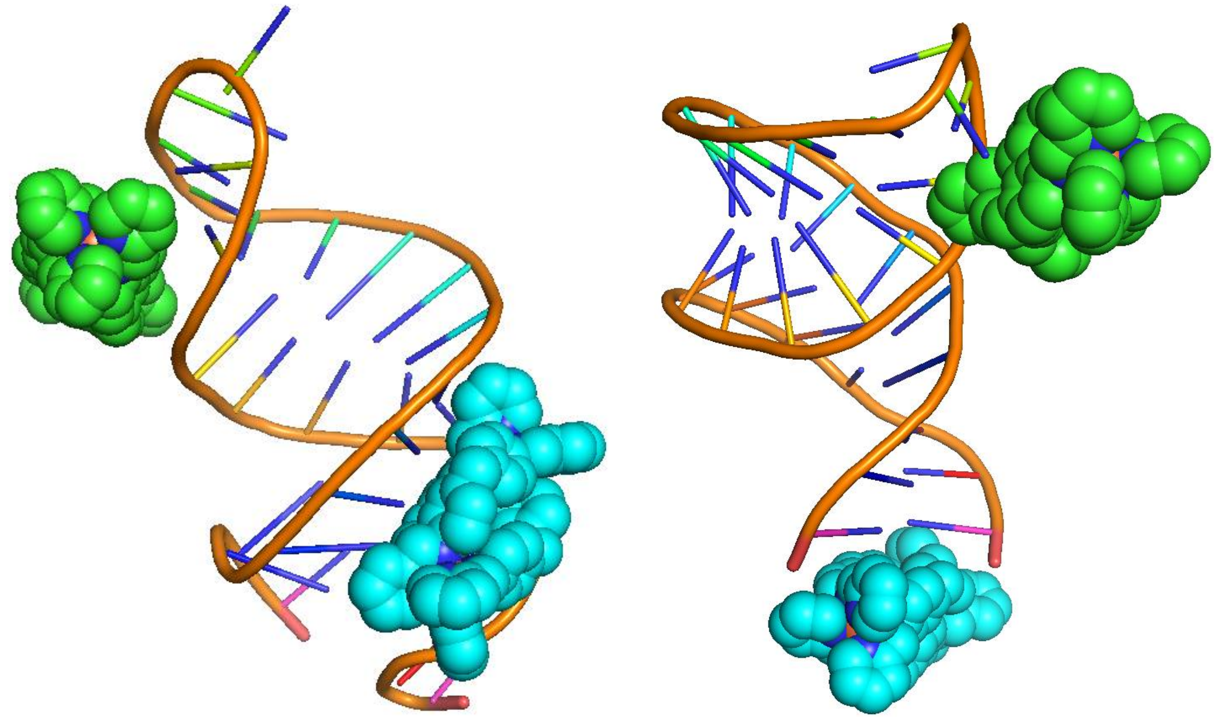

Fig. S4 Stable binding positions of the two P enantiomers (left) and M enantiomers (right) on the Coxsackievirus stem-loop in the simulations.

4b. Polio stem-loop Just one P enantiomer cylinder was added to the smaller polio Y-stem. The cylinder quickly docked to the open base pair at the end of the stem, and stayed there throughout the 2μs simulation (Fig. S4.1). This means that across the two test RNA simulations (coxsackievirus and polio) this binding mode was seen for both enantiomers; the binding mode has also been experimentally observed (both enantiomers) in X-ray crystal structures, for both RNA and DNA[21], which gives confidence that the simulation is reproducing experimentally feasible bindings. This binding mode is also observed in some of the subsequent simulations with TAR RNA (see B5a).

In the M enantiomer simulation, the cylinder initially binds on the top of the groove near the loop but later cascades down towards the base of the stem (Fig. S4.2).

a)

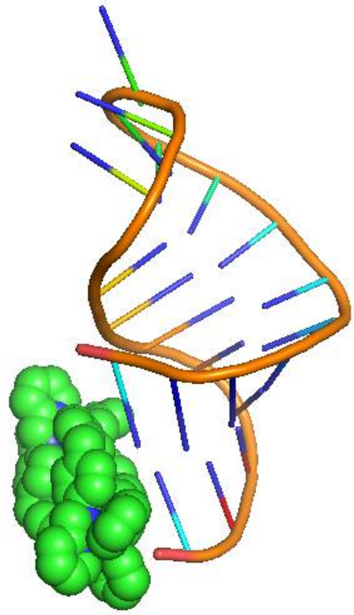

b)

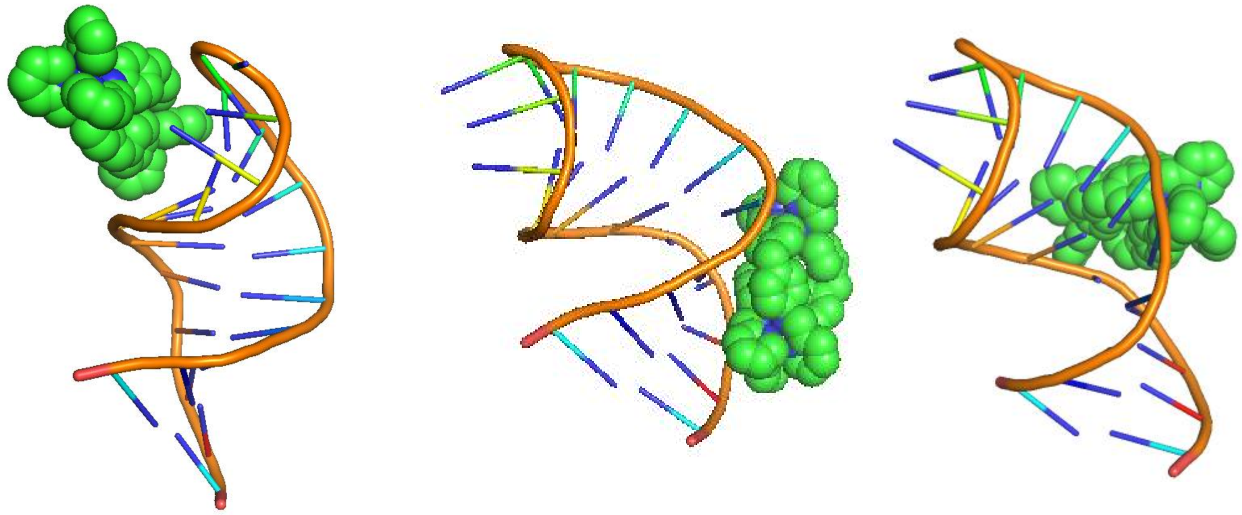

Fig. S5 (a) Stable binding position of the P enantiomer end-stacking on the Polio stem-loop in the simulations. (b) Simulation of polio stem-loop with the M cylinder showing the cylinder briefly binding to the loop and later moving along the groove.

5. Simulations of Cylinder binding to the HIV-1 TAR RNA.

Understanding that capturing an event of insertion to the bulge can be challenging in computationally available timescales we conducted over 50 simulations starting from 1anr1/3/7/12 and 1UUI again with both M and P enantiomers of Fe. Across these simulations a variety of binding modes are observed:

5a. cylinder capping the terminal base pairs; In numerous cases the cylinder (both enantiomers) found an easily kinetically accessible local minimum, occupied for at least 1μs at a time on capping the G17:C45 bases. This binding mode (Fig. S5.1), that has been observed in X-ray crystal structures, gives confidence that the simulation is replicating an experimentally validated binding mode. To assess how well the forcefield and the parametarisation (including now the cylinder) reproduces the mode as captured by the crystal structure we extracted the G17:C45 bases and the cylinder from a frame of the longest lived position and we then optimised that structure at the ssb-d-D3/LANL2DZ (DFT and semi-empirical (PM7)) and superimposed to the crystal structure of either the DNA or the RNA (pdb: 4JIY). The overlap (Fig. S5.2) is extremely good, not only implying that the forcefield is working as desired, but that the crystallographically observed binding is not being affected by packing forces. Two phenyl rings, one each from two organic ligand strands, of the cylinder form the pi-surface onto which the G17:C45 bases are stacked. These phenyl rings at the centre of the cylinder can rotate about the 1,4-phenylene axis when the cylinder is free in solution; in the optimisation they are slightly more coplanar (and therefore better stacked) than in the crystal though the difference in dihedral twisting that gives rise to this is very small.

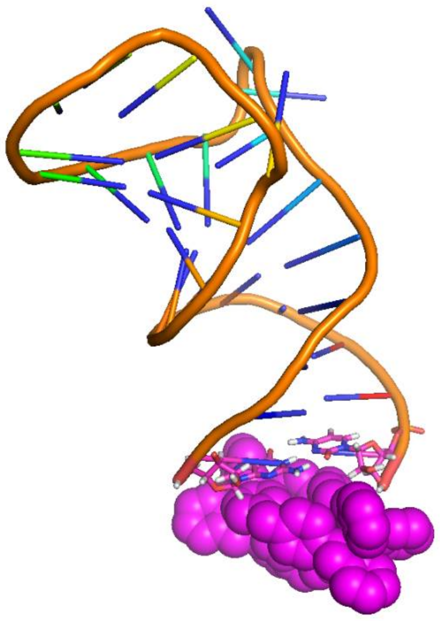

Fig. S6 End-capping of the cylinder observed in an MD simulation.

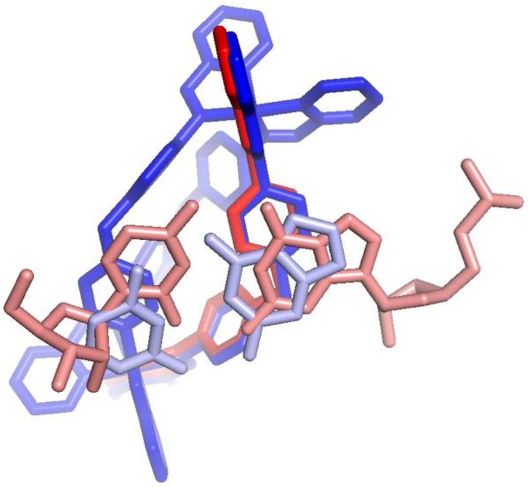

Fig. S7 Capping of the cylinder: the end-stacking experimentally observed in crystal structure 4JIY (red), overlain with that observed in an MD simulation followed by DFT optimisation (blue) .

Figure S5.1

5b. Loop binding The cylinder can take advantage of unpaired open bases of the loop and bind transiently there (as also seen in coxsackievirus stem). This binding mode is less commonly observed in the simulation compared to other binding modes and is not as stable. The fact the binding is only transient in the simulation is an important observation as loops are a common feature in RNA structures (and indeed in non-cannonical DNA structures such as G-quadruplexes and i-motifs).

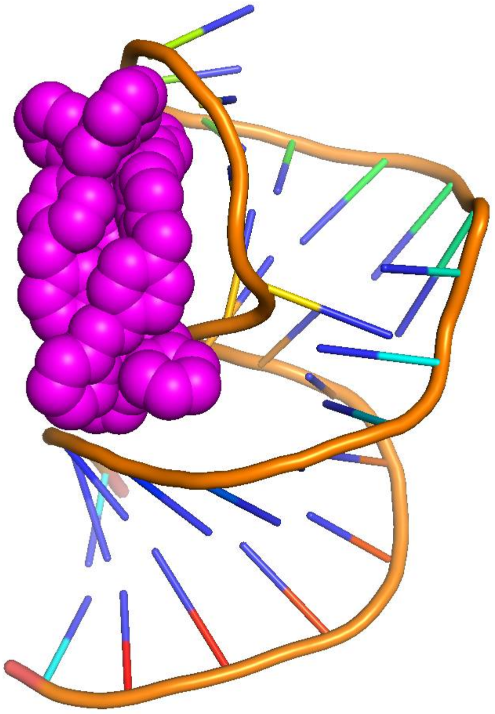

Fig. S8 Loop binding of a cylinder (P enantiomer) observed in an MD simulation

5c. Groove binding A more commonly observed binding mode is the grooves, primarily the groove of the first stem. The residence time for the M enantiomer on average is longer than for the P implying that the M enantiomer may have a higher binding affinity for this mode although the kinetics were not adequately sampled to quantify difference.

5d. Bulge area All the other binding modes are found in, around or on the bulge and this is where the cylinder is most frequently found, with M and P being very similar in their preference for this location. Conformations that involve the loop bridging to the bulge (U25) can be stabilised for longer, with the cylinder sitting on top of the bridge or mediating stacking. In the absence of the bridge, the cylinder can also sit between the bulge and the opposite RNA strand, in a mode in which it opens up the base pairing protecting the TAT binding site. The cylinder is also observed in a stable mode which splits two of the bulge nucleotides (C24 and U25) creating a pocket for the cylinder to bind in. The stacking in this pocket, with the bulge strand tracking an organic ligand strand of the cylinder and stacking the two bases above the two phenyl ring of the strand) is reminiscent of the crystallographically observed stacking in the heart of the 3WJ.

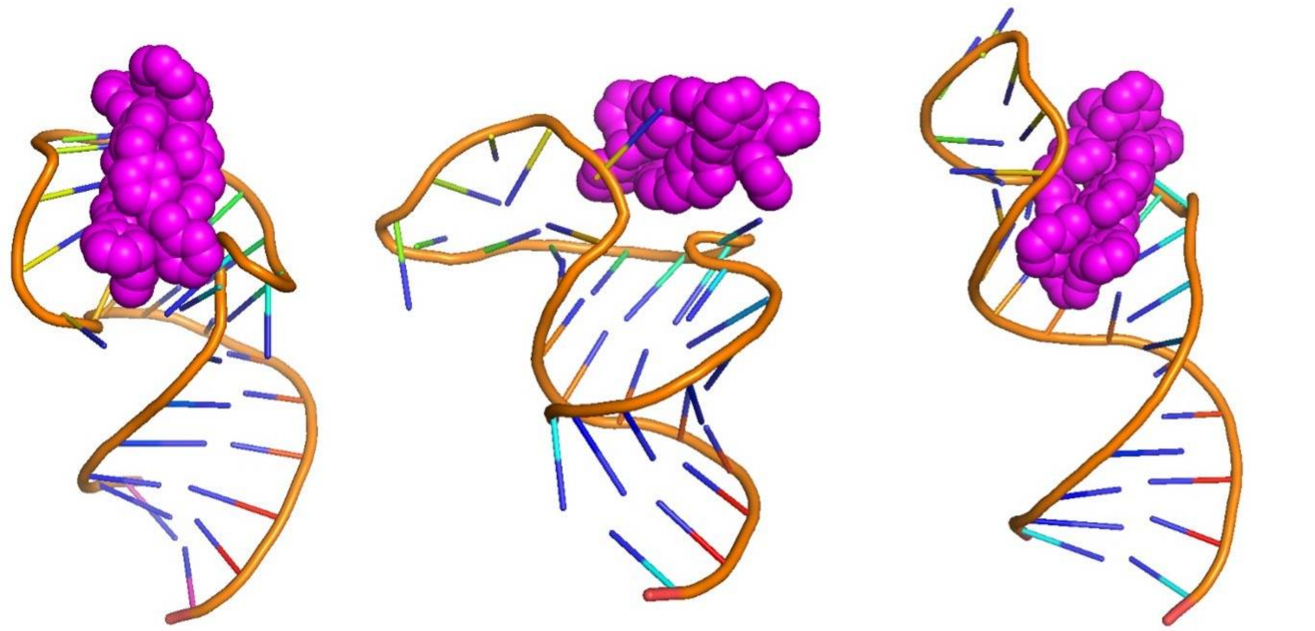

Fig. S9 Exemplar bulge-binding modes observed in the simulations, including bridging from bulge to loop. The right hand figure has the cylinder in the position where the a cyclic peptide has been observed to bind to TAR.

5e. Insertion into bulge This is discussed in detail in the main text and can be observed in the accompanying SI videos.

5f Binding of the ruthenium cylinder. We also repeated simulations for the ruthenium cylinder (total 17.3 ms) in place of the iron cylinder. The ruthenium cylinder behaved analogously in its binding, though its movement was slower due to the increased molecular mass.

Part B: Approach used to analyse the simulation results

To analyse the vast volume of data, over 200,000,000 coordinate frames we employ the PyEmma workflow)[13] and Markov State Modelling (MSM)) (details in methods). This involves reducing the dimensionality by choosing appropriate feature of the simulation and identify macrostates of each simulations using MSM and extract those metastable structures with Perron-cluster cluster analysis (PCCA). Those extracted structures and the whole simulation are also presented in the Leontis-Westholf (LW) nomenclature using barnaba)

In detail;

The whole compressed trajectory is loaded into PyEMMA with 10ps time steps, without concatenating the independent simulations of the same system.

1. The dimensionality of the simulation is reduced by feature selection and using Principal Component Analysis (PCA)(Figure S12 and similar) . This step aims to capture as much of the kinetic variance of the simulation as possible in fewer dimensions. PCA gives a broad picture of the overall kinetics of the simulations, highlighting minima in a throughout-the-simulation manner. The distribution of the principal component eigen values reveals that significant variance in the data is described by higher order features beyond the traditional first two components(example of Figure S36) and we therefore choose to plot the projections onto the pairwise combinations of the first four components.
2. To extent the representation into the time domain we use Time-lagged Independent Component Analysis to examine the similarity between time points with a specified offset (lag time)(Figure example S18 S19). This was used to select a lag time that produced the fewest number of independent components that incorporated 95% of the overall kinetic variance. For visualisation purposes, we plot the projections onto the pairwise combinations of the first four components (Figure S13).
3. The data are projected onto the first 2 ICs for the selected lag time (number of steps usually between 200 and 500), and are then clustered using k-means (Figure S16). The number of clusters is chosen as the starting point of the plateau in the VAMP2 graph(Figure S17). This allows the problem to be mapped onto a set of discrete states (clusters) which then allows Markov State Models (MSMs) to be constructed that describe transitions between pairs of clusters. MSM calculates every pairwise transition at a given lag-time for the MSM (which independent of the TICA lag time). It is essential that the selected lag time is long enough to ensure Markovian dynamics (ie. to have a stable transition matrix), but short enough to resolve the transition dynamics. To achieve this the smallest possible lag-time (in the range 1 to 1200 steps) that shows convergence of the underlying processes is chosen (Figure S16). We validate the model with the Chapman-Kolmogorov test (Figure 15), ensuring that the model also describes longer time-scales. If the resulted model includes all states, the original space can then be split into the longest living meta-stable states with care taken to ensure sure that those states are well defined on the discretised surface(Figure S14).For each state a sample of 10 pdb structures is extracted and represented in Leontis-Westholf notation using barnaba (Figure S14). Finally spectral clustering using PCCA++ algorithm coarse-grains the space of the metastable distributions and approximates the stationary probabilities and relative free energies for that particular set of data (Table 1) and not for the overall system.

The following Supplementary Figures illustrate the results of this workflow for each of the simulations in turn:

Figures	
S10-S32	Coxsackie-virus stem-loop free
S33-S34	Coxsackie-virus stem-loop P enantiopmer
S35-S40	Coxsackie-virus stem-loop free 500mM NaCl
S41-S46	Poliovirus stem loop free
S47-S55	Poliovirus stem loop P and M cylinders
S56-S62	HIV-2 TAR bulge free
S63-S125	HIV-1 TAR bulge free
S126-S155	HIV-1 TAR bulge M cylinder
S156-S182	HIV-1 TAR bulge P cylinder
Tables	
Tables 3-8	Relative energy estimate of each state for given simulation

Table 1

	LW	PCA	TICA	TICA vs time	macrostates	TICA - time lags
1ANR 1						
10µs	S63	S64	S65	S66	S67	S68
6µs	S69	S70	S71	S72	S73	S74
1ANR 3	S75	S76	S77	S78	S79	S80
1ANR 4	S81	S82	S83	S84	S85	S86
1ANR 7	S87	S88	S89	S90	S91	S92
1ANR 10	S93	S94	S95	S96	S97	S98
1ANR 12	S99	S100	S101	S102	S103	S104
1ANR 16	S105	S106	S107	S108	S109	S110
1UUI	S111	S112	S113	S114	S115	S116
Summary 1ANR	S117	S118	S119	S120	S121-122	S123-S125

Table 2

Coxsackievirus stem-loop

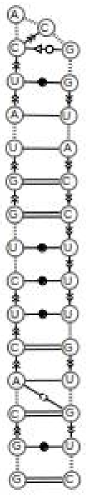

Figure S10 LW representation of the 20 NMR solutions of pdb ; 1RFR

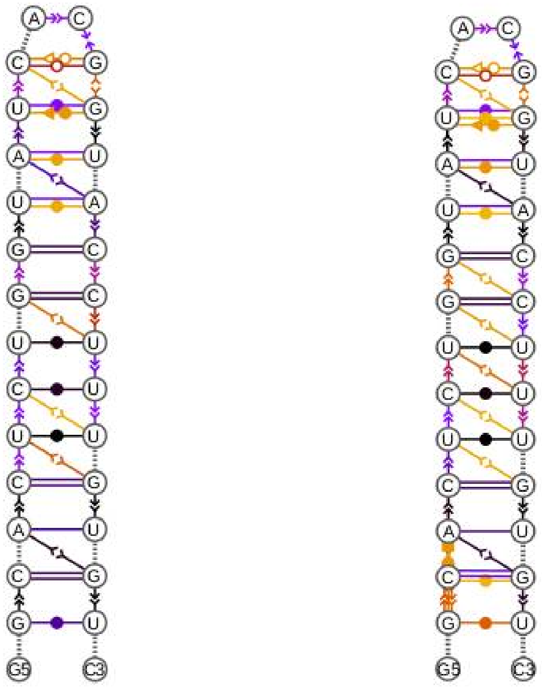

Figure S11 LW representation of first (left) and second (right) run of stem loop.

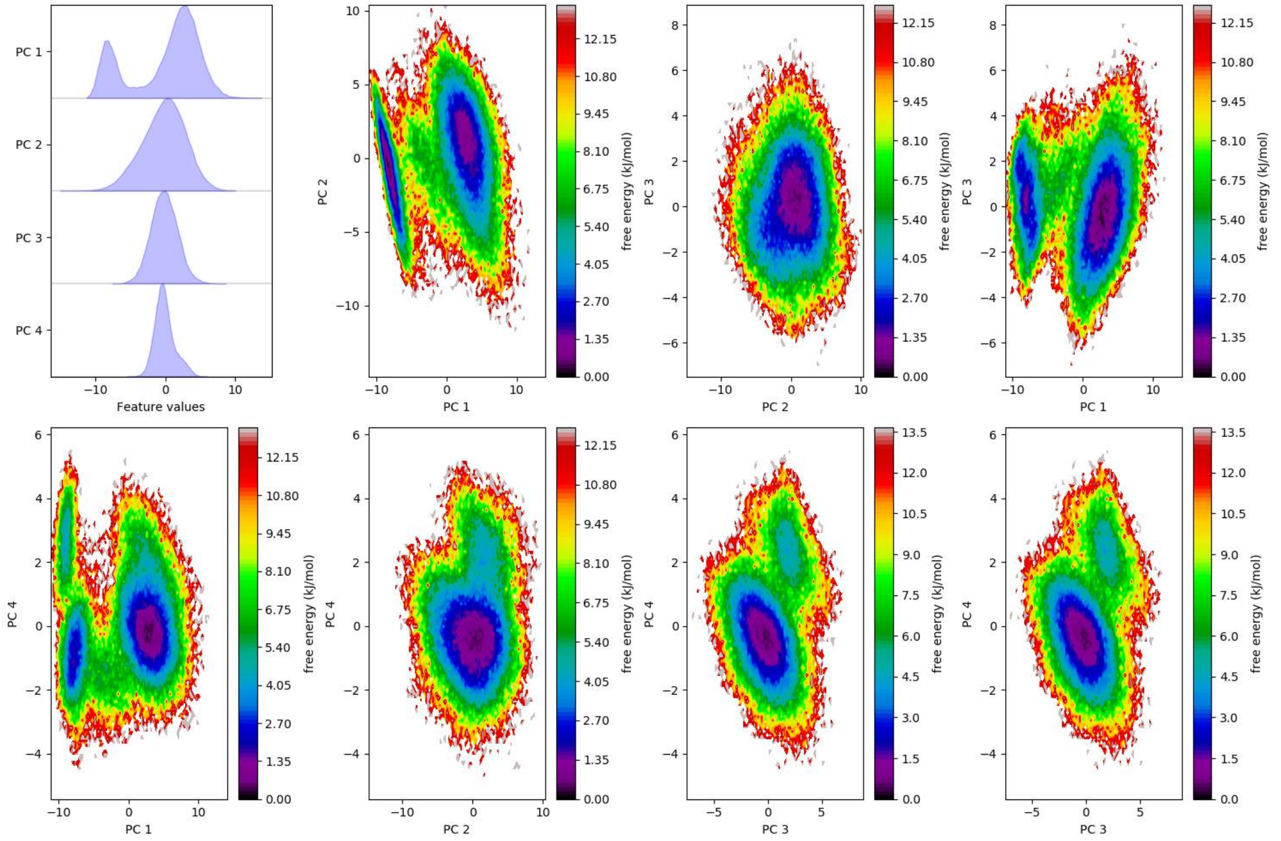

Figure S12 PCA projections between 1-4 of combined simulation of Coxsackievirus stem-loop

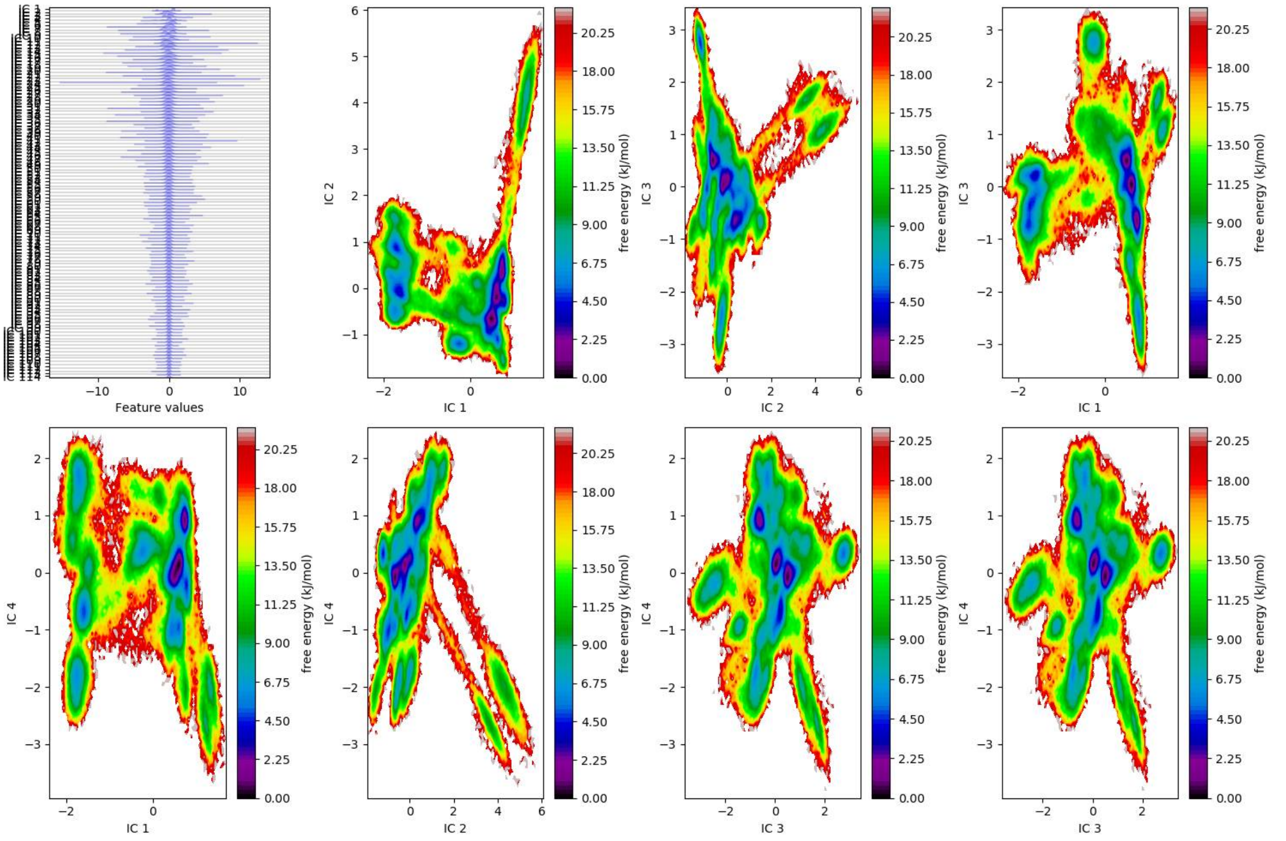

Figure S13 TICA projections between 1-4 of combined simulation of Coxsackievirus stem-loop

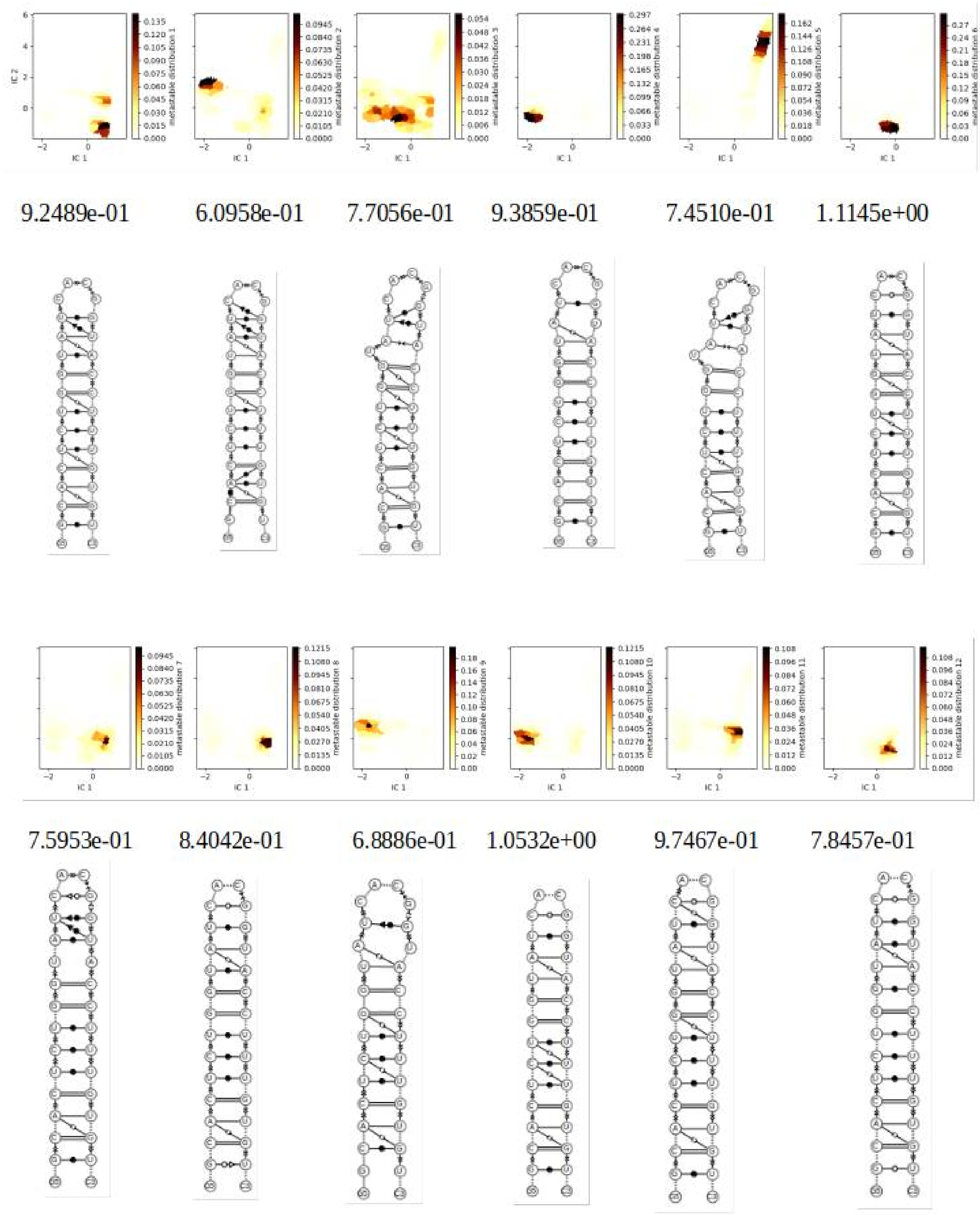

Figure S14 Regions of the TICA landscape and corresponding structure for combined simulation of Coxsackievirus stem-loop

state	π	G/kT
1	0.017950	4.020147
2	0.015814	4.146865
3	0.023508	3.750394
4	0.028997	3.540548
5	0.035619	3.334886
6	0.037003	3.296763
7	0.096863	2.334453
8	0.142168	1.950743
9	0.082000	2.501040
10	0.119014	2.128512
11	0.161520	1.823125
12	0.239542	1.429025

Table 3 Relative energy estimate of each state of combined simulation of Coxsackievirus stem-loop

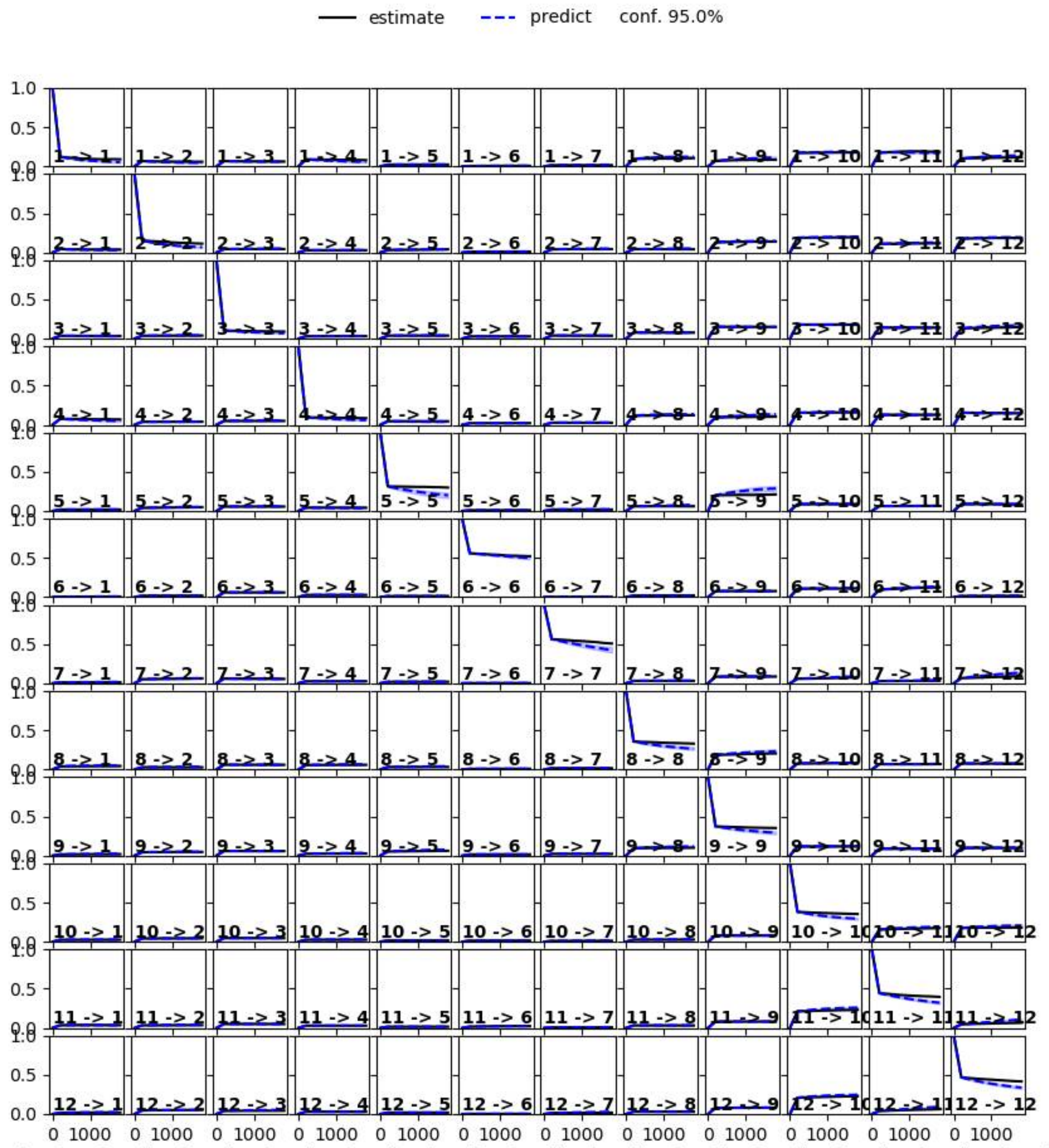

Figure S15 CK-test for 12 states

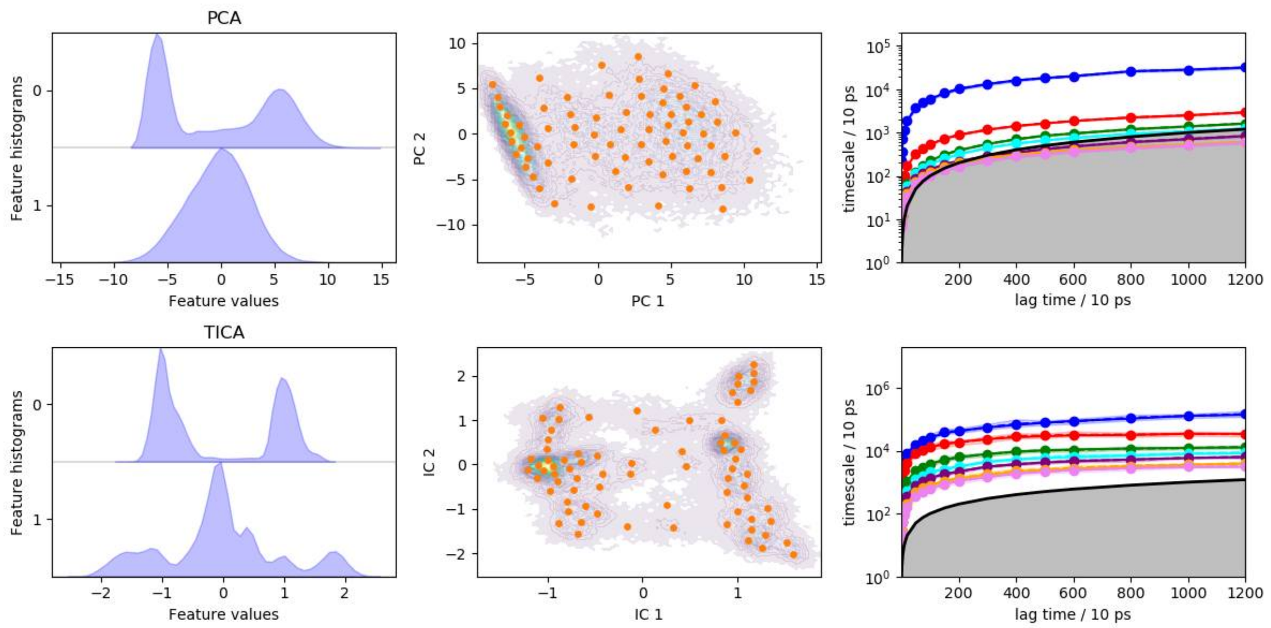

Figure S16 PCA TICA and model quality for 1 to 1200 steps of 10ps

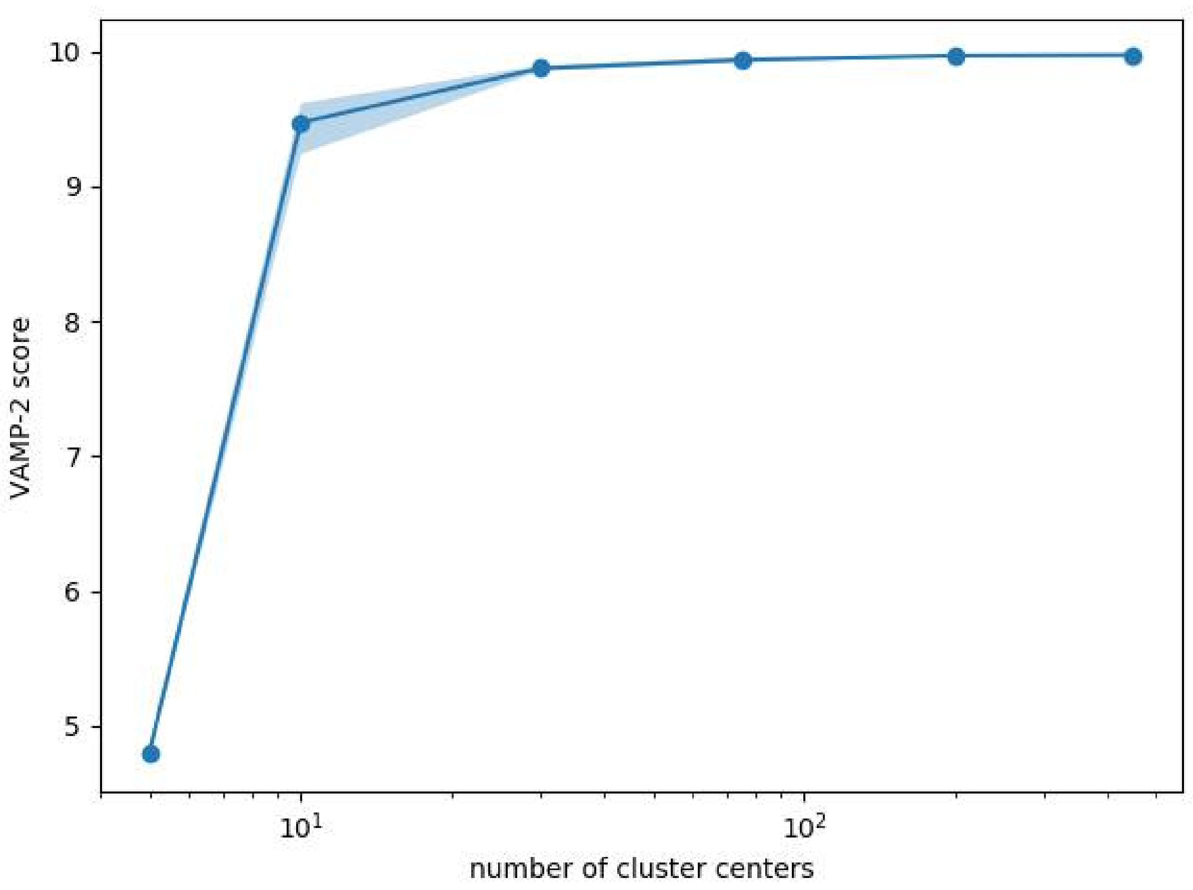

Figure S17 ; VAMP-2 score with respect to number of cluster centres on the TICA surface

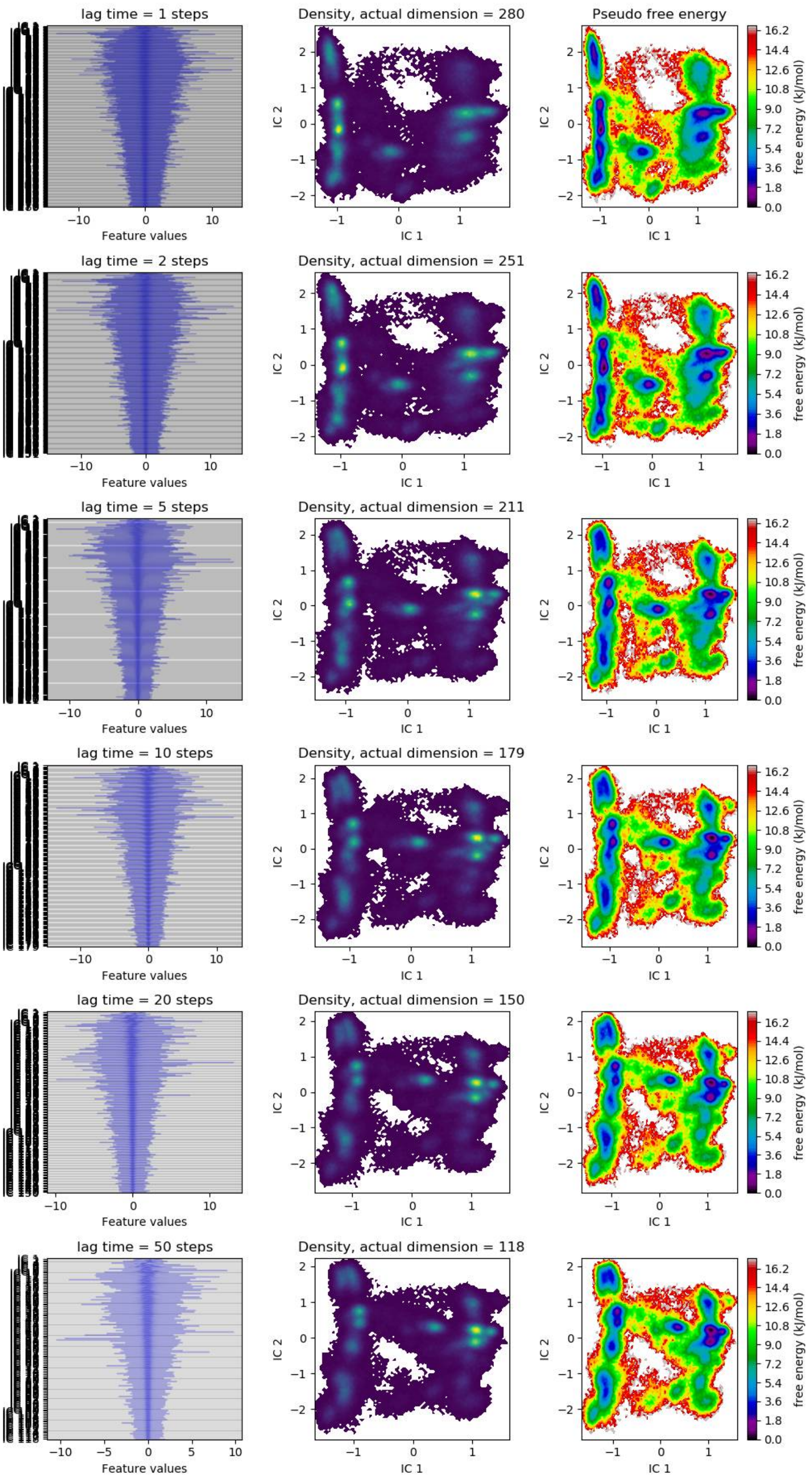

#

Figure S18 TICA dimensions, density and corrected heat map for steps 1 to 50 of combined simulation of Coxsackievirus stem-loop

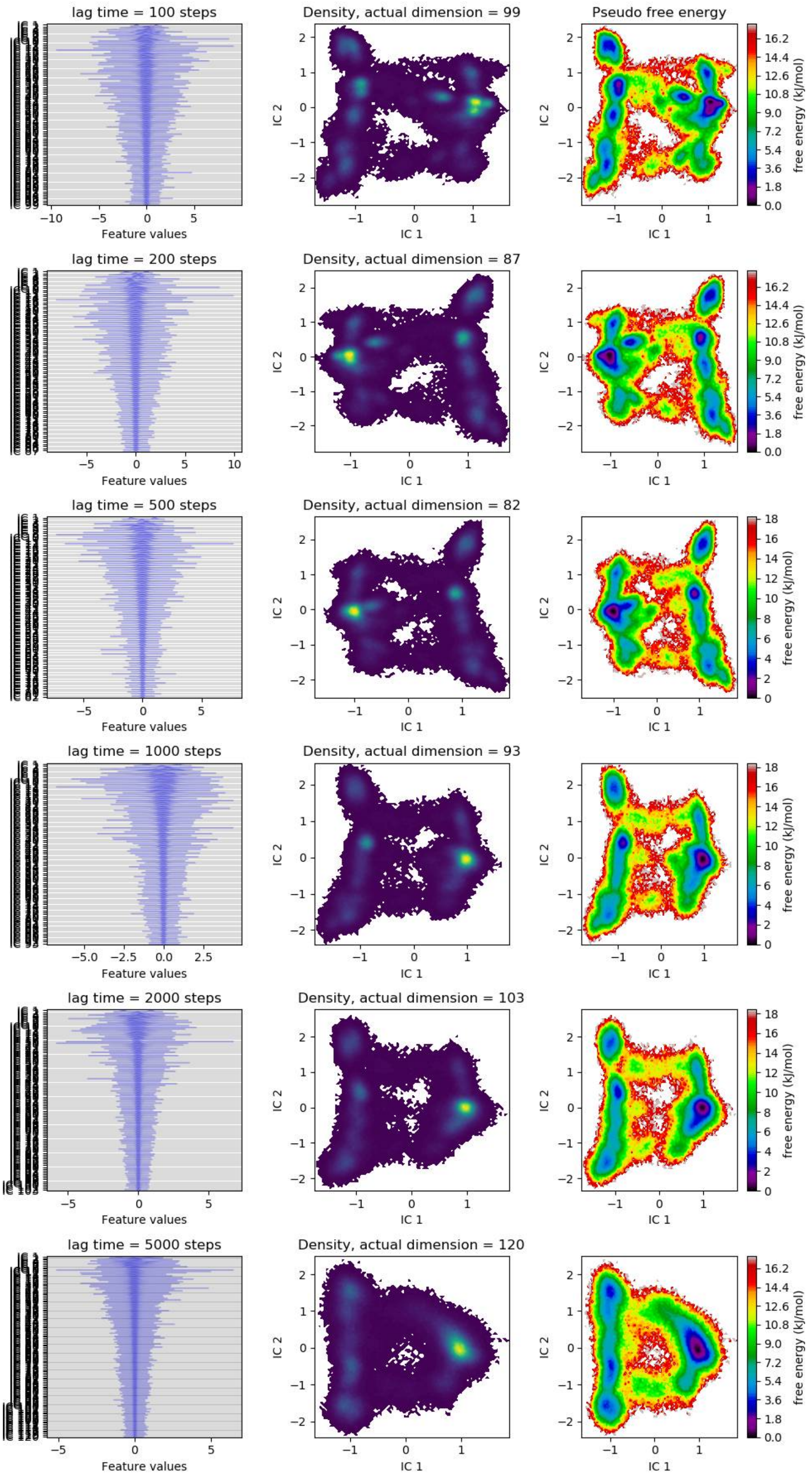

Figure S19 TICA dimensions, density and corrected heat map for steps 100 to 5000 of combined simulation of Coxsackievirus stem-loop

Individual runs of coxsackievirus stem-loop
Run 1

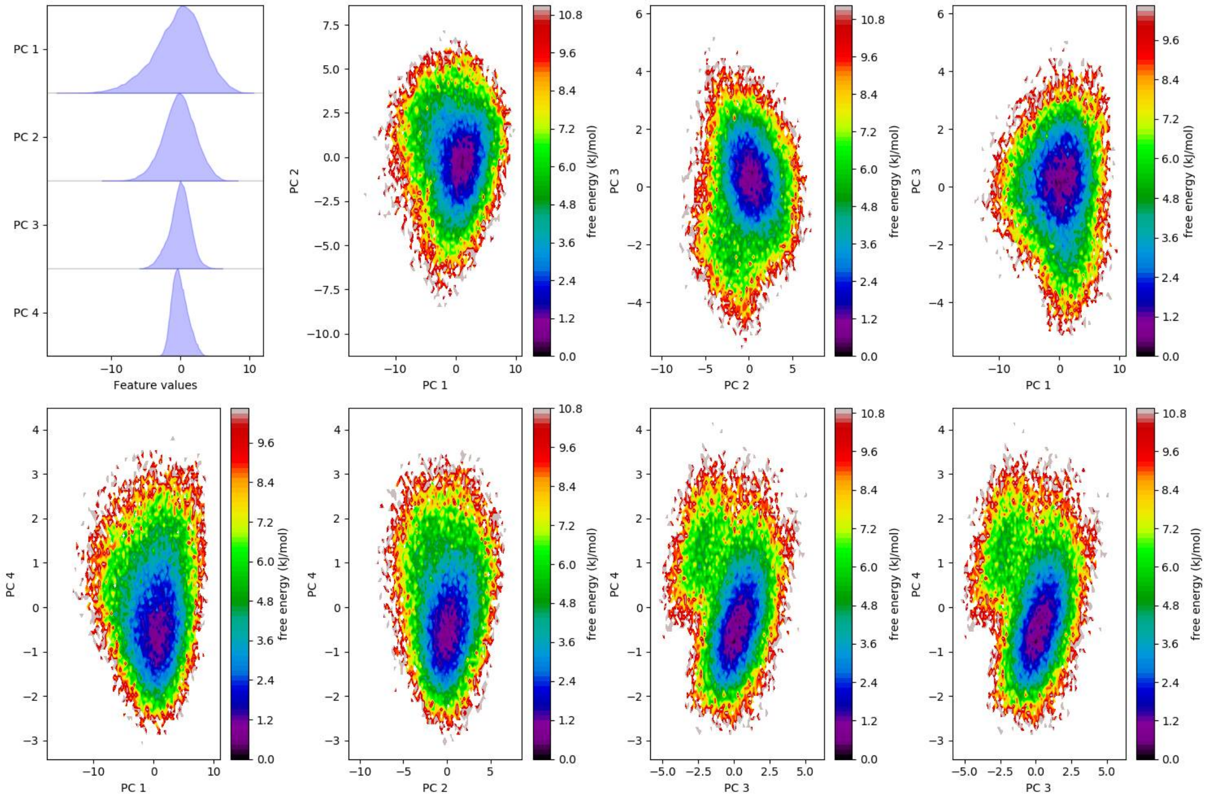

Figure S20 PCA projections between 1-4 of run 1

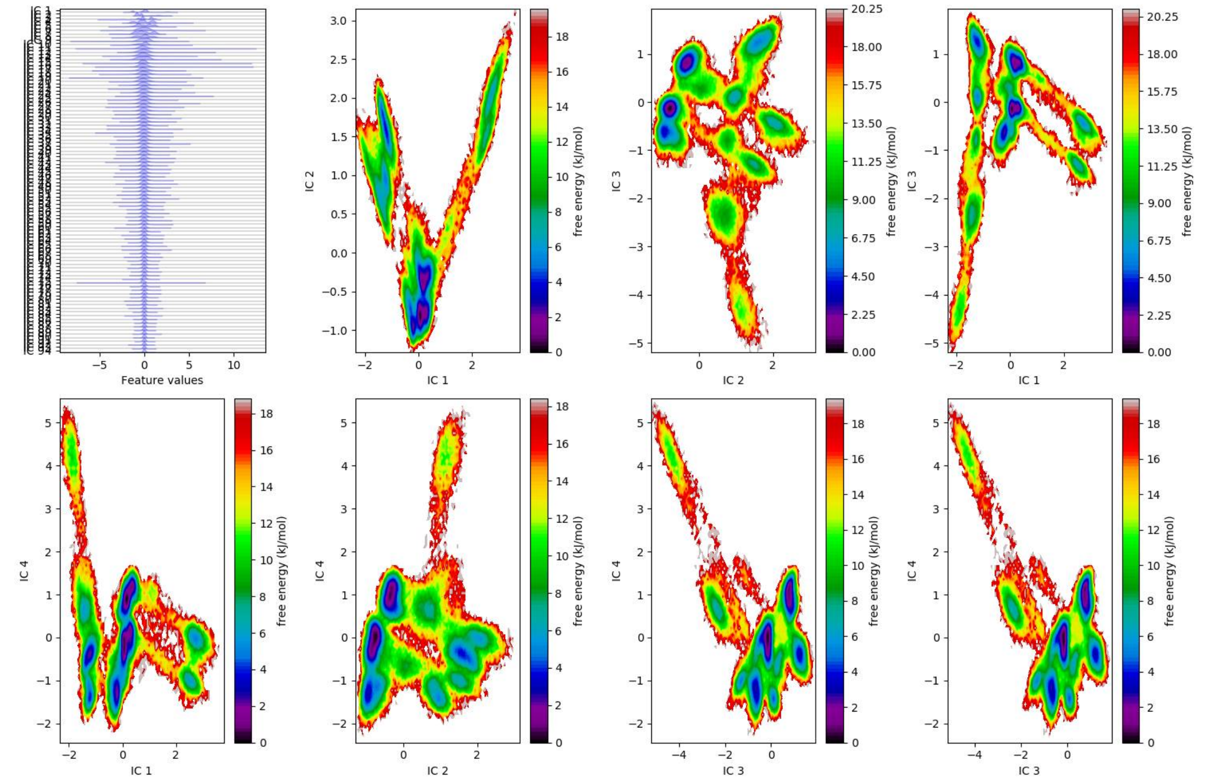

Figure S21 TICA projections between 1-4 of run 1

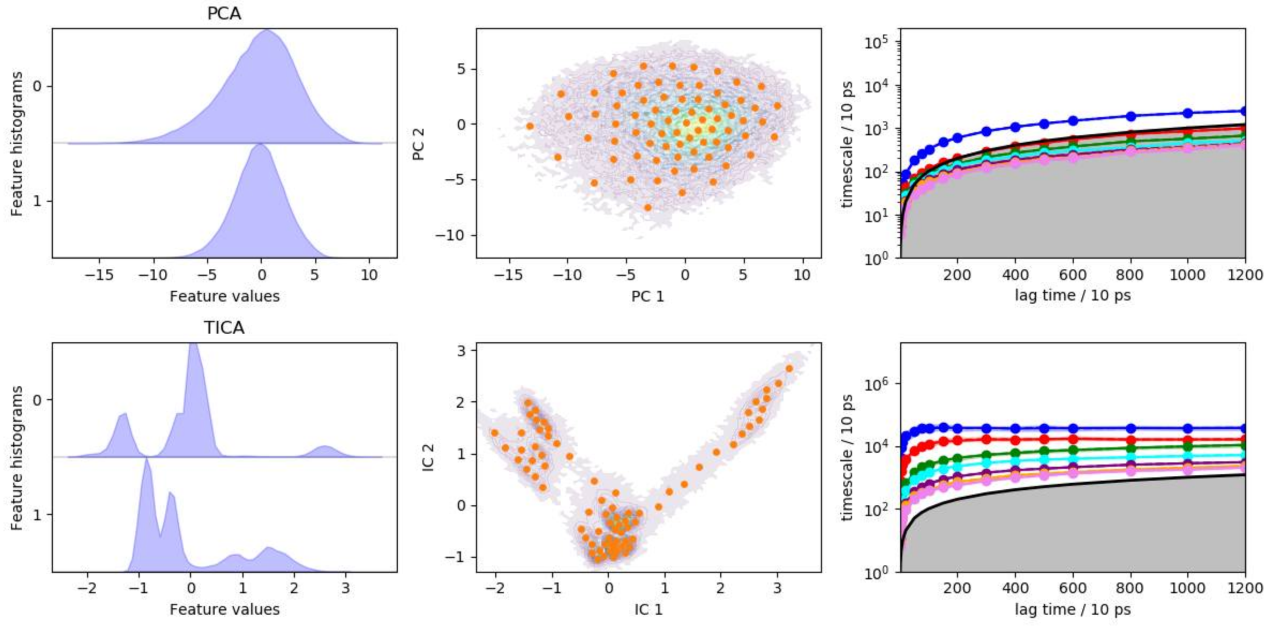

Figure S22 PCA TICA and model quality for 1 to 1200 steps of 10ps of run 1

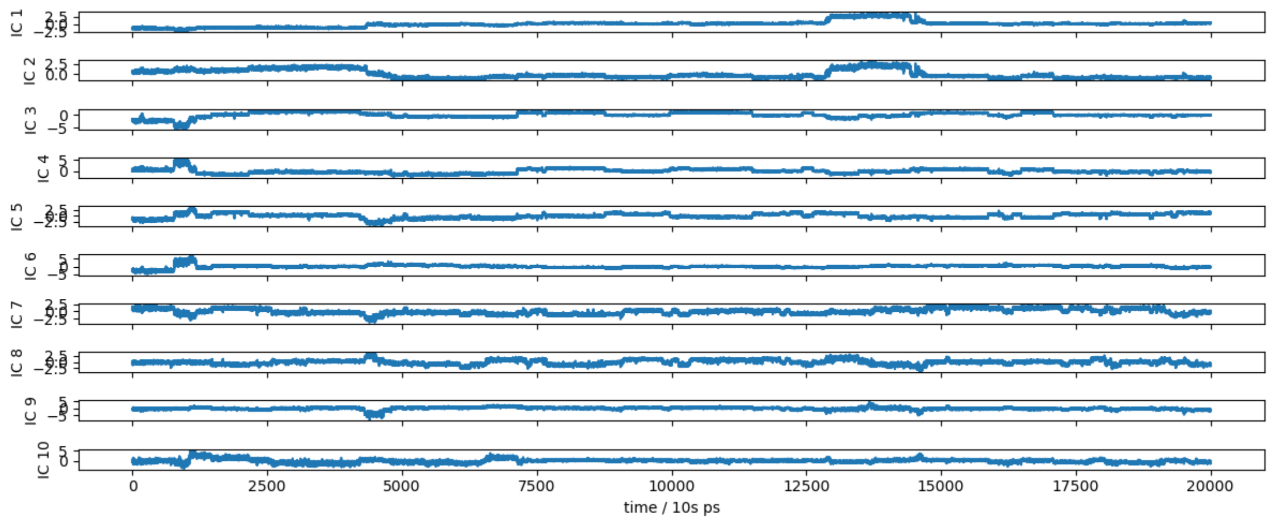

Figure S23 First 10 IC values over the simulation time of run 1

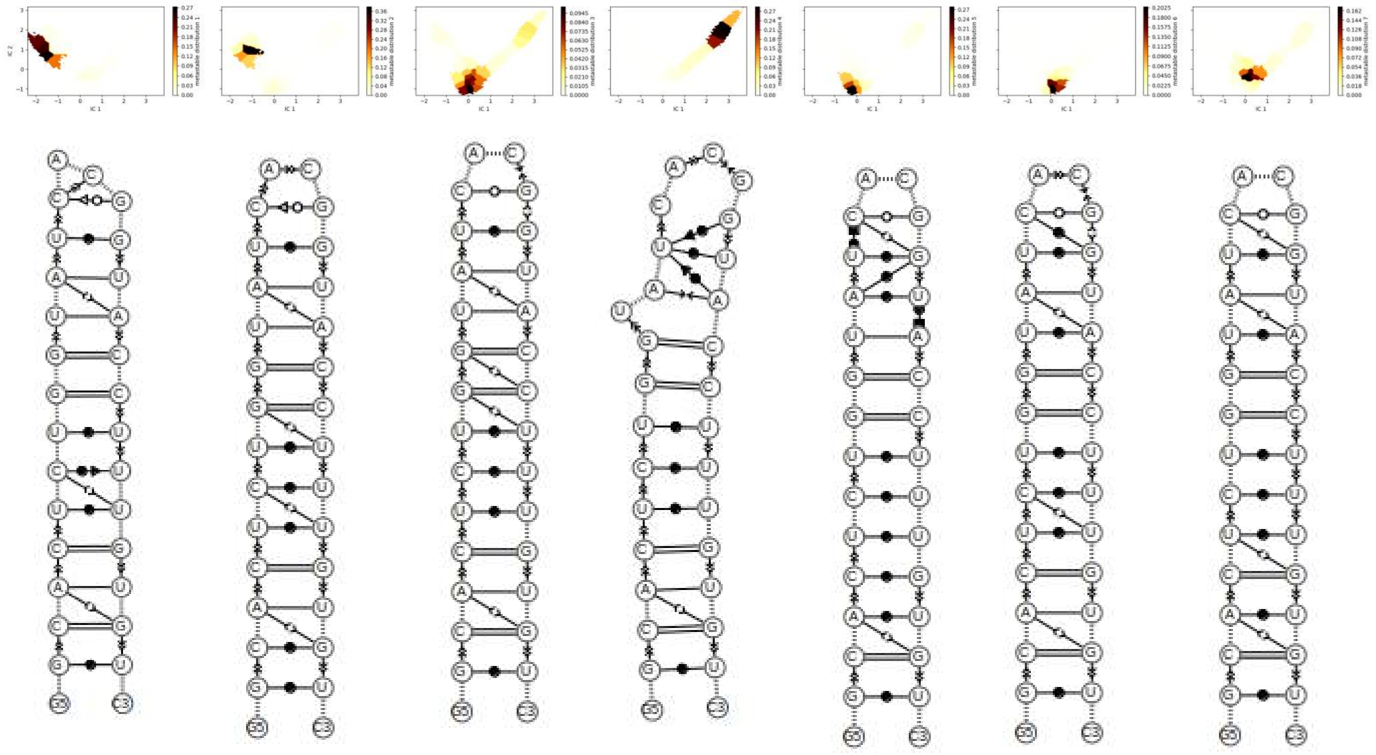

S24 Regions of the TICA landscape and corresponding structure for run 1

state	π	G/kT
1	0.000423	7.767057
2	0.001393	6.576271
3	0.002380	6.040546
4	0.097398	2.328949
5	0.152402	1.881235
6	0.381115	0.964655
7	0.364889	1.008163

Table 4 Relative energy estimate of each state of run 1

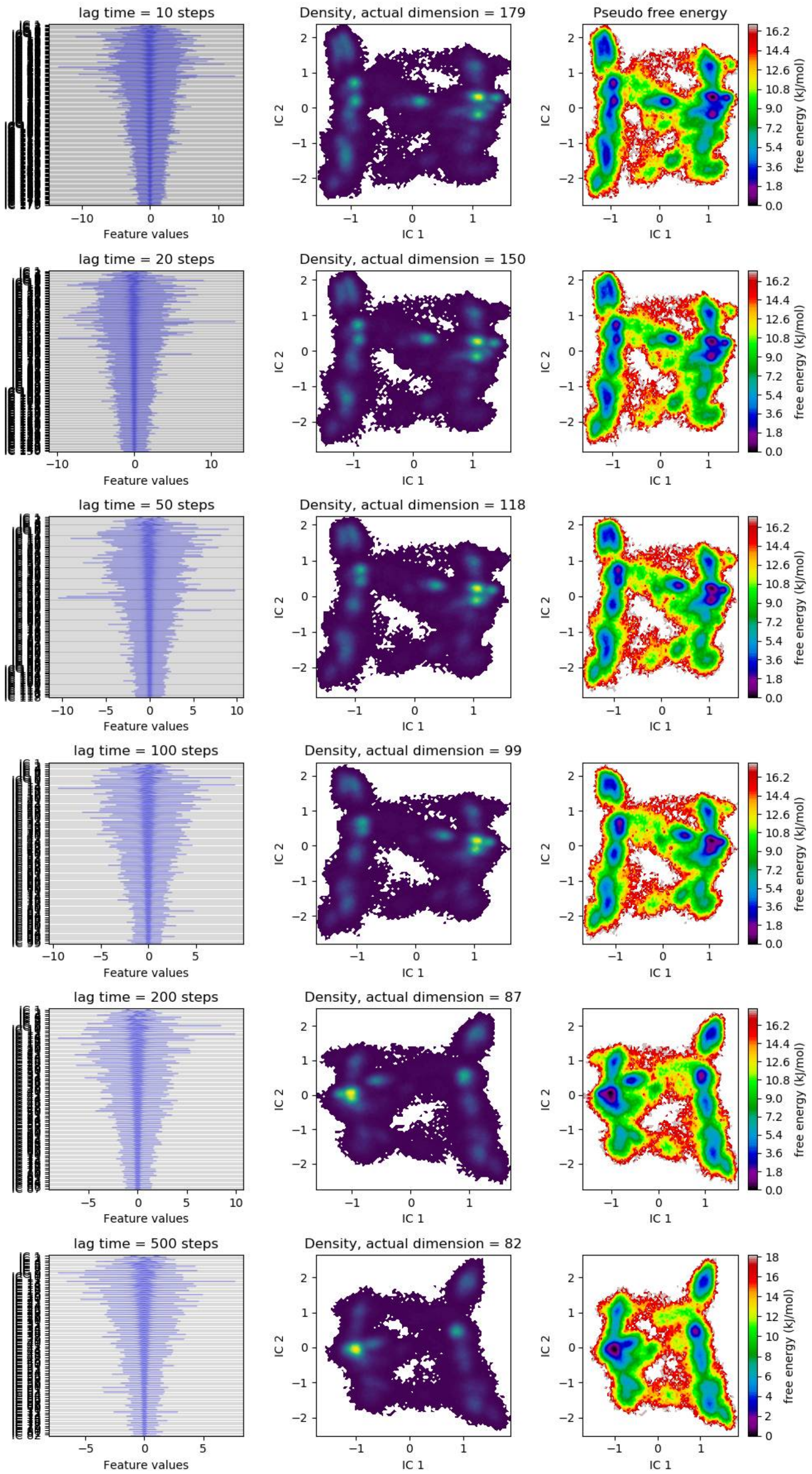

Figure S25 TICA dimensions, density and corrected heat map for steps 10 to 500 of run 1

Coxsackievirus stem-loop;l Run 2

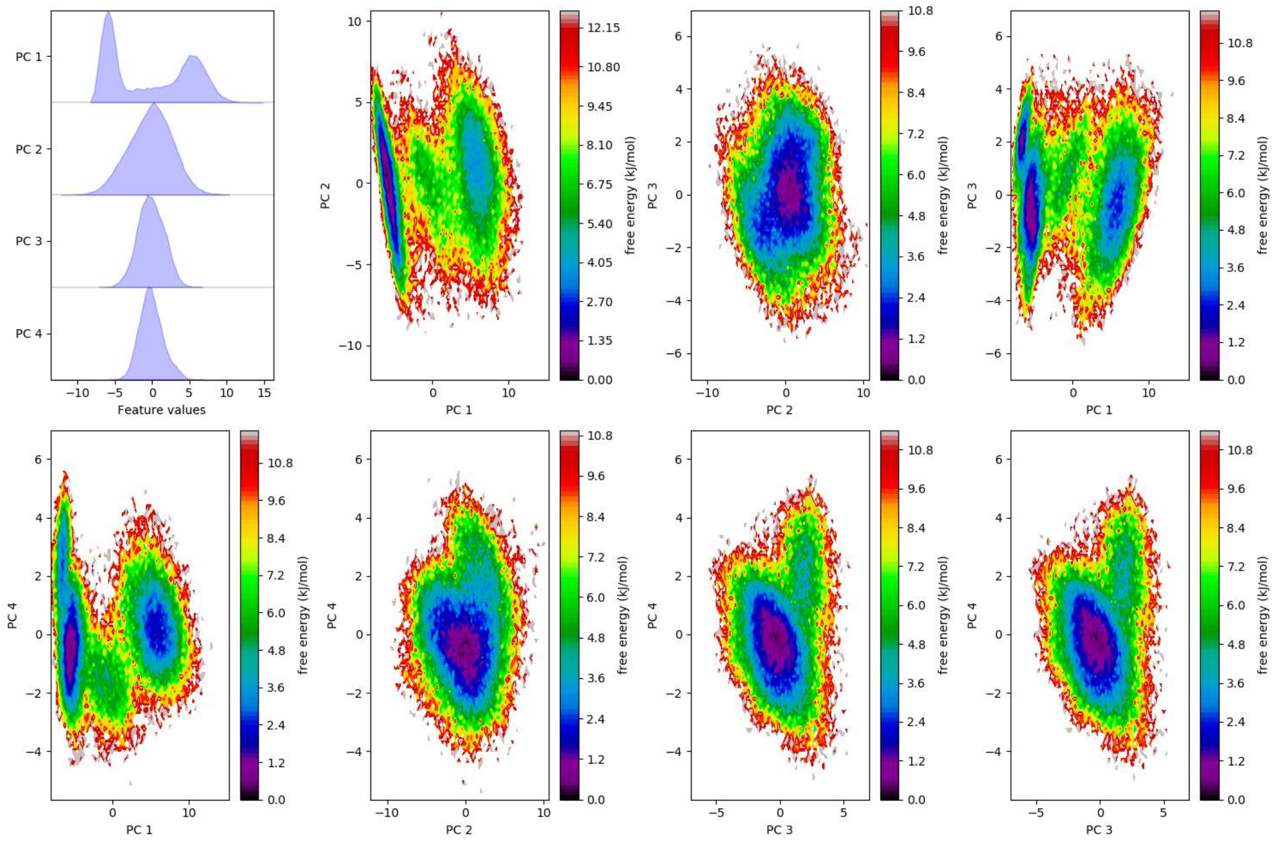

Figure S26 PCA projections between 1-4 of run 2

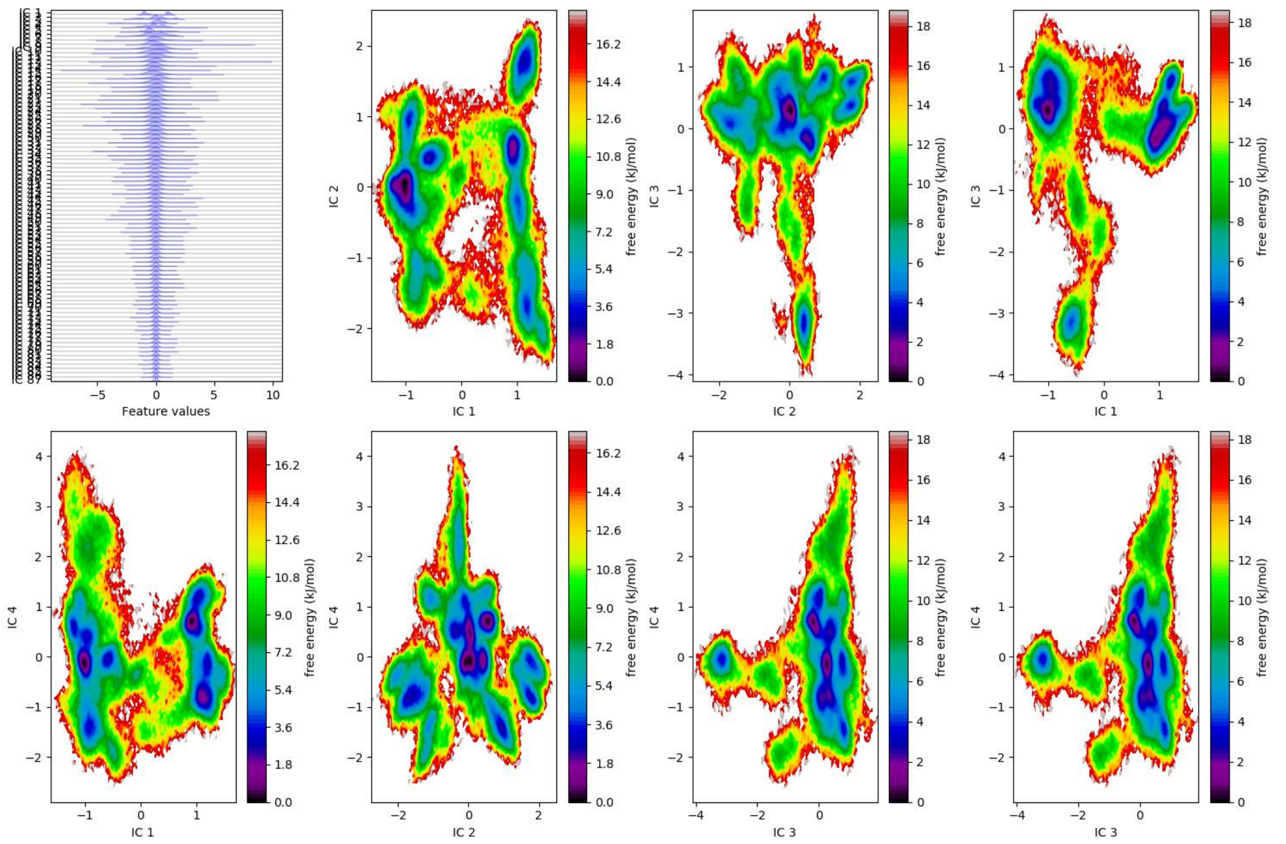

Figure S27 TICA projections between 1-4 of run 2

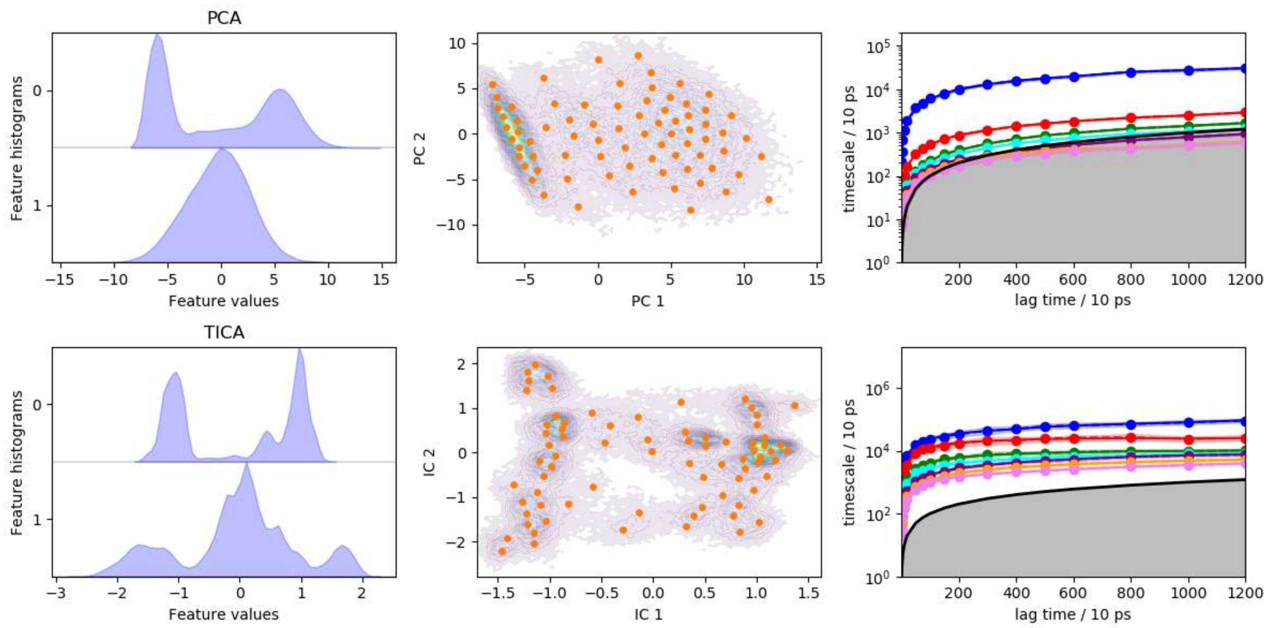

Figure S28 PCA TICA and model quality for 1 to 1200 steps of 10ps of run 2

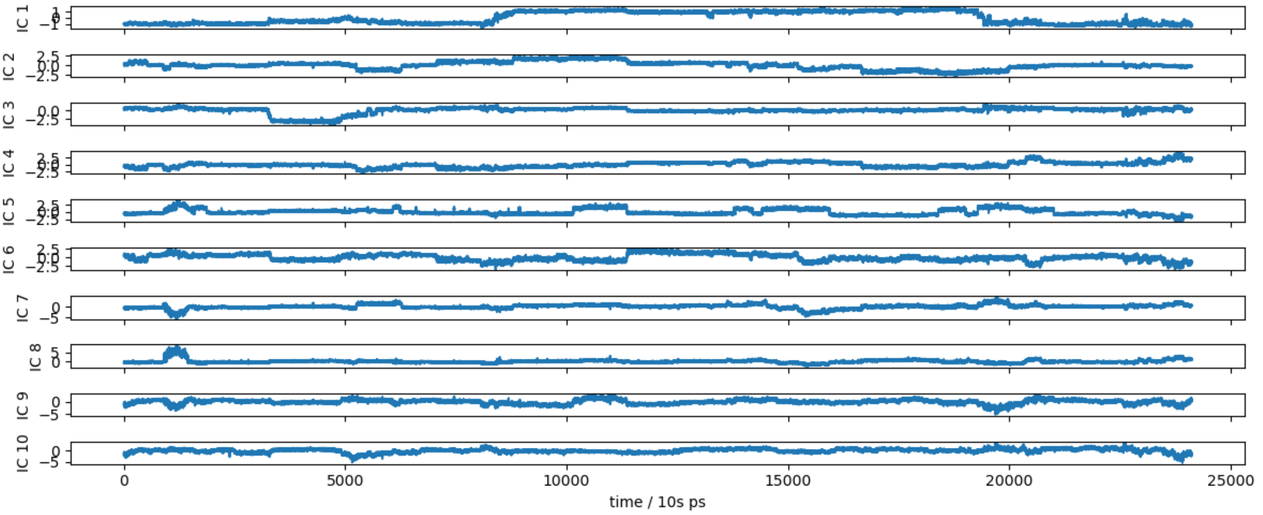

Figure S29 First 10 IC values over the simulation time of run 2

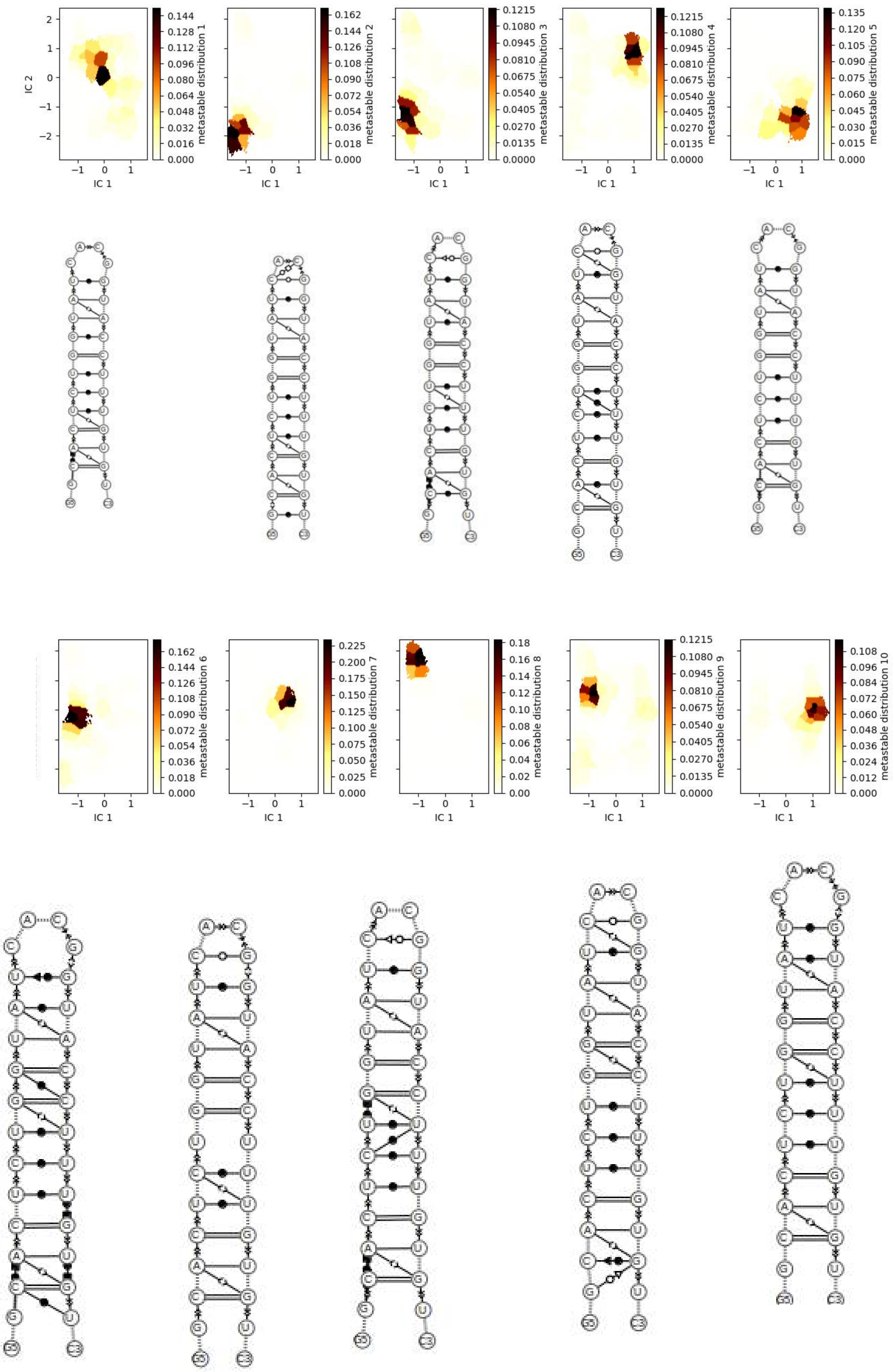

Figure S30 Regions of the TICA landscape and corresponding structure of run 2

state	π	G/kT
1	0.006777	4.994165
2	0.026901	3.615606
3	0.106978	2.235136
4	0.070643	2.650114
5	0.076508	2.570357
6	0.071913	2.632301
7	0.077083	2.562879
8	0.107365	2.231518
9	0.133113	2.016556
10	0.322719	1.130972

Table 5 Relative energy estimate of each state of run 2

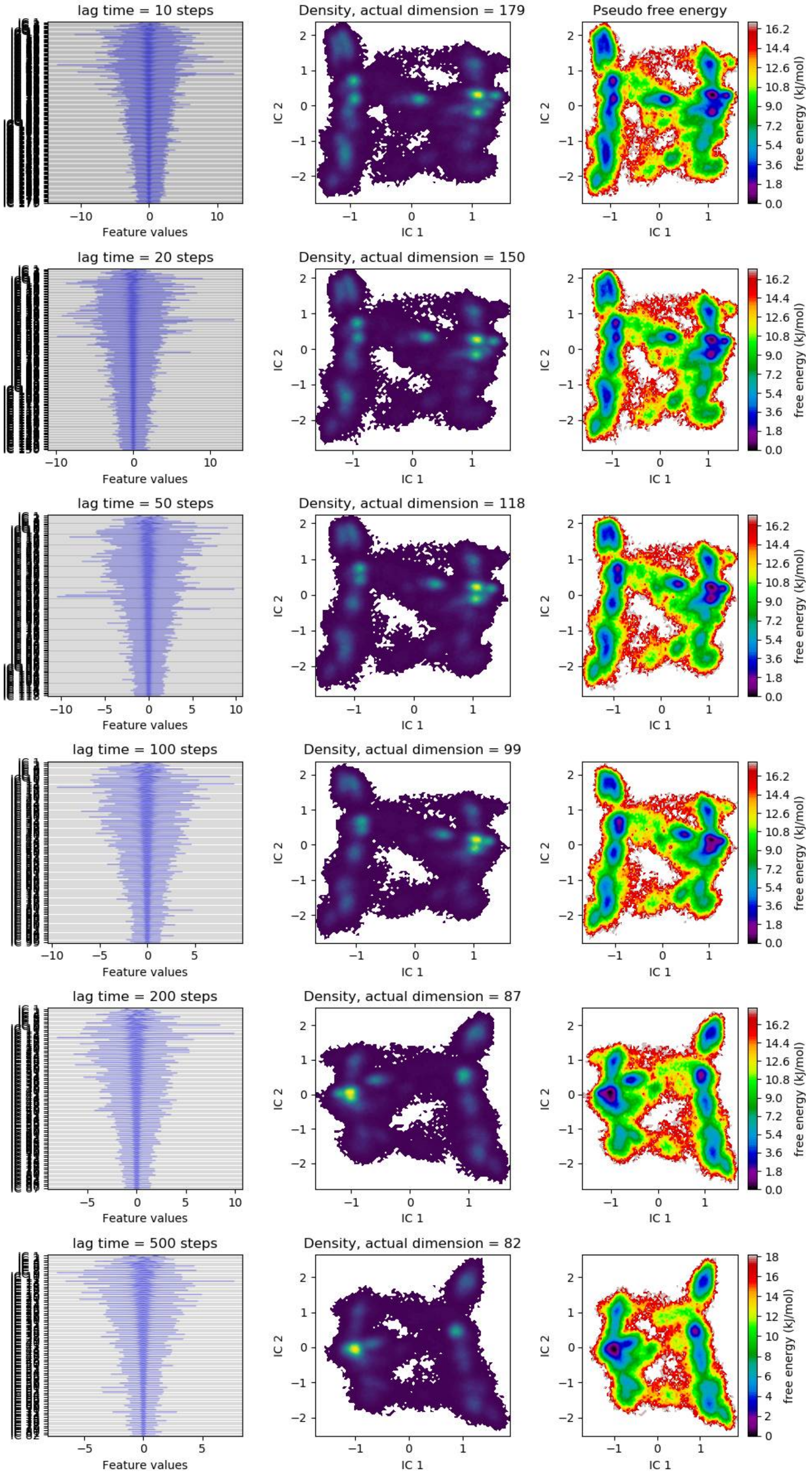

Figure S31 TICA dimensions, density and corrected heat map for steps 10 to 500 of run 2

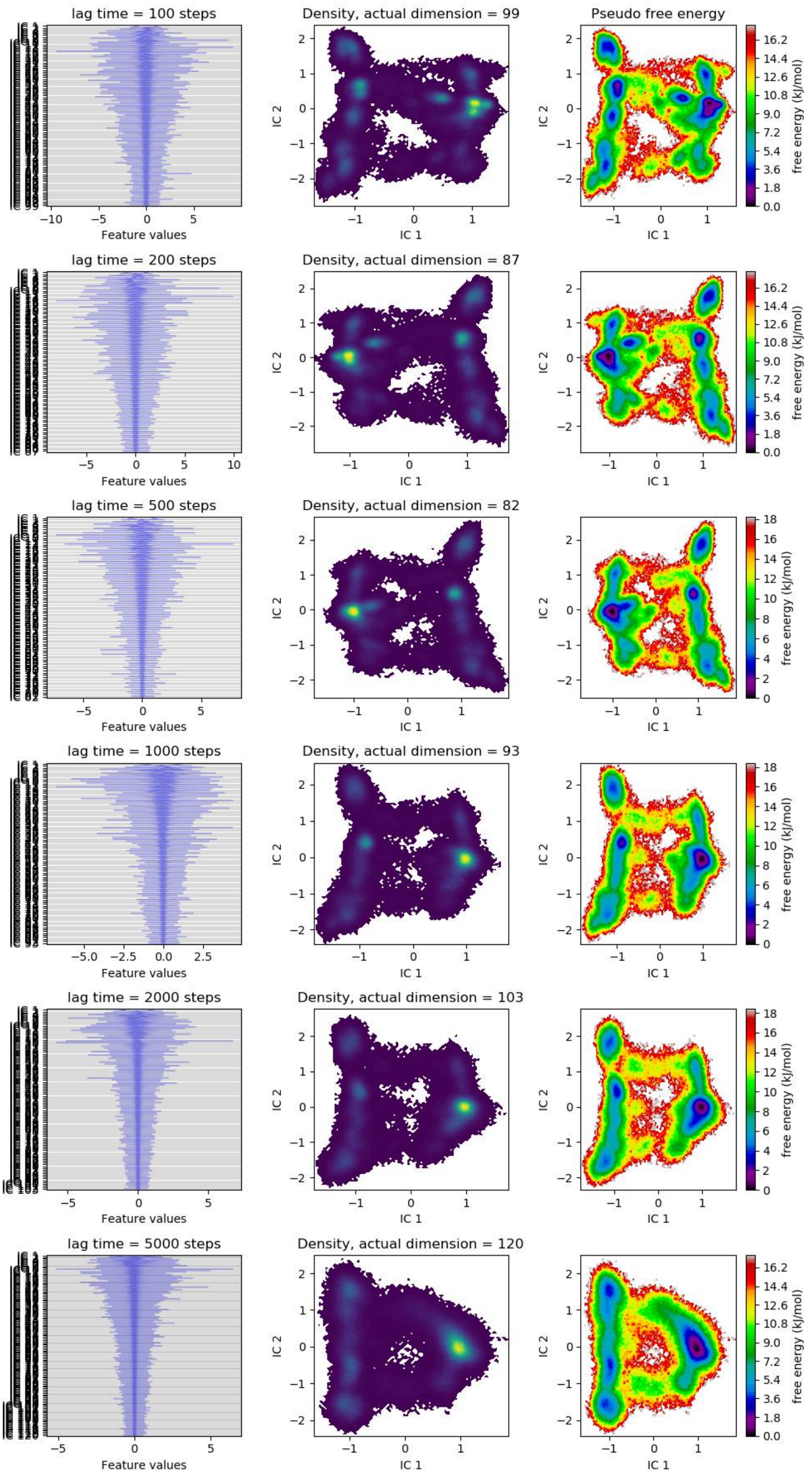

Figure S32 TICA dimensions, density and corrected heat map for steps 100 to 5000 of run 2

Coxsackievirus with cylinders

with P enantiomer- analysis of RNA only

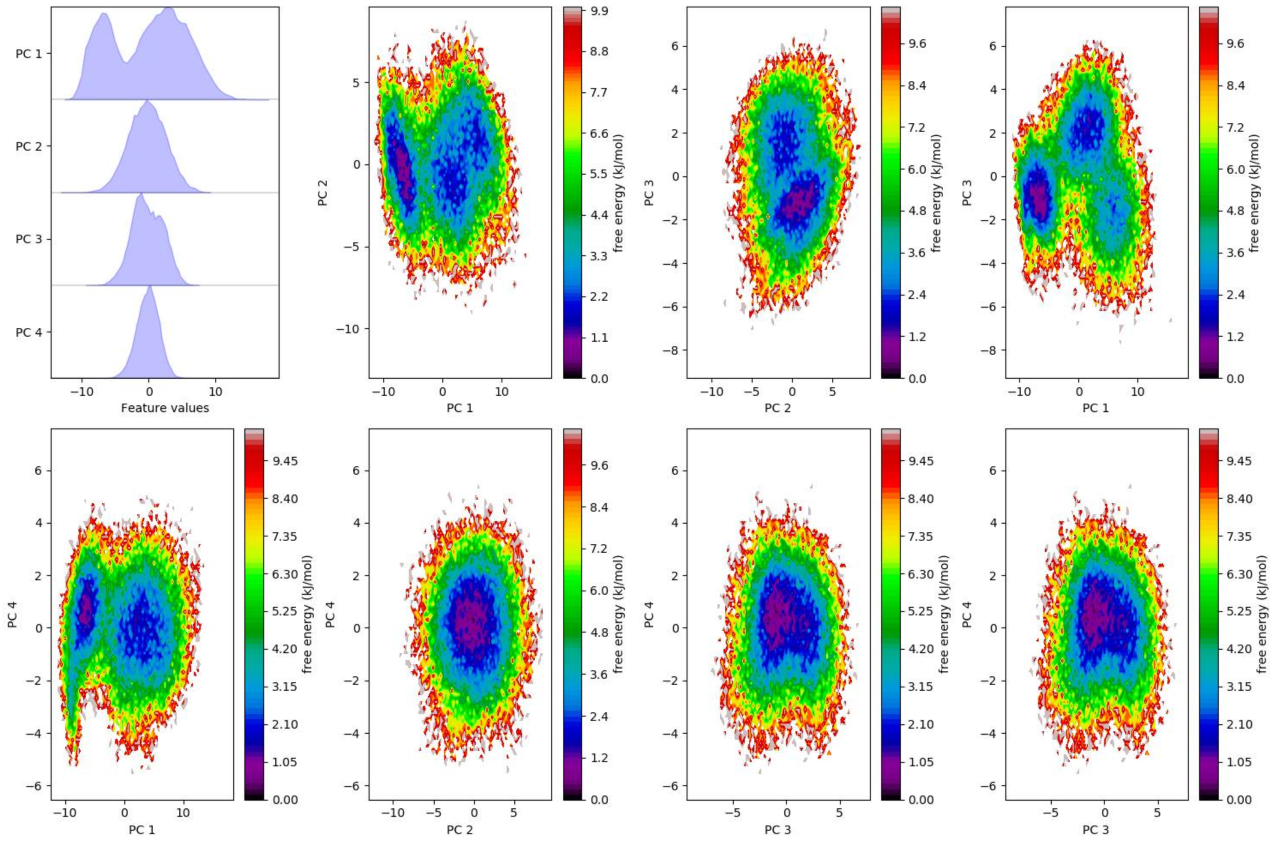

Figure S33 PCA projections between 1-4 of RNA conformations of coxsaackievirus stem loop in the presence of cylinder

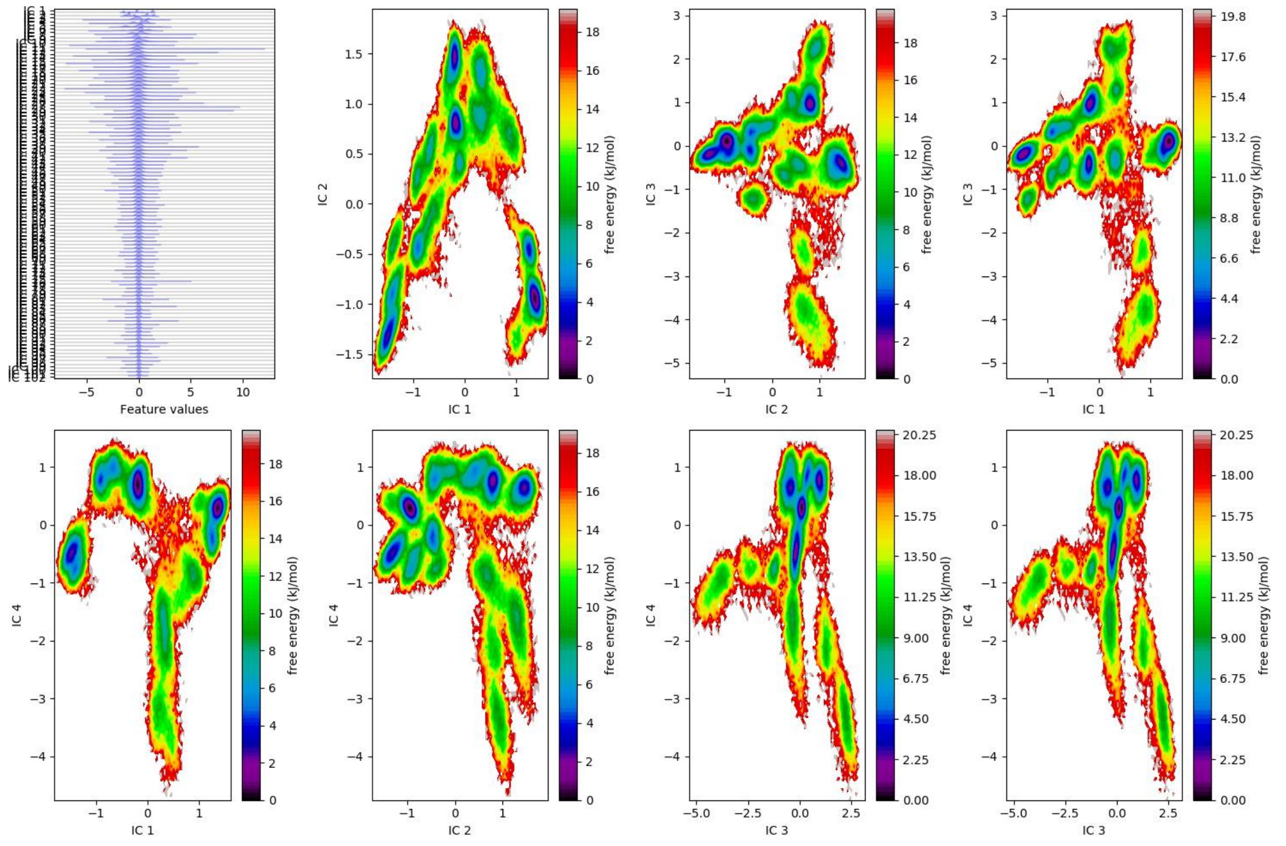

Figure S34 TICA dimensions, density and corrected heat map for 200 steps

Coxackievirus High 500mM

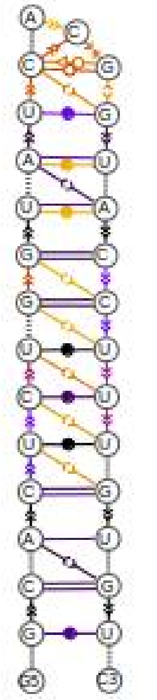

Figuire S35 LW representation of the coxsackievirus Stem-loop in 500mM NaCl concentration

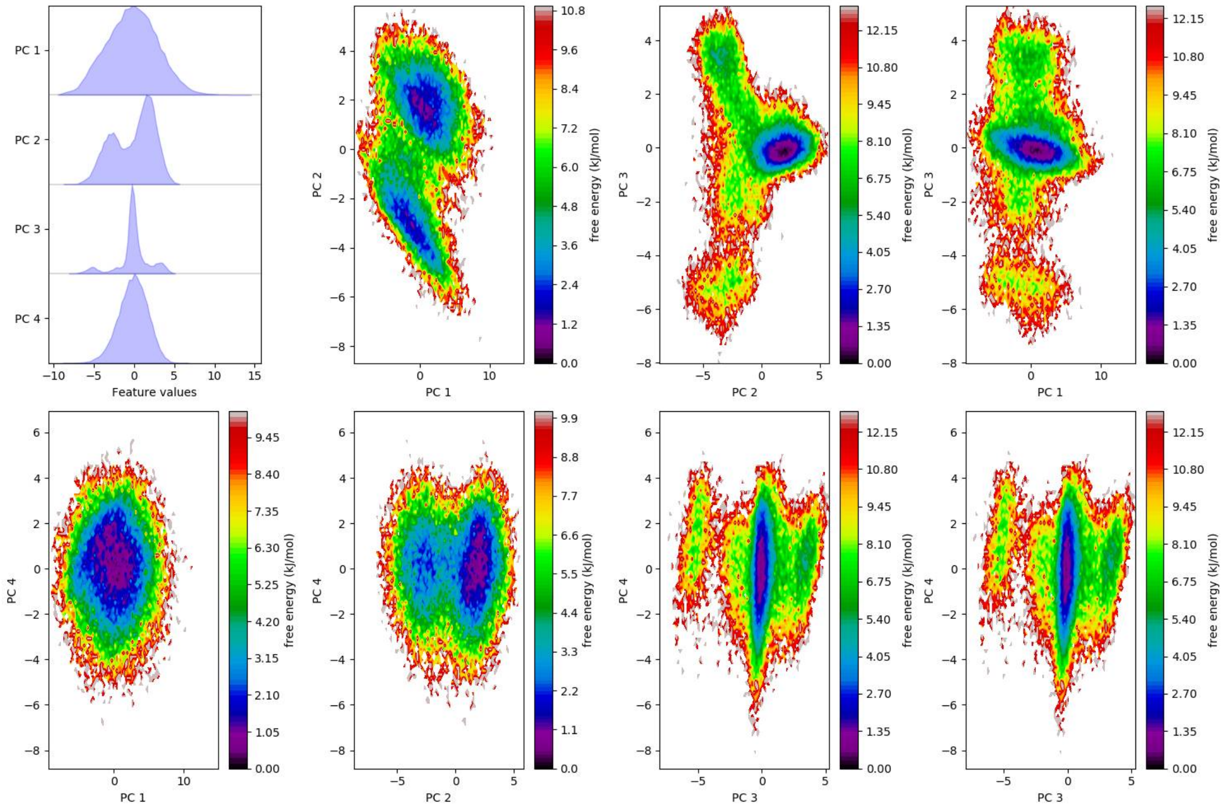

Figuire S36 PCA projections between 1-4 Stem-loop in 500mM NaCl concentration

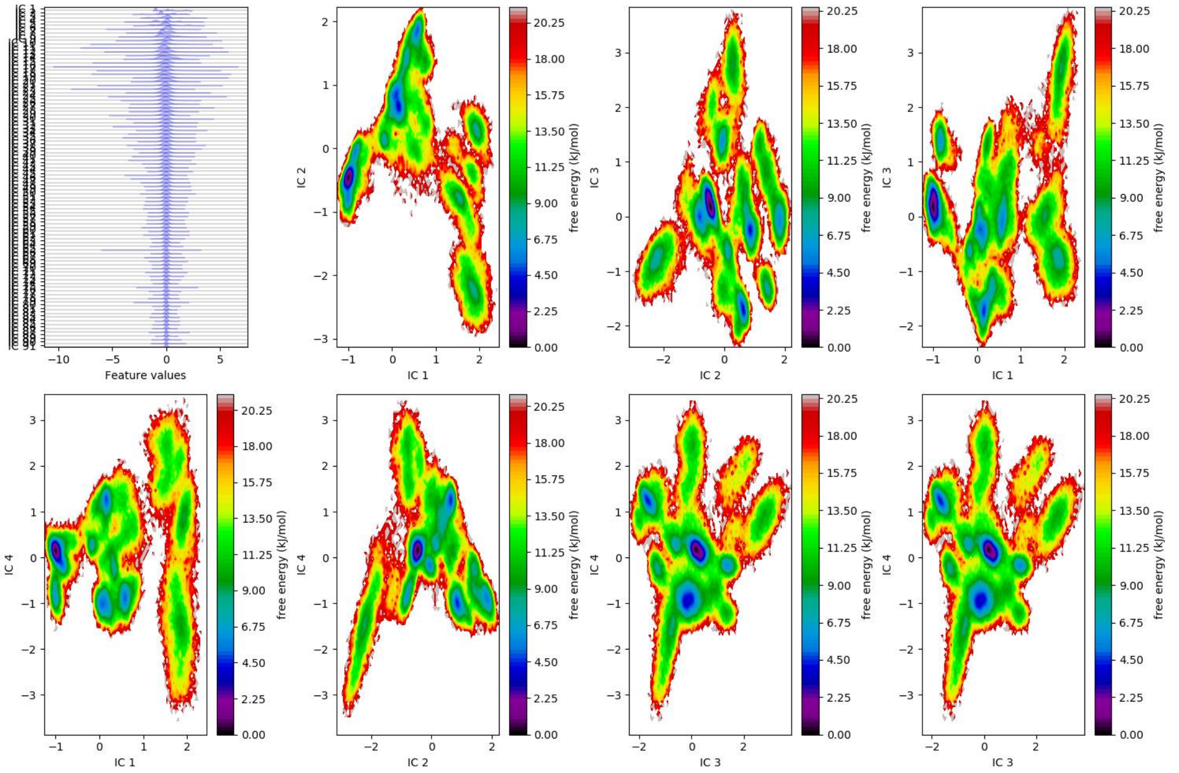

Figure S37 TICA projections between 1-4 for stem-loop in 500mM NaCl concentration

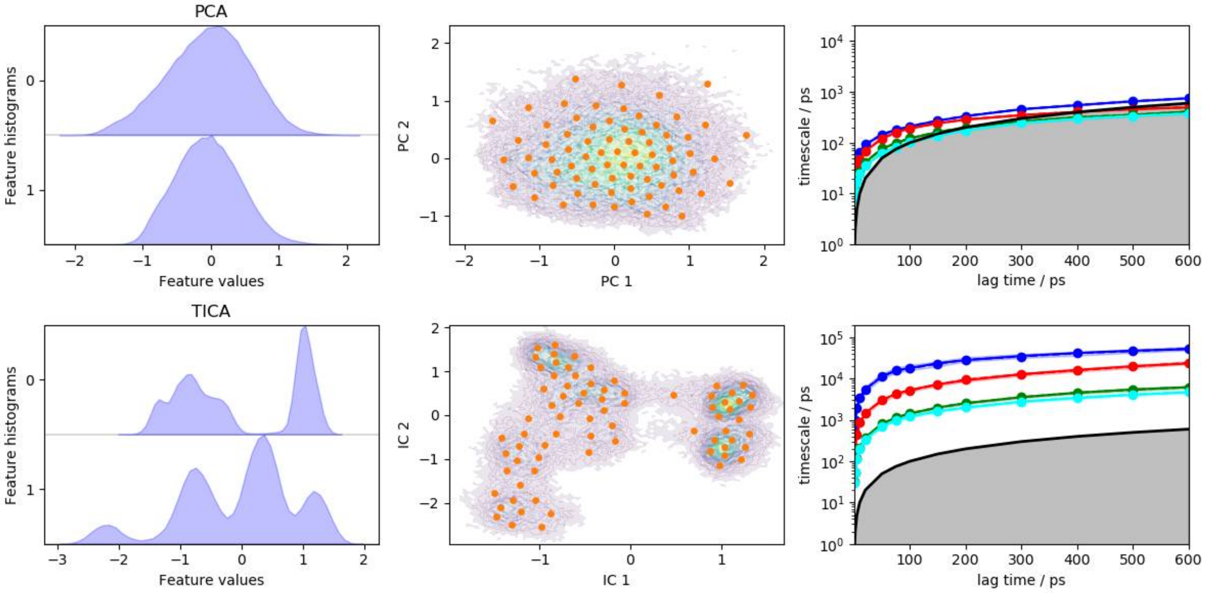

Figure S38 PCA TICA and model quality for 1 to 600 steps of 10ps

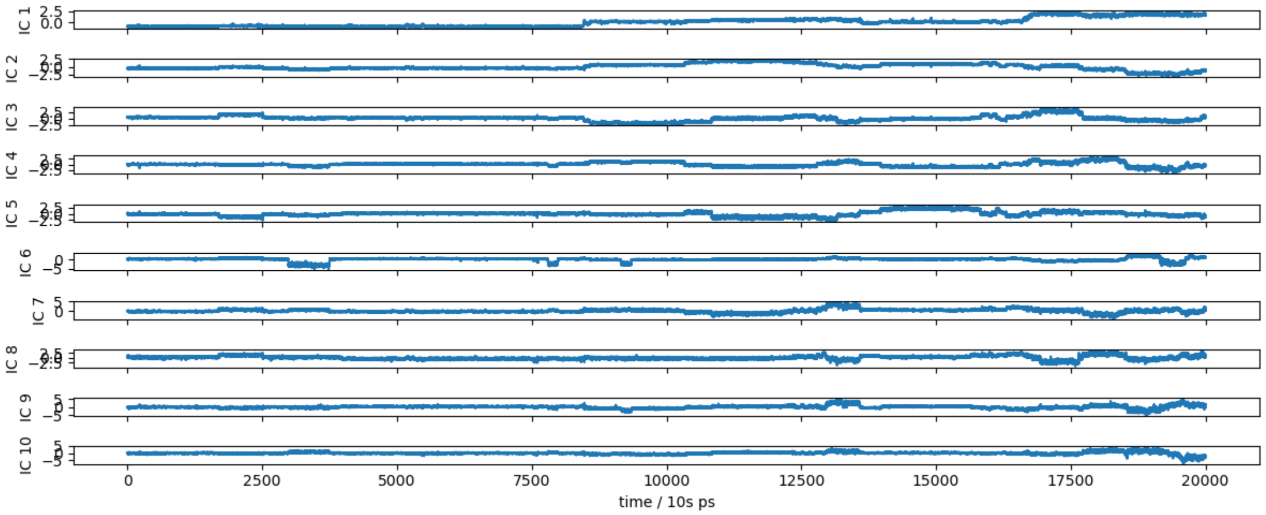

Figure S39 First 10 IC values over the simulation time

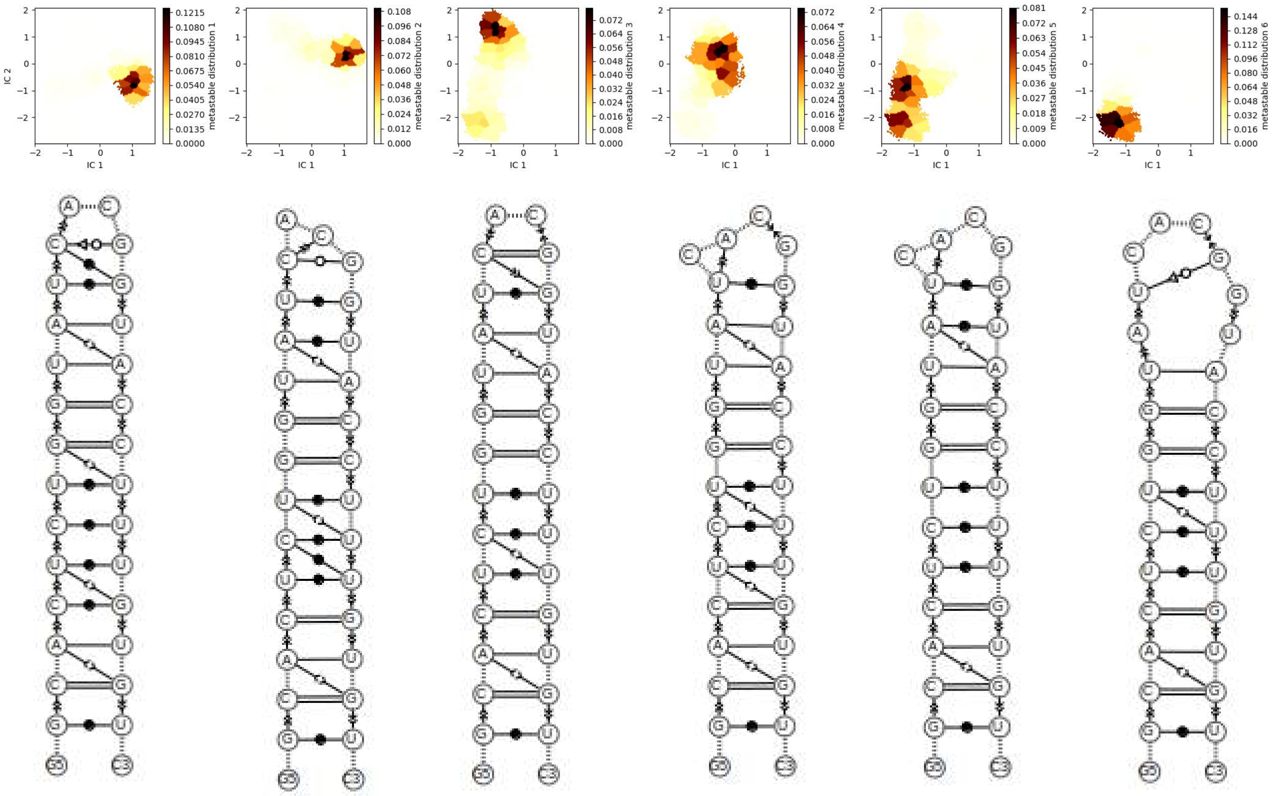

Figure S40 Regions of the TICA landscape and corresponding structure for 500μM NaCl

Poliovirus stem-loop

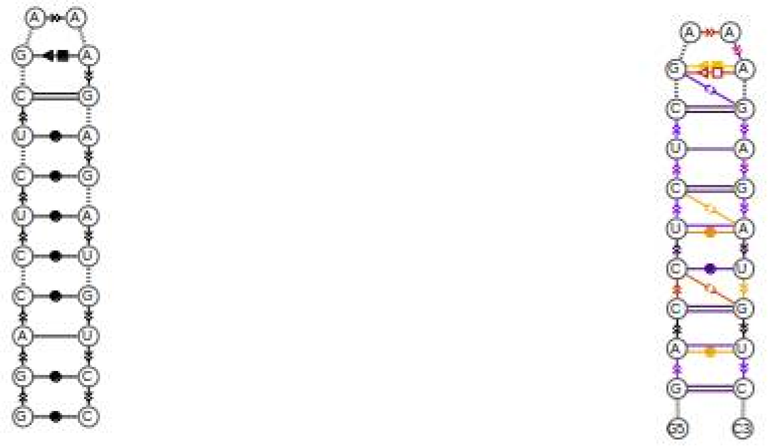

Figure S41 LW representation of deposited structures (left) and after 5μs simulation.

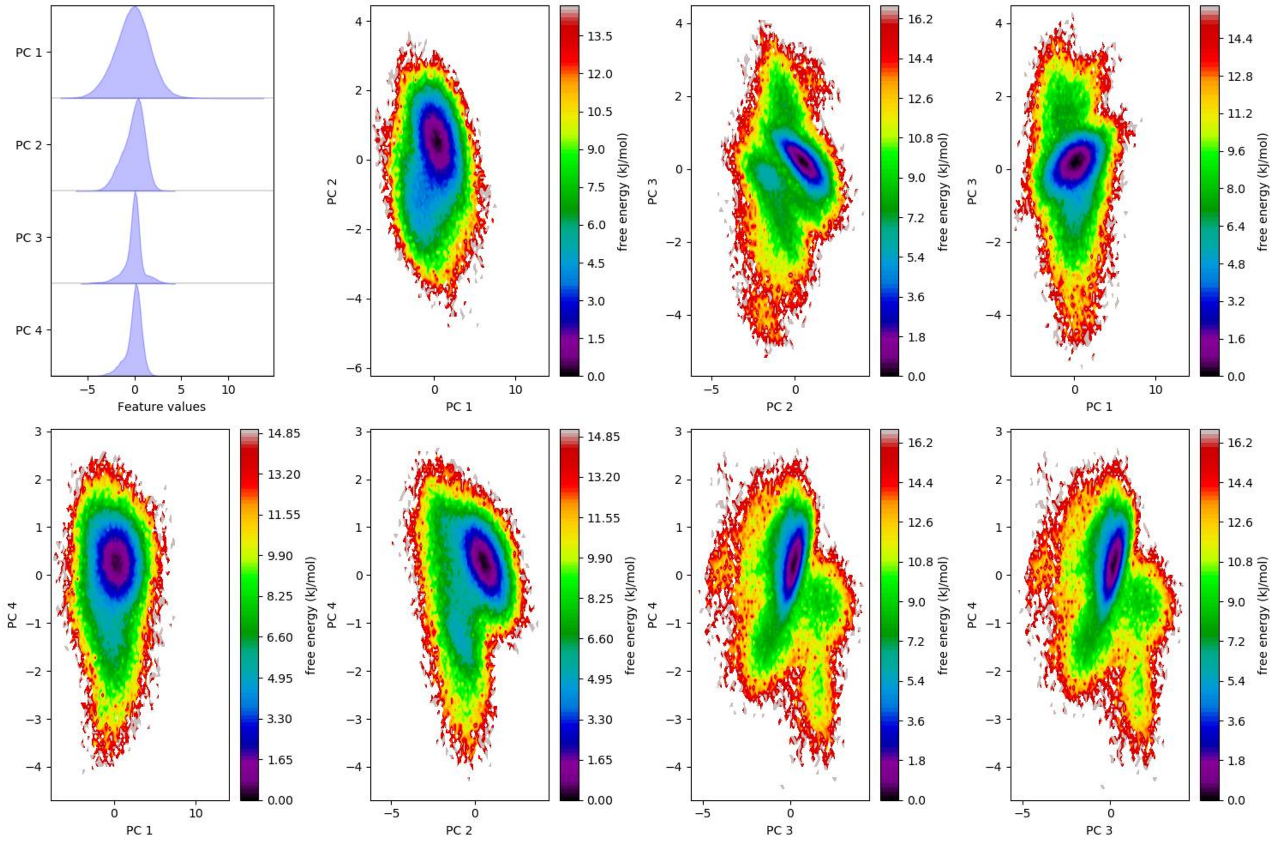

Figure S42 PCA projections between 1-4

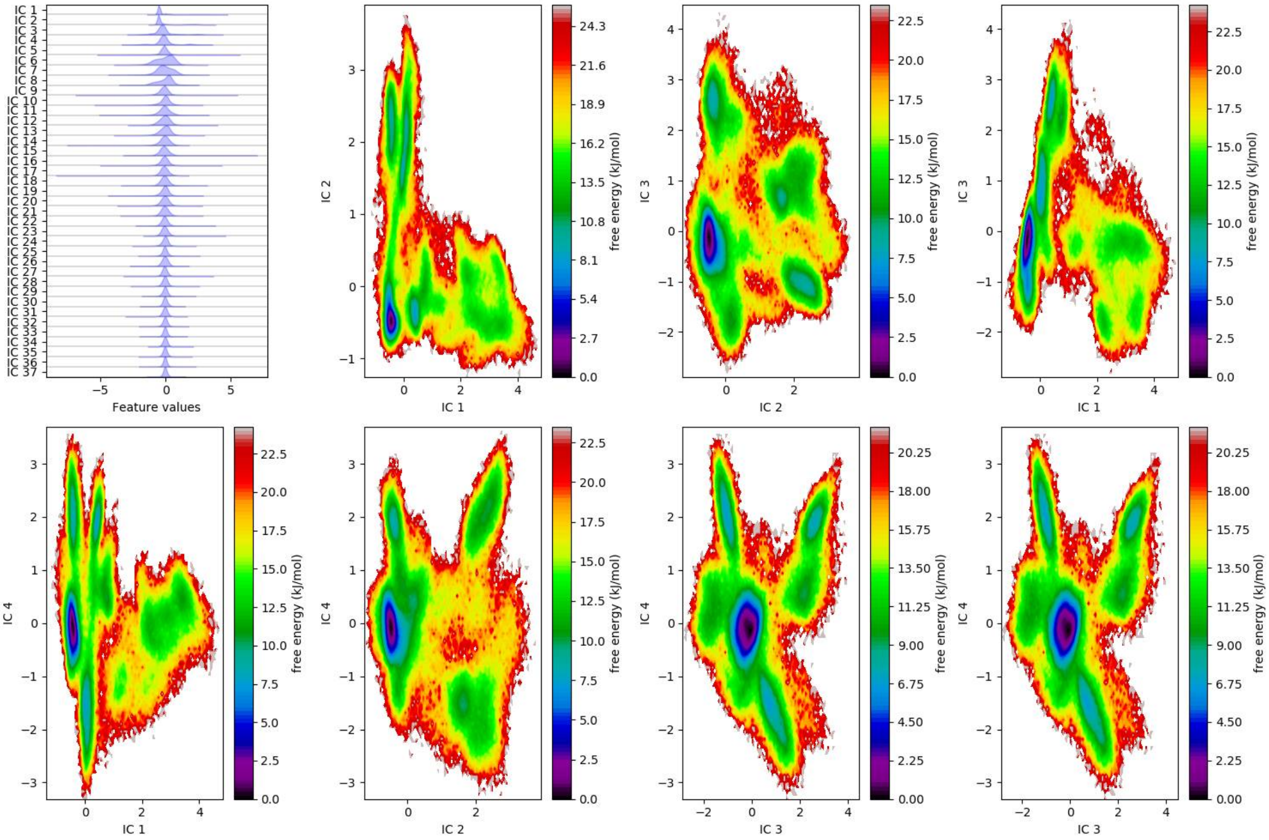

Figure S43 TICA projections between 1-4

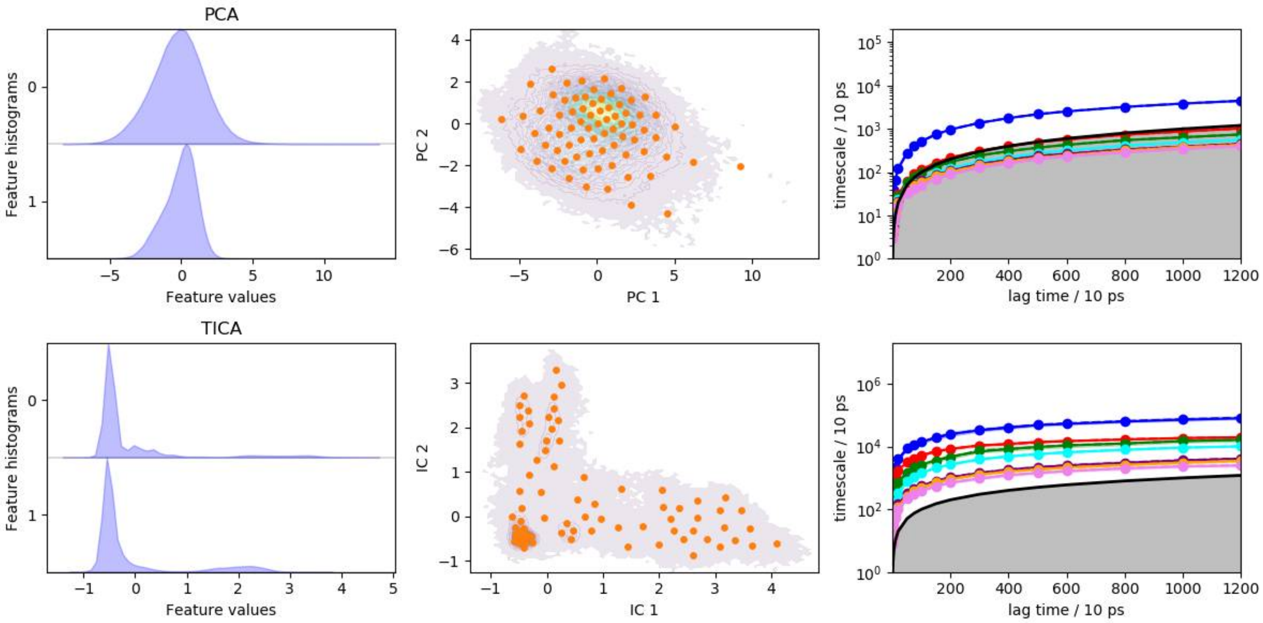

Figure S44 PCA TICA and model quality for 1 to 1200 steps of 10ps

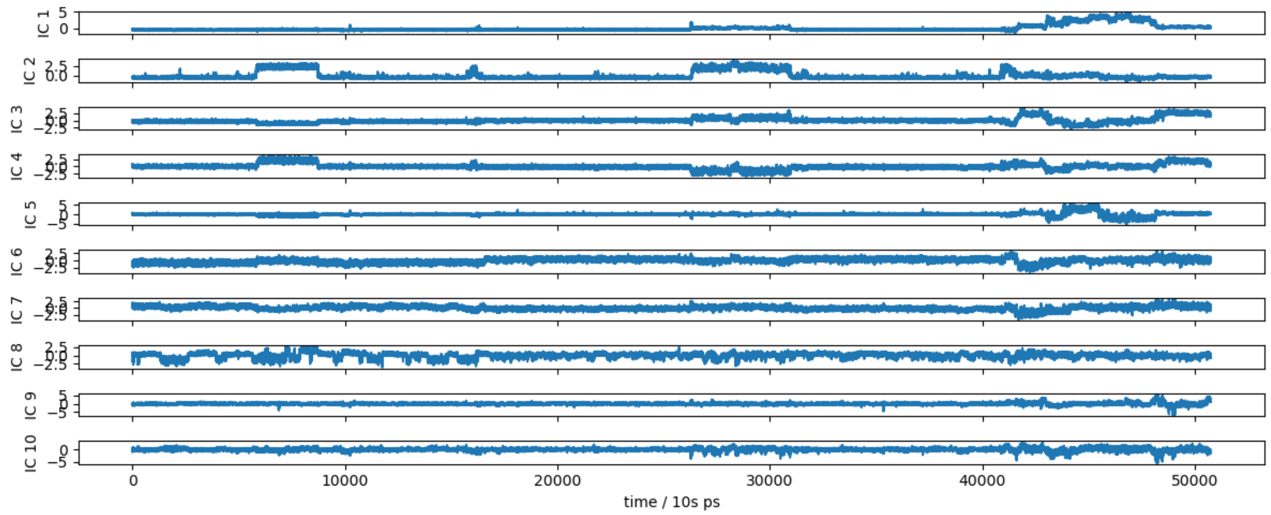

Figure S45 First 10 IC values over the simulation time

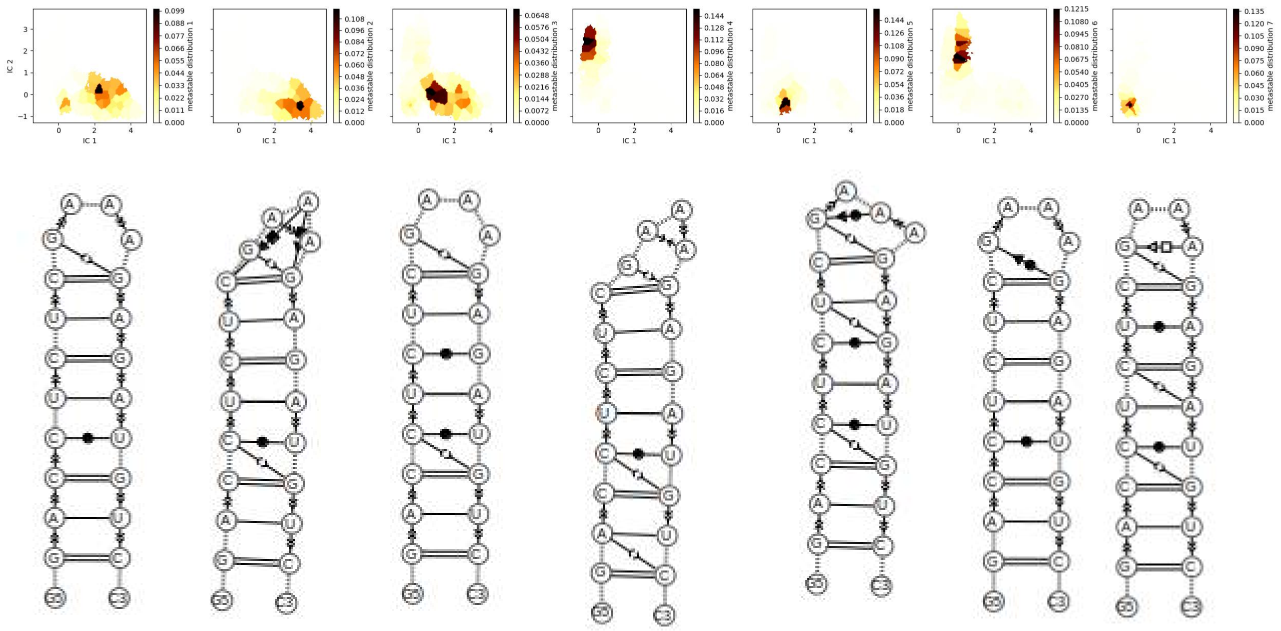

Figure S46 Regions of the TICA landscape and corresponding structure

Poliovirus with P enantiomer

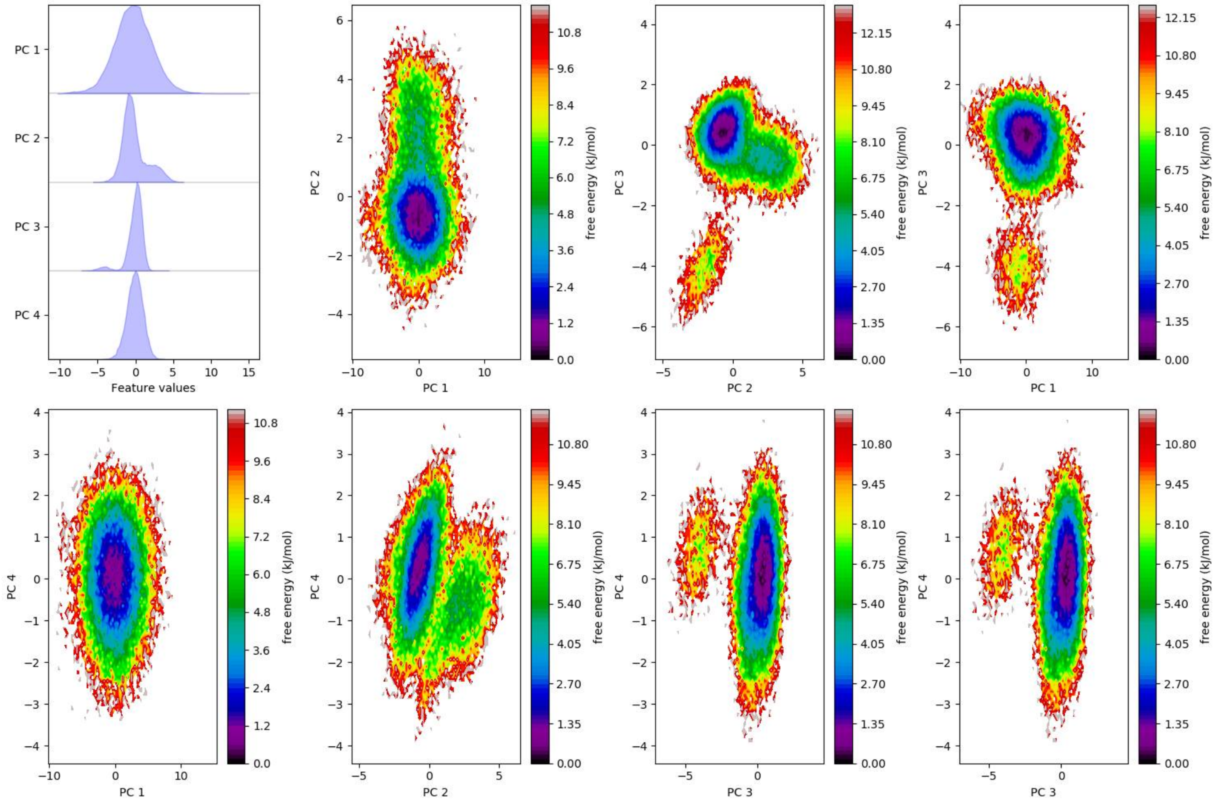

Figure S47 PCA projections between 1-4

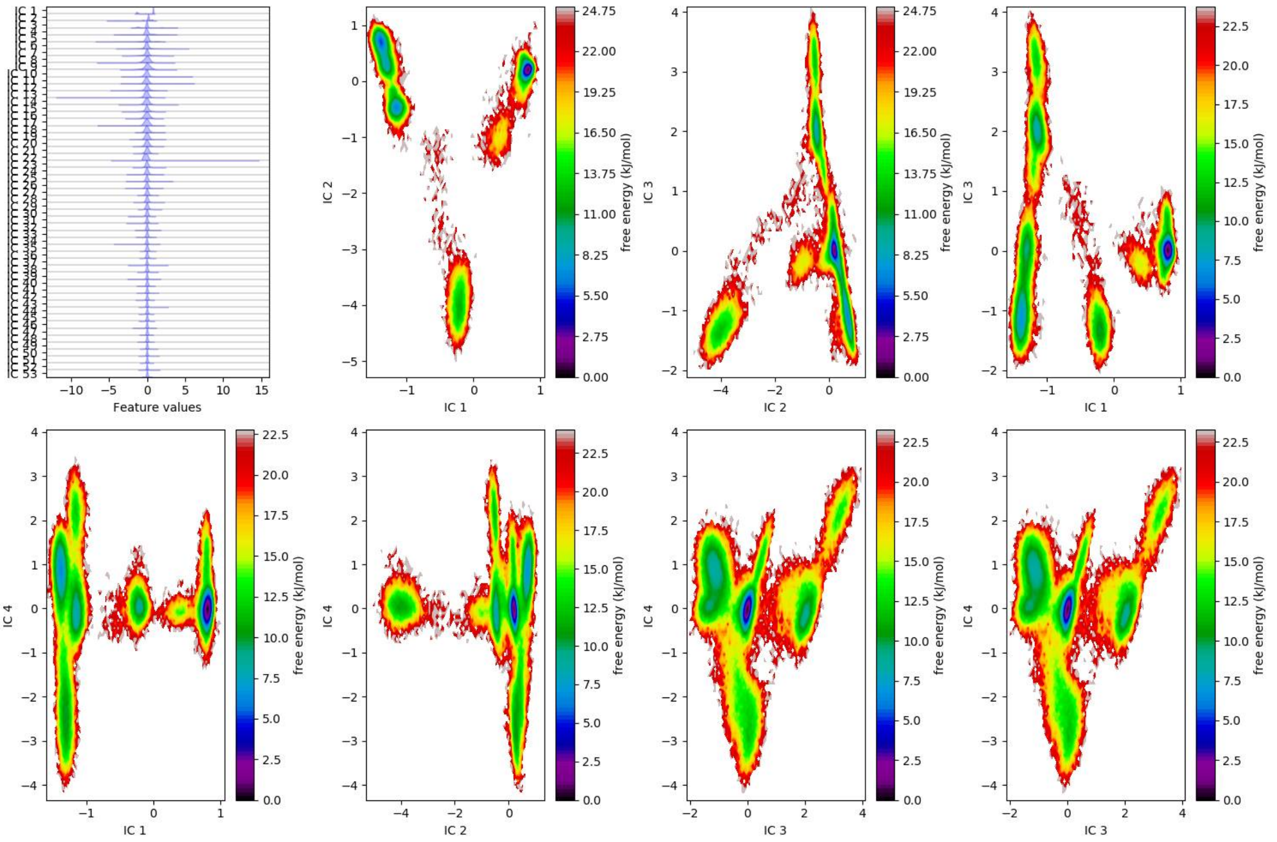

Figure S48 TICA projections between 1-4

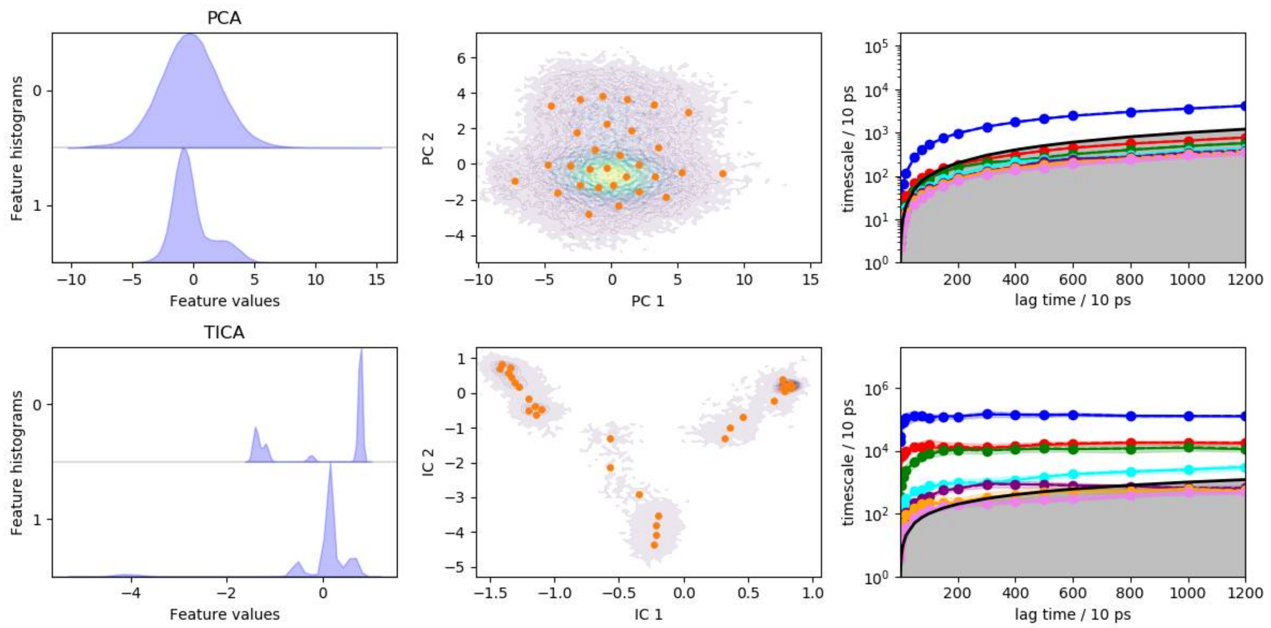

Figure S49 PCA TICA and model quality for 1 to 1200 steps of 10ps

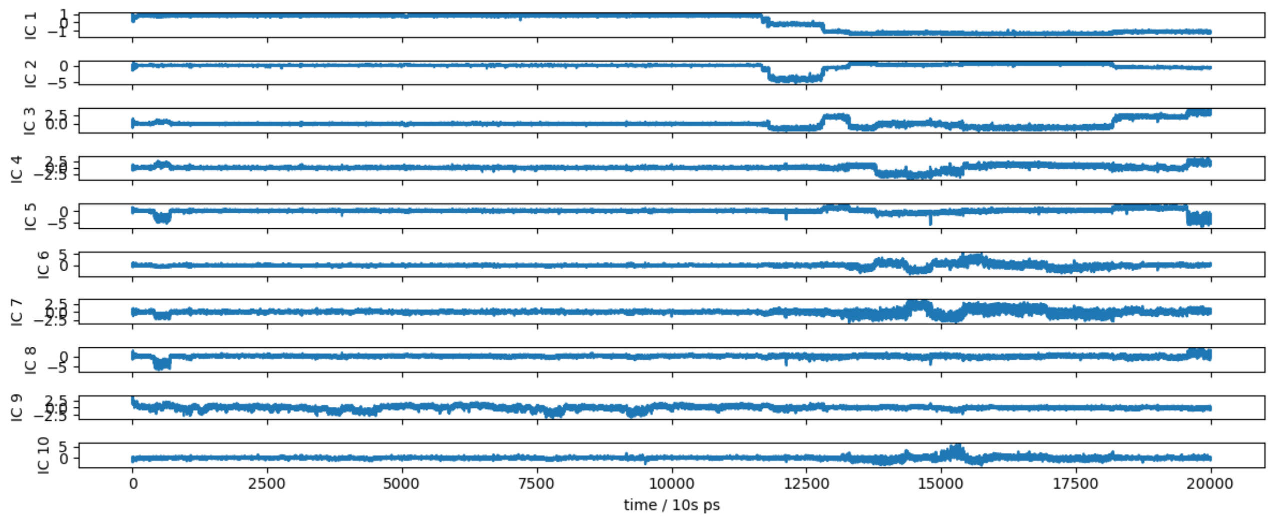

Figure S50 First 10 IC values over the simulation time

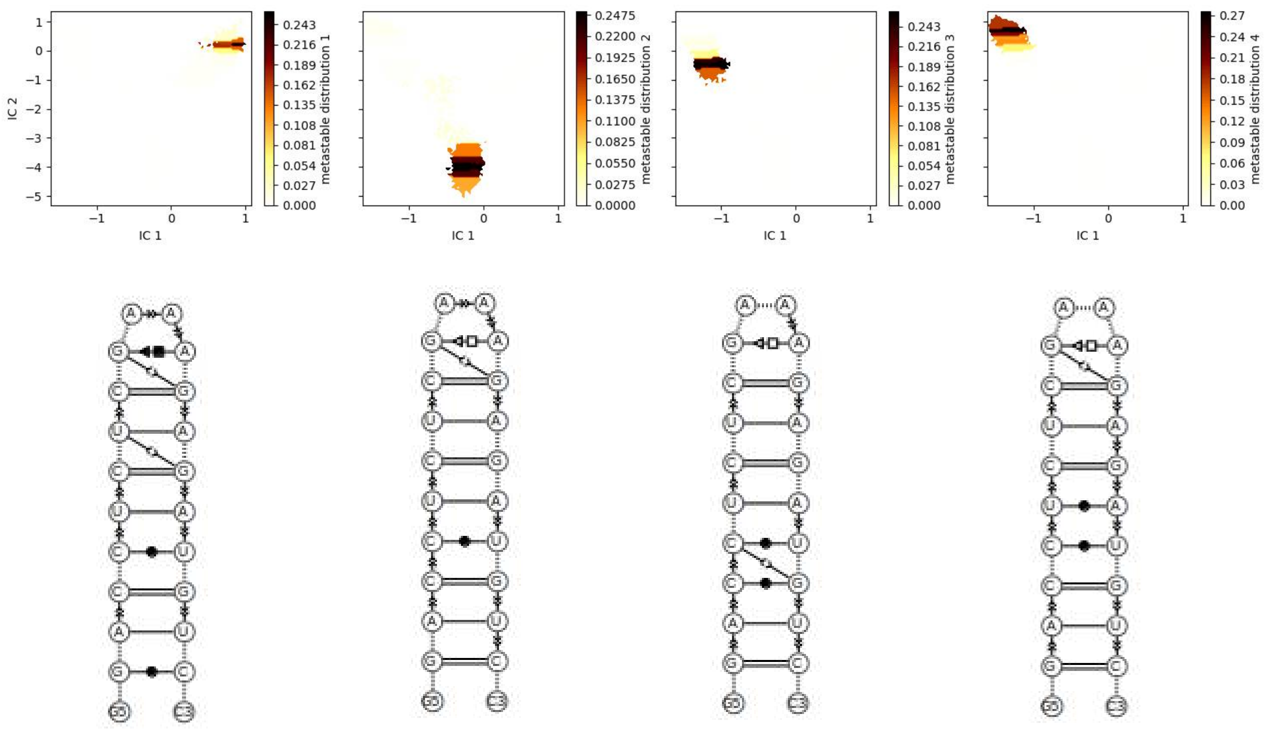

Figure S51 Regions of the TICA landscape and corresponding structure

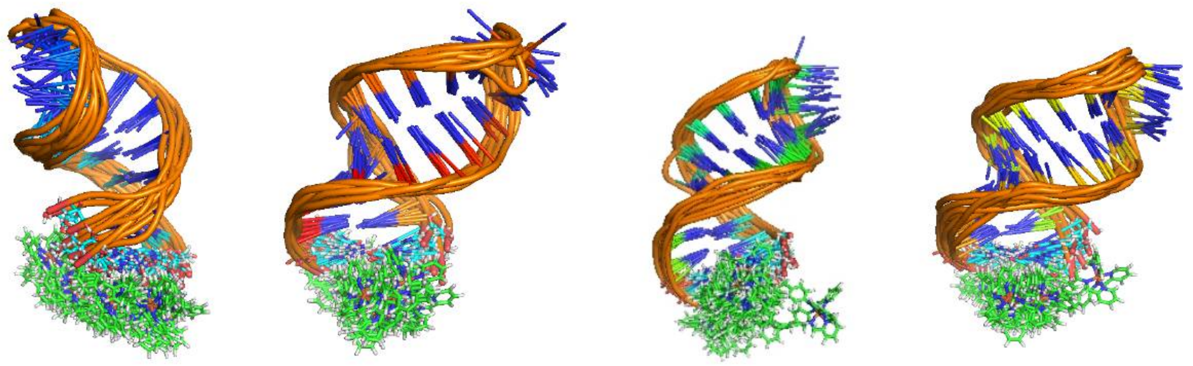

Figure S52 3D representation of the corresponding micro-states.

Poliovirus with M enantiomer

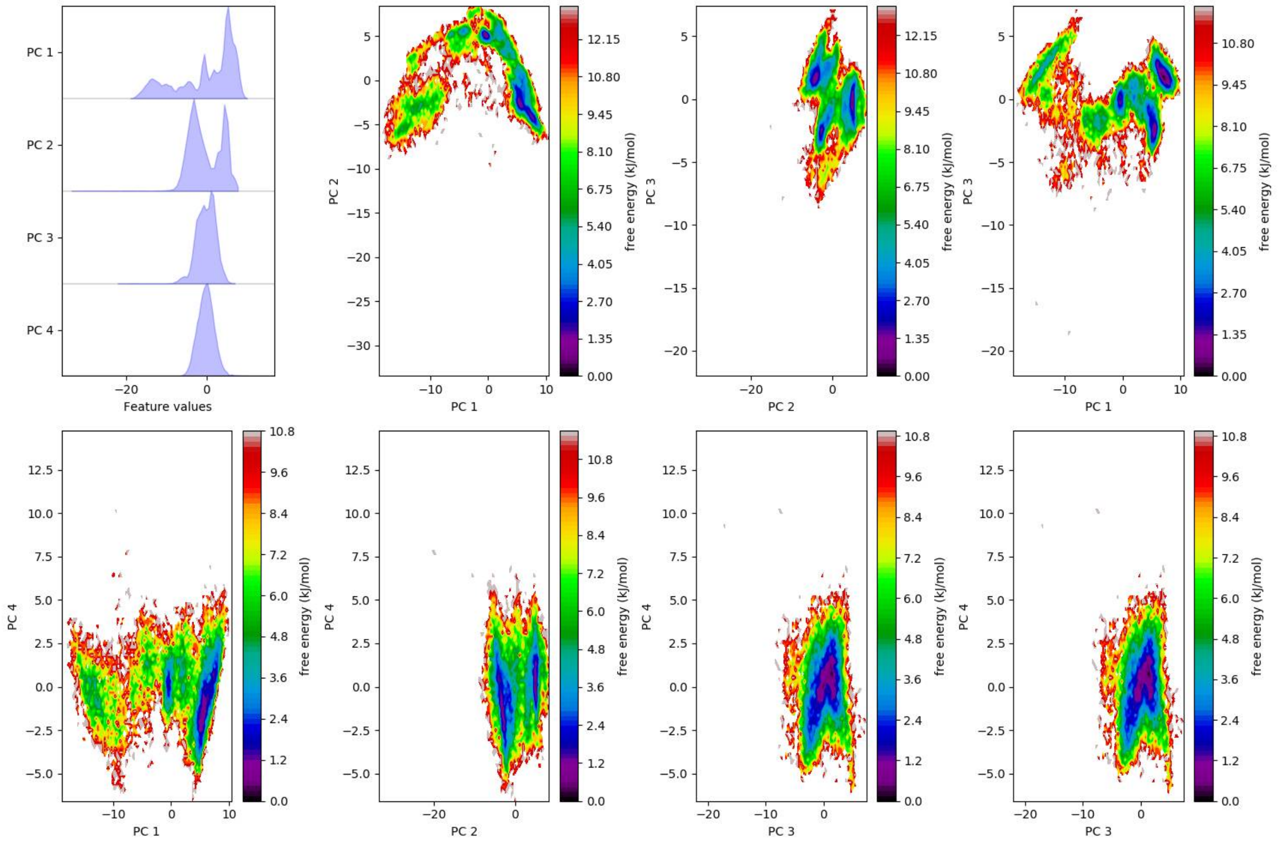

Figure S53 PCA projections between 1-4

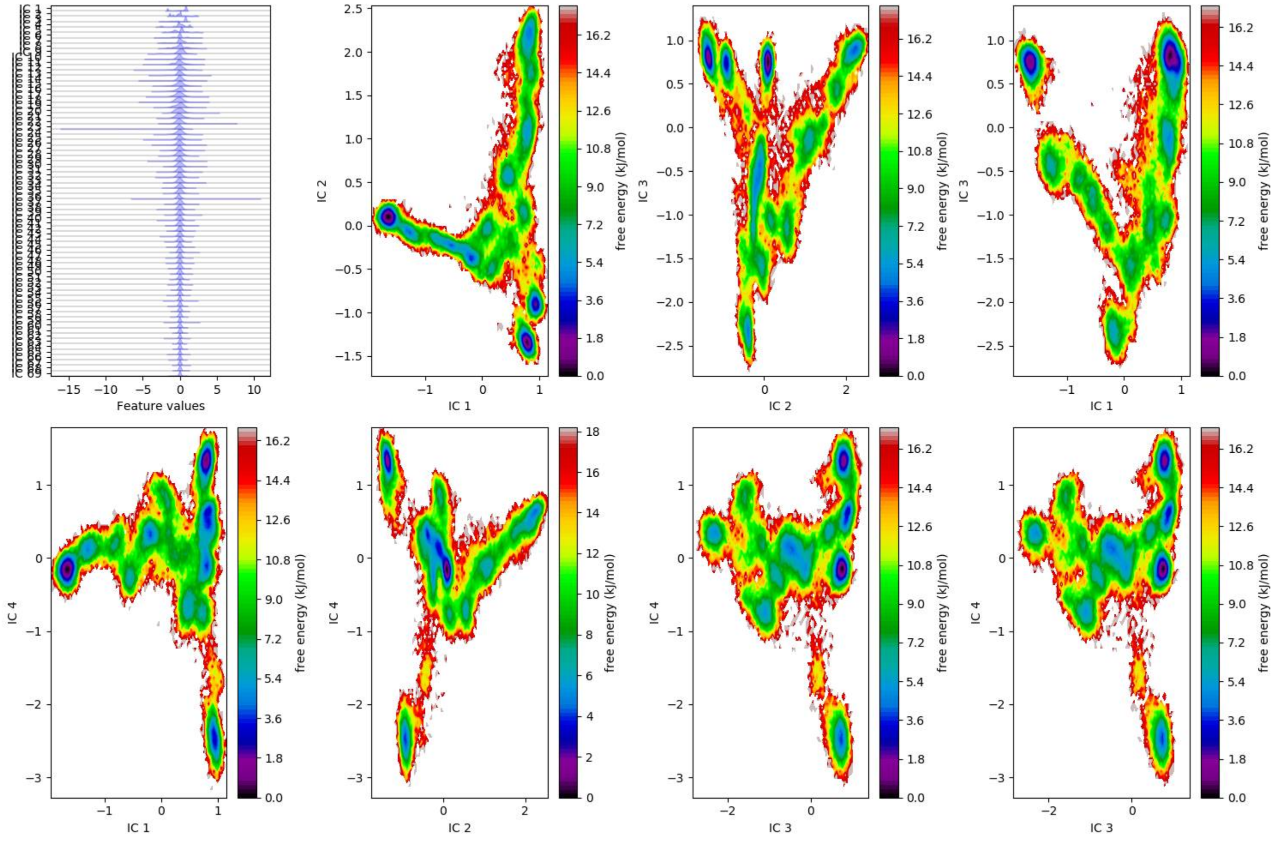

Figure S54 TICA projections between 1-4

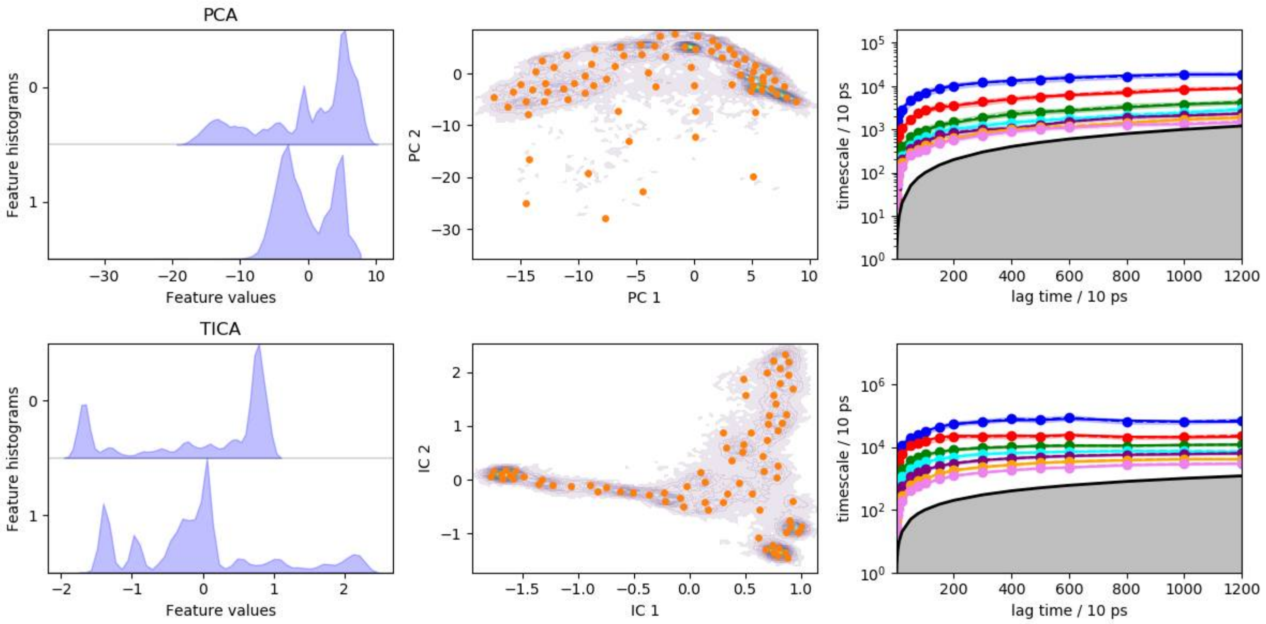

Figure S55 PCA TICA and model quality for 1 to 1200 steps of 10ps

HIV2

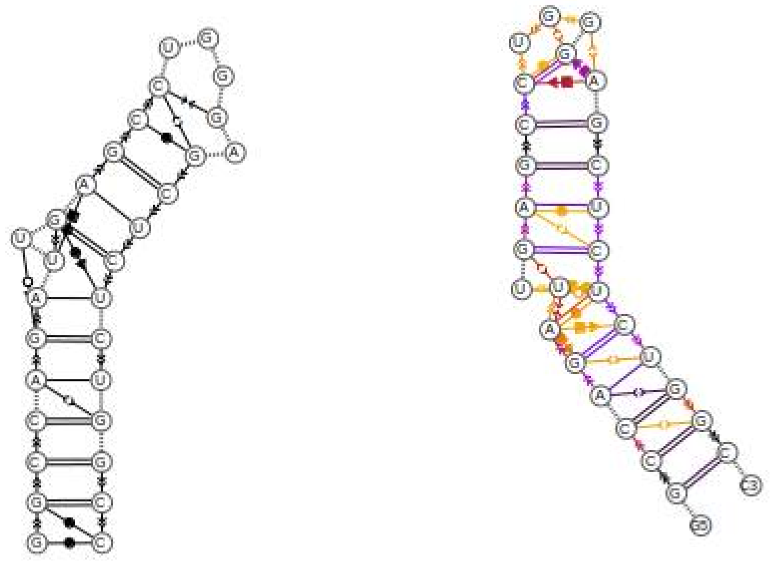

Figure S56 LW representation of deposited structures (left) and after 3μs simulation of HIV2-TAR element

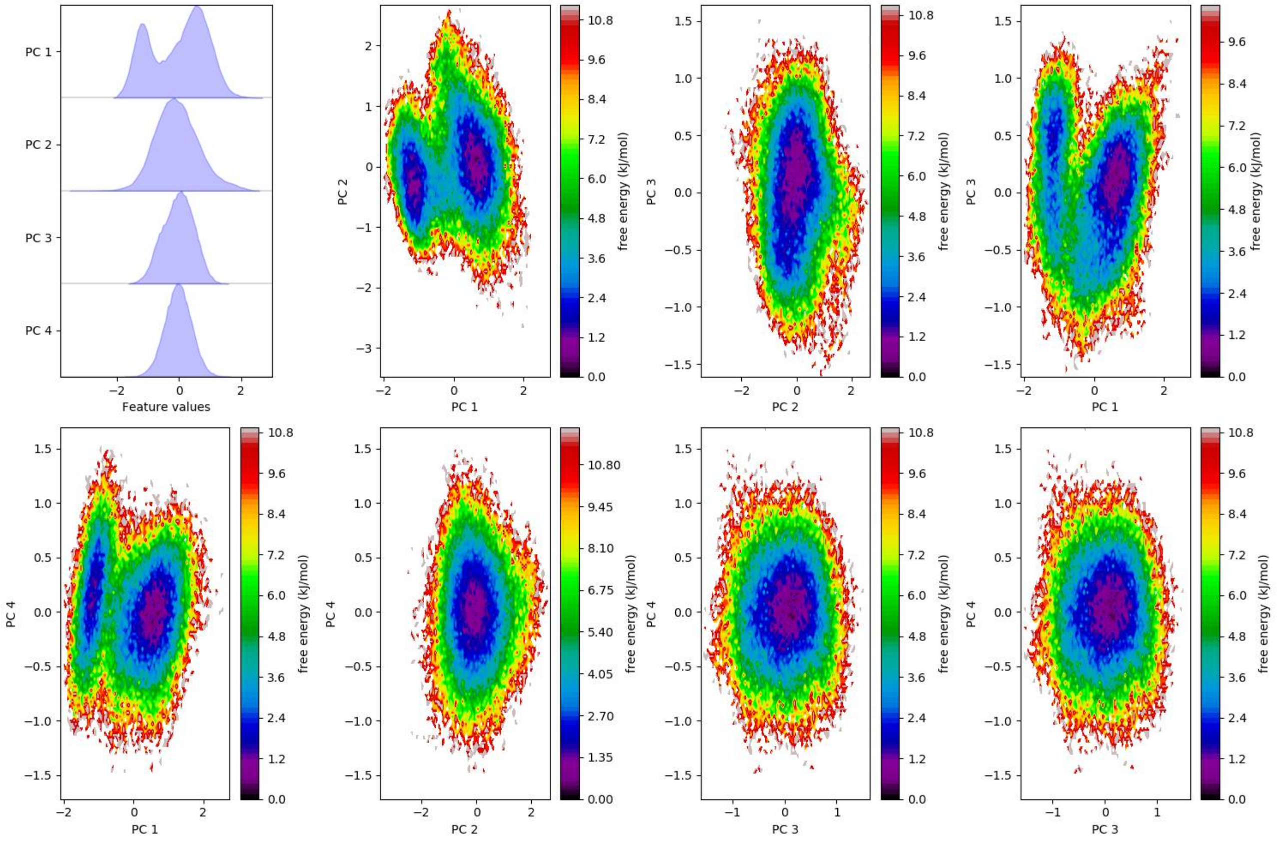

Figure S57 PCA projections between 1-4

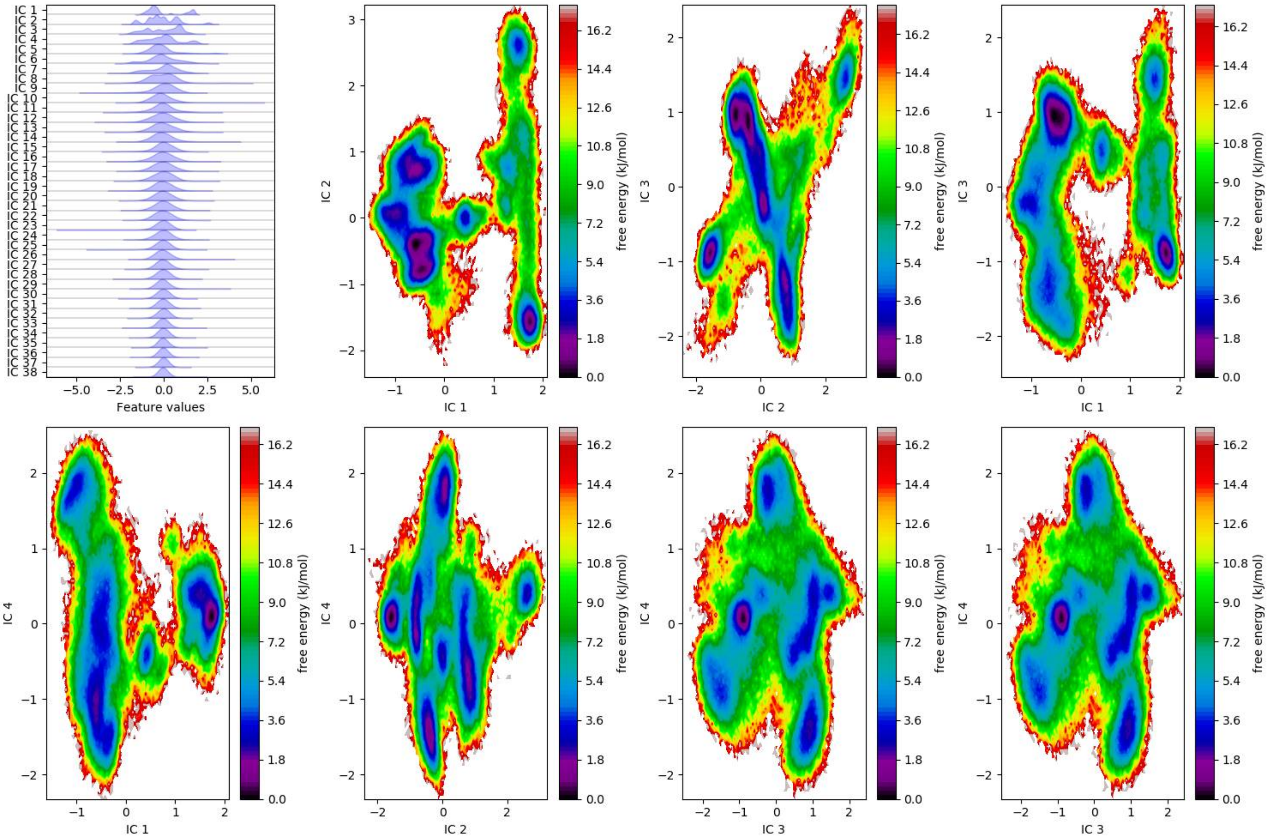

Figure S58 TICA projections between 1-4

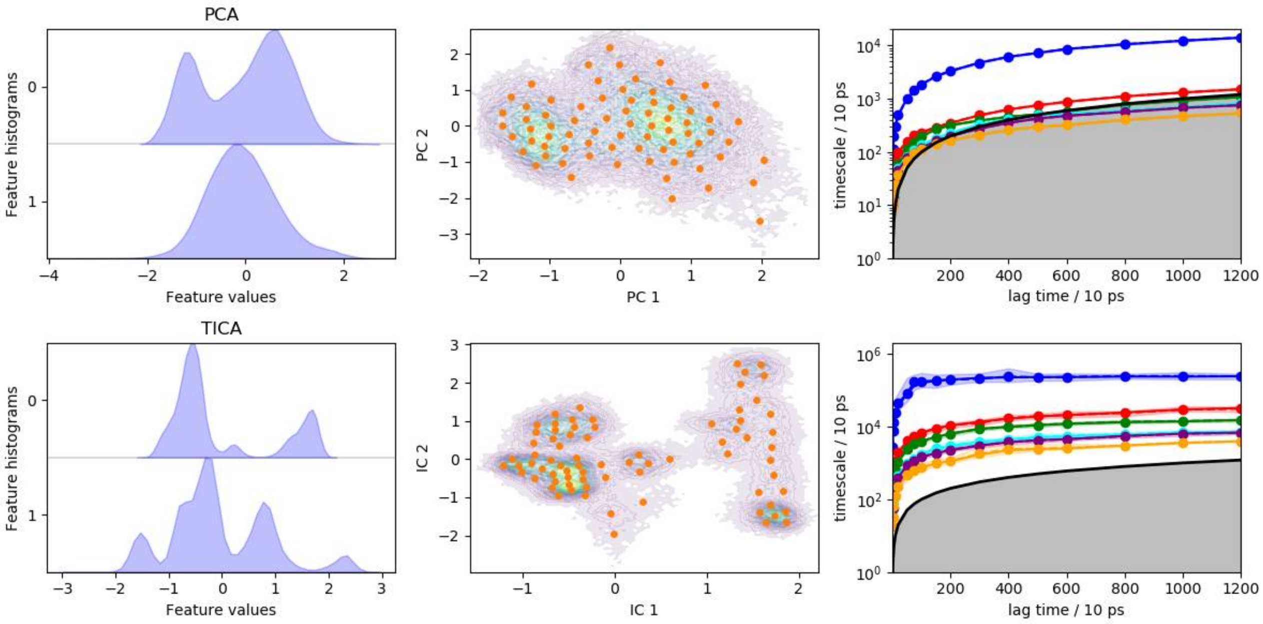

Figure S59 PCA TICA and model quality for 1 to 1200 steps of 10ps

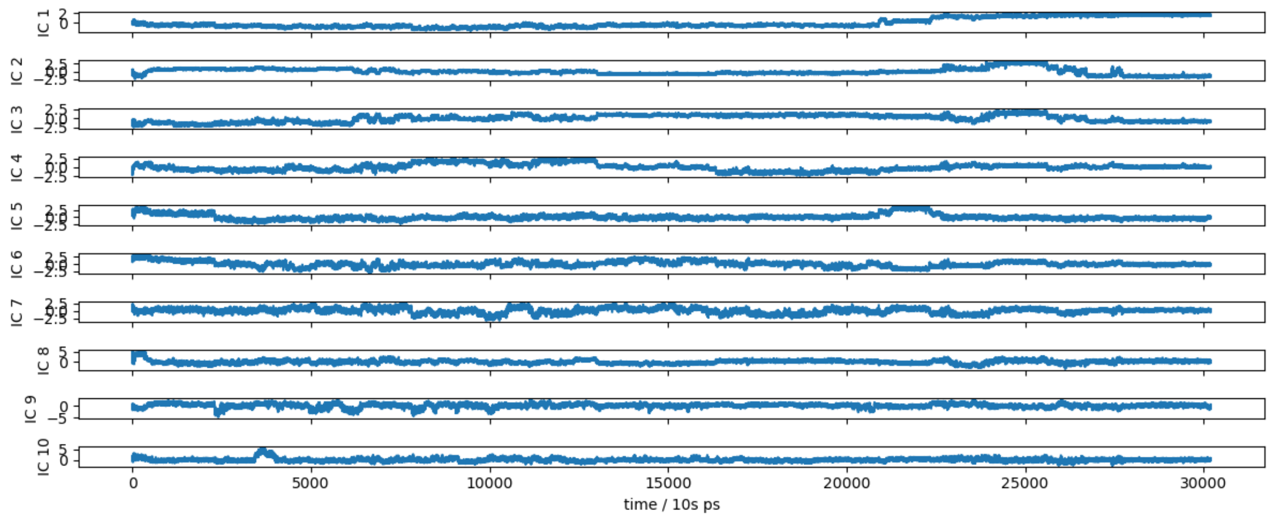

Figure S60 First 10 IC values over the simulation time

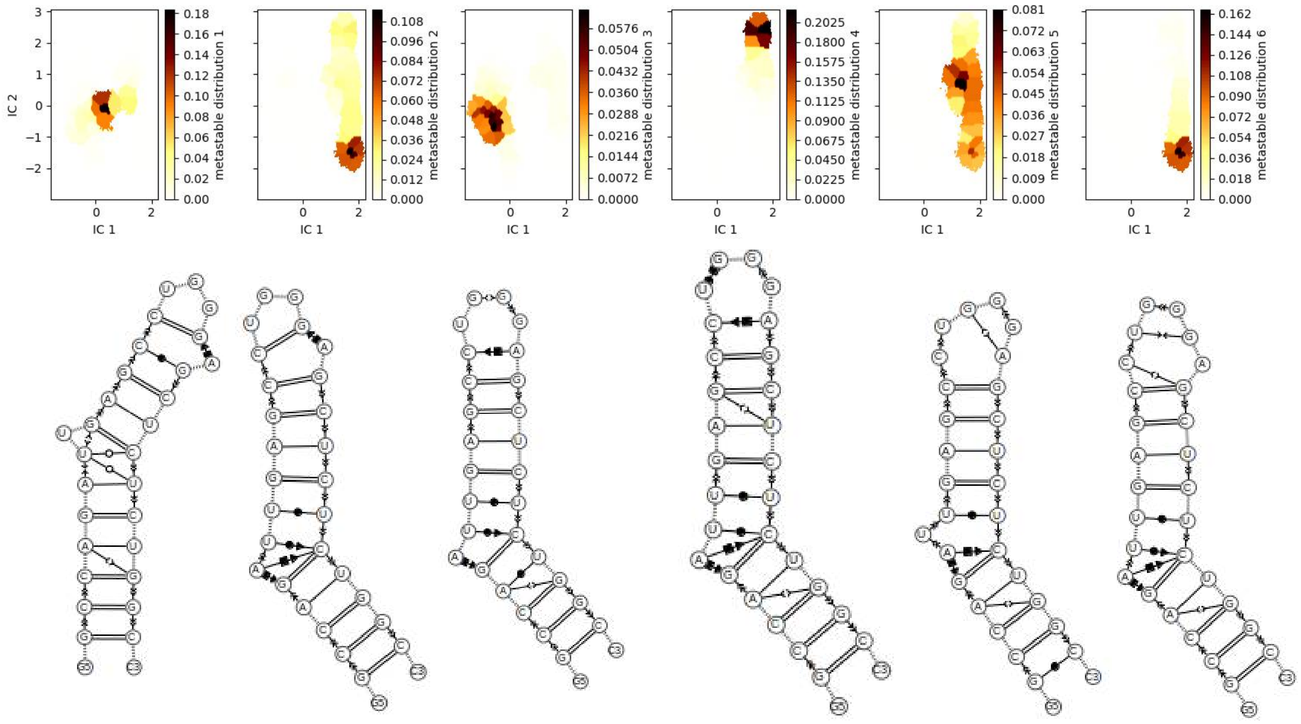

Figure S61 Regions of the TICA landscape and corresponding structure

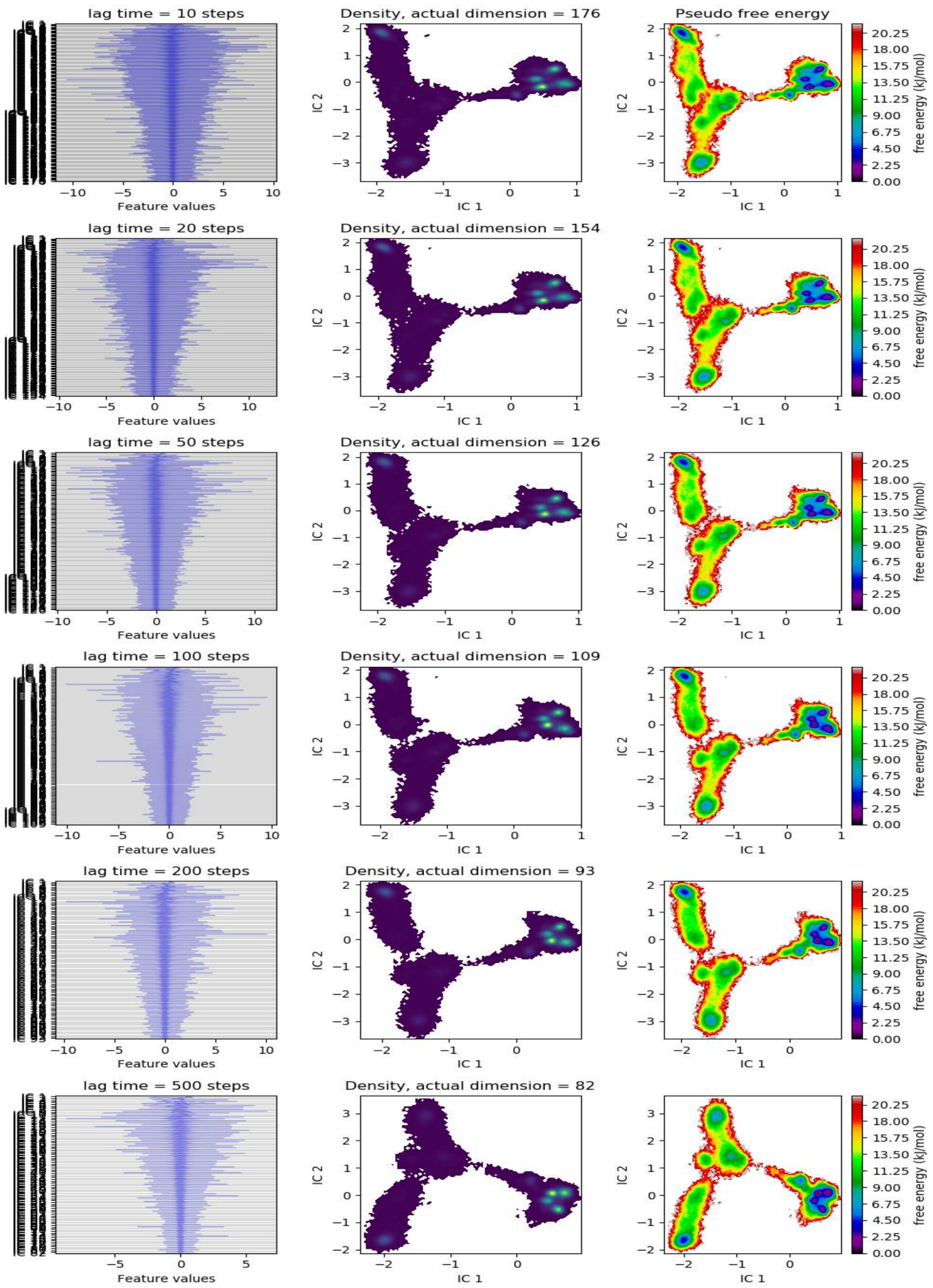

Figure S62 TICA dimensions, density and corrected heat map for steps 10 to 500

HIV-1 TAR (Free)
1ANR

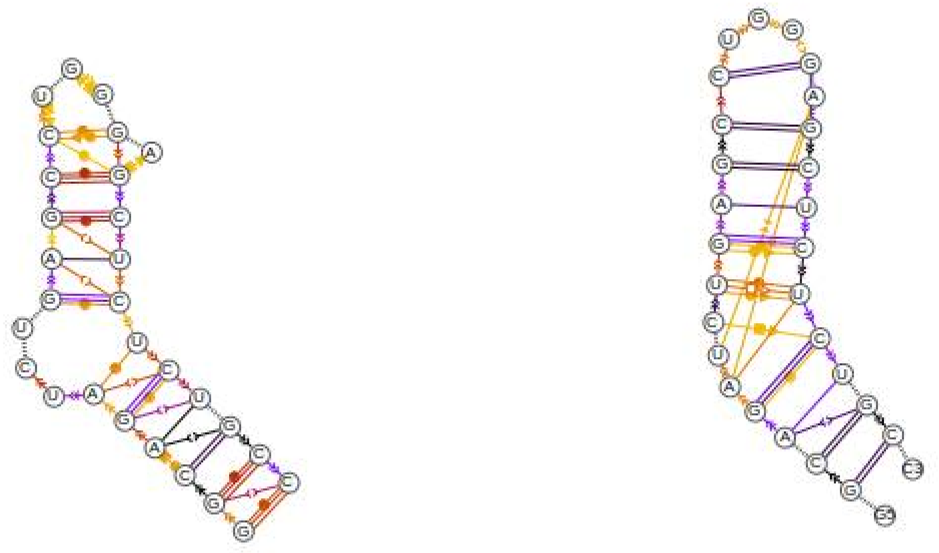

Figure S63 LW representation of 1ANR solutions (left) and after 10μs simulation (right)

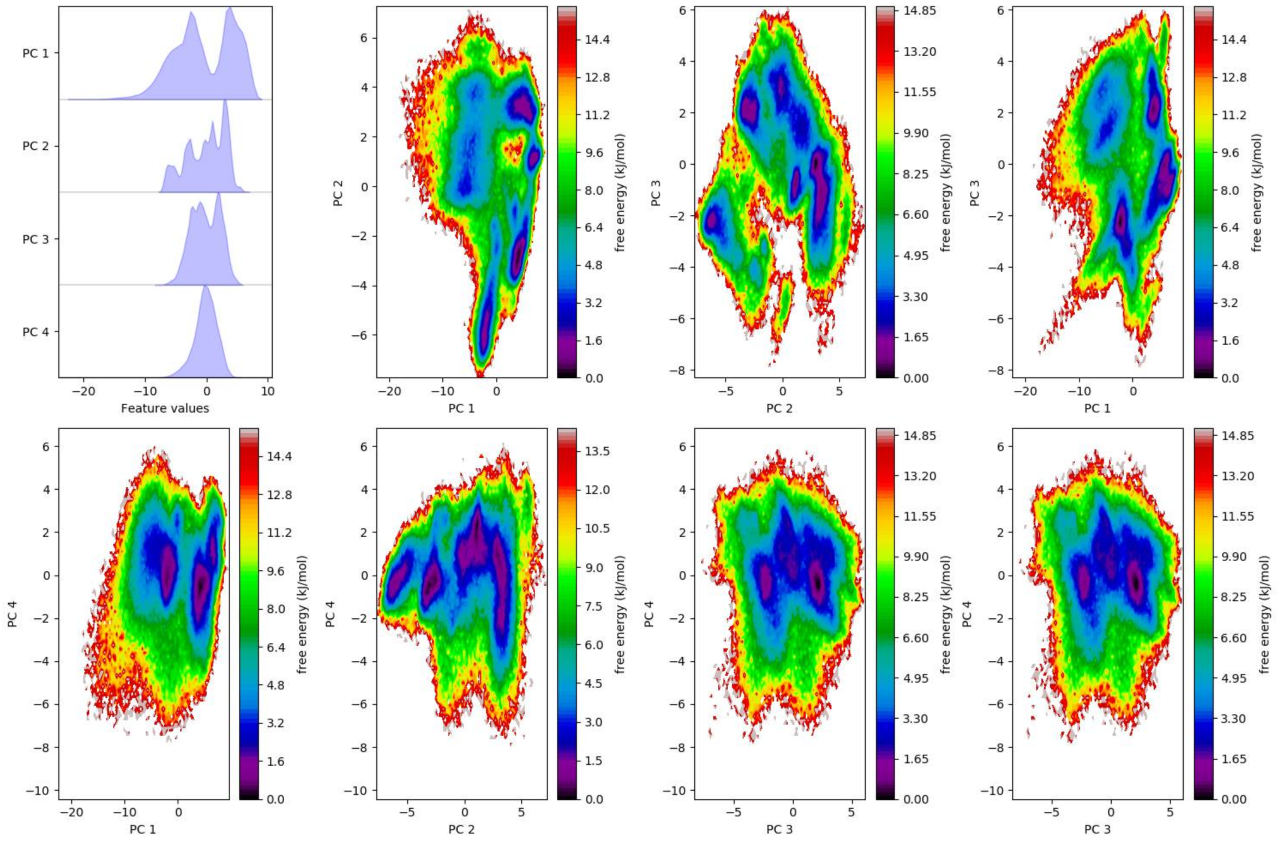

Figure S64 PCA projections between 1-4

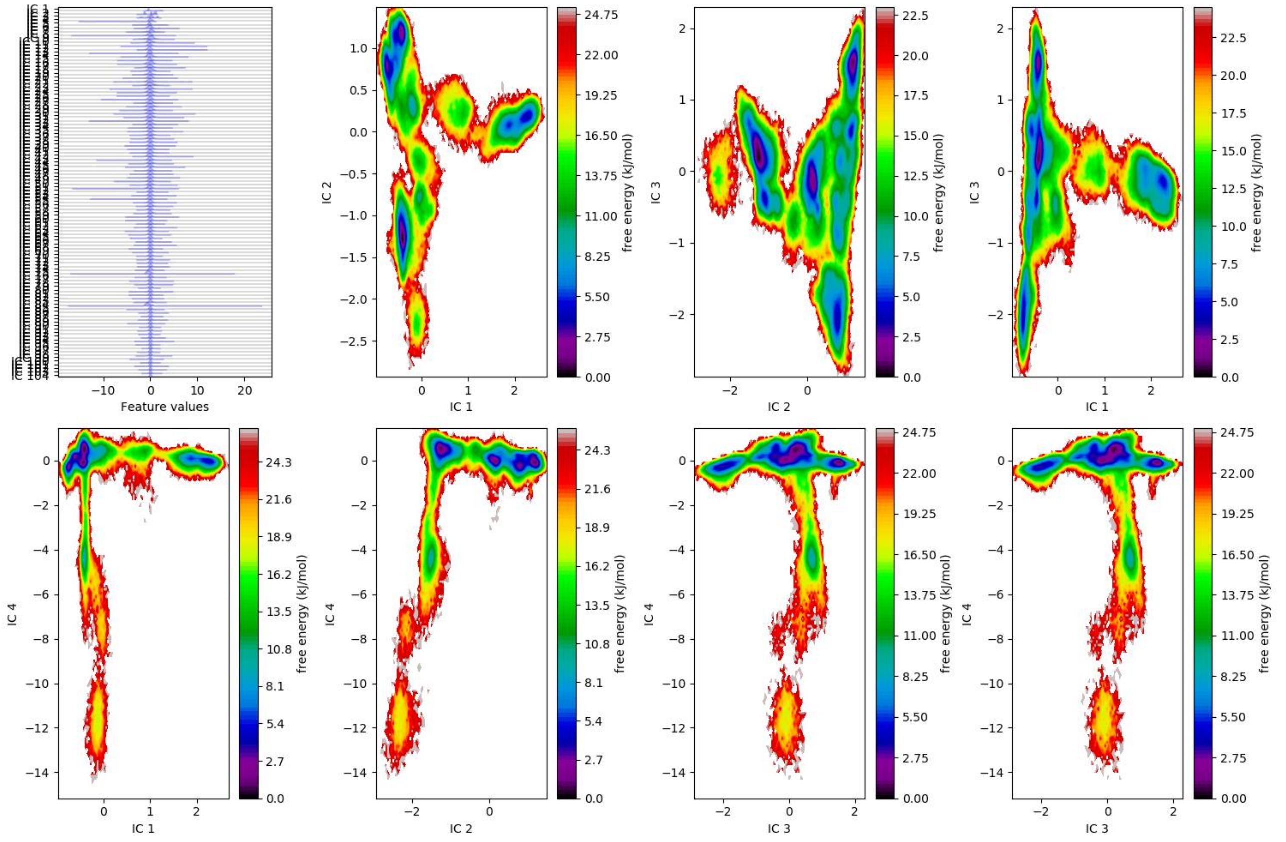

Figure S65 TICA projections between 1-4

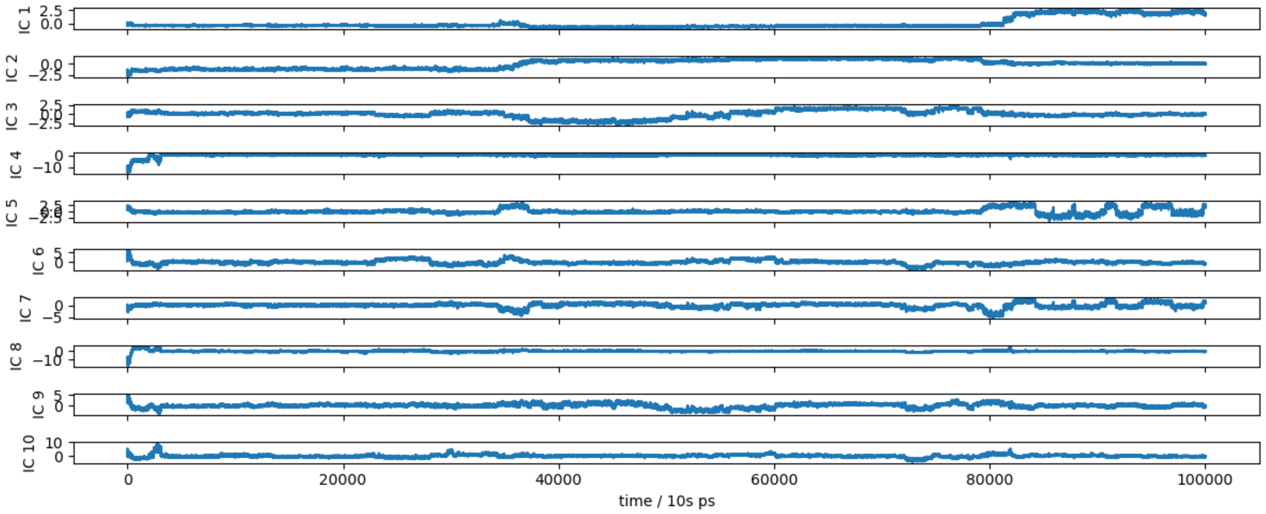

Figure S66 First 10 IC values over the simulation time

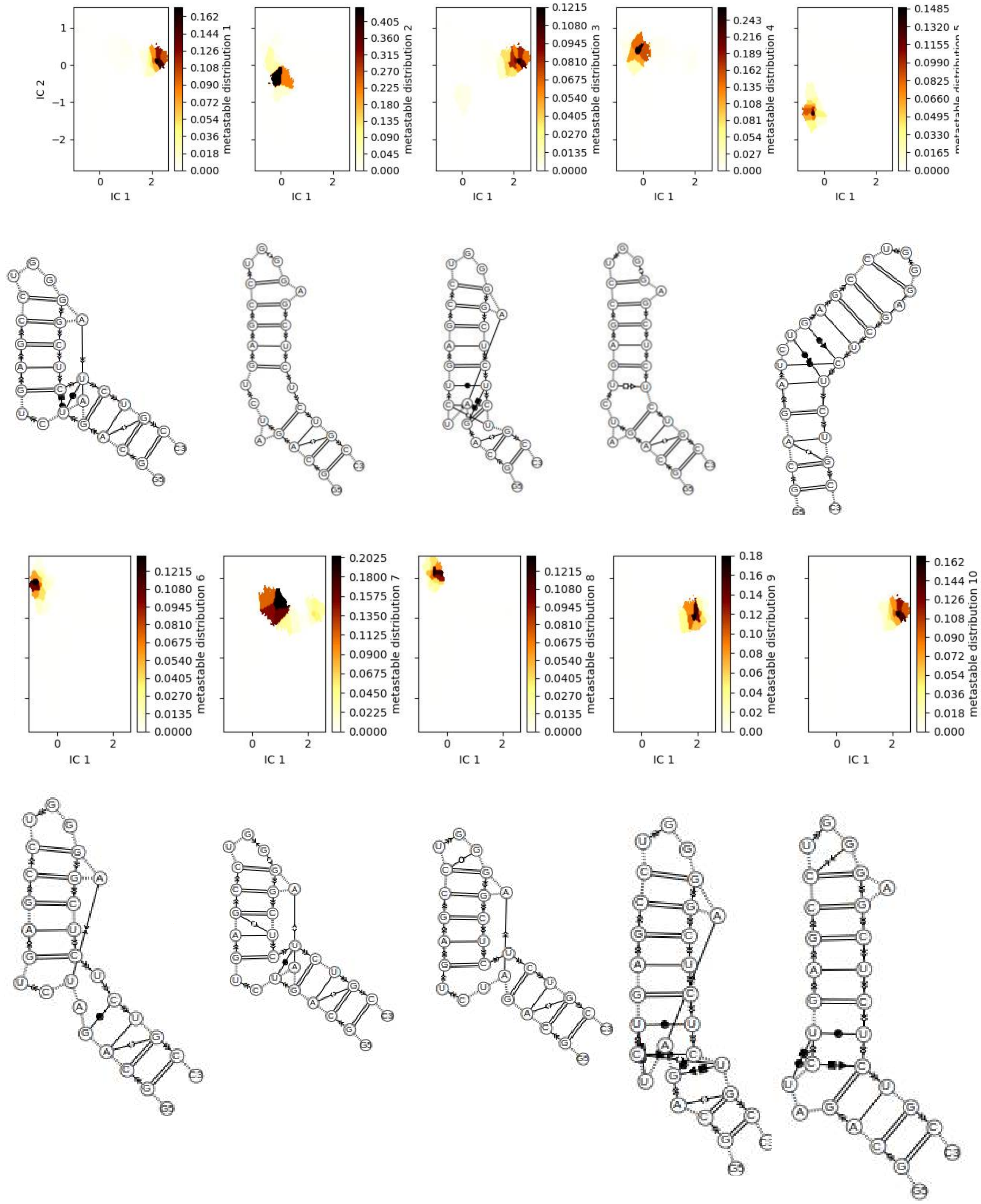

Figure S67 Regions of the TICA landscape and corresponding structure

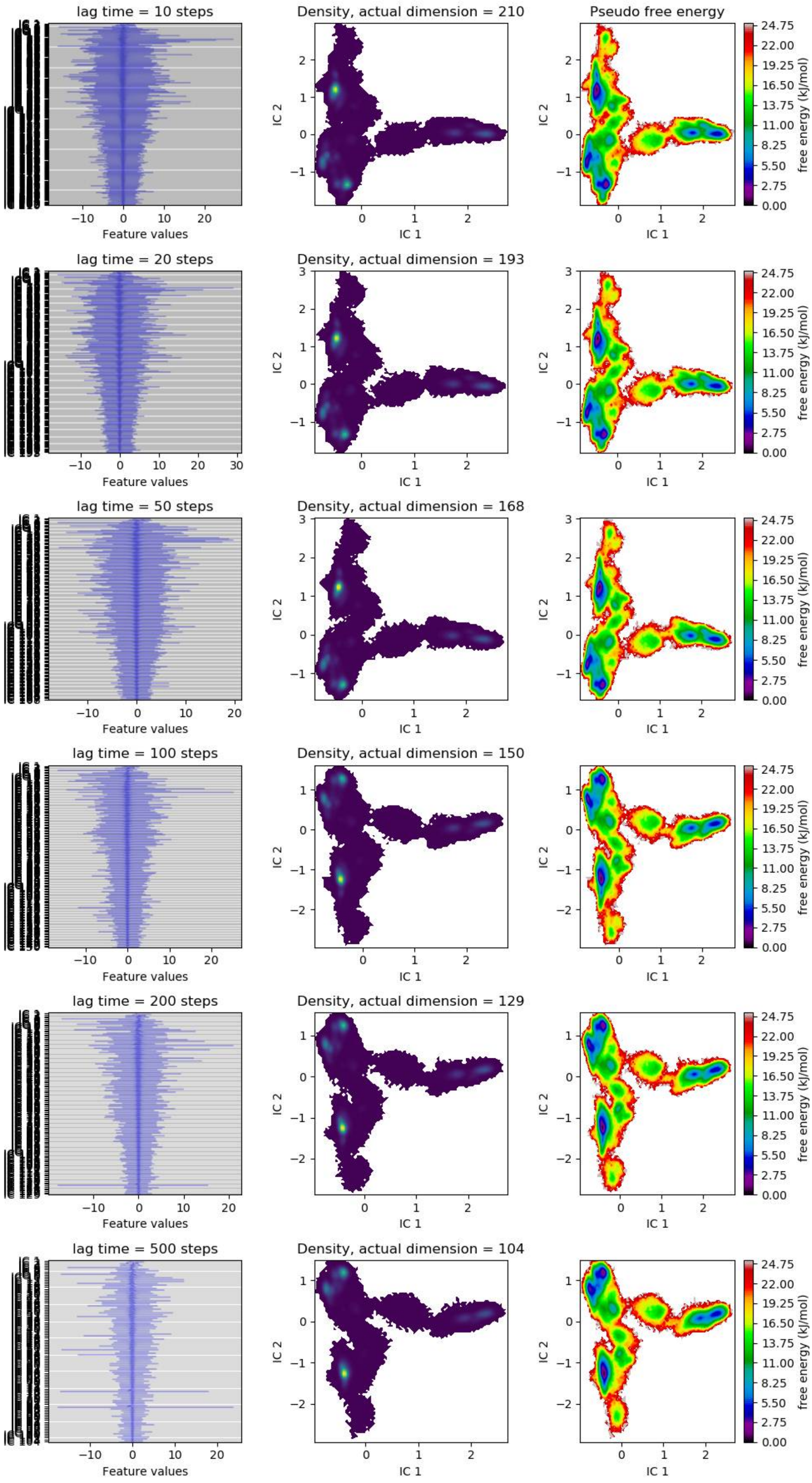

Figure S68 TICA dimensions, density and corrected heat map for steps 10 to 500

1ANR 1 _2

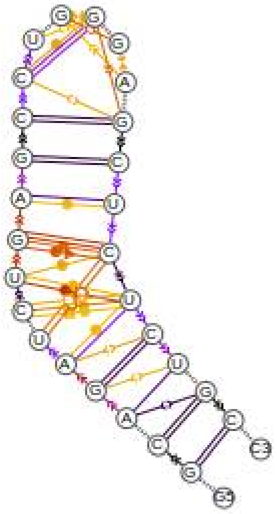

Figure S69 LW representation of the 6μs simulation

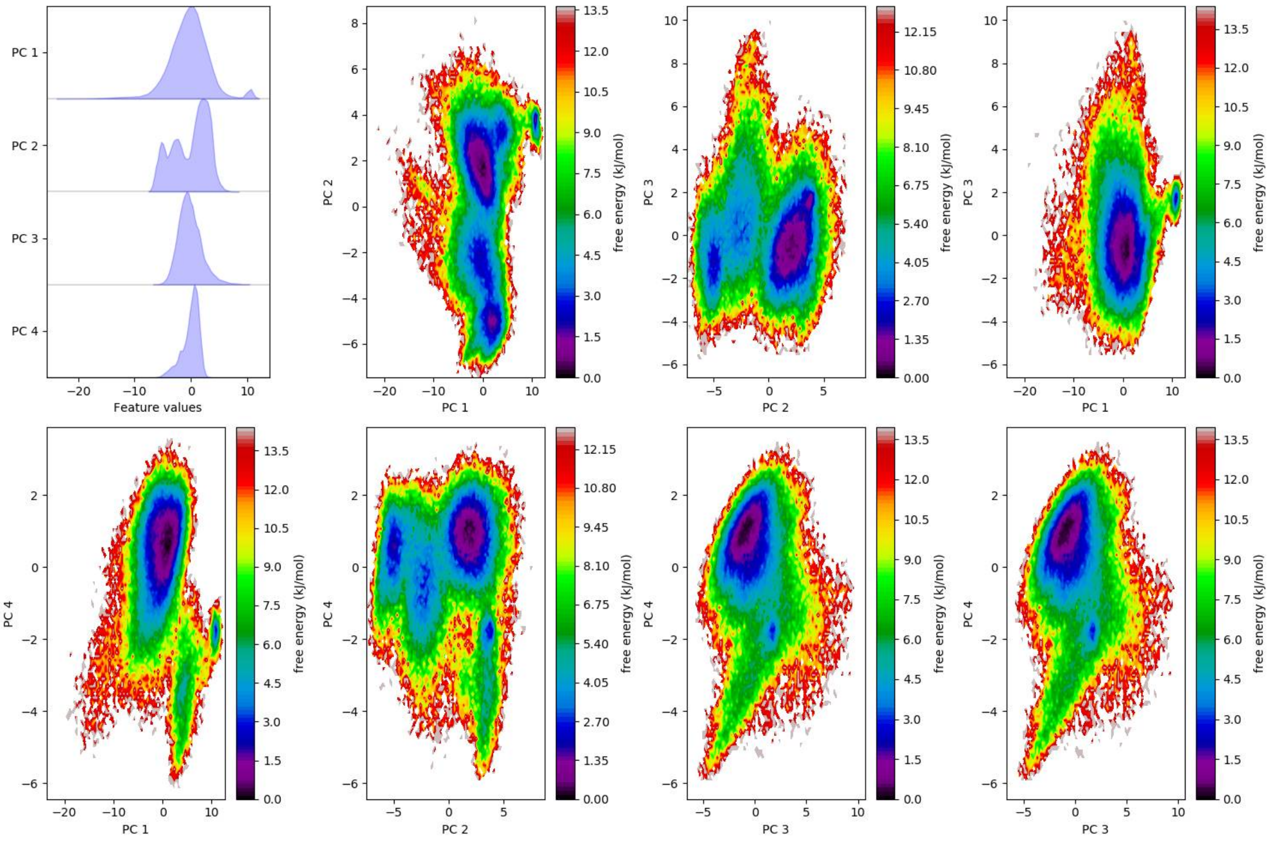

Figure S70 PCA projections between 1-4

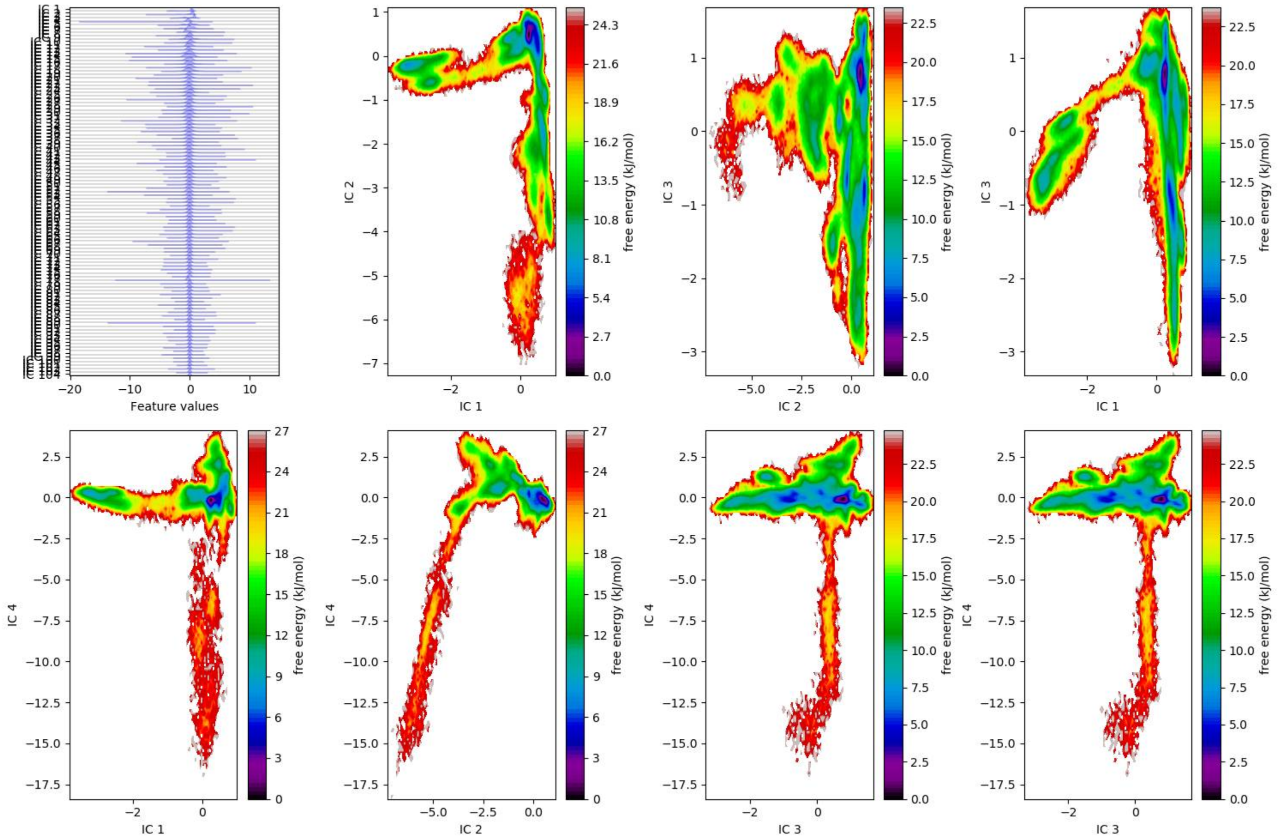

Figure S71 TICA projections between 1-4

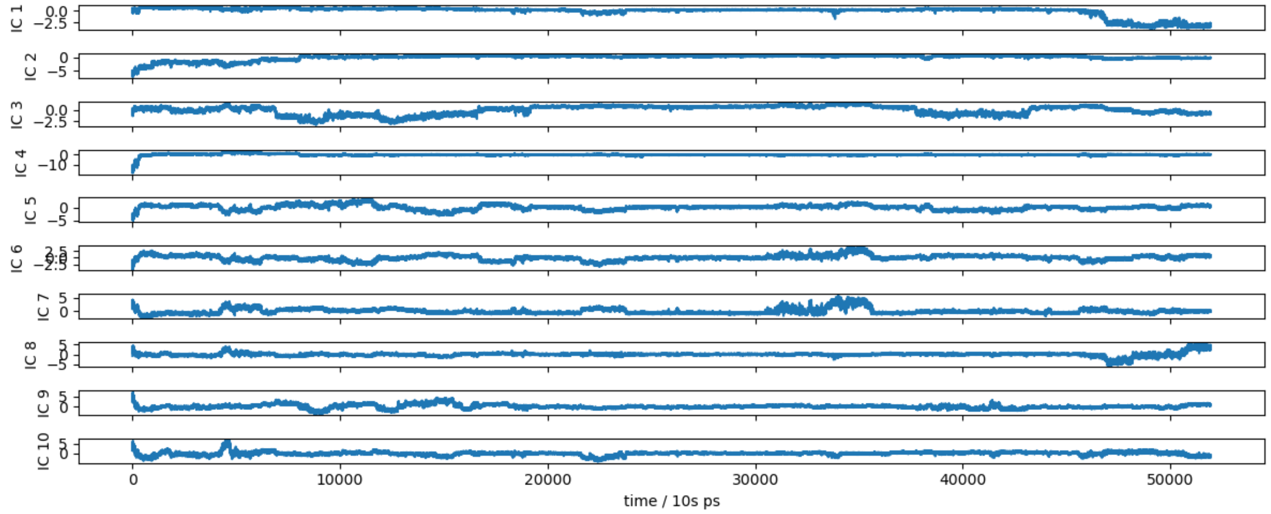

Figure S72 First 10 IC values over the simulation time

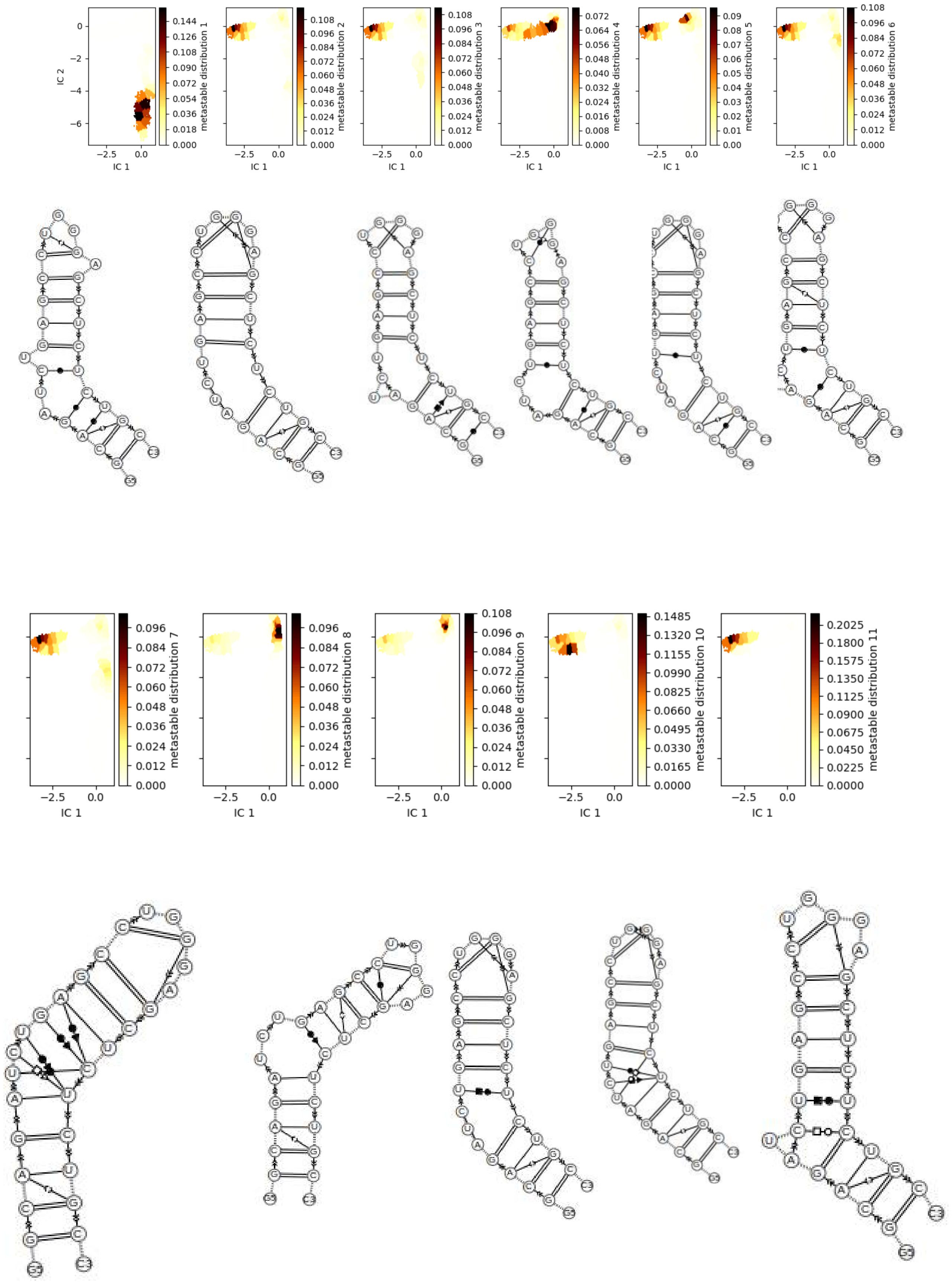

Figure S73 Regions of the TICA landscape and corresponding structure

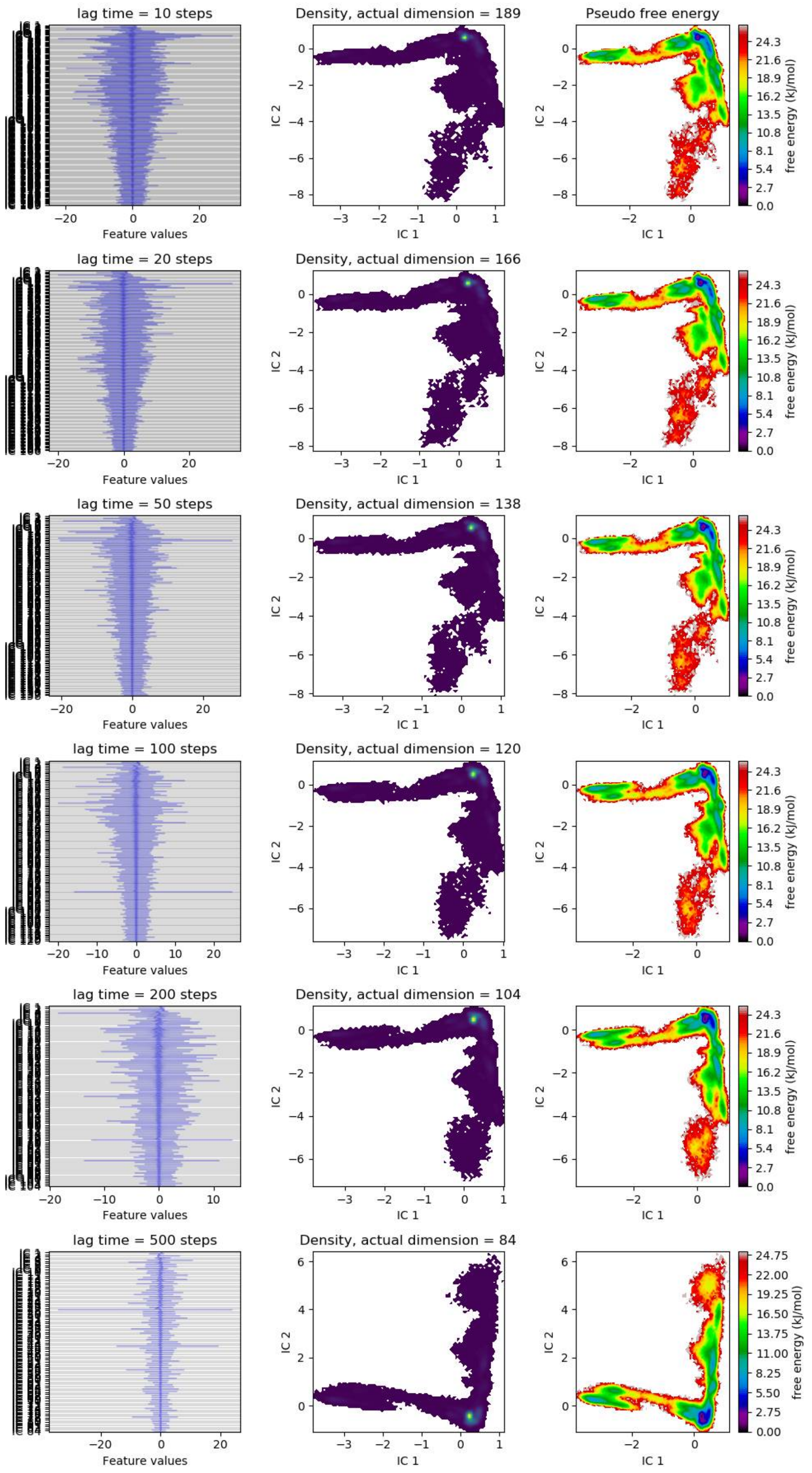

Figure S74 TICA dimensions, density and corrected heat map for steps 10 to 500

1ANR 3

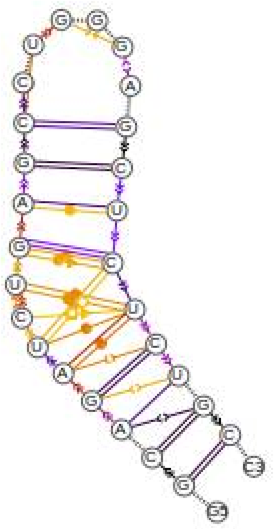

Figure S75 simulation starting from 3 solution of 1ANR

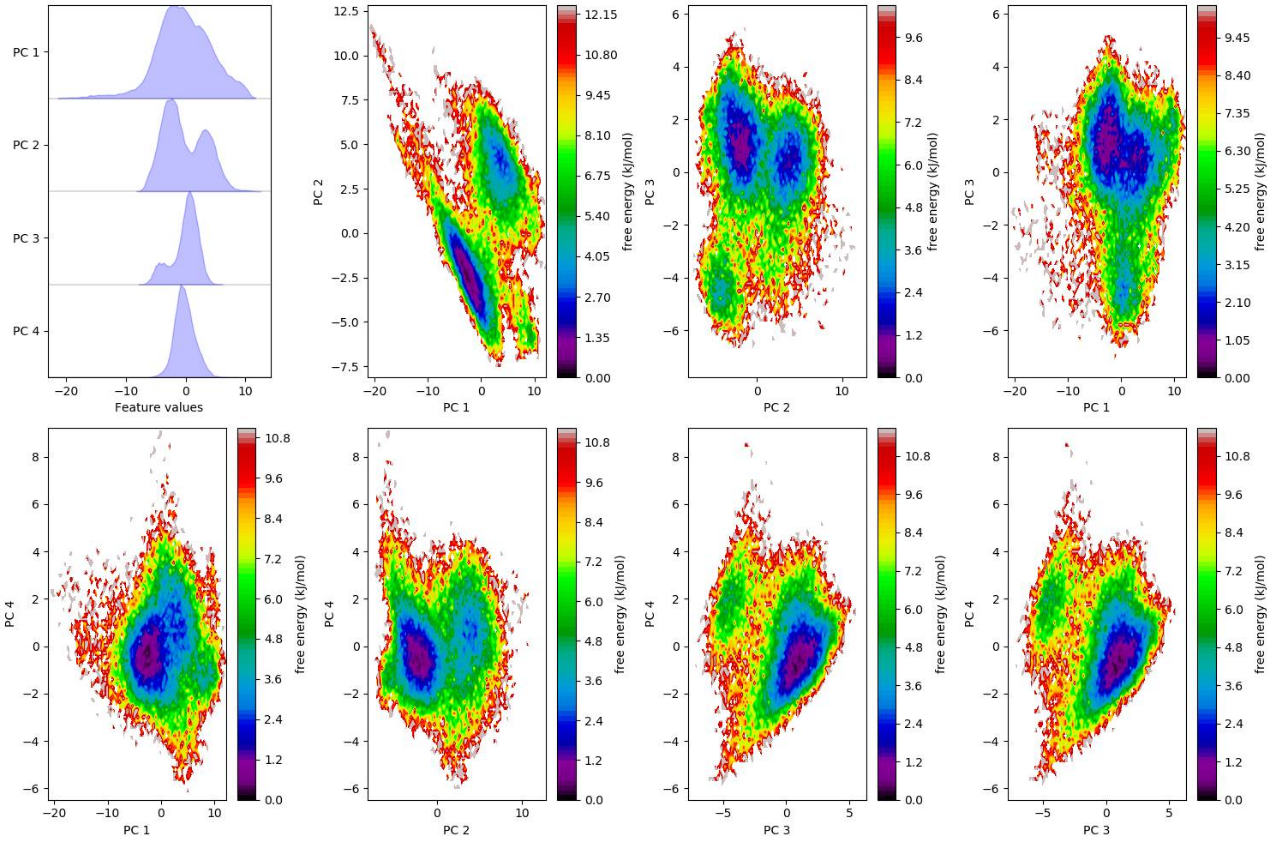

Figure S76 PCA projections between 1-4

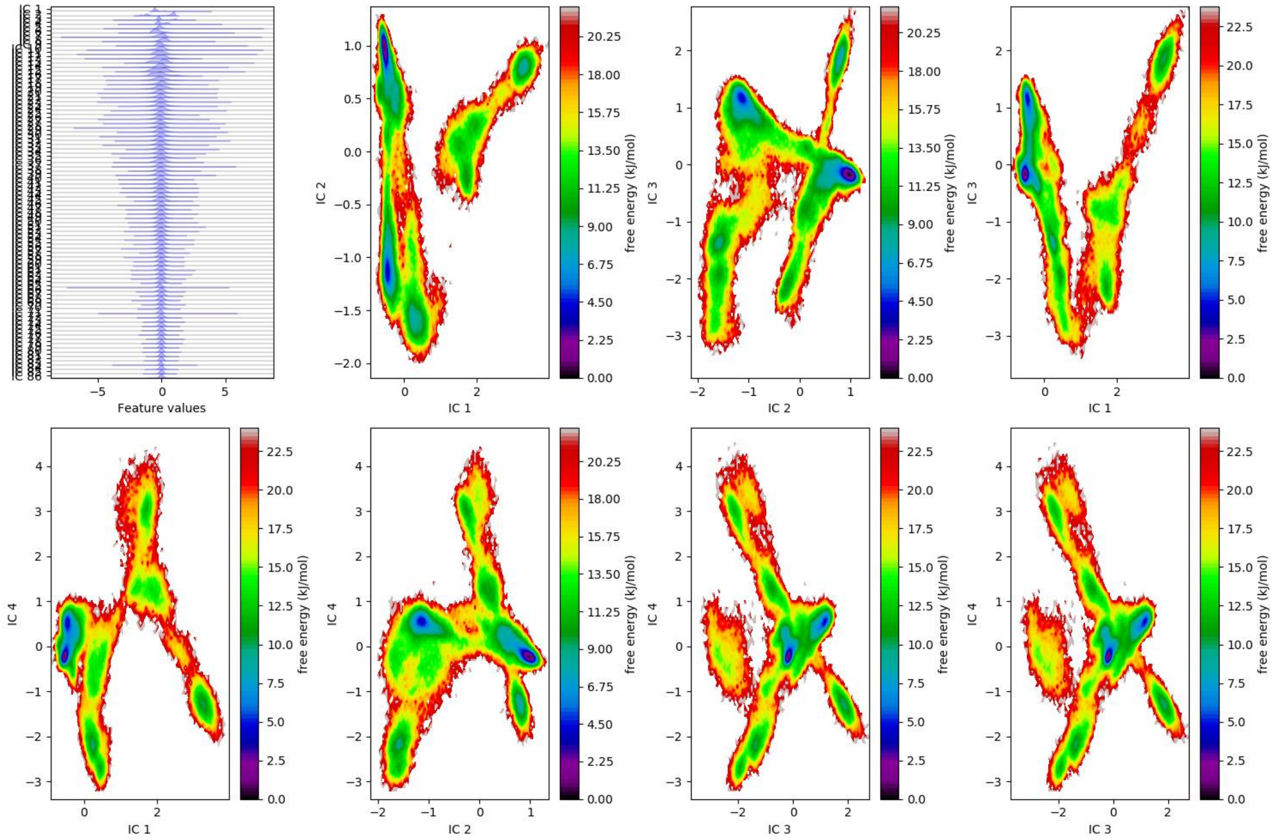

Figure S77 TICA projections between 1-4

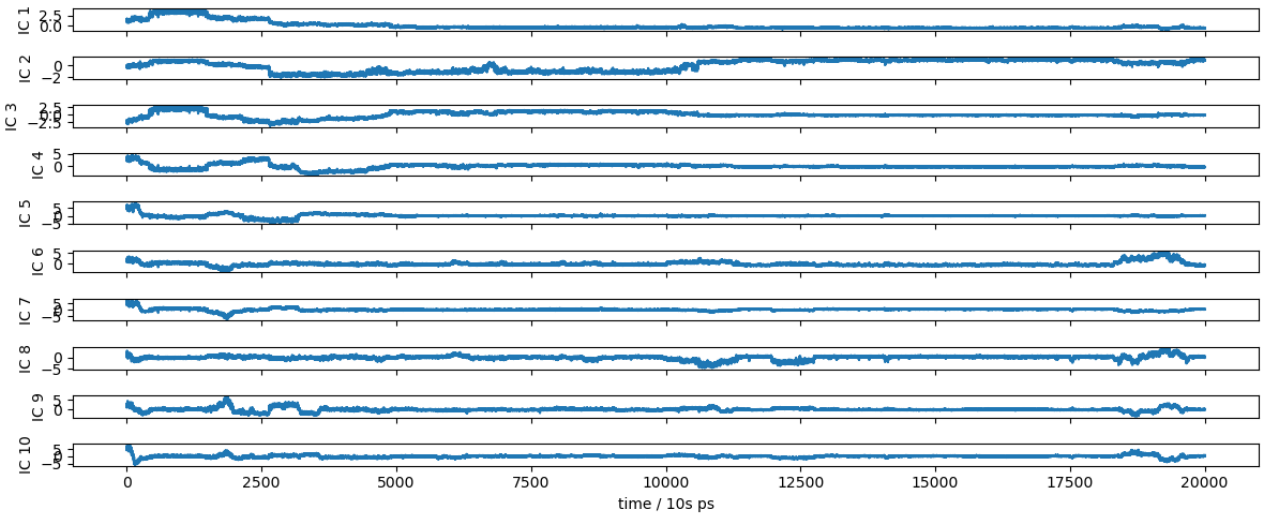

Figure S78 First 10 IC values over the simulation time

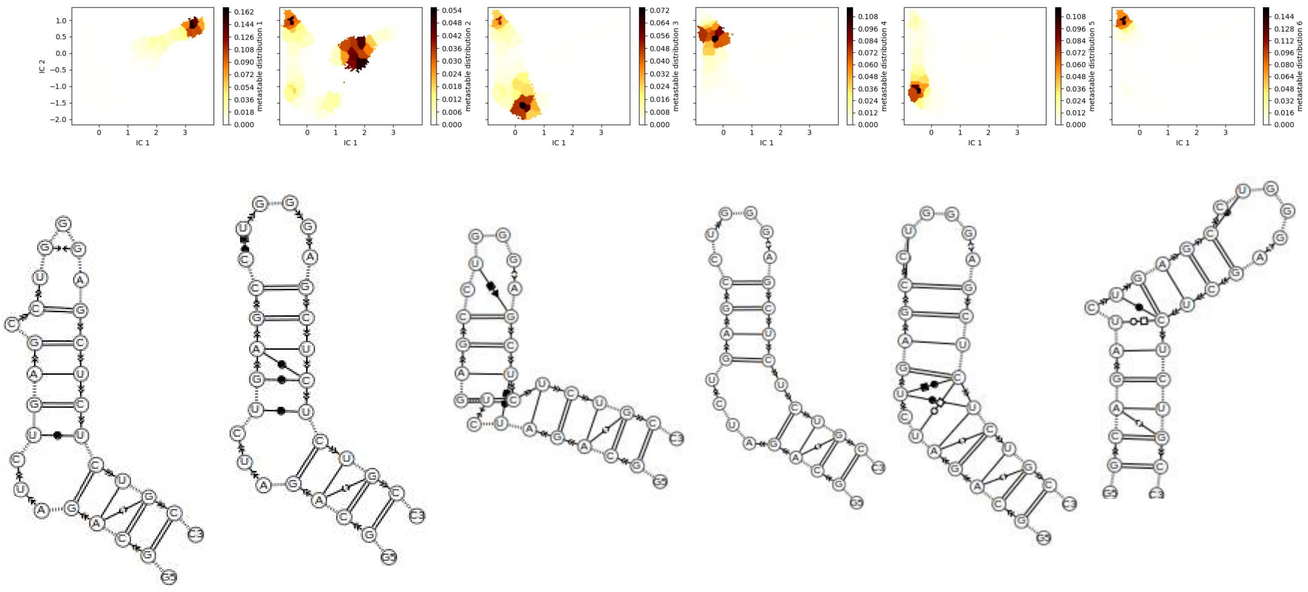

Figure S79 Regions of the TICA landscape and corresponding structure

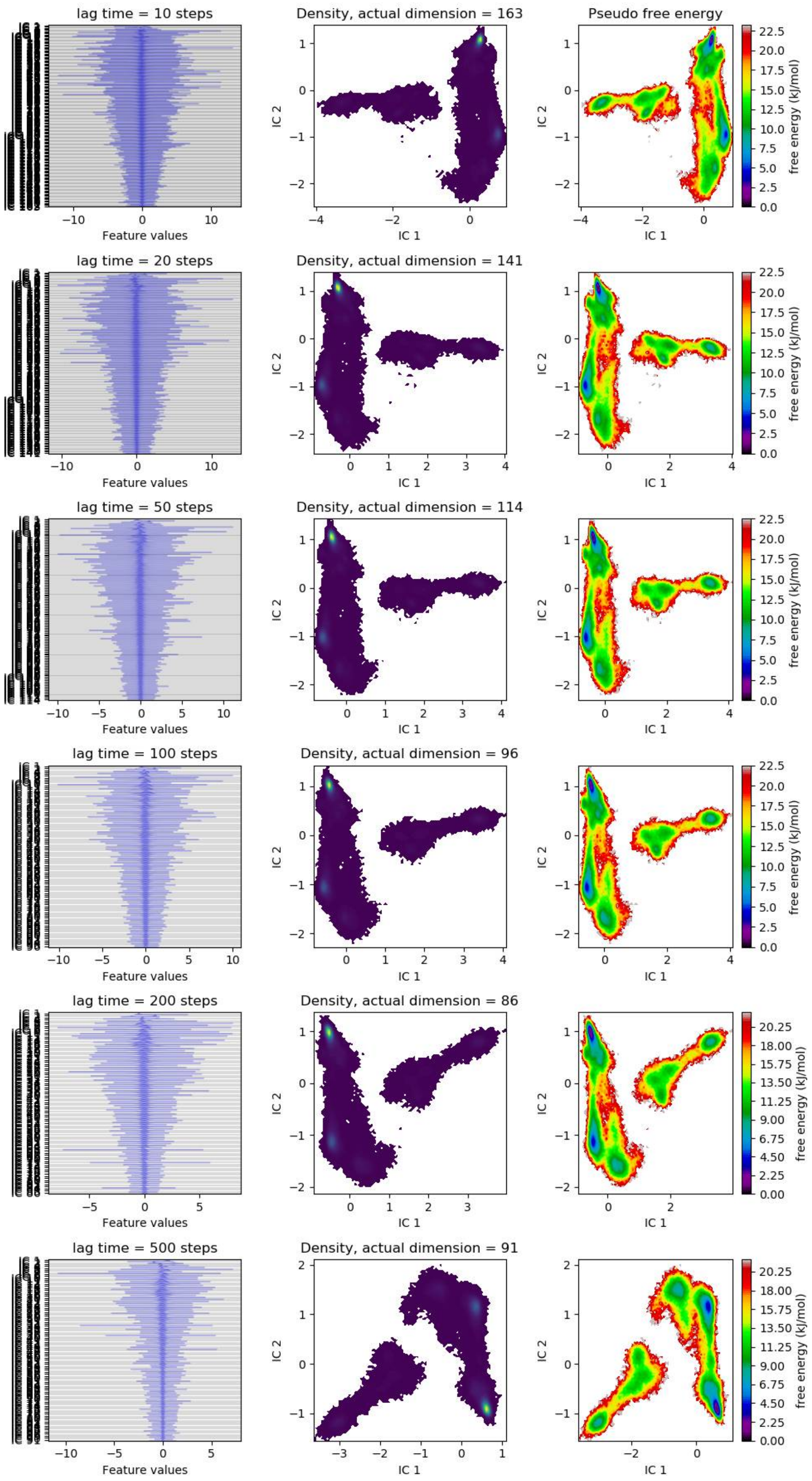

Figure S80 TICA dimensions, density and corrected heat map for steps 10 to 500

1ANR 4

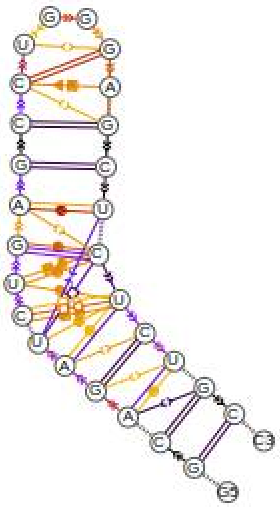

Figure S81 simulation starting from 4 solution of 1ANR

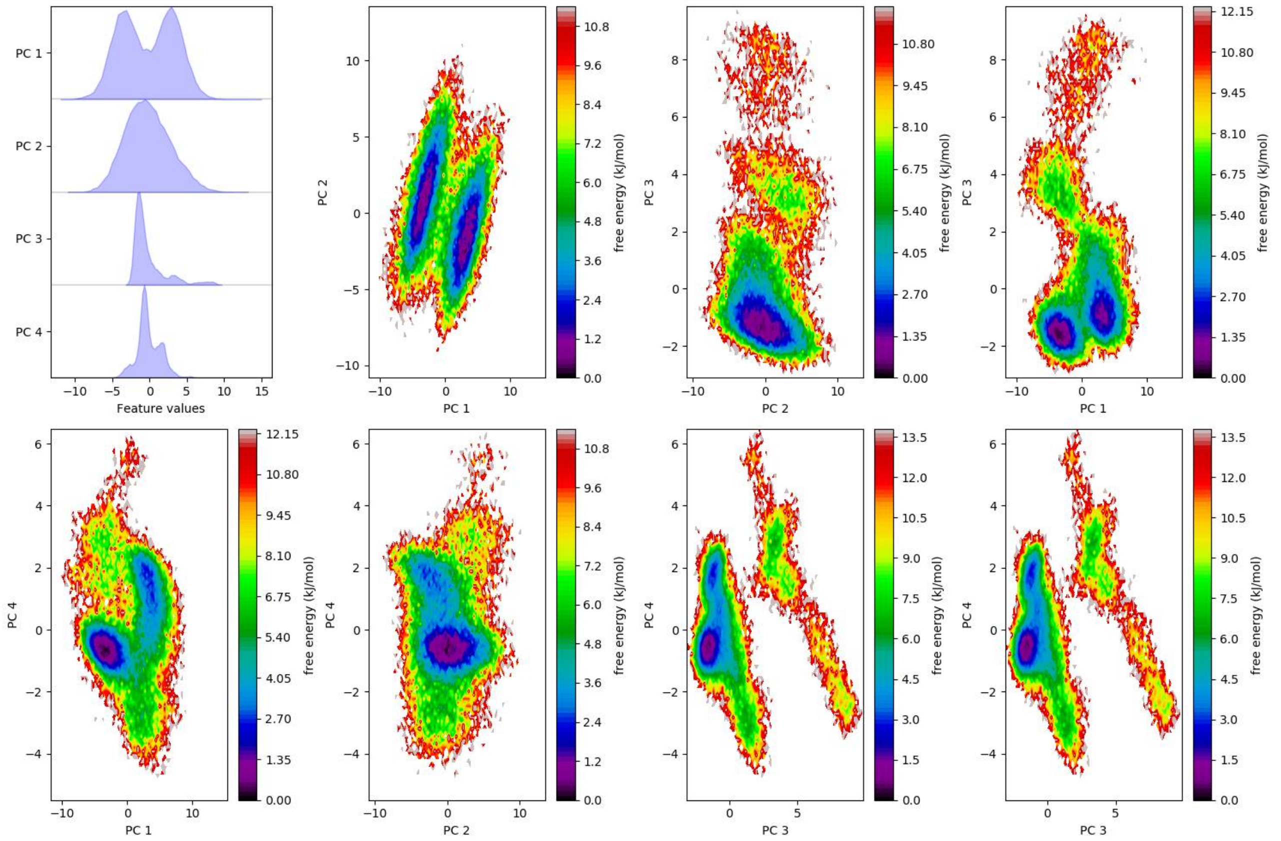

Figure S82 PCA projections between 1-4

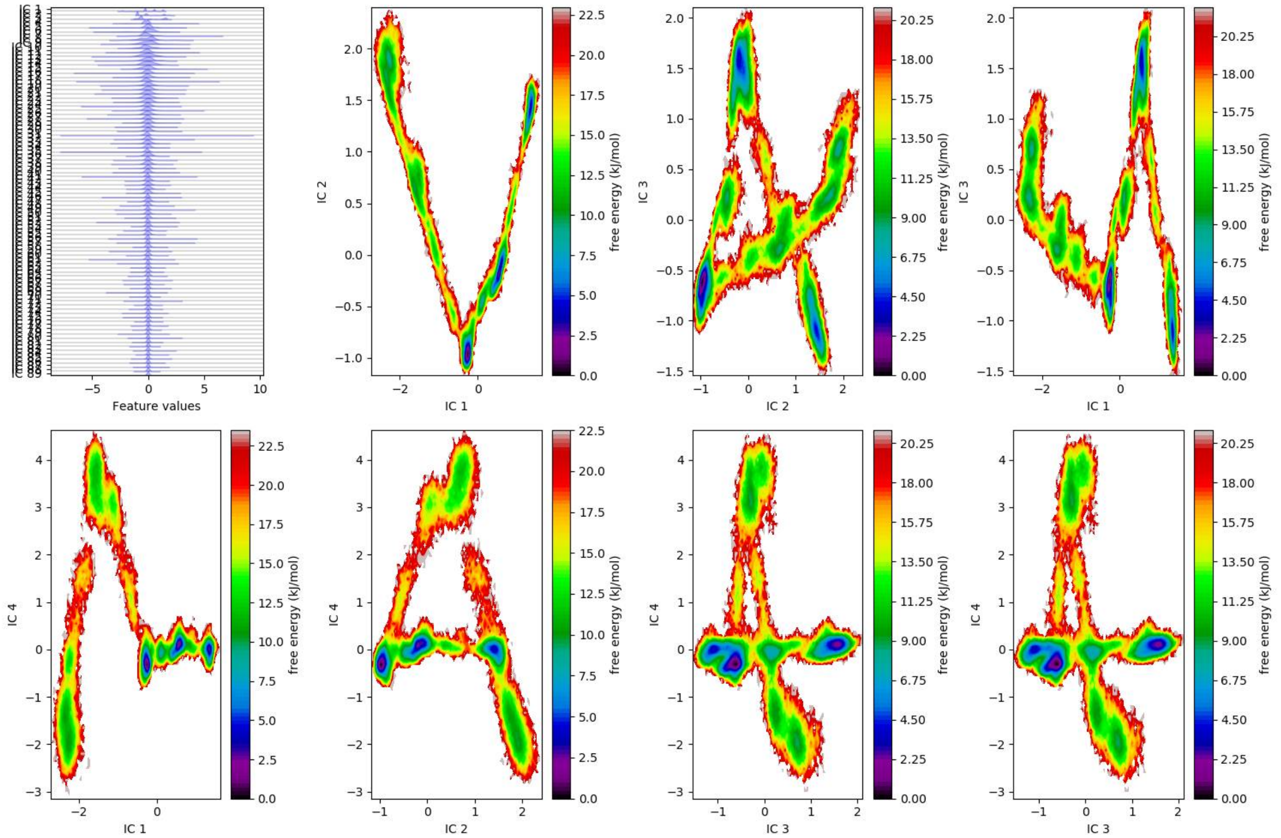

Figure S83 TICA projections between 1-4

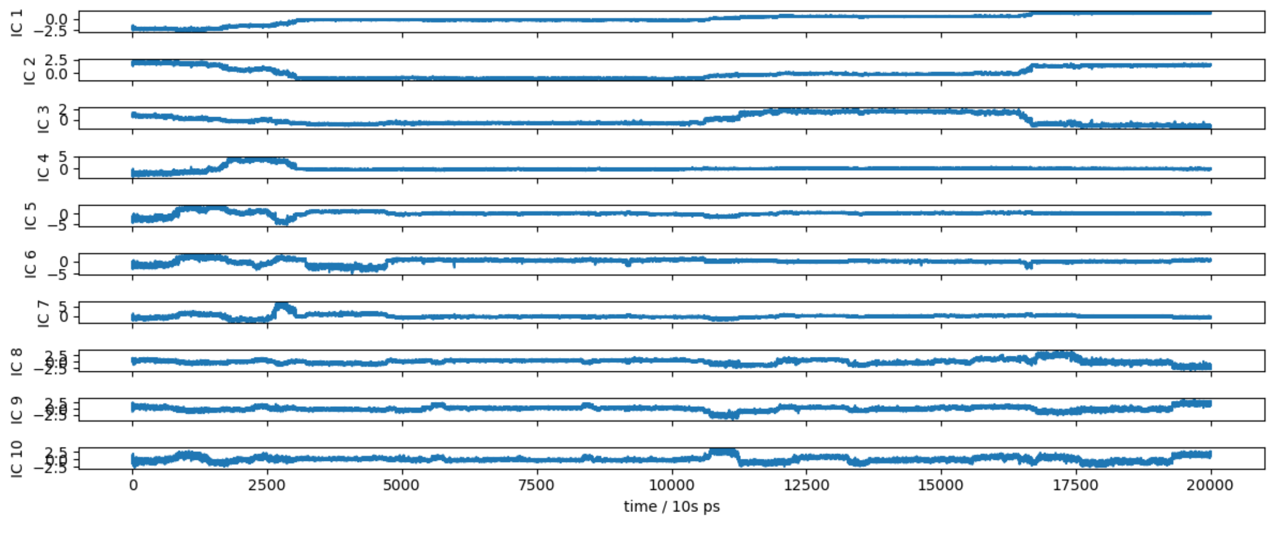

Figure S84 First 10 IC values over the simulation time

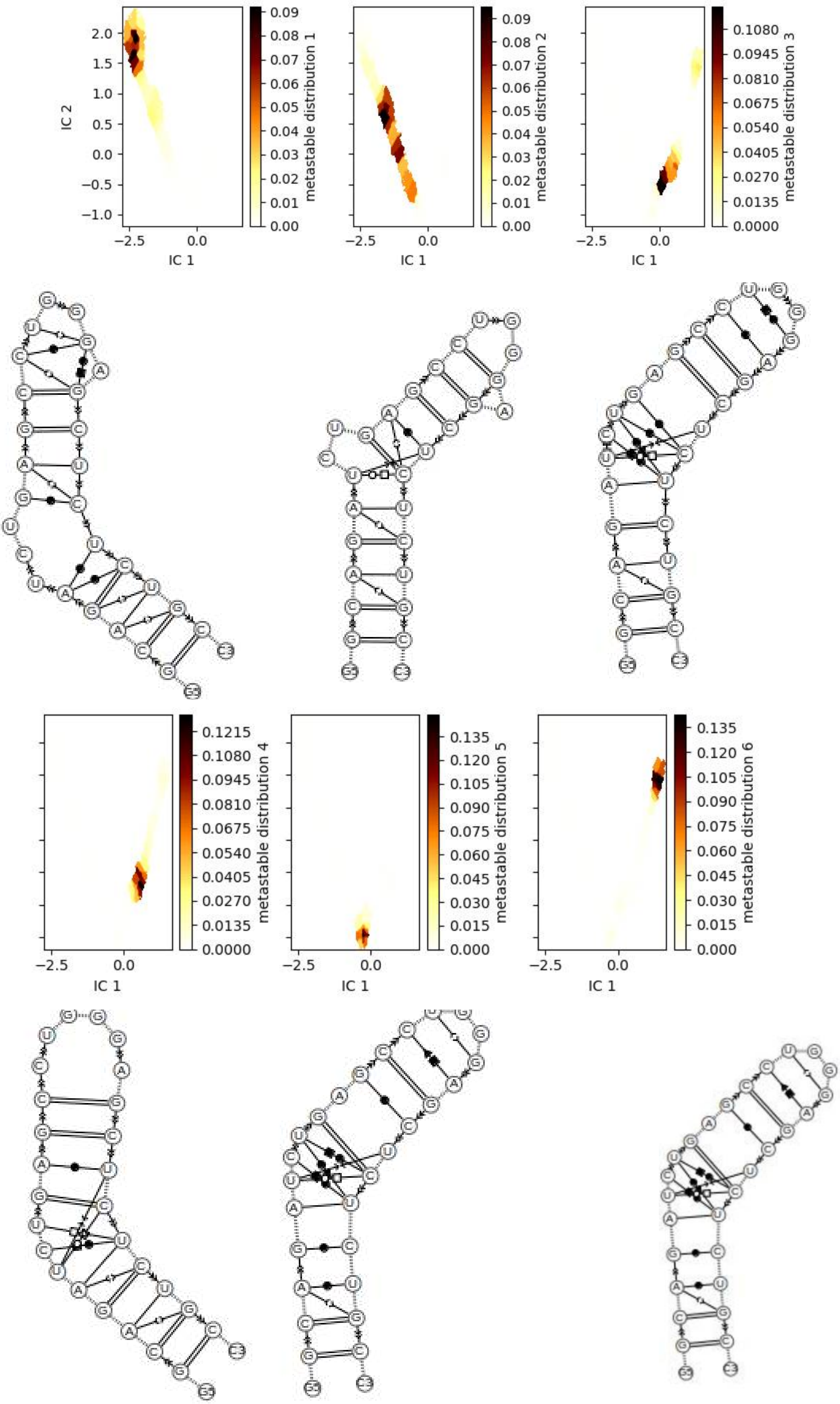

Figure S85 Regions of the TICA landscape and corresponding structure

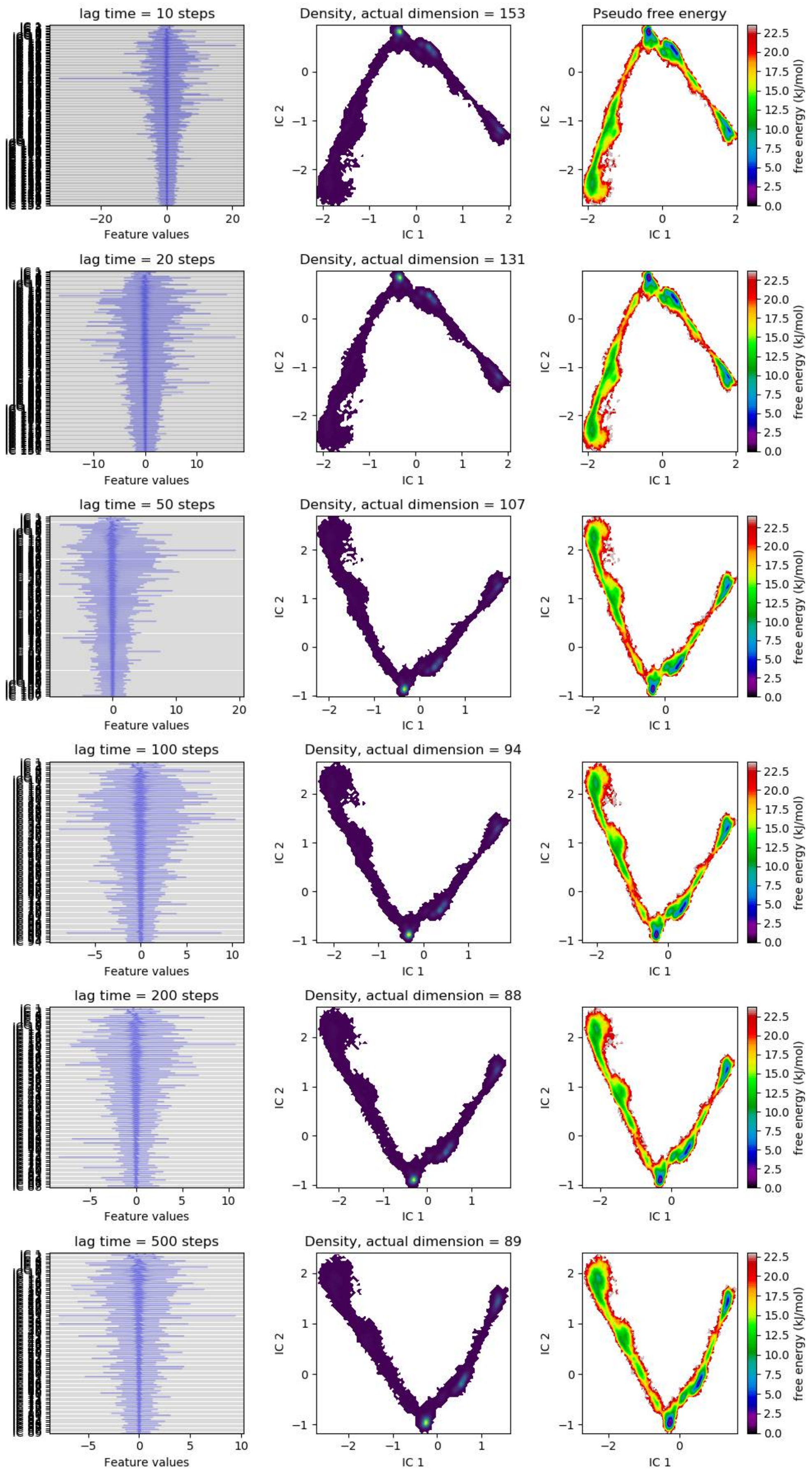

Figure S86 TICA dimensions, density and corrected heat map for steps 10 to 500

1ANR 7

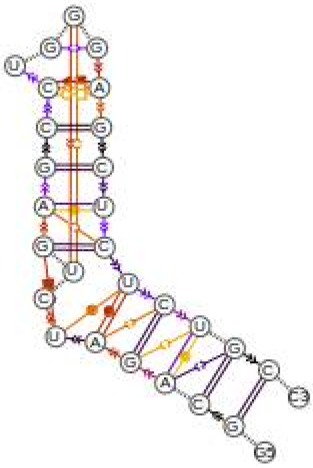

Figure S87 Simulation starting from 7 solution of 1ANR

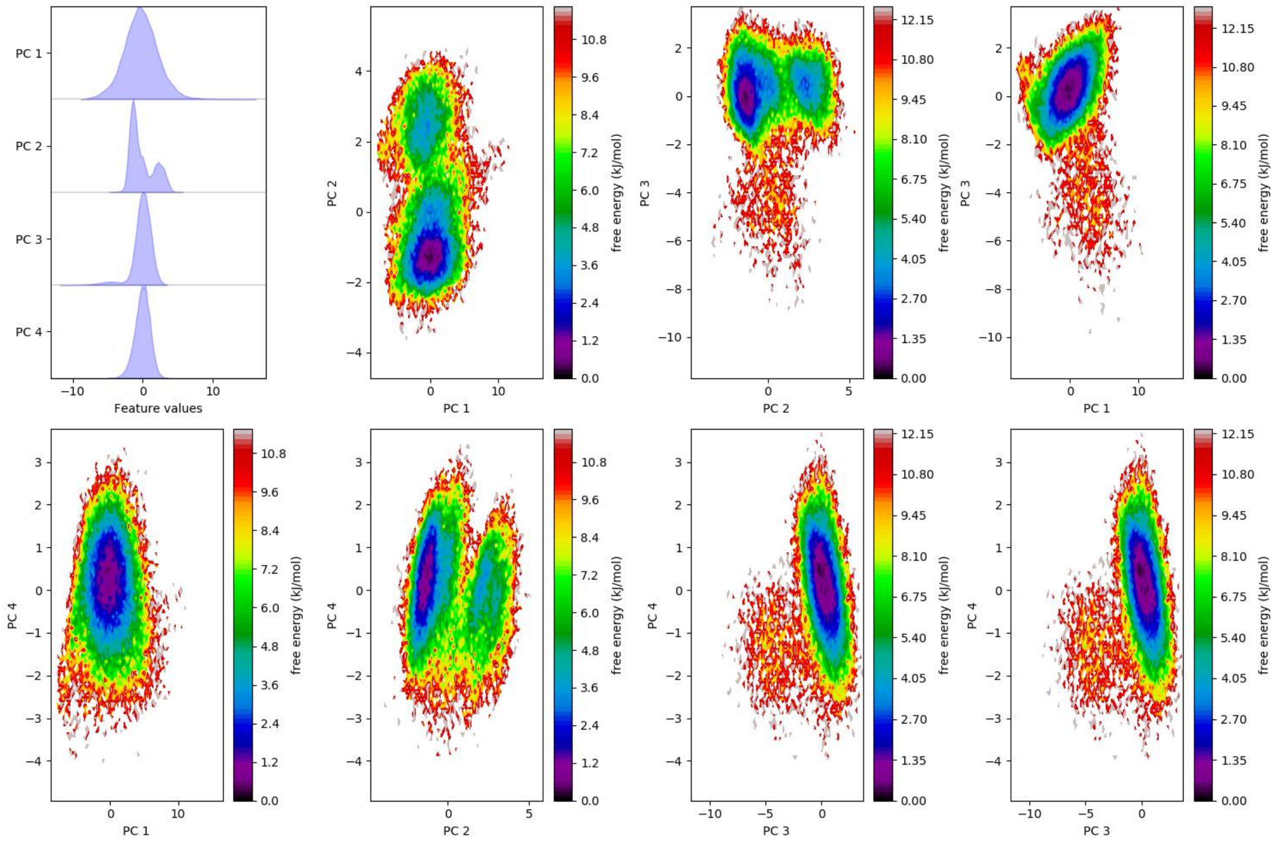

Figure S88 PCA projections between 1-4

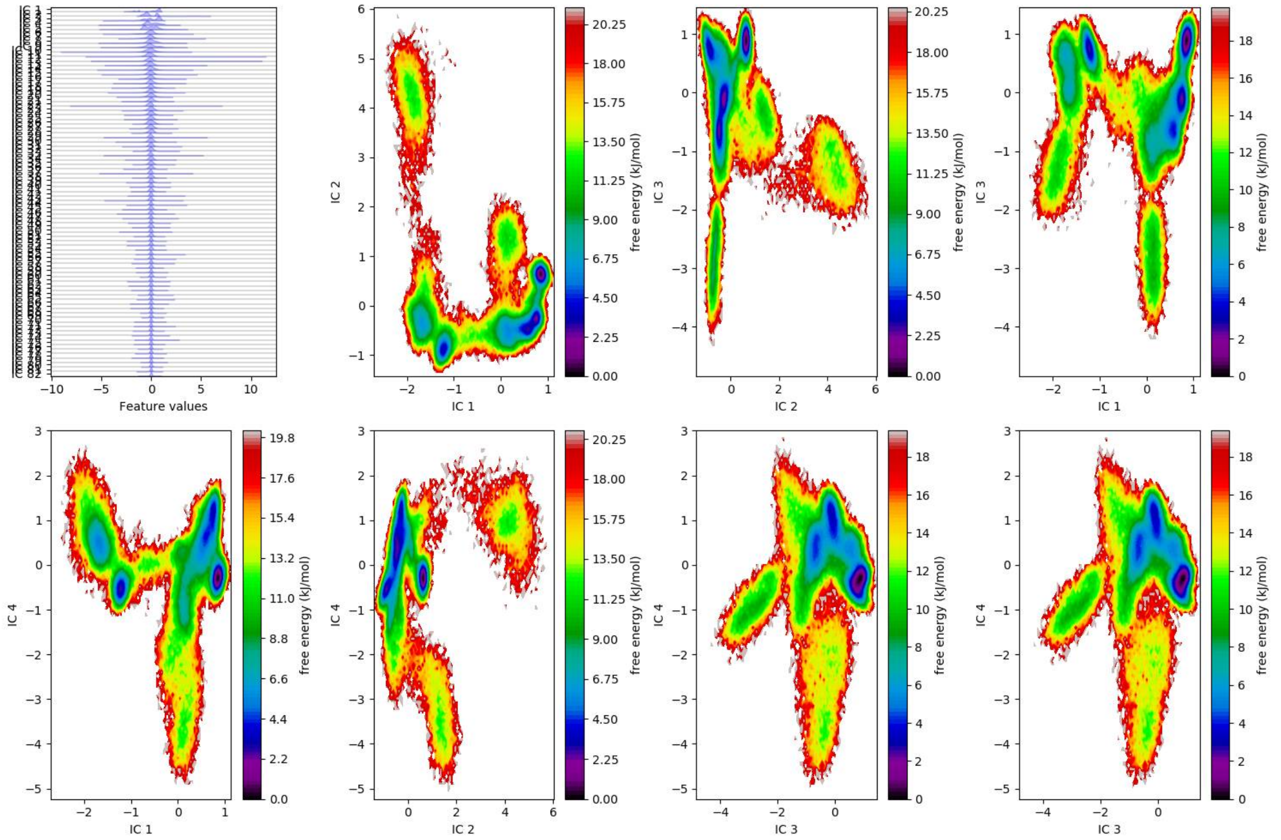

Figure S89 TICA projections between 1-4

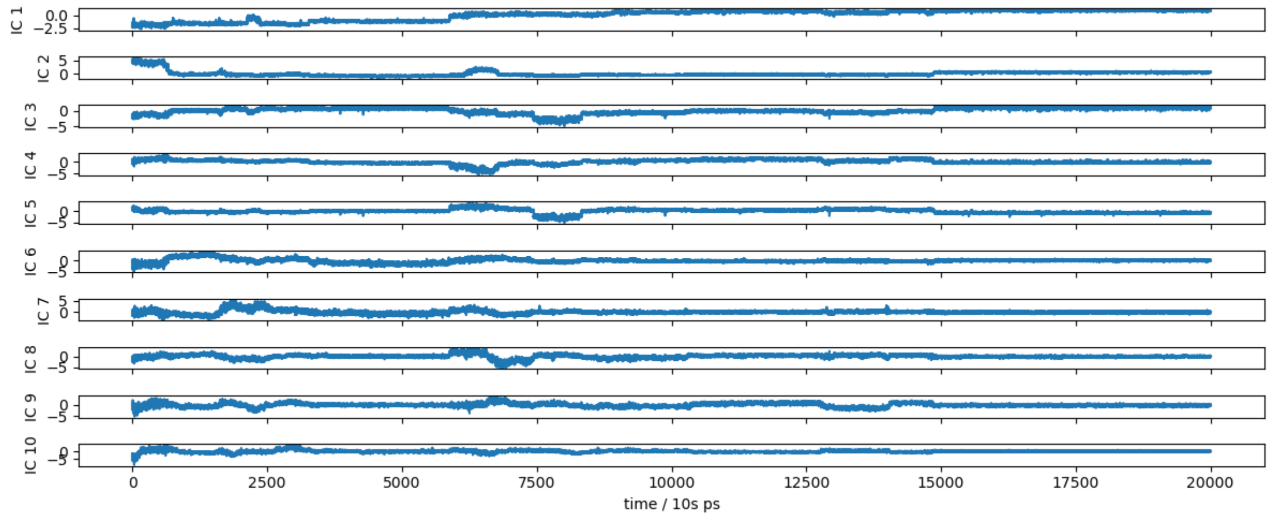

Figure S90 First 10 IC values over the simulation time

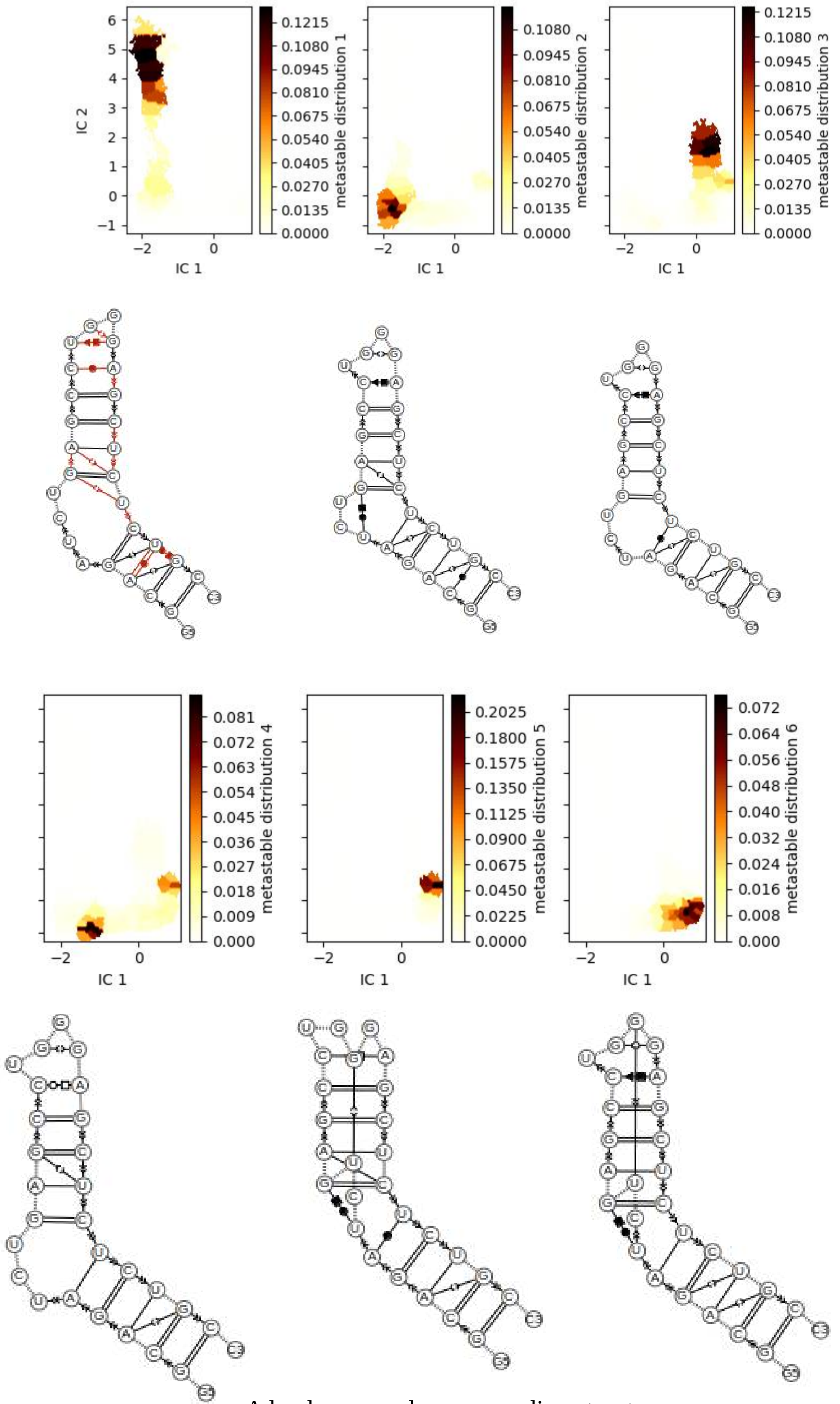

Figure [illegible] Regions of the TICA landscape and corresponding structure

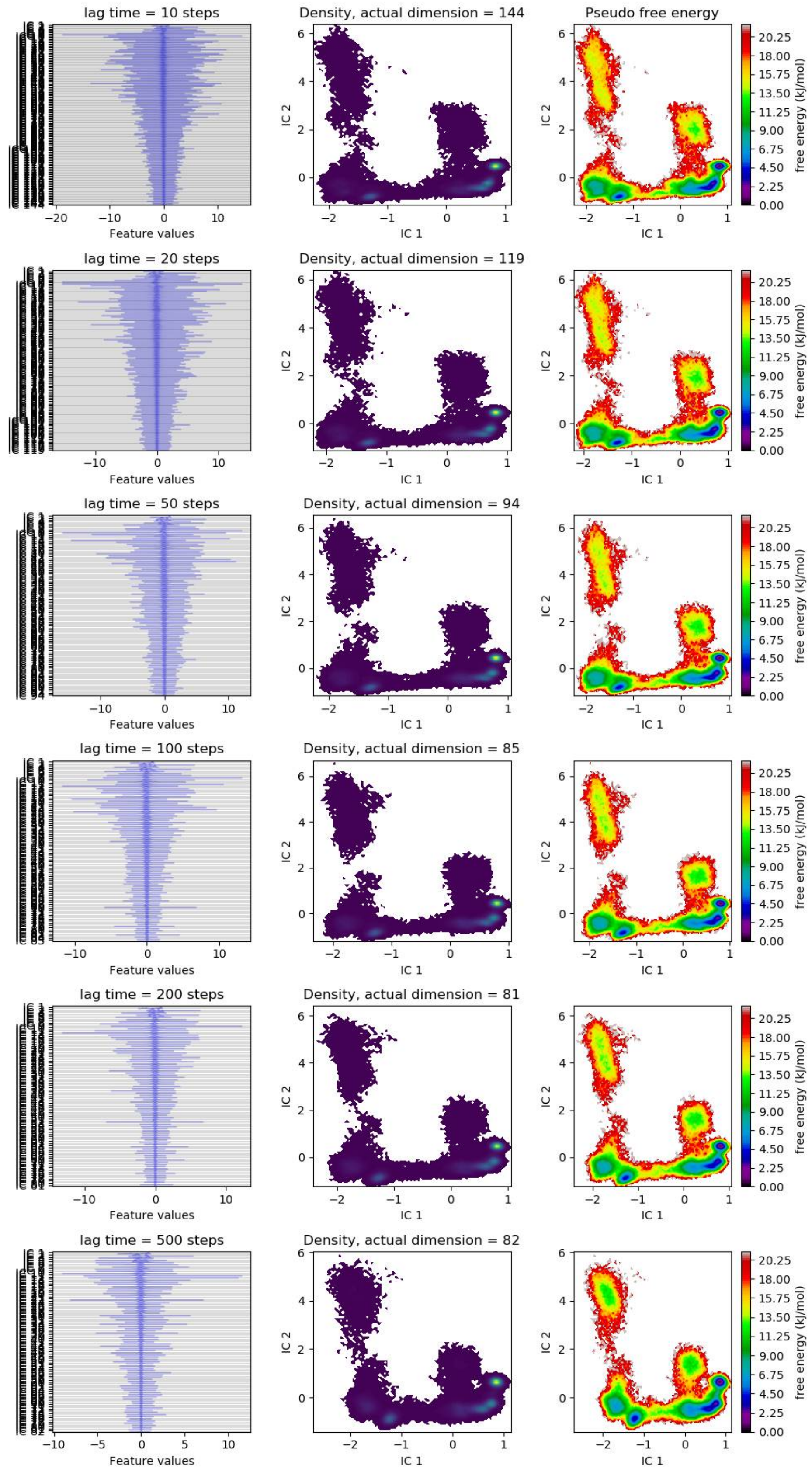

Figure S92 TICA dimensions, density and corrected heat map for steps 10 to 500

1ANR 10

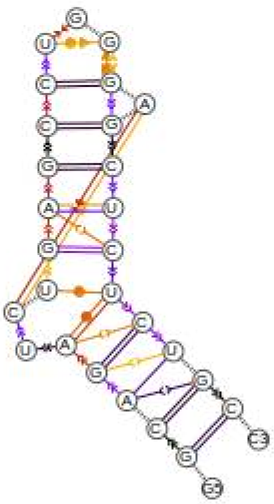

Figure S93 Simulation starting from 10 solution of 1ANR

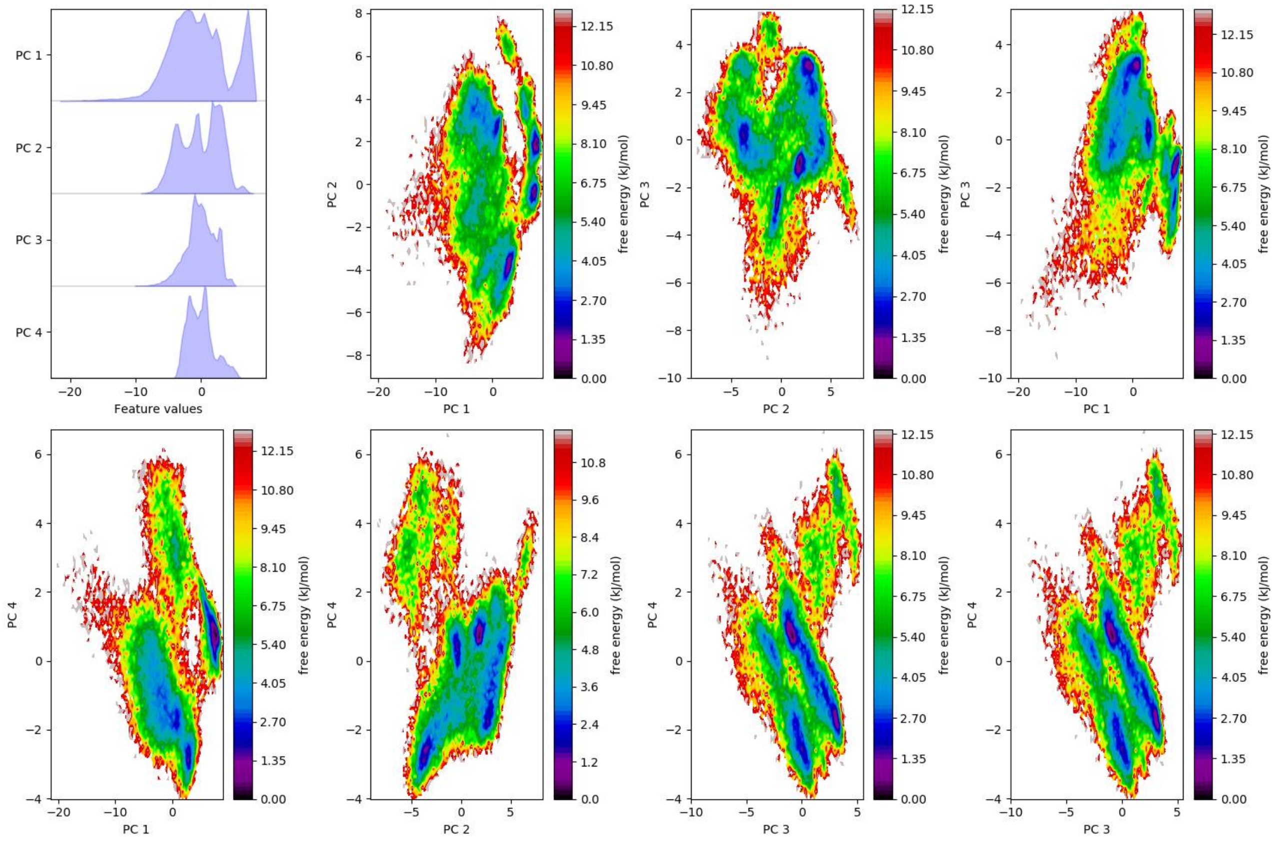

Figure S94 PCA projections between 1-4

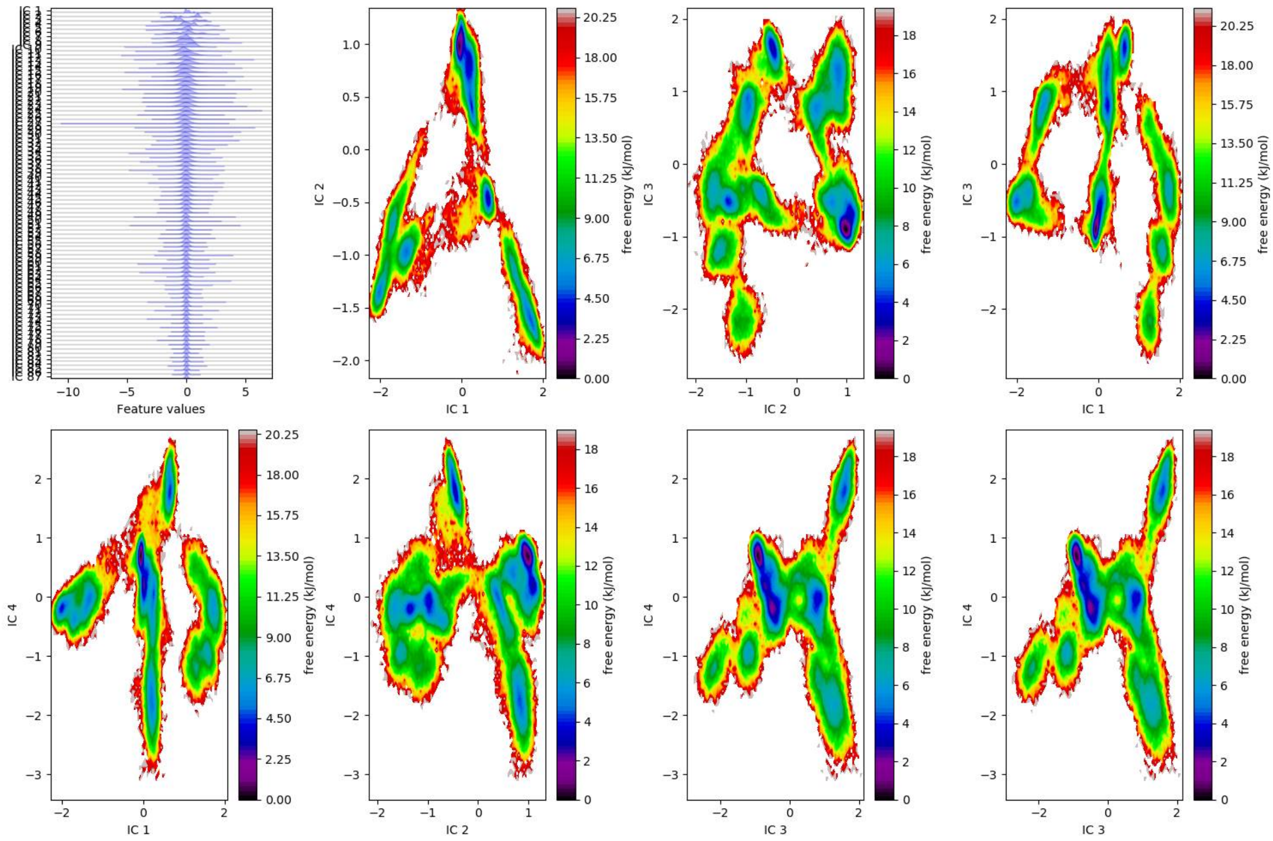

Figure S95 TICA projections between 1-4

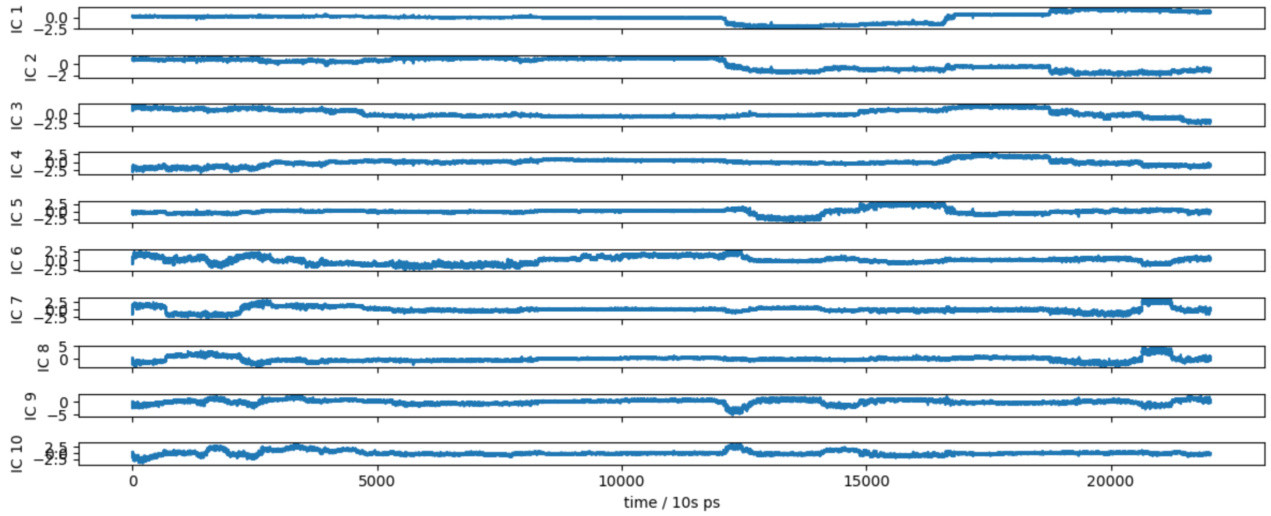

Figure S96 First 10 IC values over the simulation time

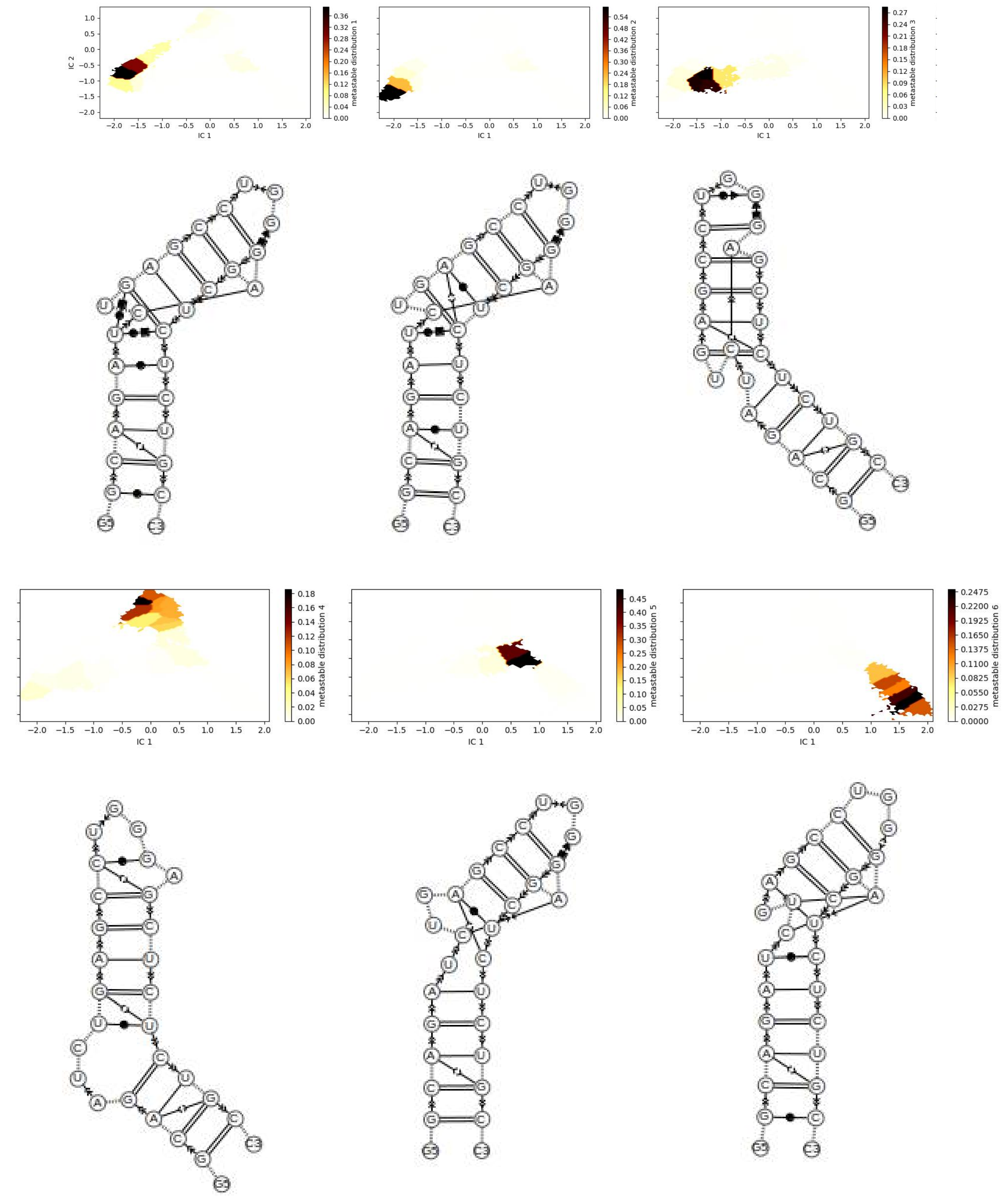

Figure S97 Regions of the TICA landscape and corresponding structure

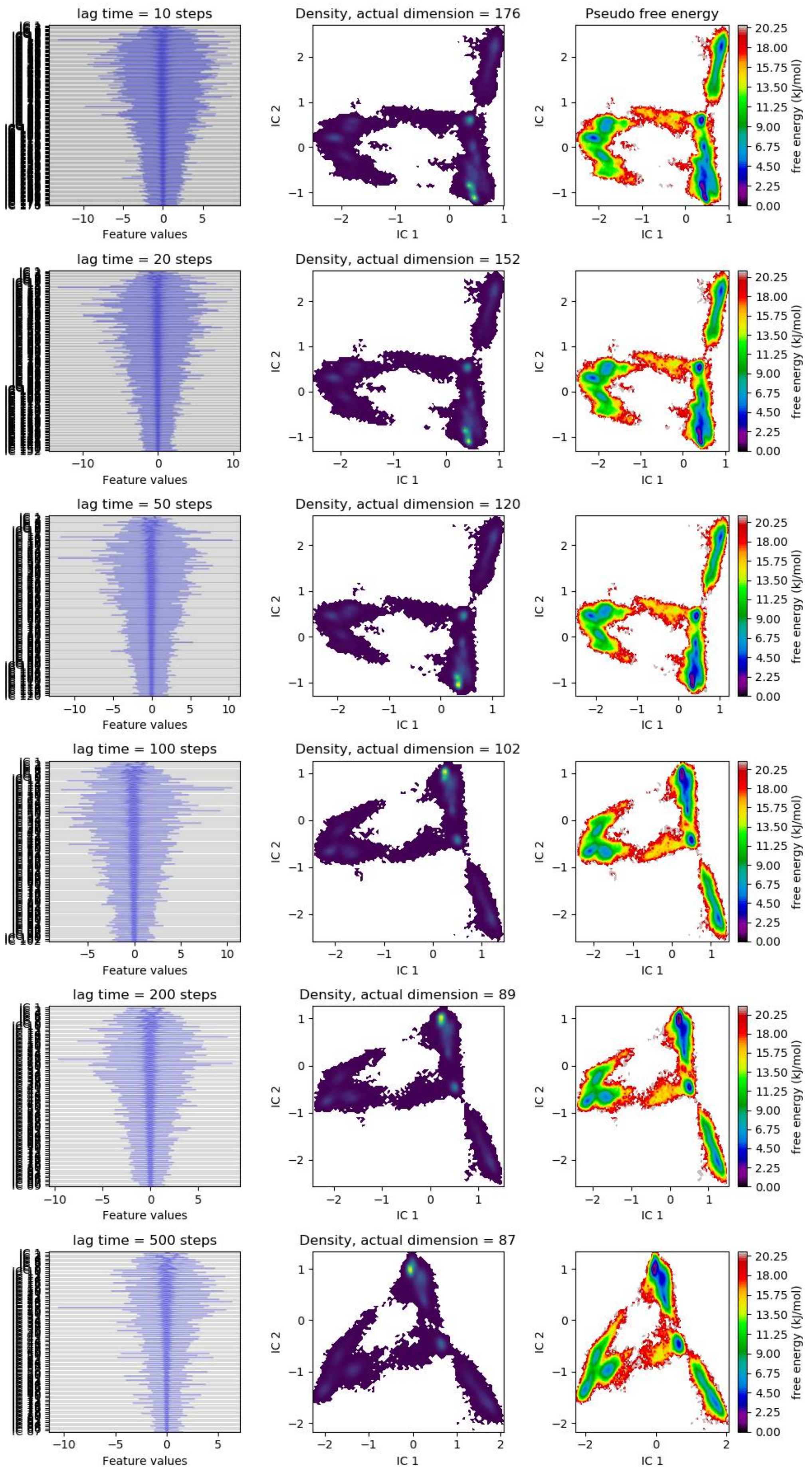

Figure S98 TICA dimensions, density and corrected heat map for steps 10 to 500

1ANR 12

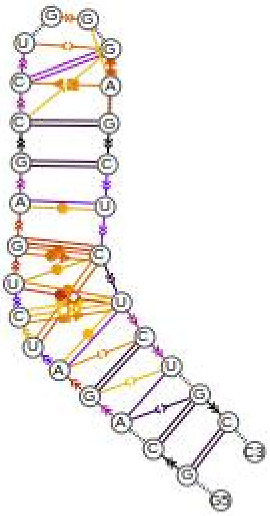

Figure S99 Simulation starting from 12 solution of 1ANR

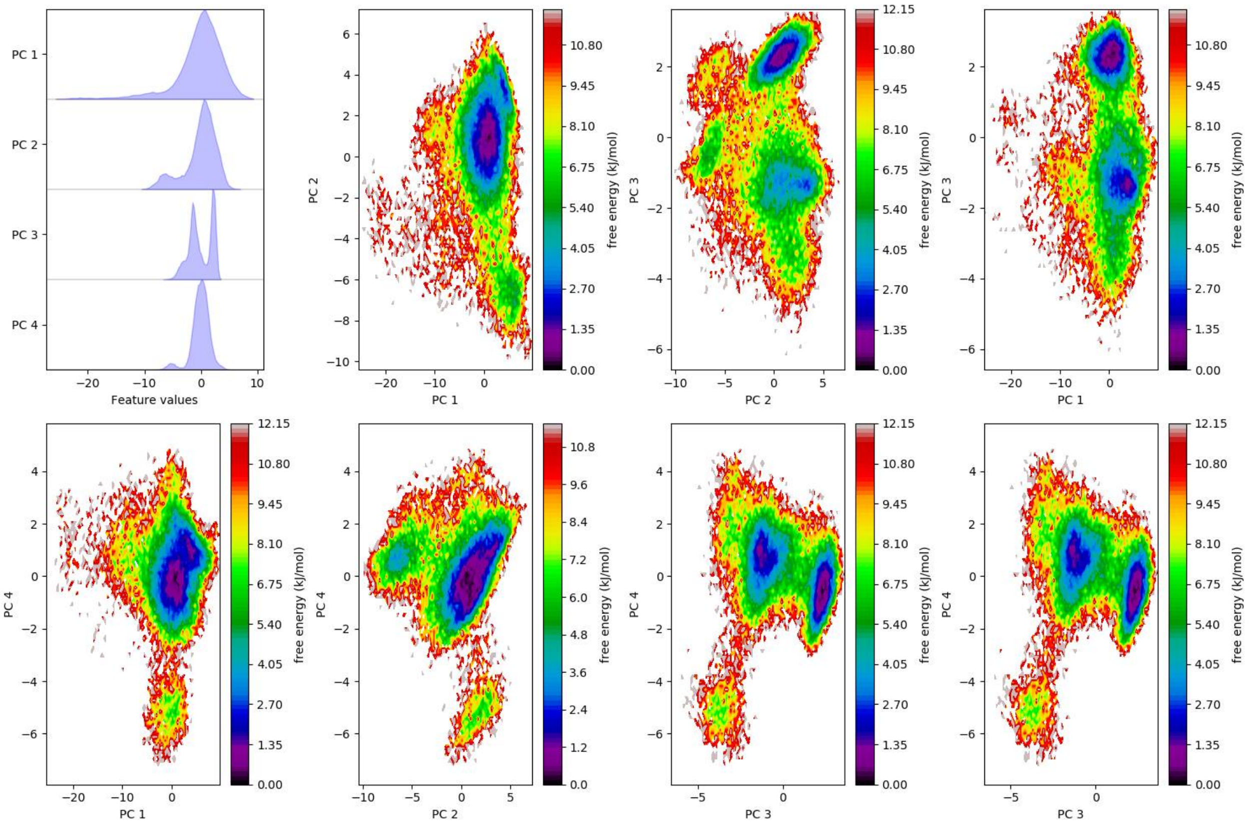

Figure S100 PCA projections between 1-4

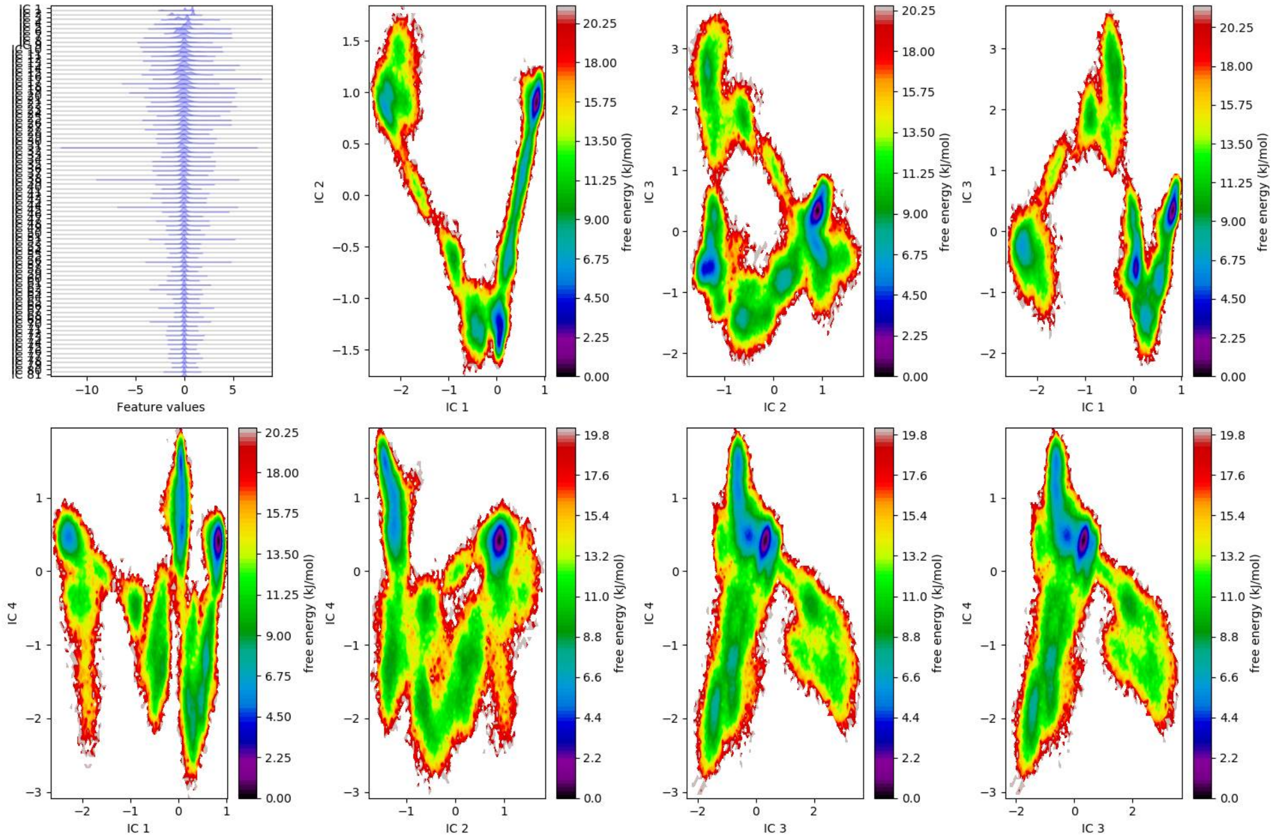

Figure S101 TICA projections between 1-4

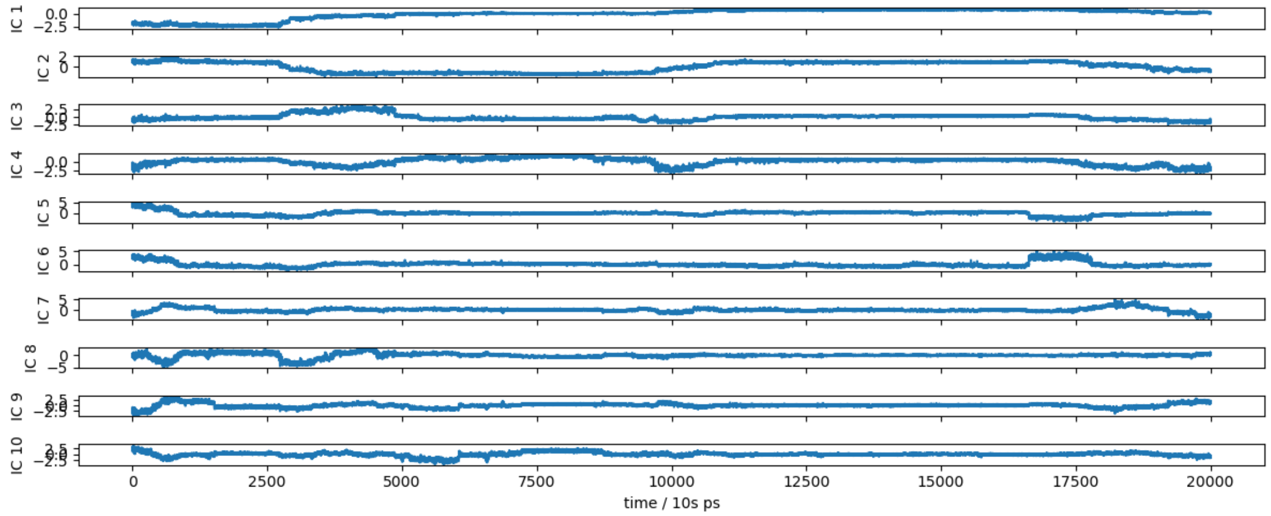

Figure S102 First 10 IC values over the simulation time

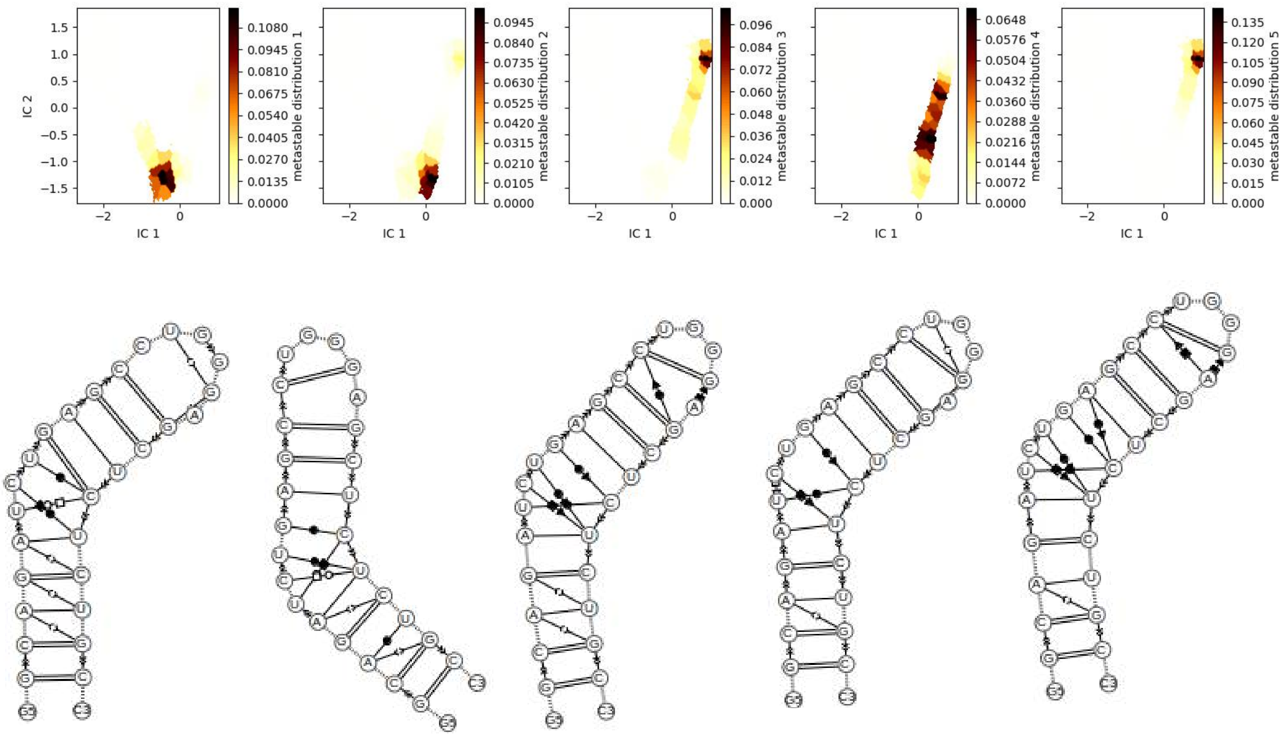

Figure S103 Regions of the TICA landscape and corresponding structure

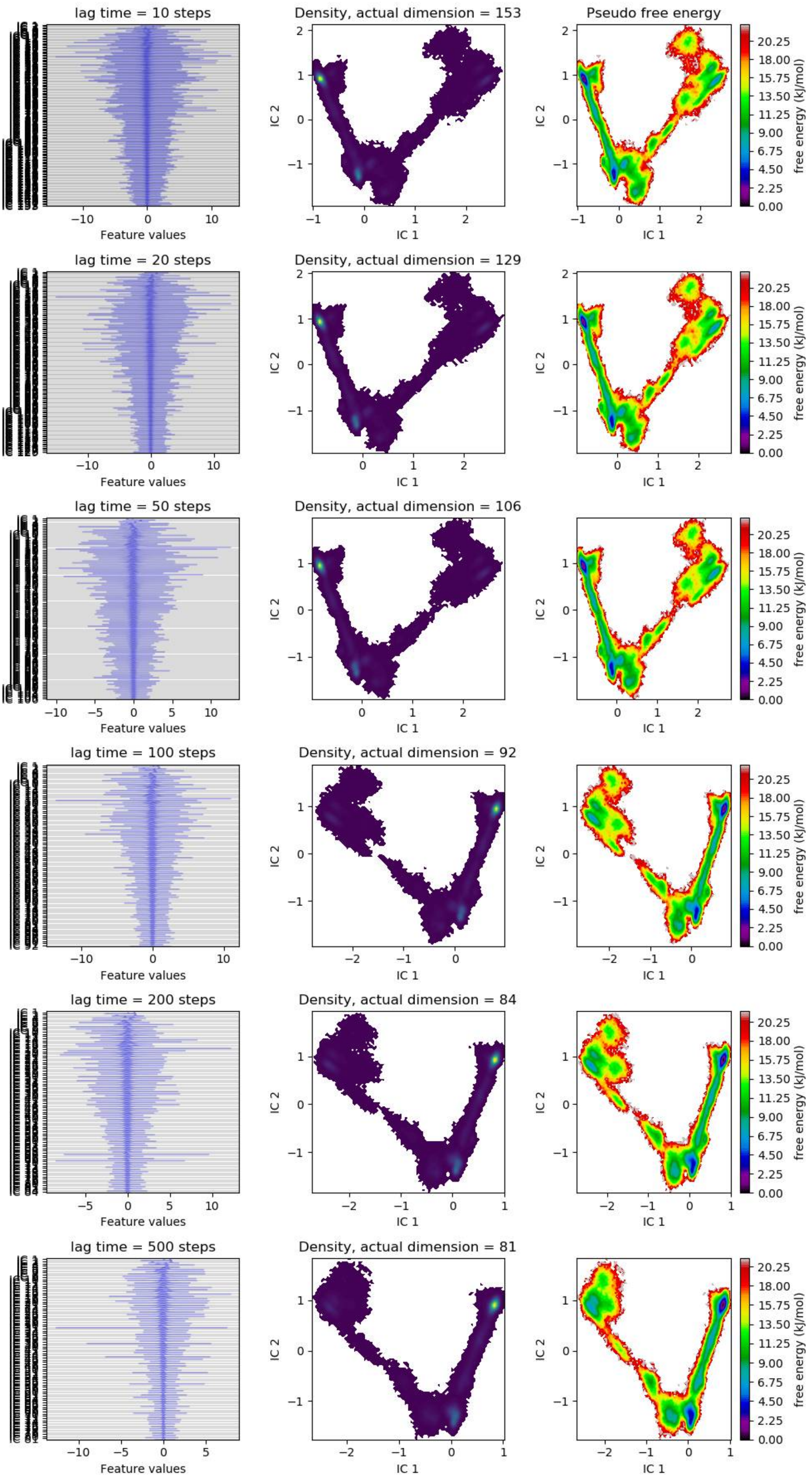

Figure S104 TICA dimensions, density and corrected heat map for steps 10 to 500

1ANR 16

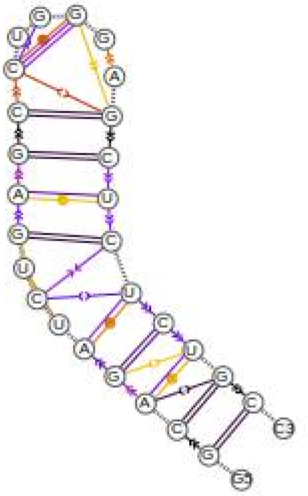

Figure S105 Simulation starting from 16 solution of 1ANR

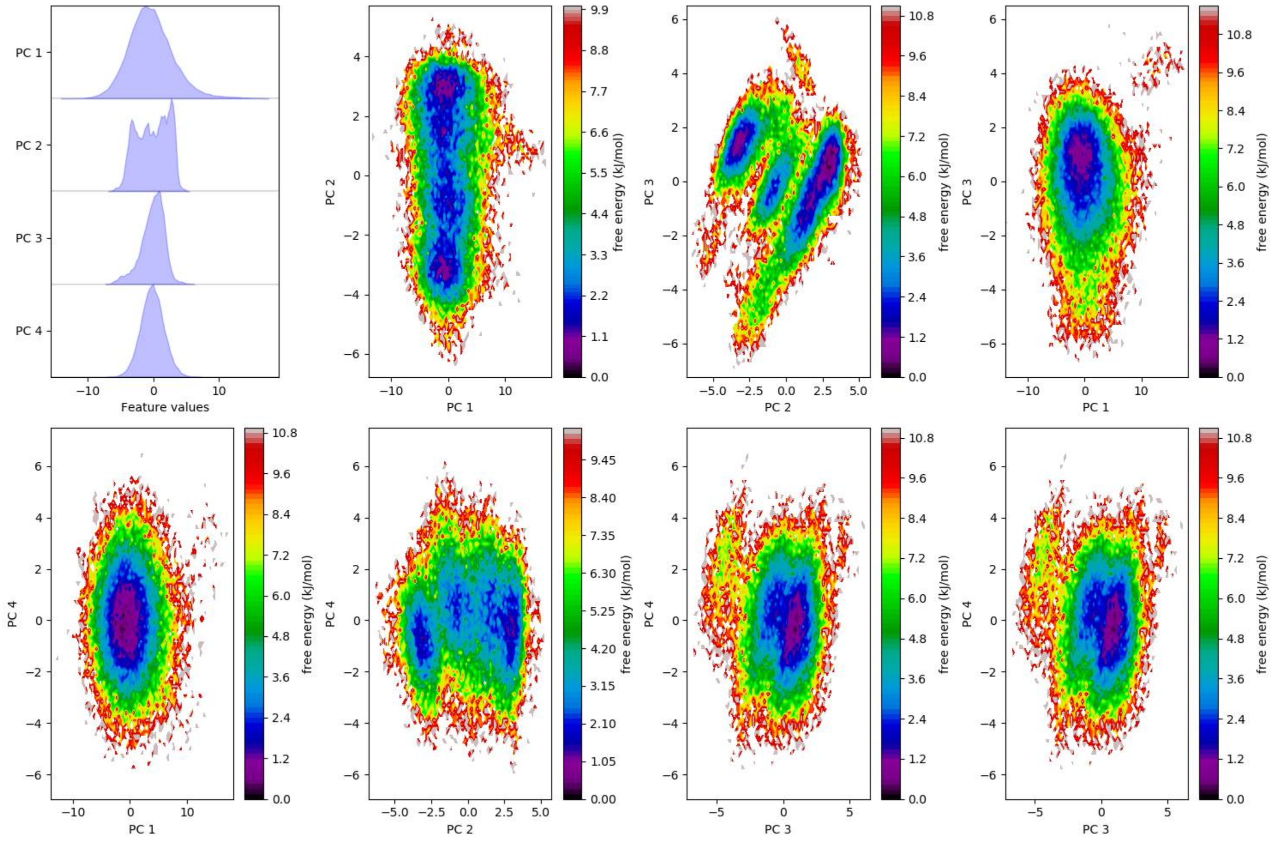

Figure S106 PCA projections between 1-4

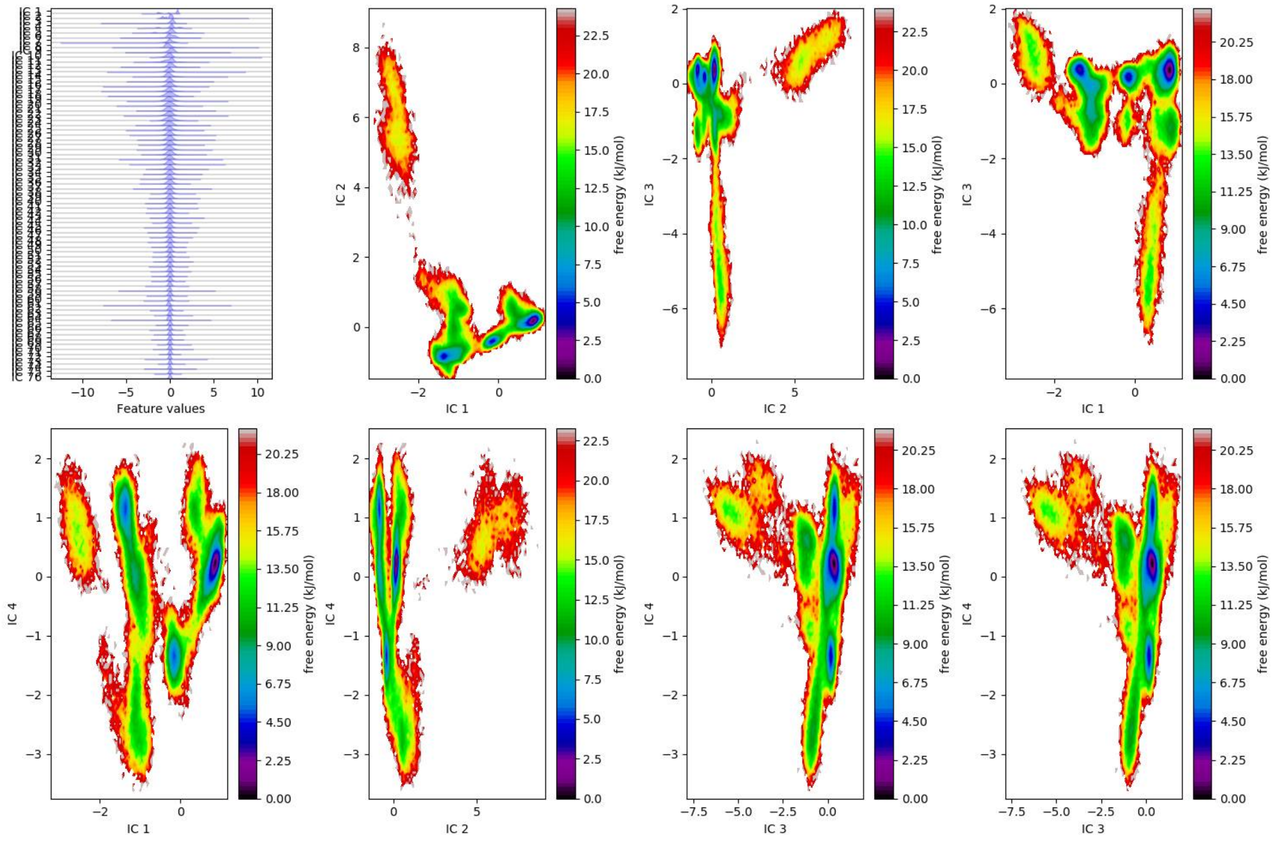

Figure S107 TICA projections between 1-4

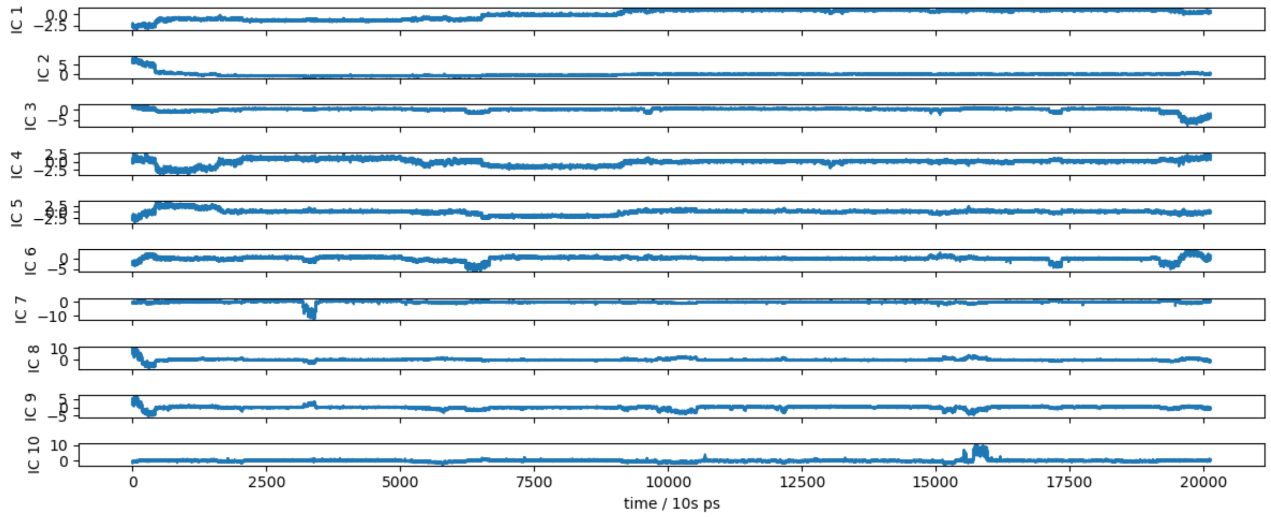

Figure S108 First 10 IC values over the simulation time

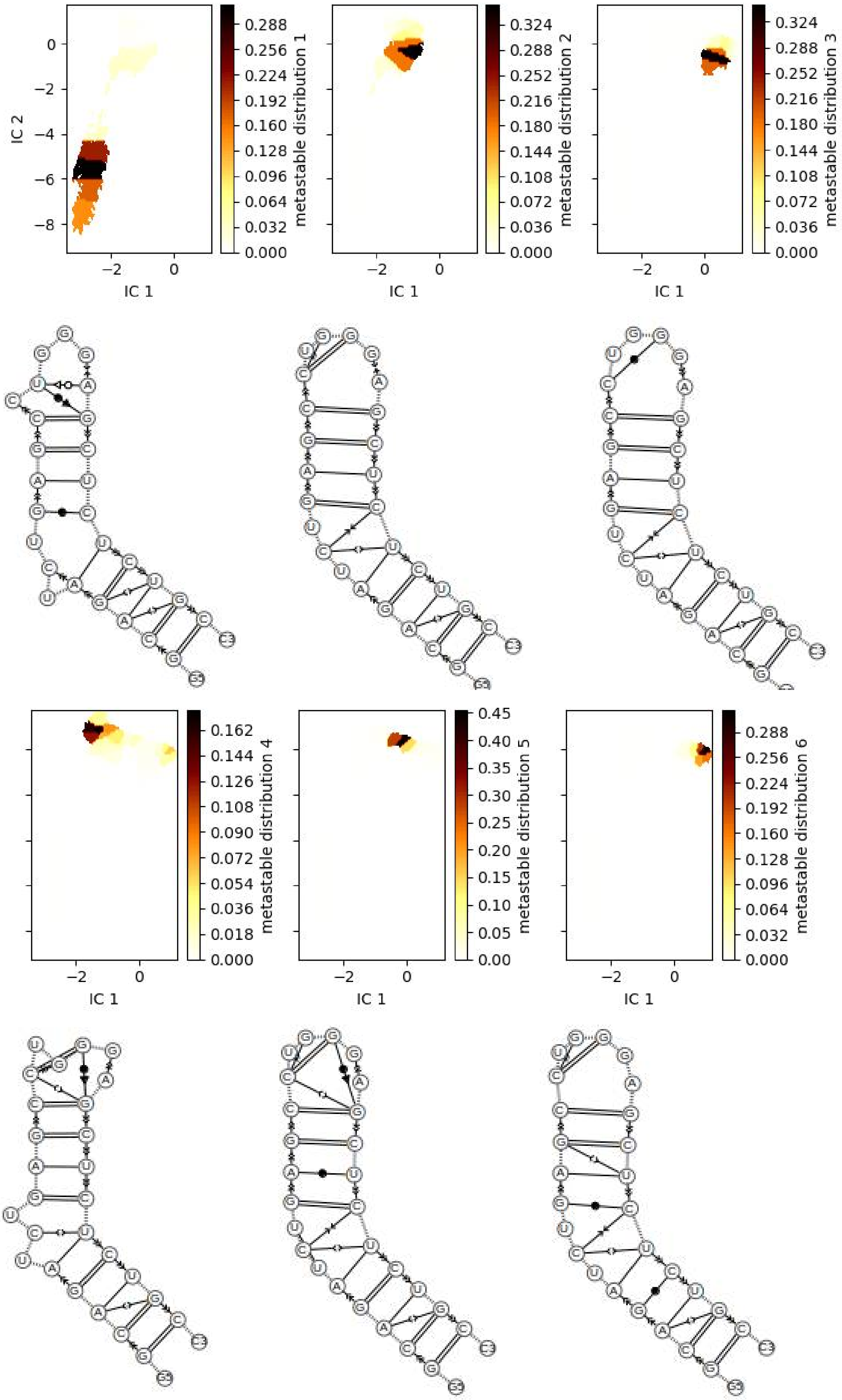

Figure S109 Regions of the TICA landscape and corresponding structure

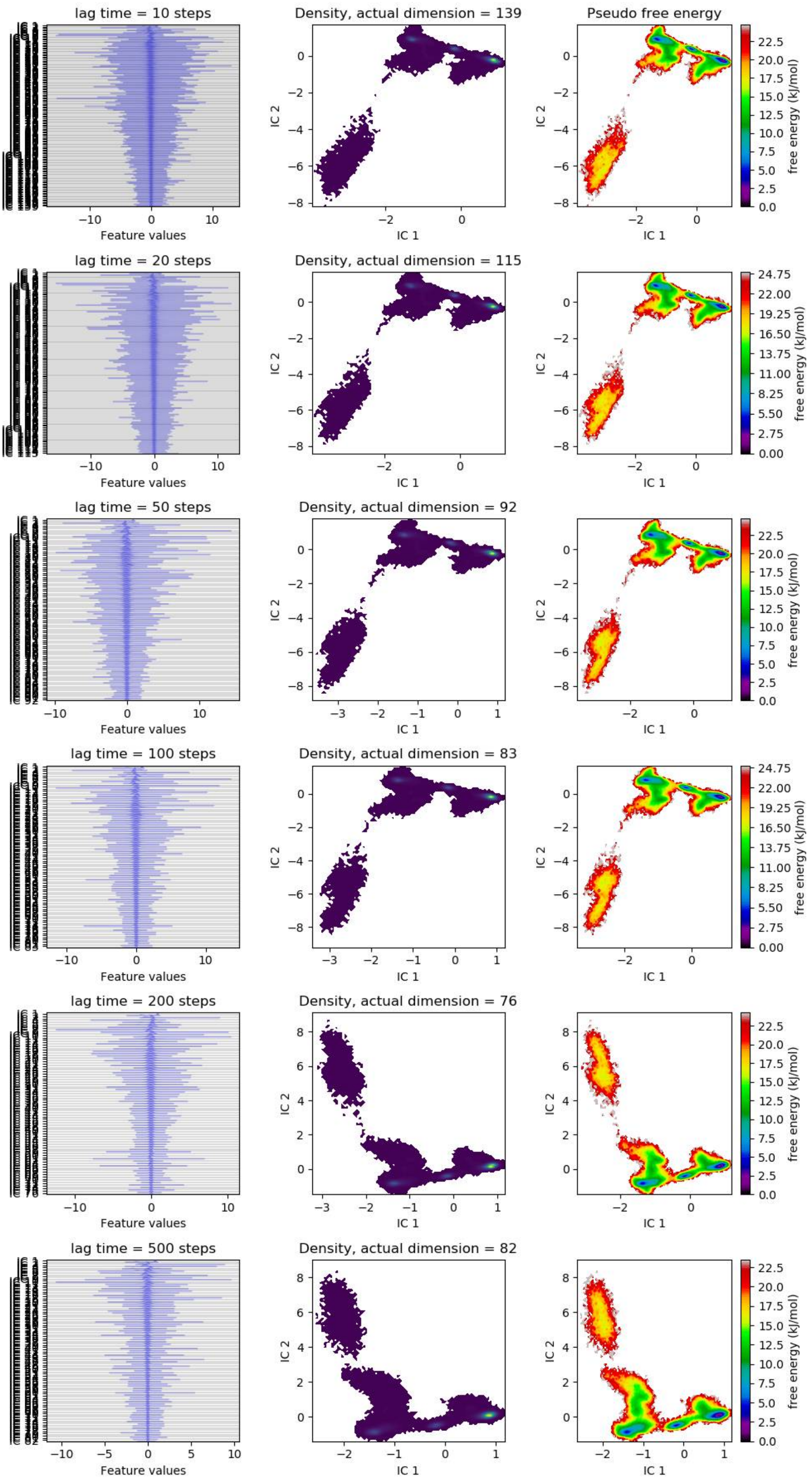

Figure S110 TICA dimensions, density and corrected heat map for steps 10 to 500

1uui

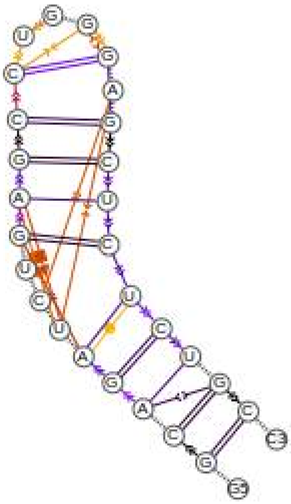

Figure S111 LW representation of the 2μs simulation starting from pdb 1UUI after removing the ligand.

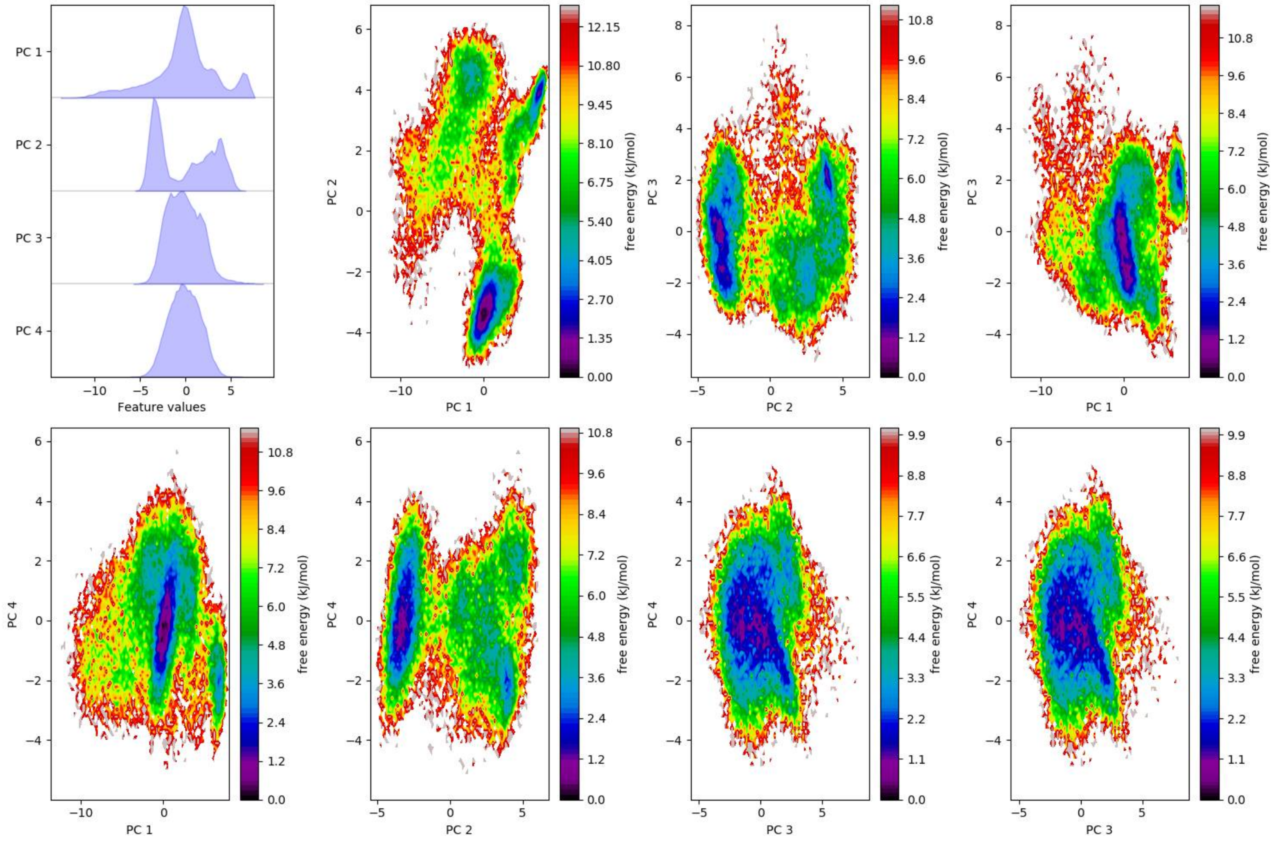

Figure S112 PCA projections between 1-4

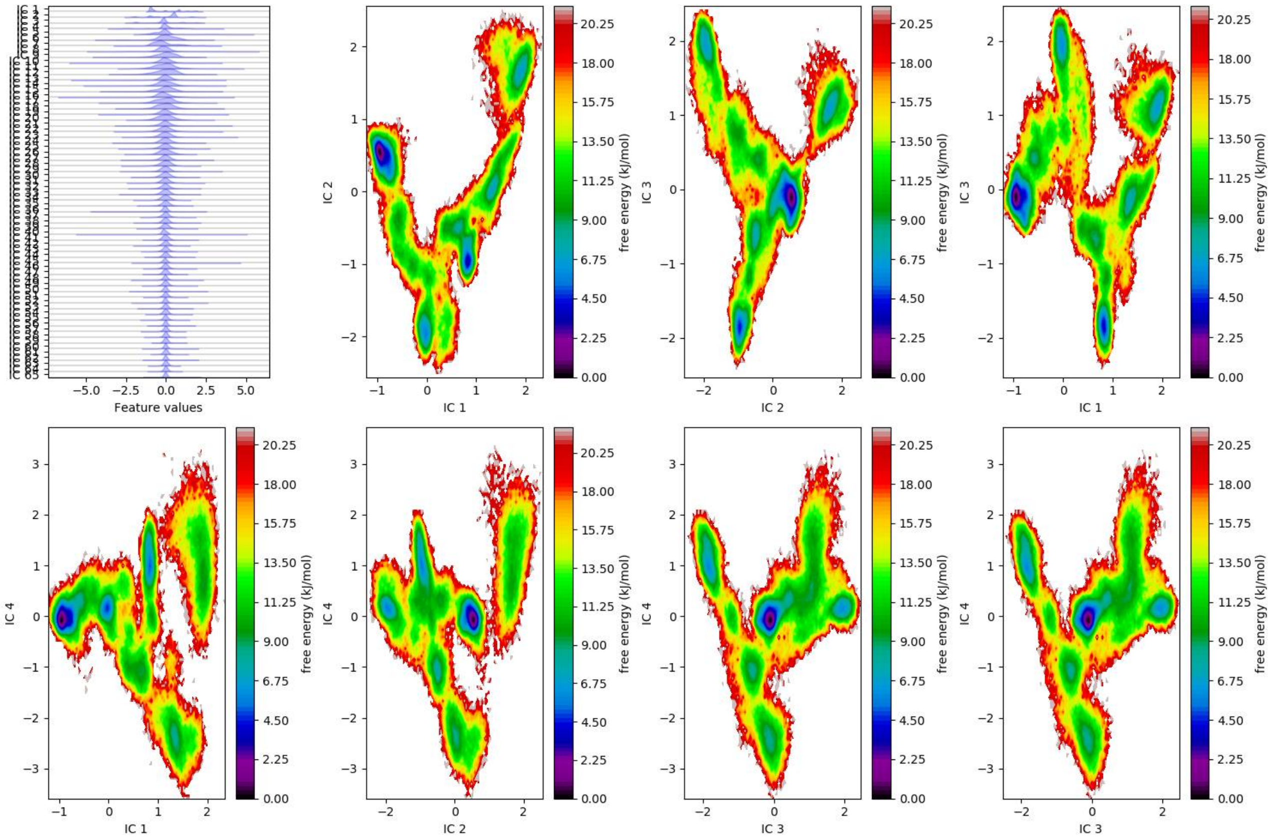

Figure S113 TICA projections between 1-4

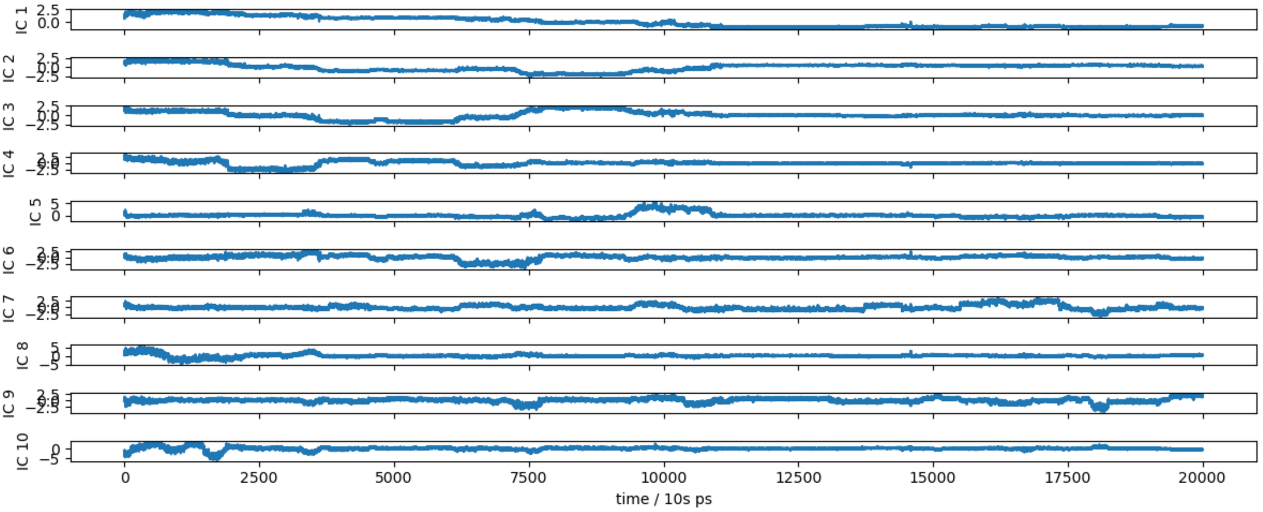

Figure S114 First 10 IC values over the simulation time

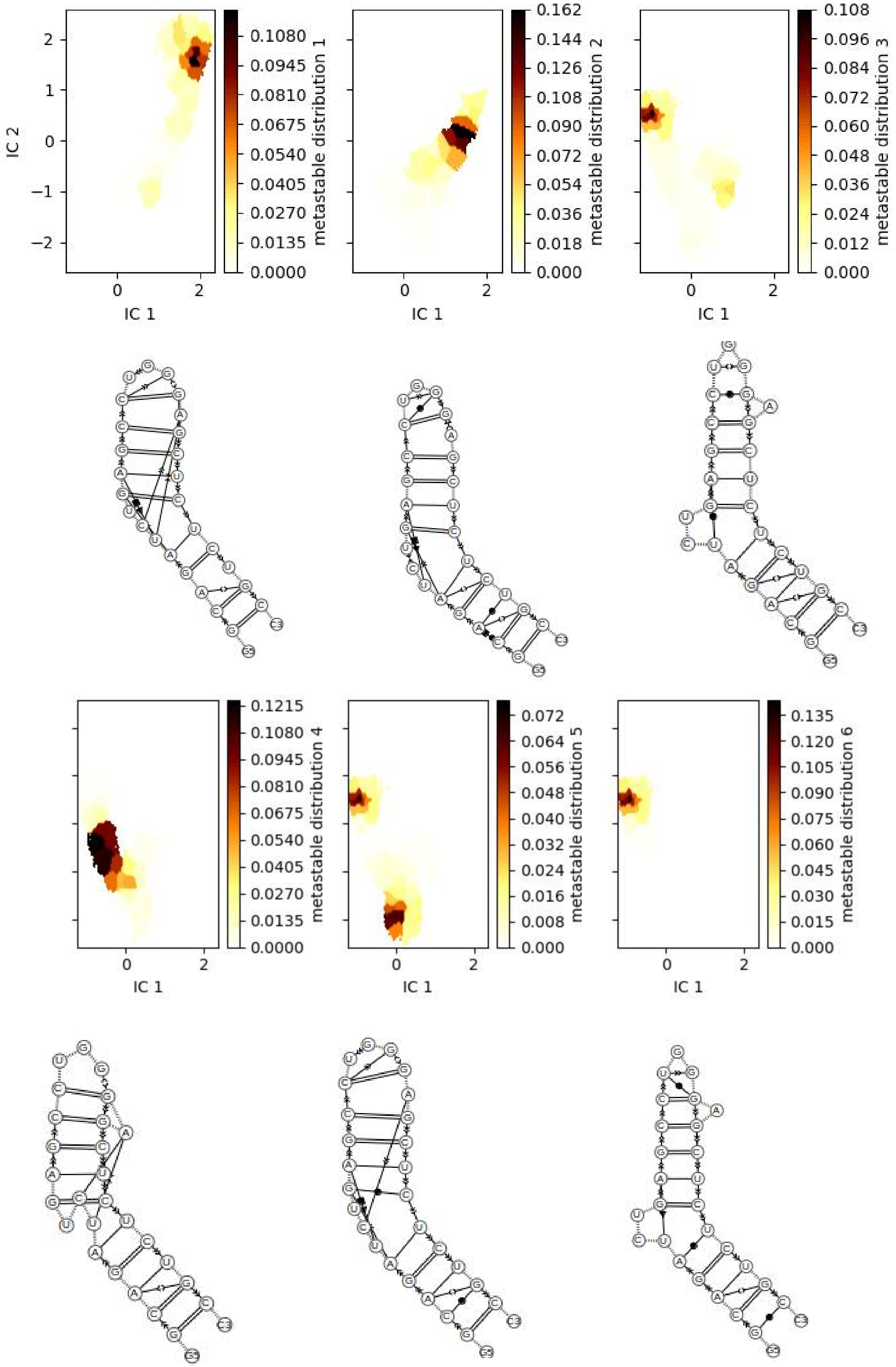

FigureS115 Regionsof the TICA landscape and corresponding structure

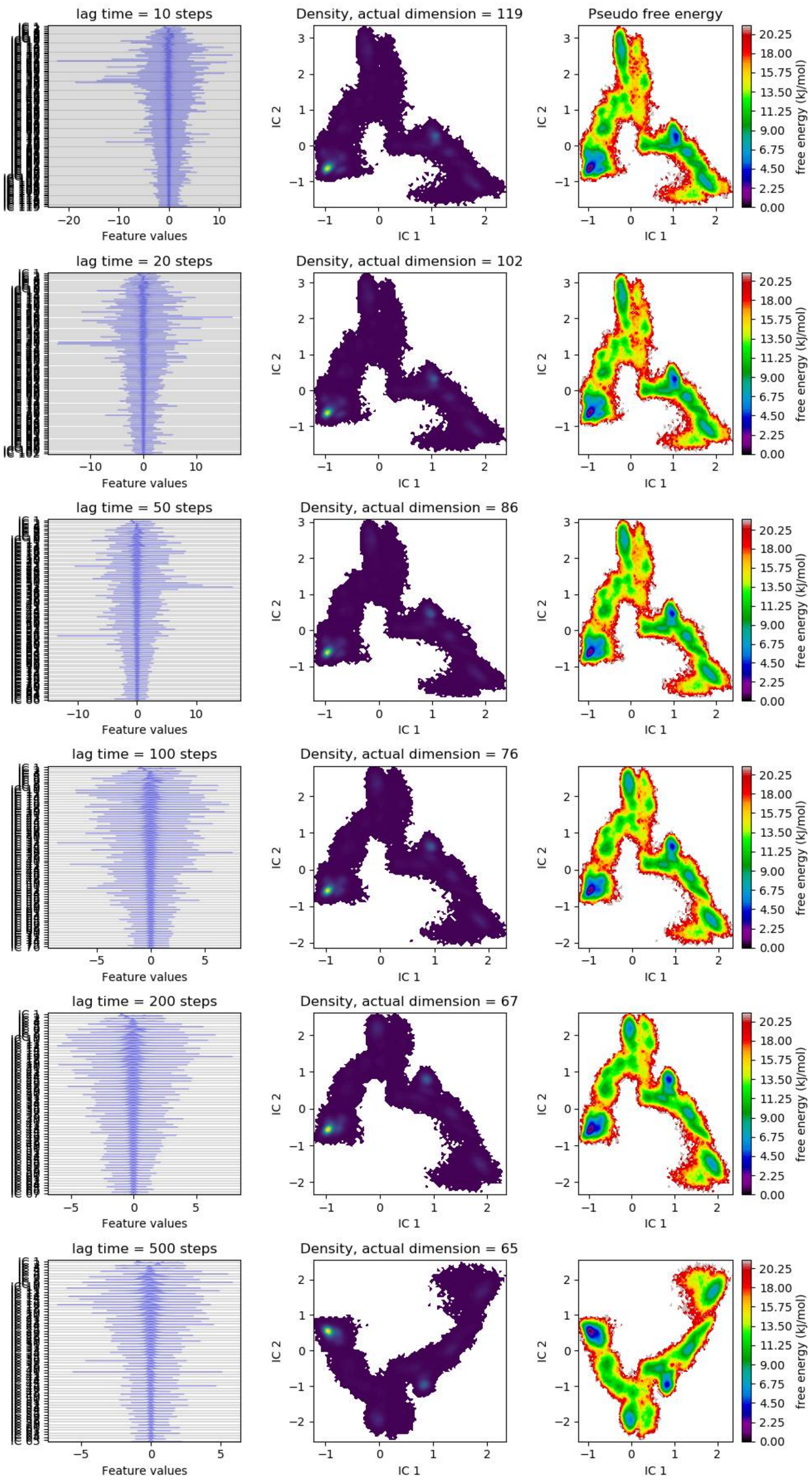

Figure S116 TICA dimensions, density and corrected heat map for steps 10 to 500

SUMMARY of simulations starting from 1 3 4 10 12 16

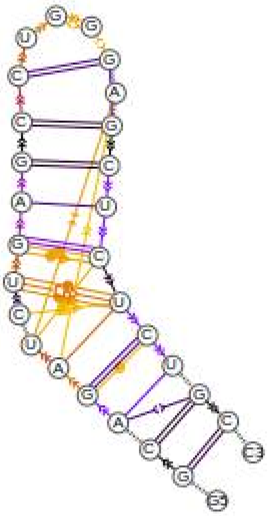

Figure S117 LW representation of concatenated independent trajectories

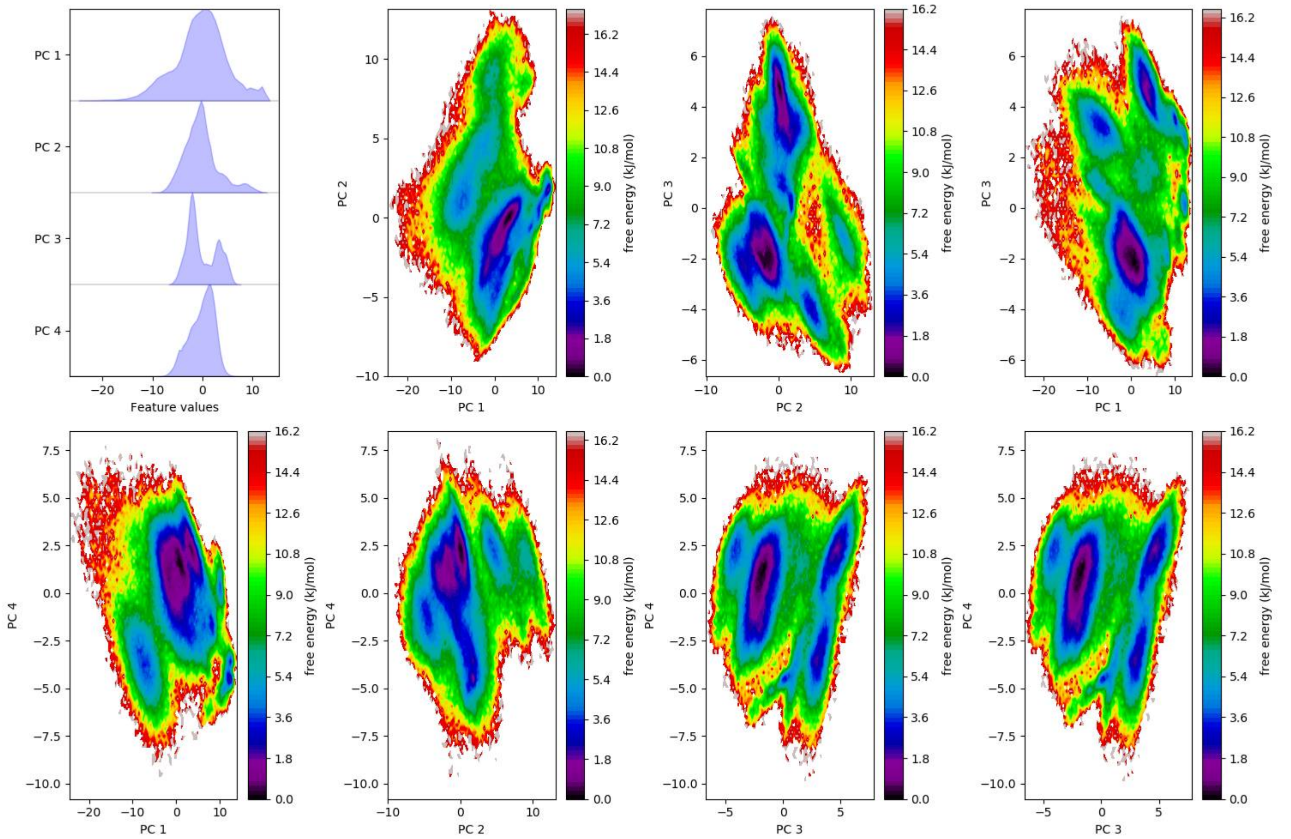

Figure S118 PCA projections between 1-4

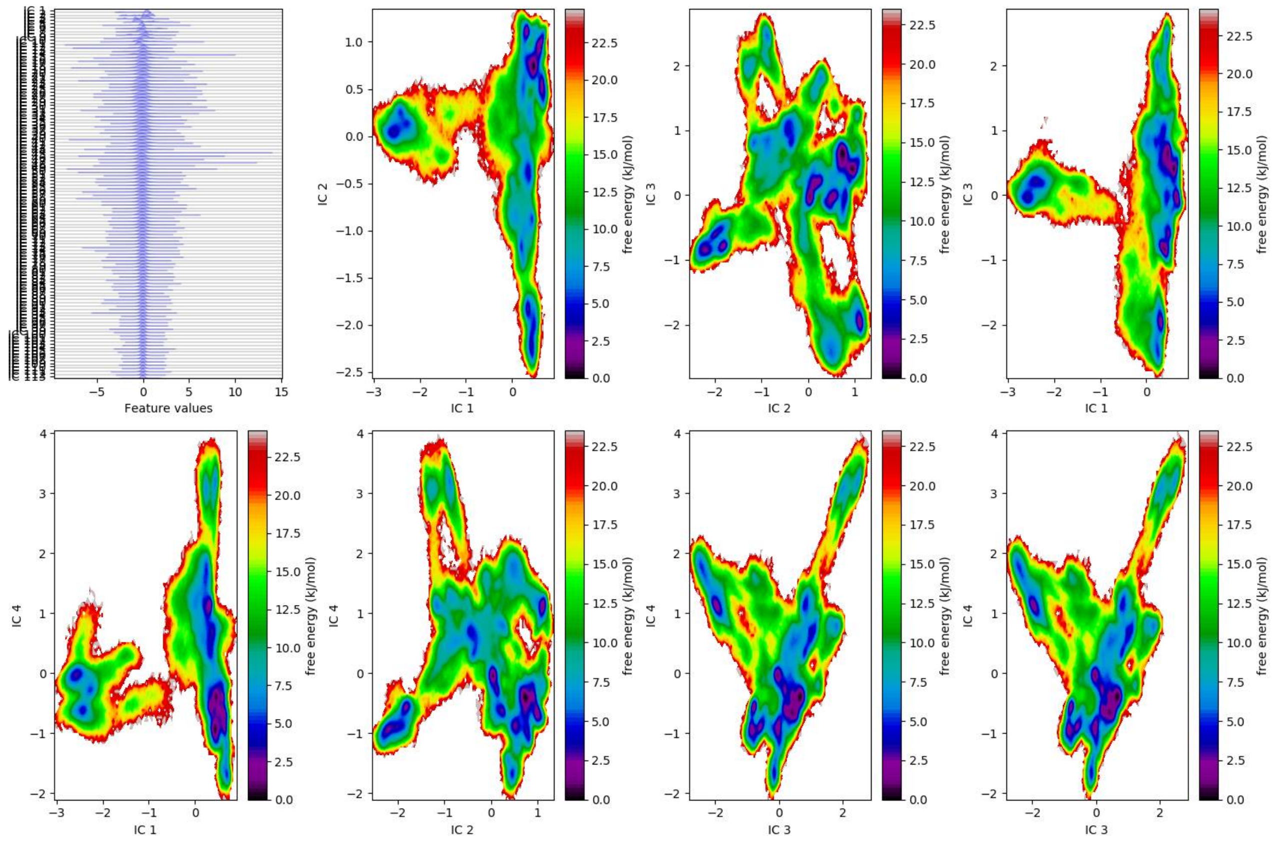

Figure S119 TICA projections between 1-4

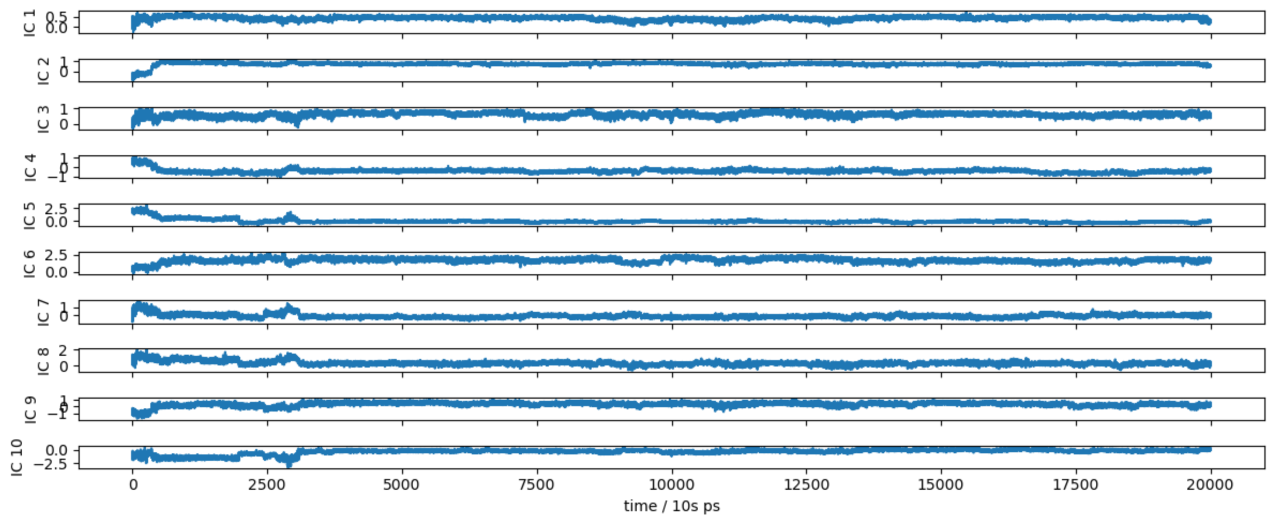

Figure S120 First 10 IC values over the simulation time

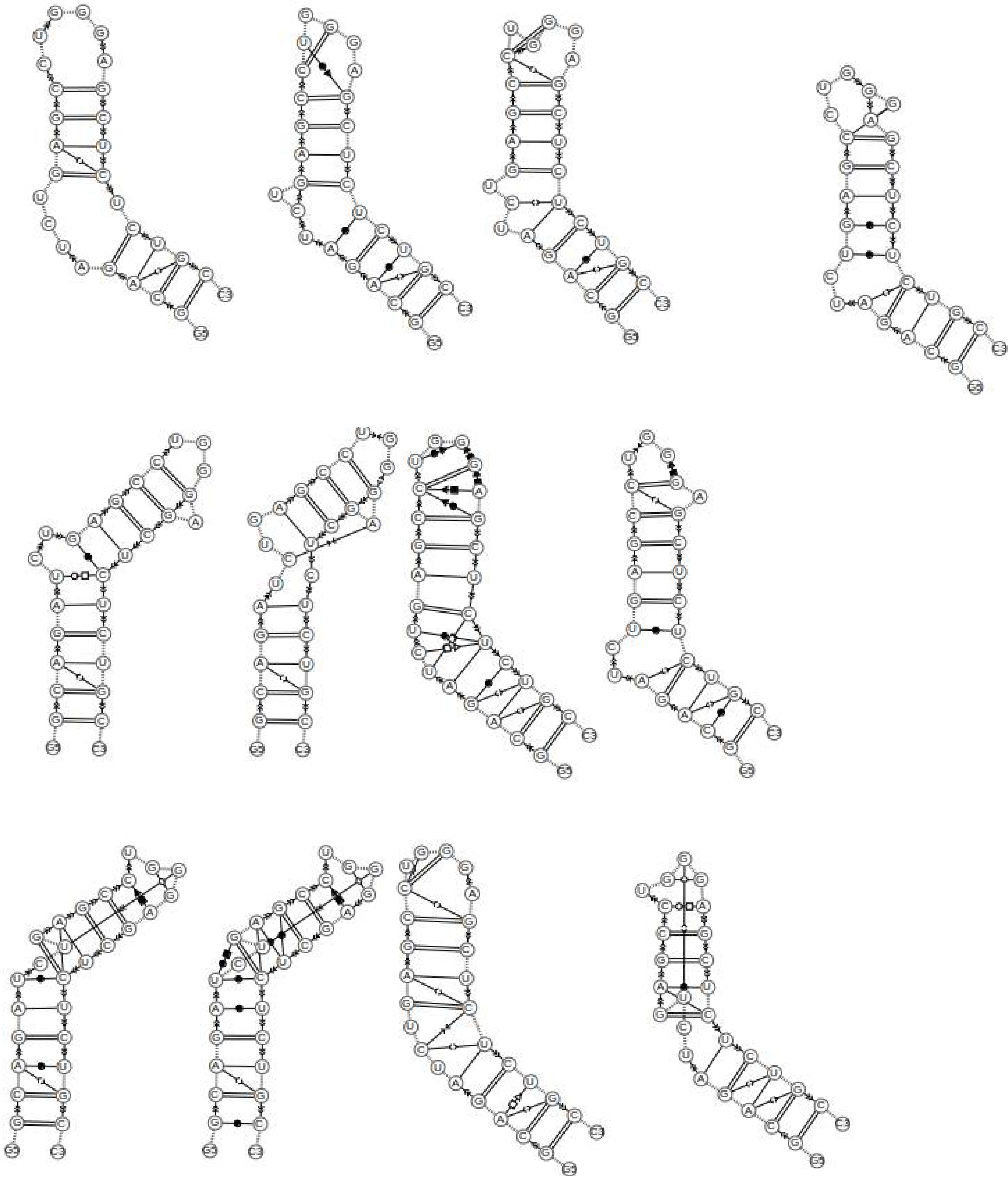

Figure S121 Secondary structures of metastable points in the simulation (i)

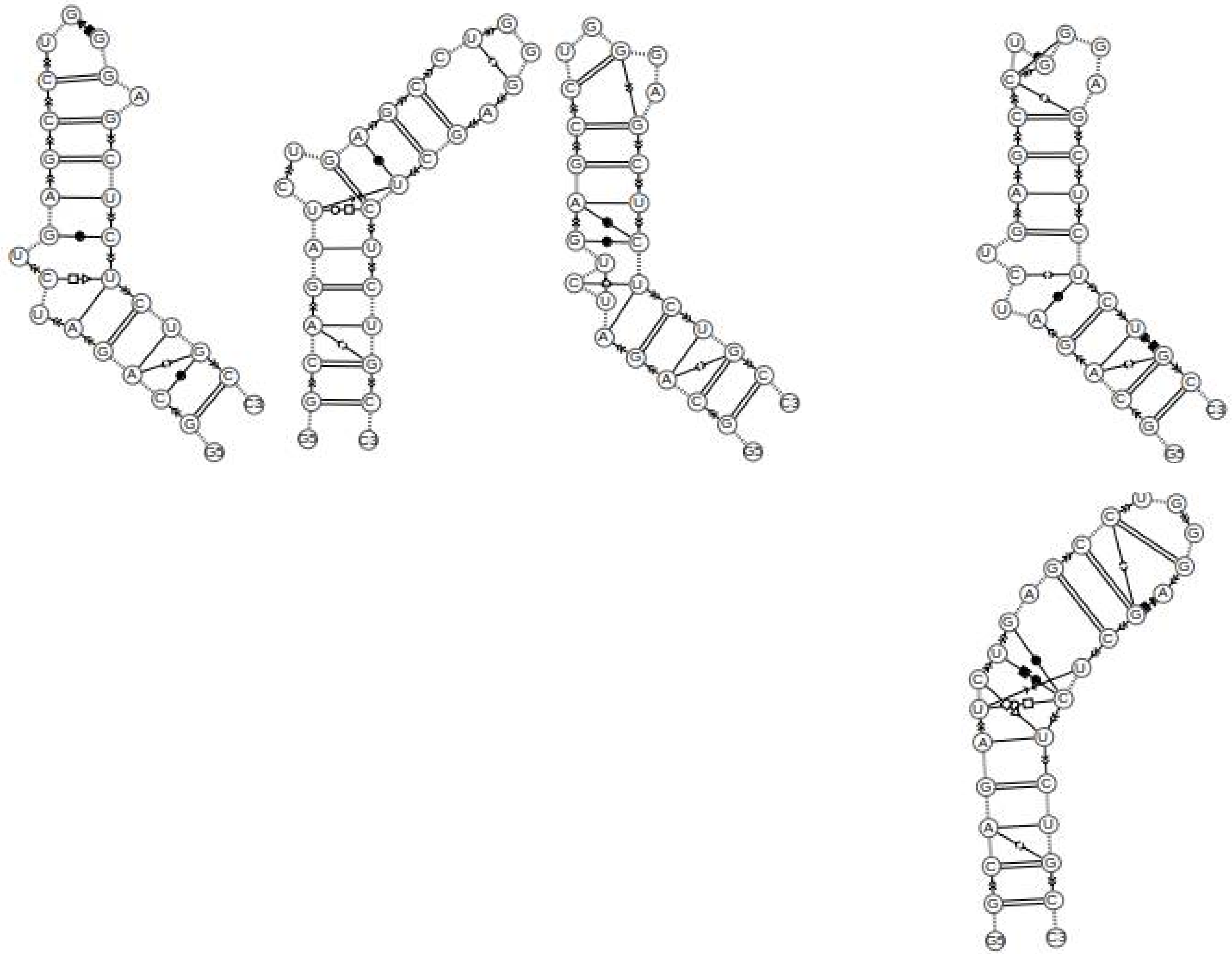

Figure S122 Secondary structures of metastable points in the simulation (ii)

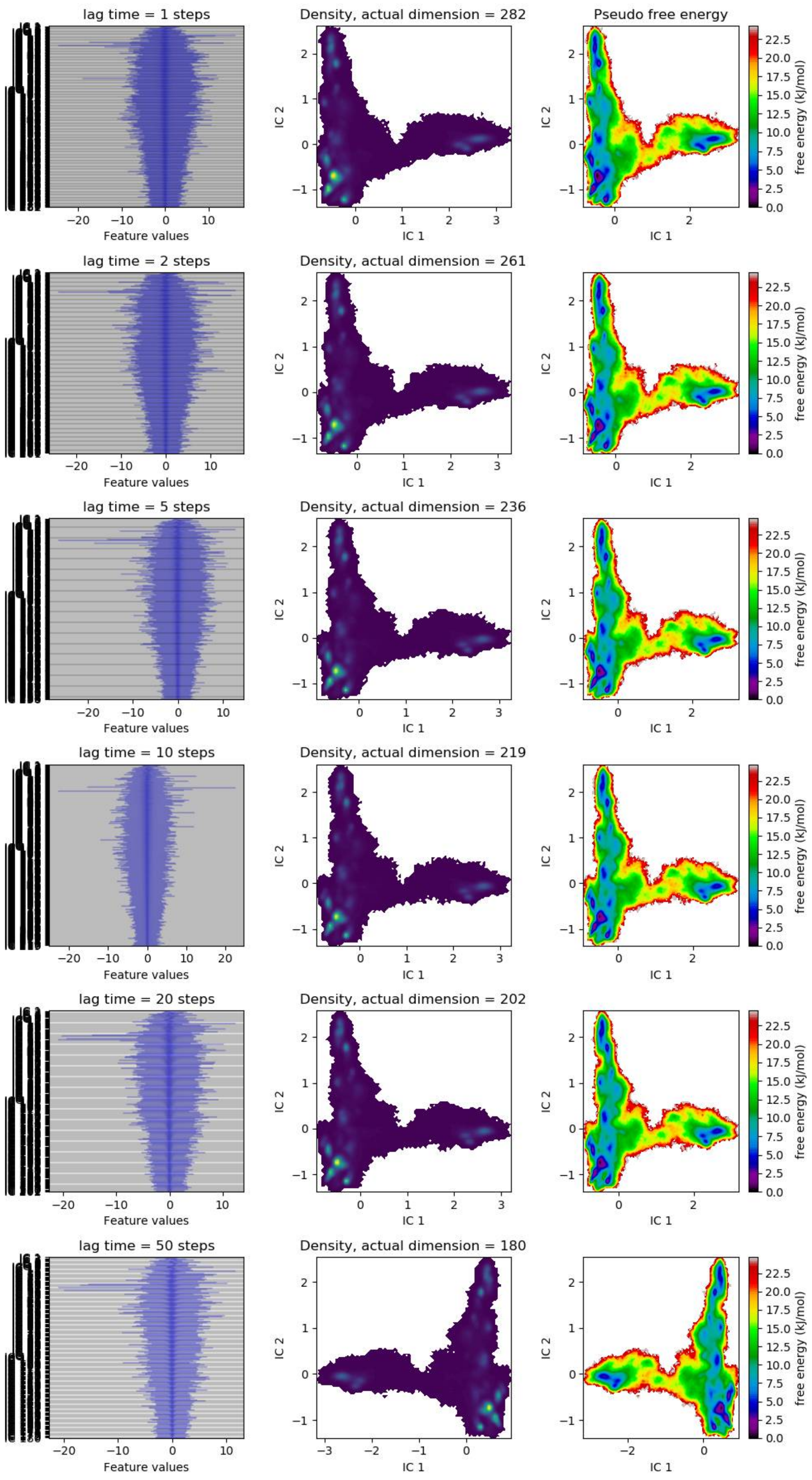

Figure S123 TICA dimensions, density and corrected heat map for steps 1 to 50

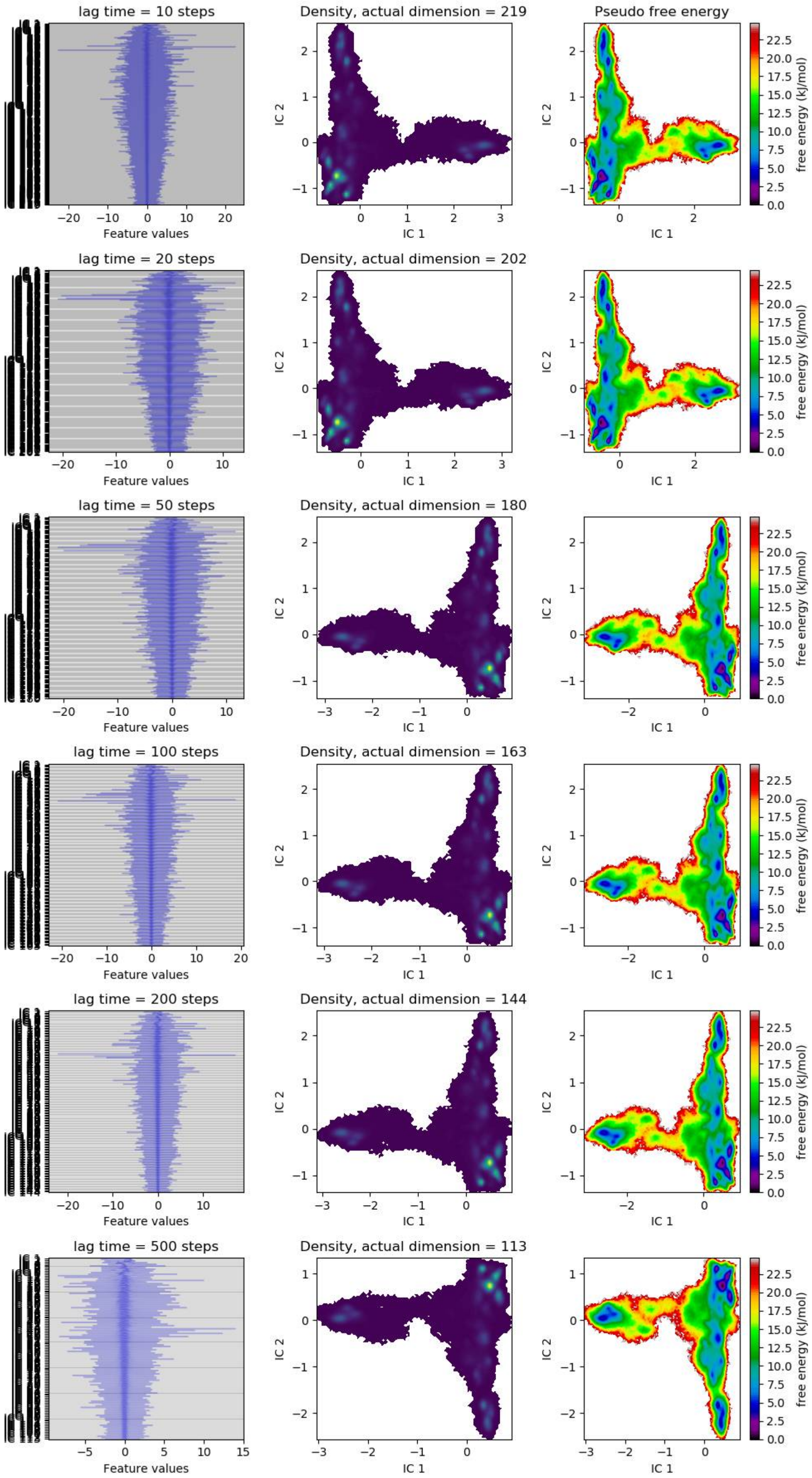

Figure S124 TICA dimensions, density and corrected heat map for steps 10 to 500

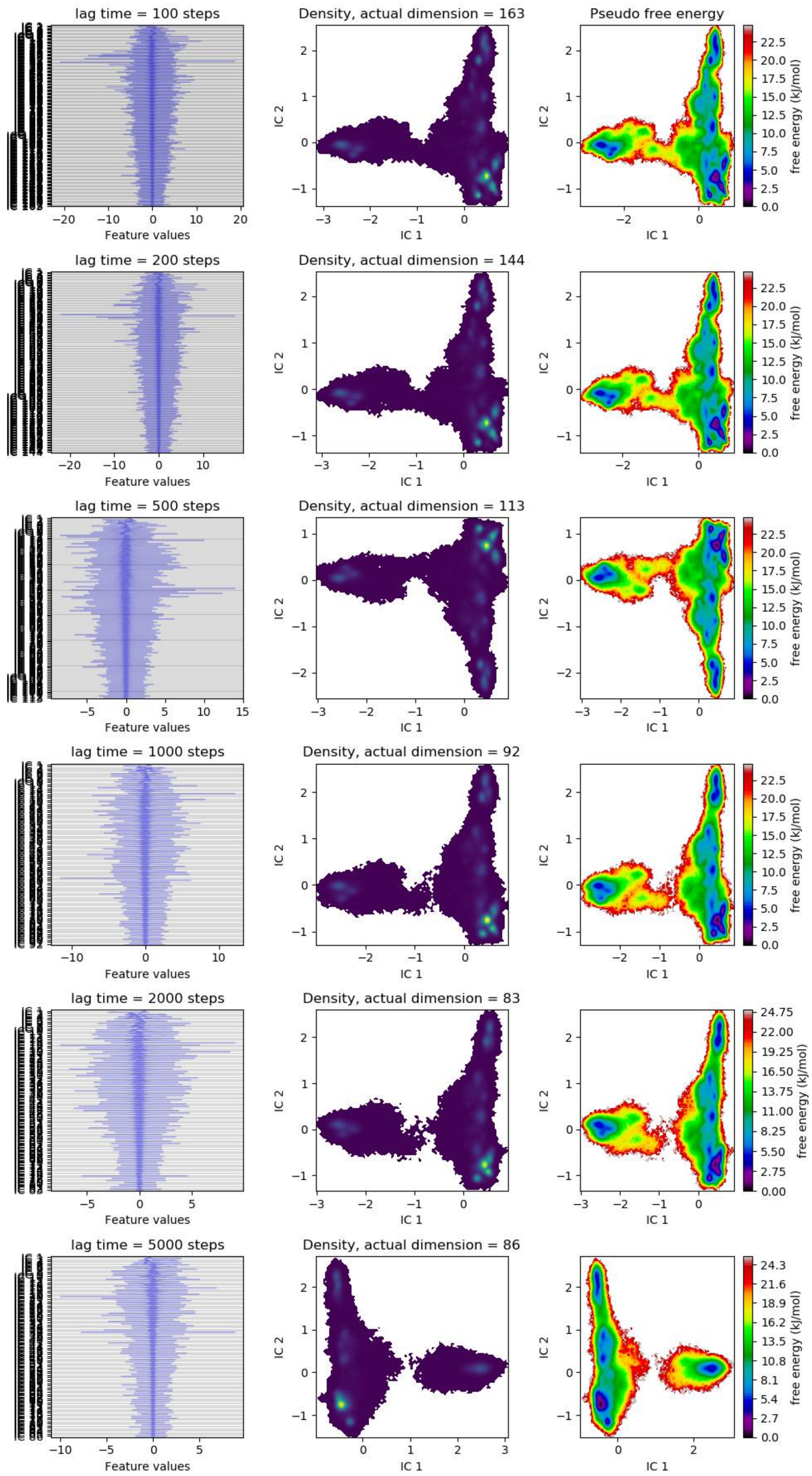

Figure S125 TICA dimensions, density and corrected heat map for steps 100 to 5000

Simulations with cylinder

M -collective

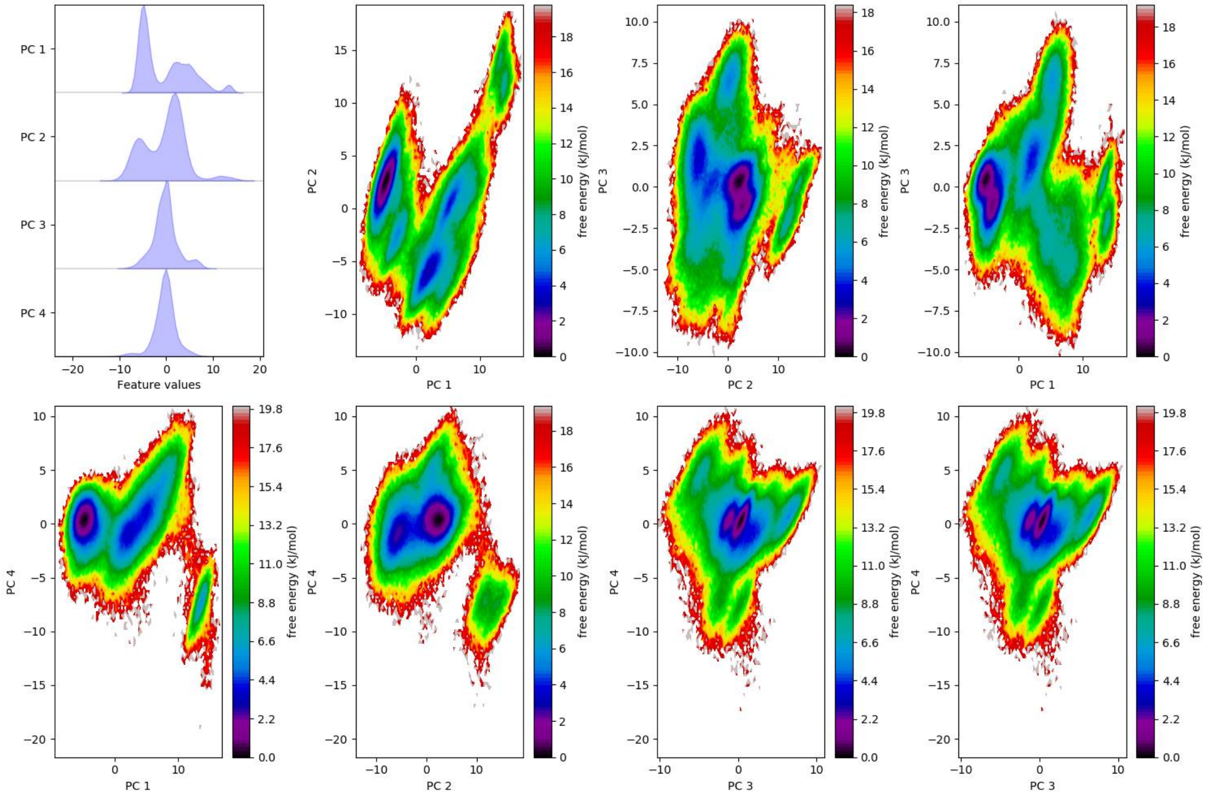

Figure S118 PCA projections between 1-4

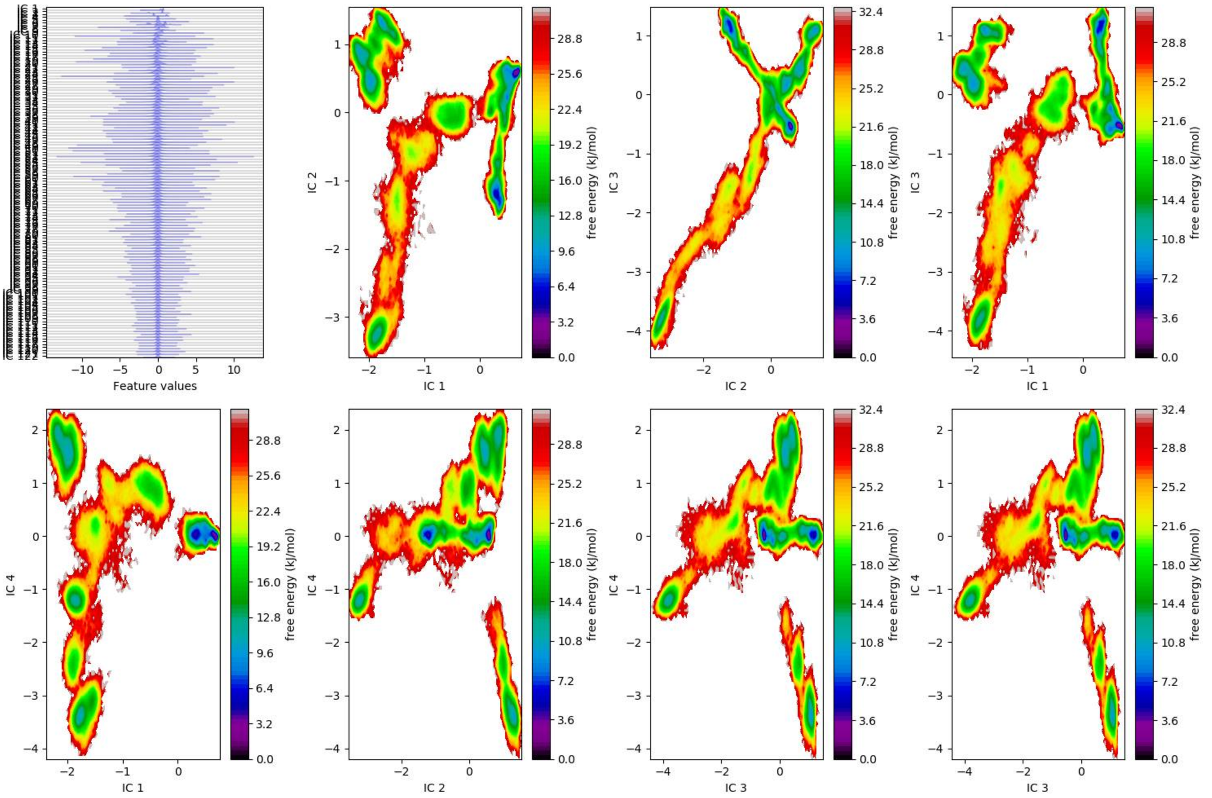

Figure S119 TICA projections between 1-4

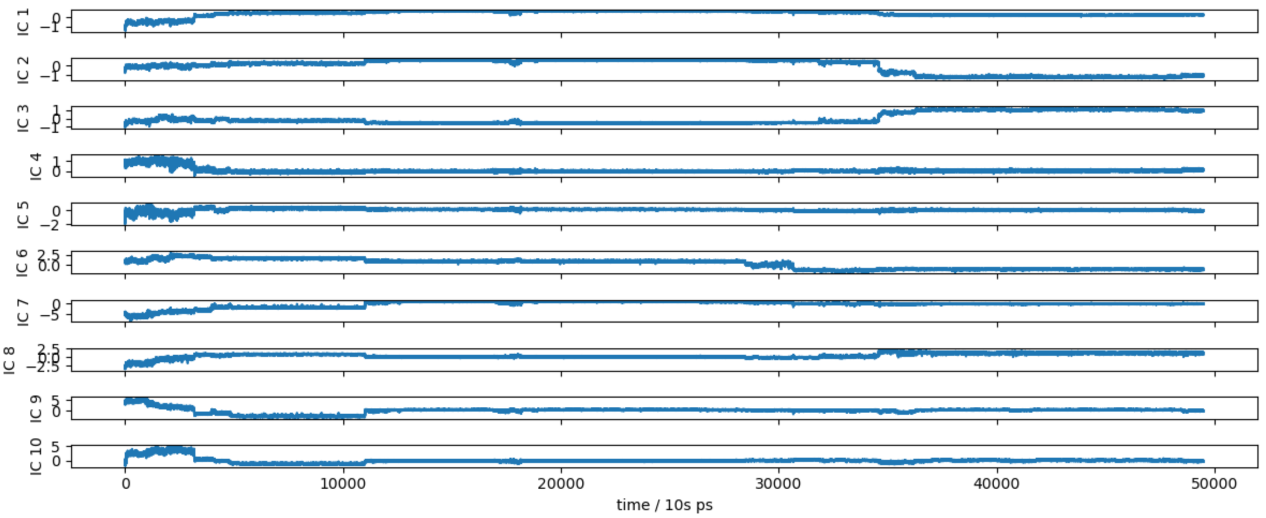

Figure S120 First 10 IC values over the simulation time

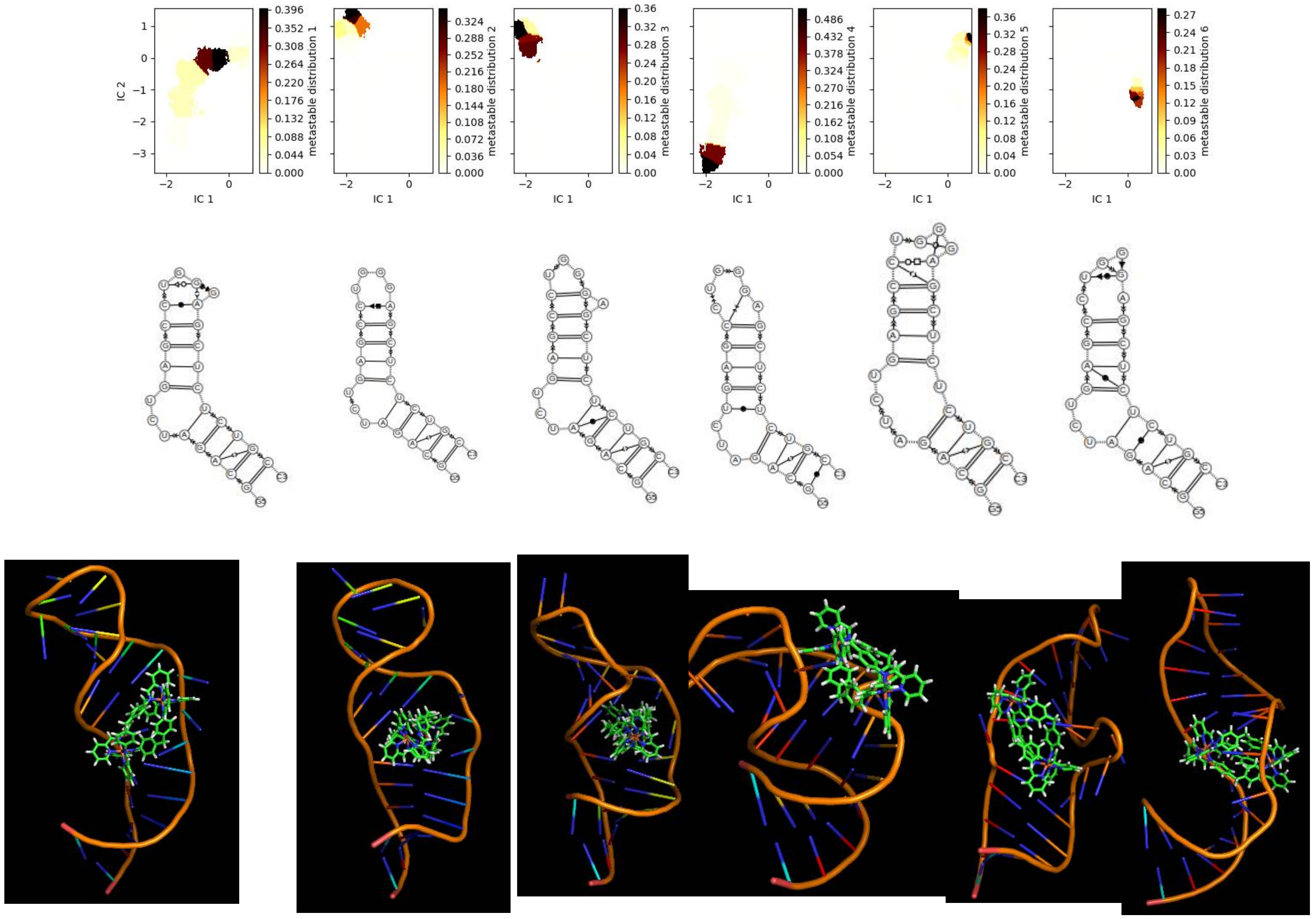

Figure S121 Regions of the TICA landscape and corresponding structure and corresponding e-scores and 3d structure

state	π	G/kT
1	0.000897	7.016480
2	0.001639	6.413909
3	0.003419	5.678400
4	0.003186	5.748975
5	0.314203	1.157715
6	0.676656	0.390592

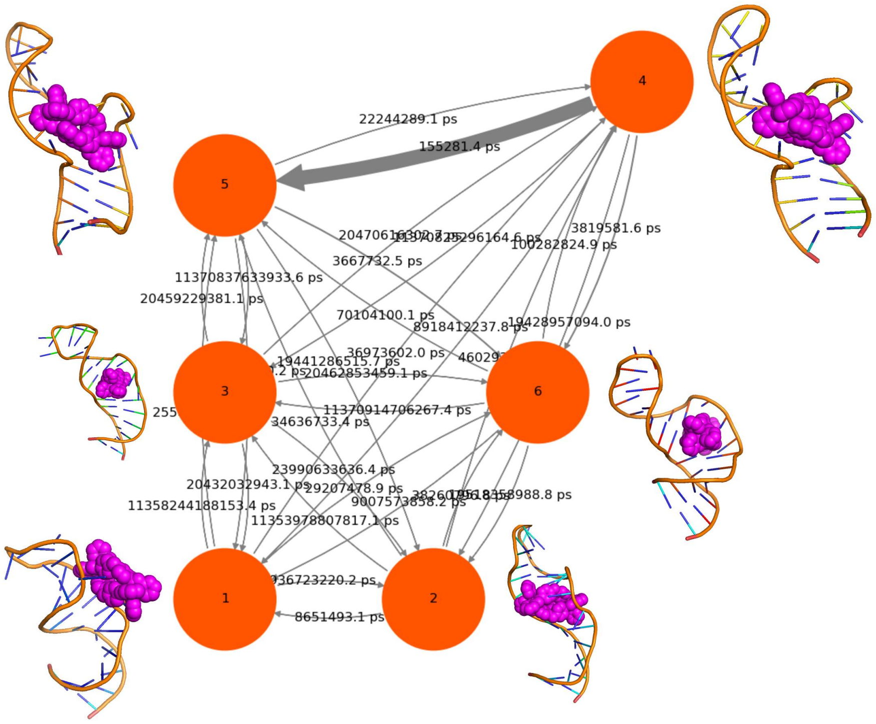

Table 5 Relative energy estimate of each state and transition efficiency

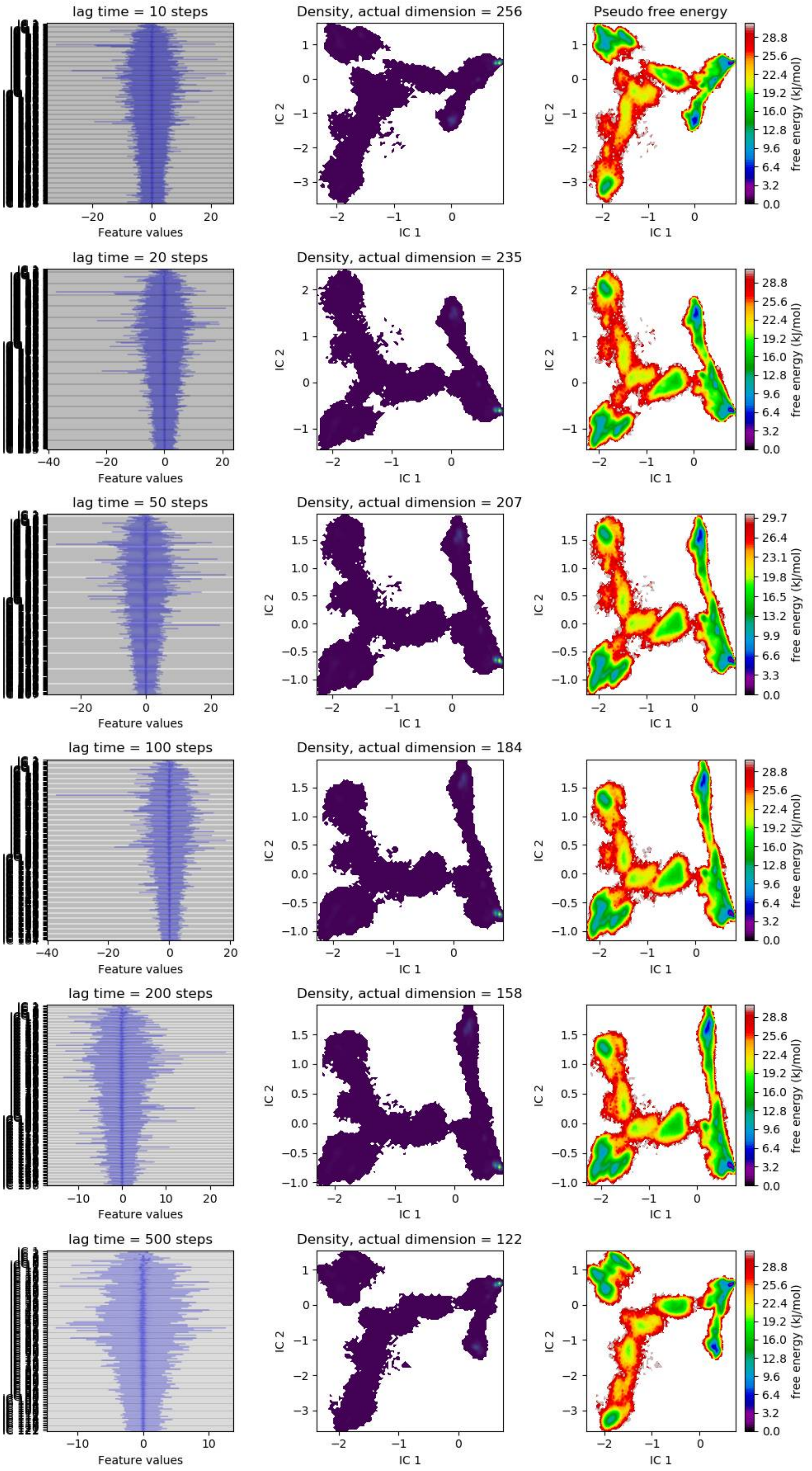

Figure S122 TICA dimensions, density and corrected heat map for steps 10 to 500

M1- 5mcs

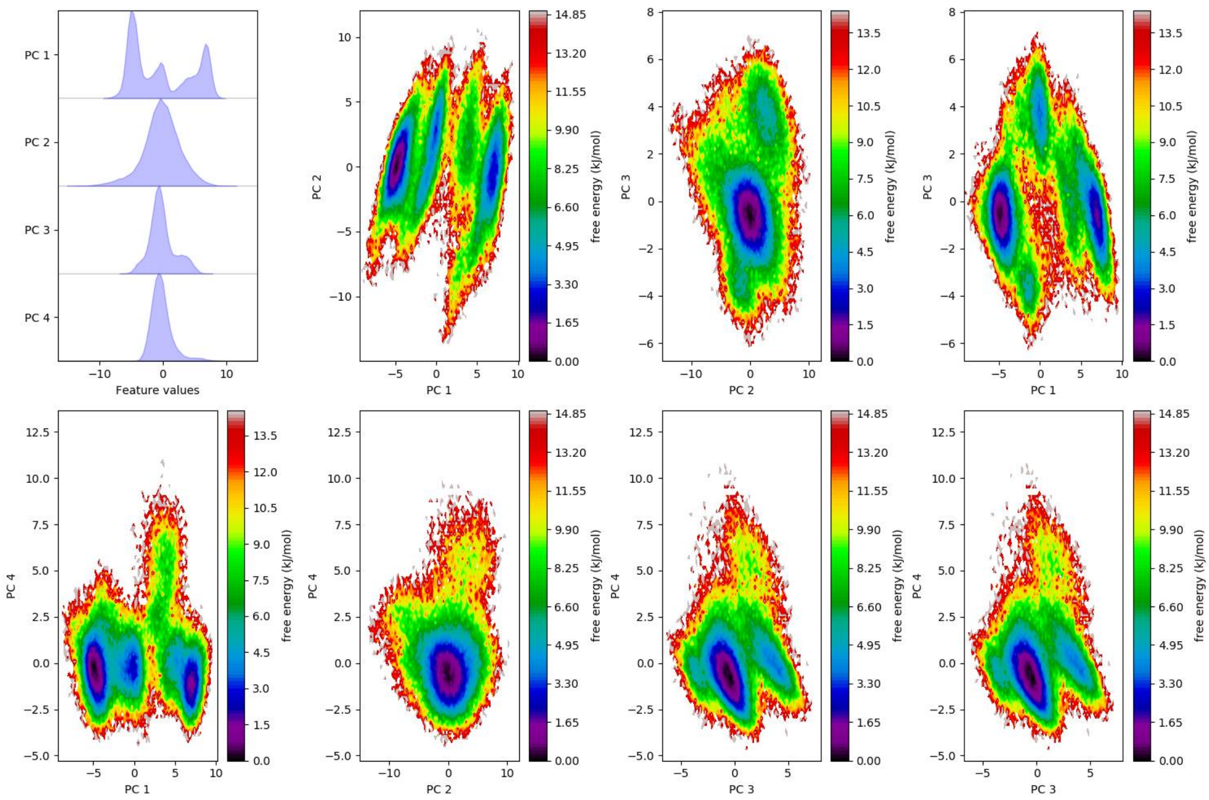

Figure S123 PCA projections between 1-4

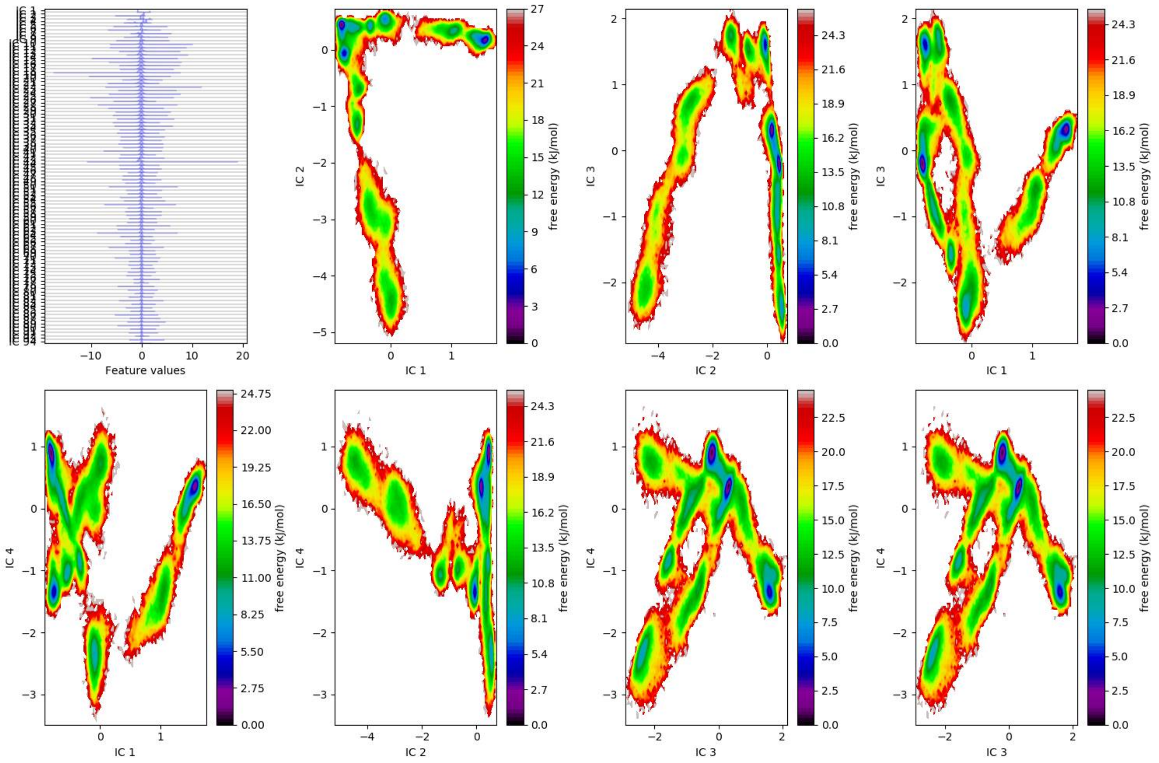

Figure S124 TICA projections between 1-4

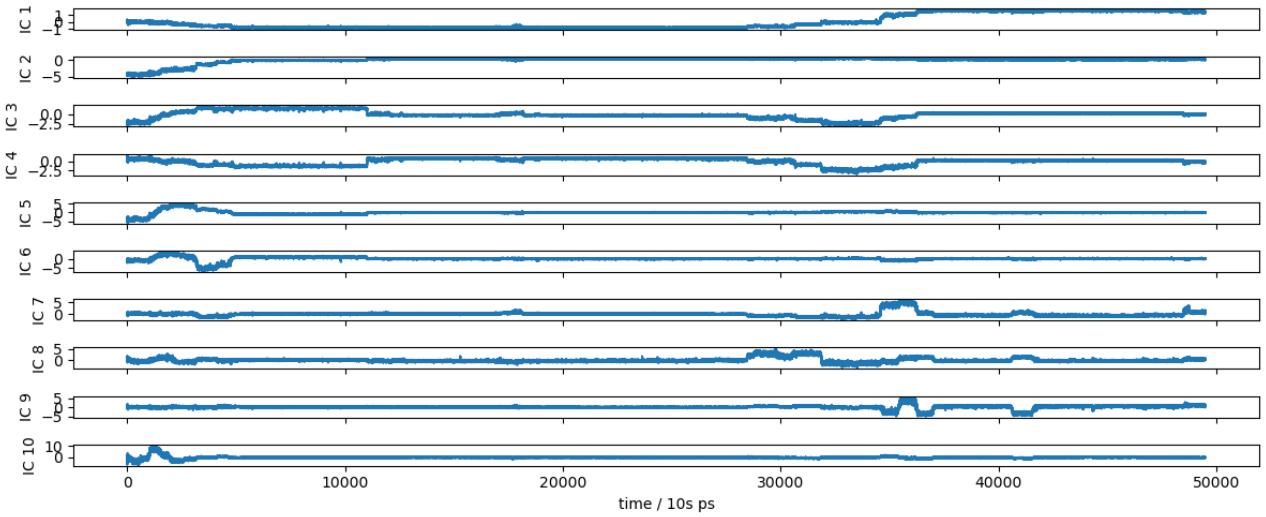

Figure S125 First 10 IC values over the simulation time

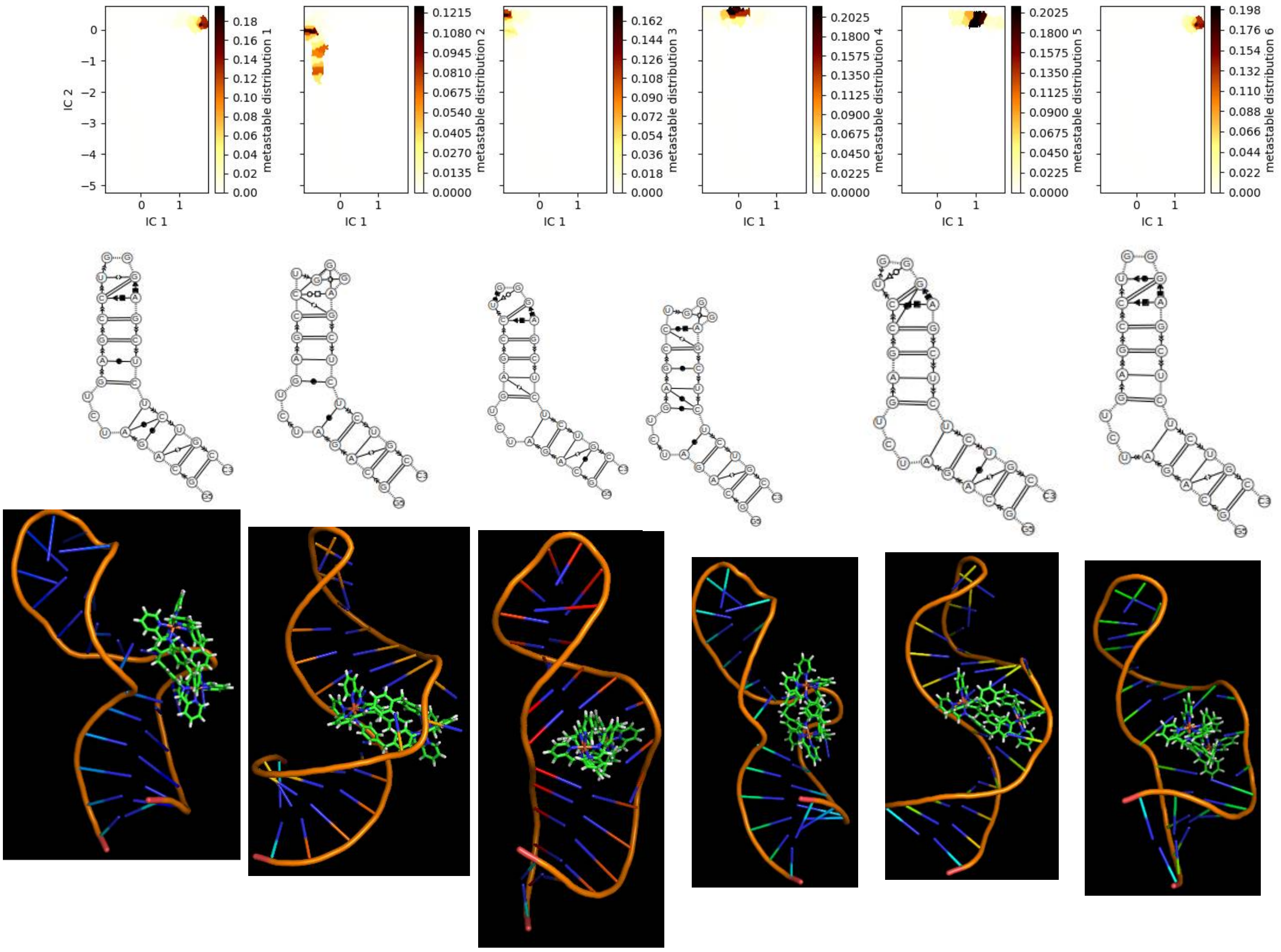

Figure S126 Regions of the TICA landscape and corresponding structure and the corresponding 3D structure

state	π	G/kT
1	0.000002	12.964154
2	0.000066	9.620205
3	0.008501	4.767574
4	0.002728	5.904145
5	0.053610	2.926021
6	0.935092	0.067110

Table 7 Relative energy estimate of each state

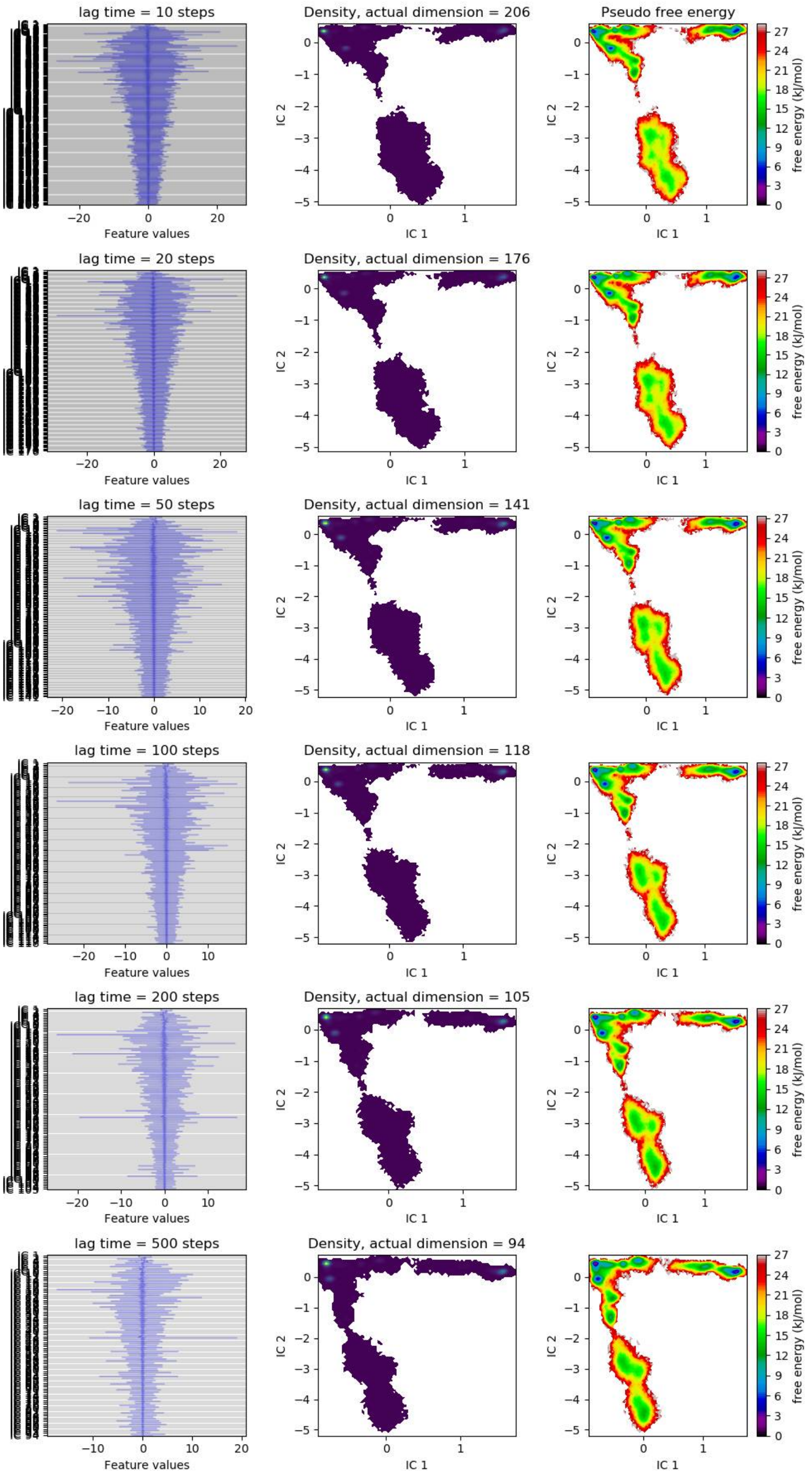

Figure S127 TICA dimensions, density and corrected heat map for steps 10 to 500

M1 5 -M2

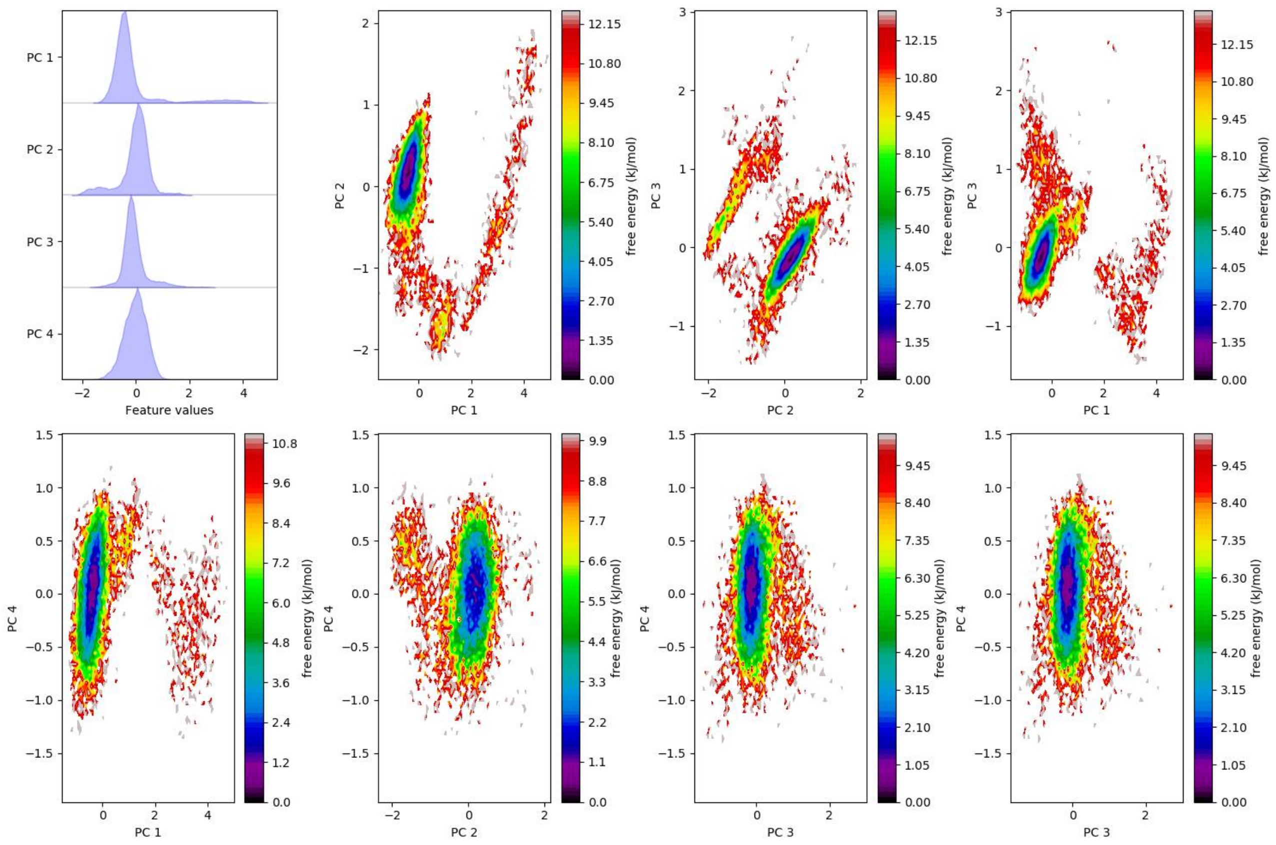

Figure S128 PCA projections between 1-4

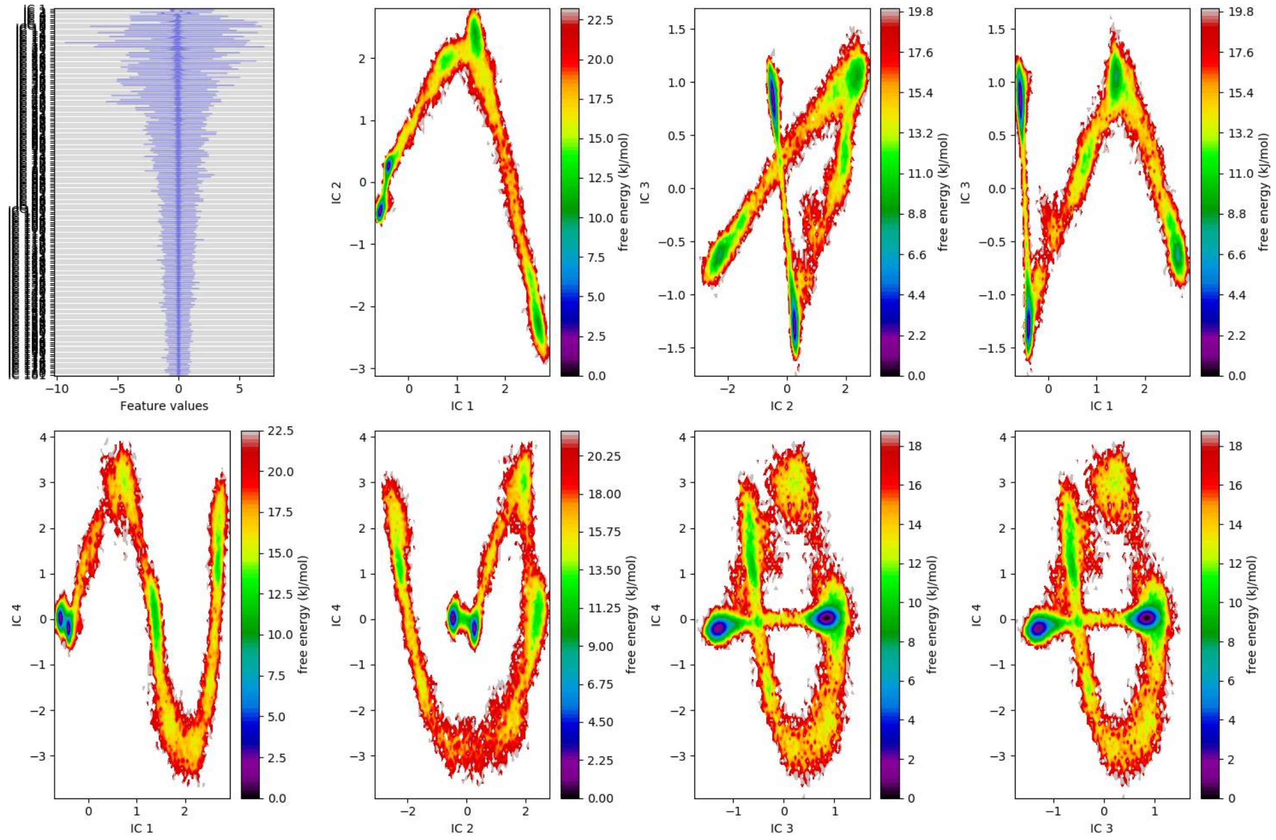

Figure S129 TICA projections between 1-4

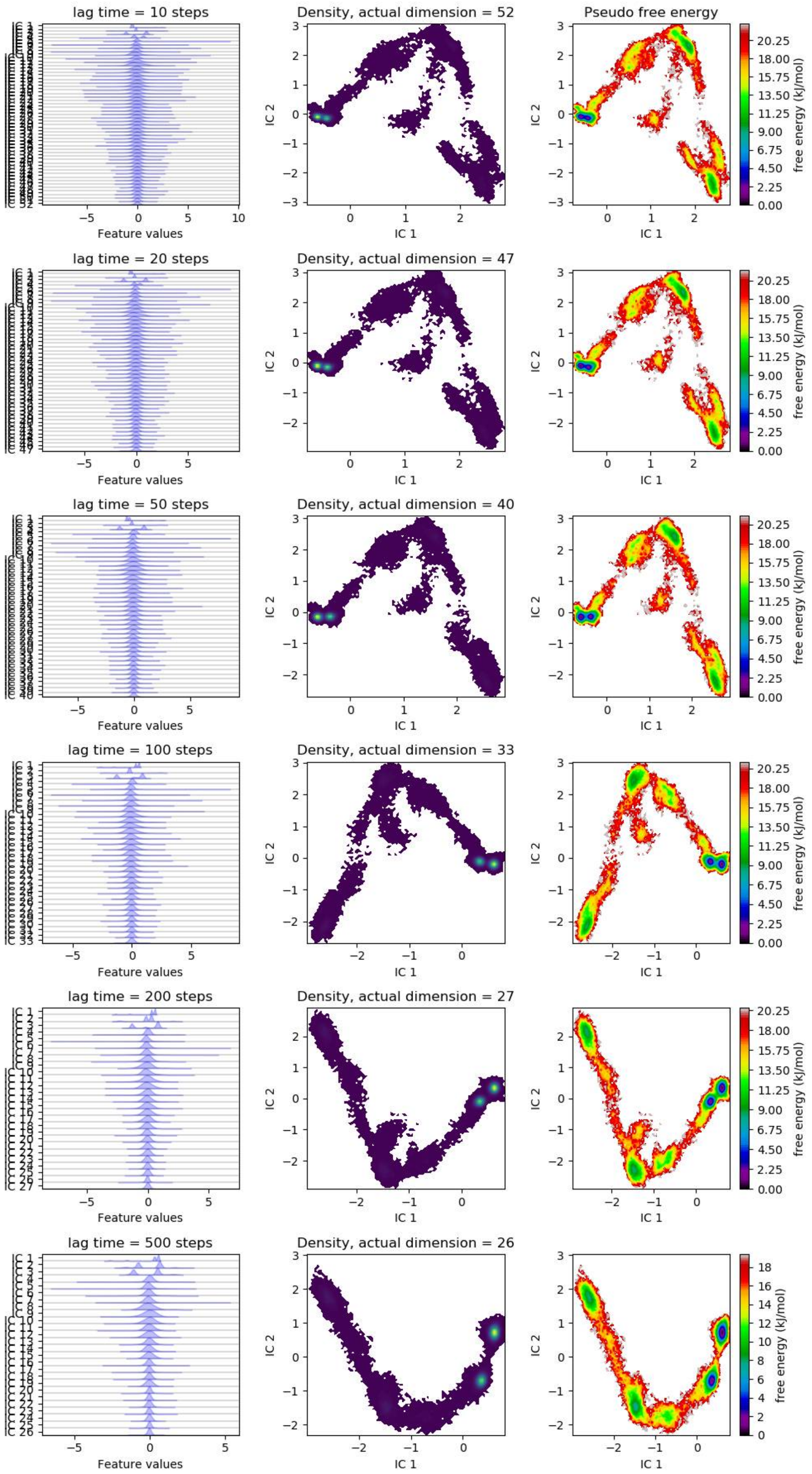

Figure S130 TICA dimensions, density and corrected heat map for steps 10 to 500

M3

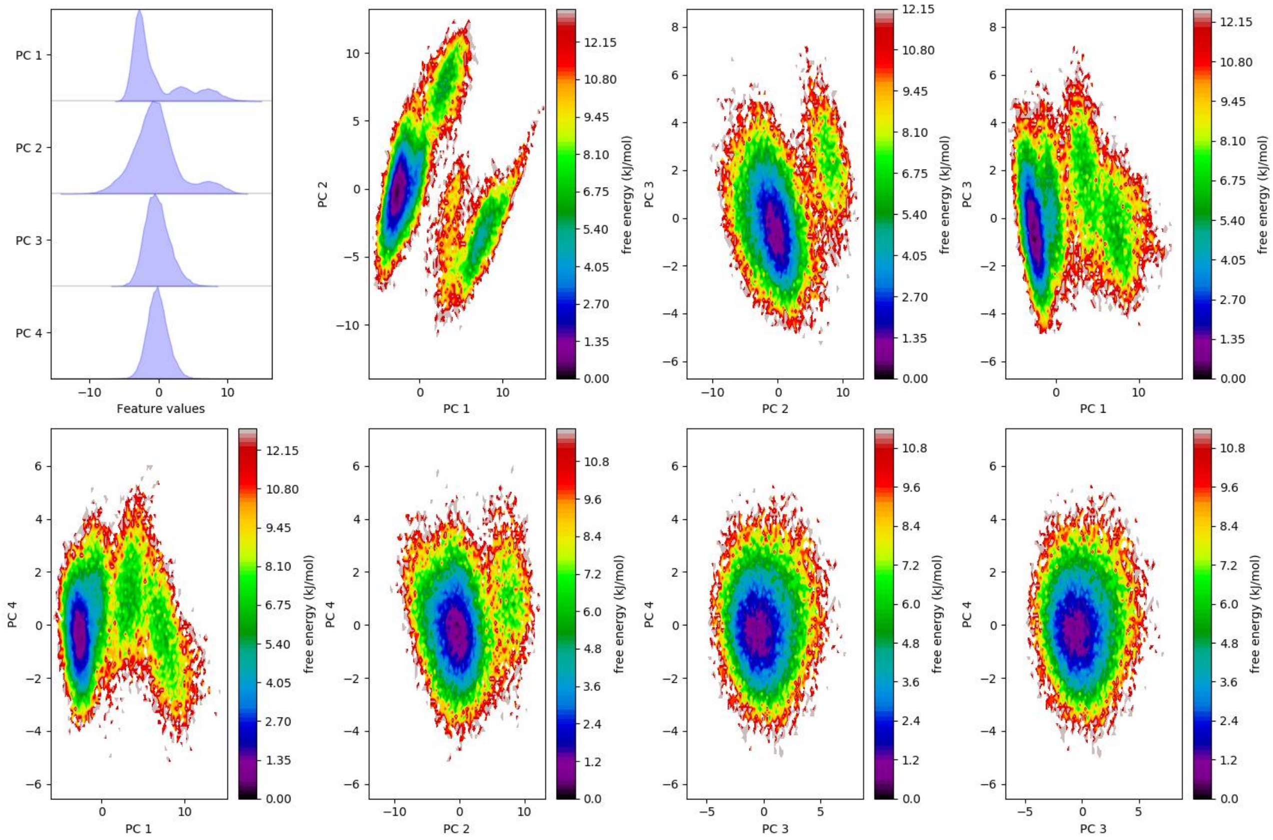

Figure S131 PCA projections between 1-4

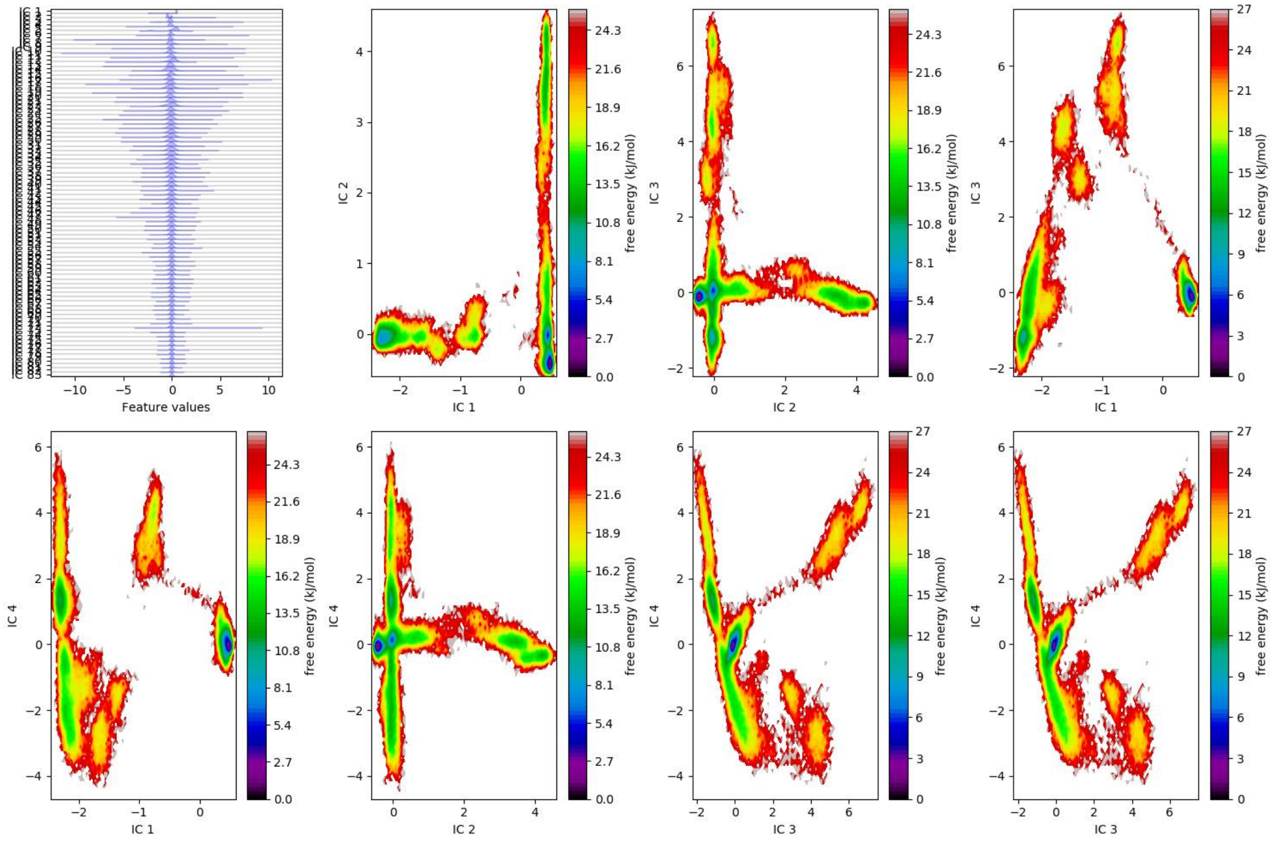

Figure S132 TICA projections between 1-4

M3 2

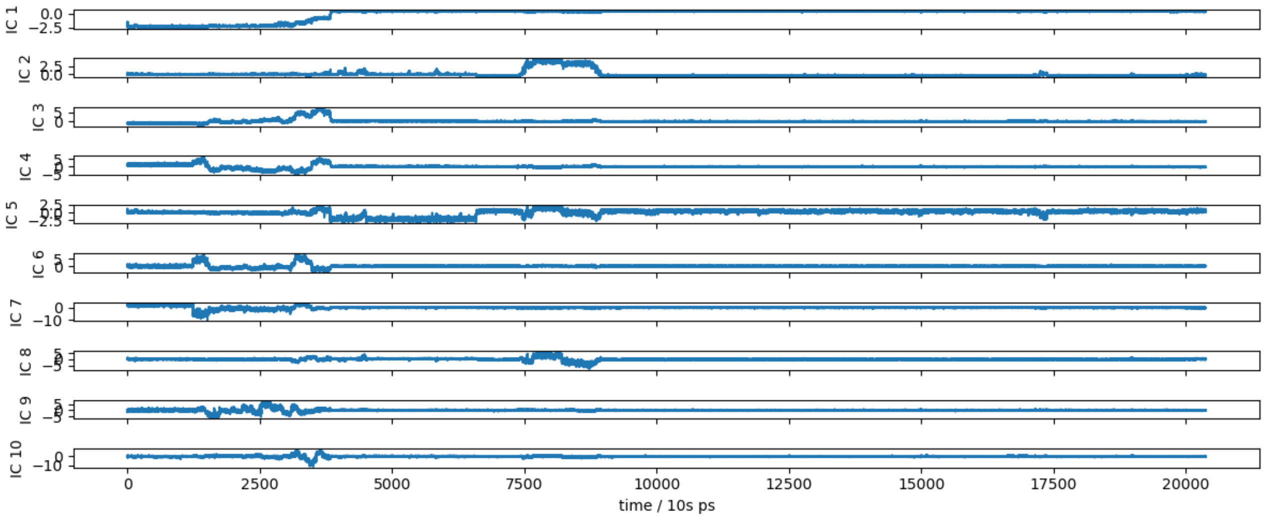

Figure S133 First 10 IC values over the simulation time

M3 3

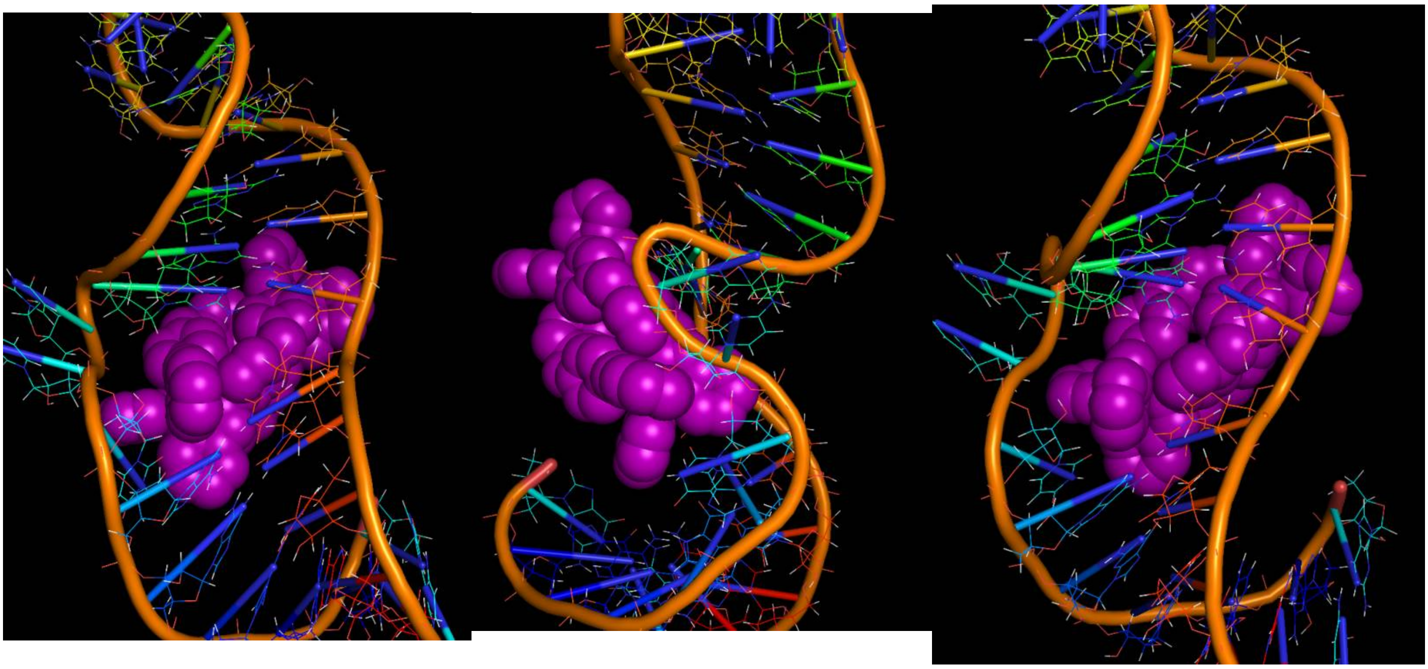

Figure S134 Stages of entering of the M cylinder in this run

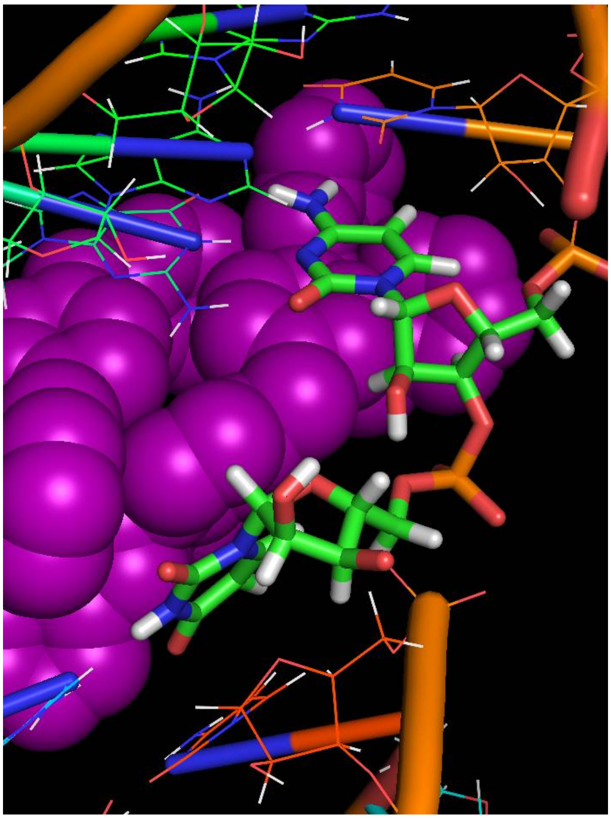

Figure S135 Intermediate splitting of the stacking of bases reminiscent of 3 way junction splitting

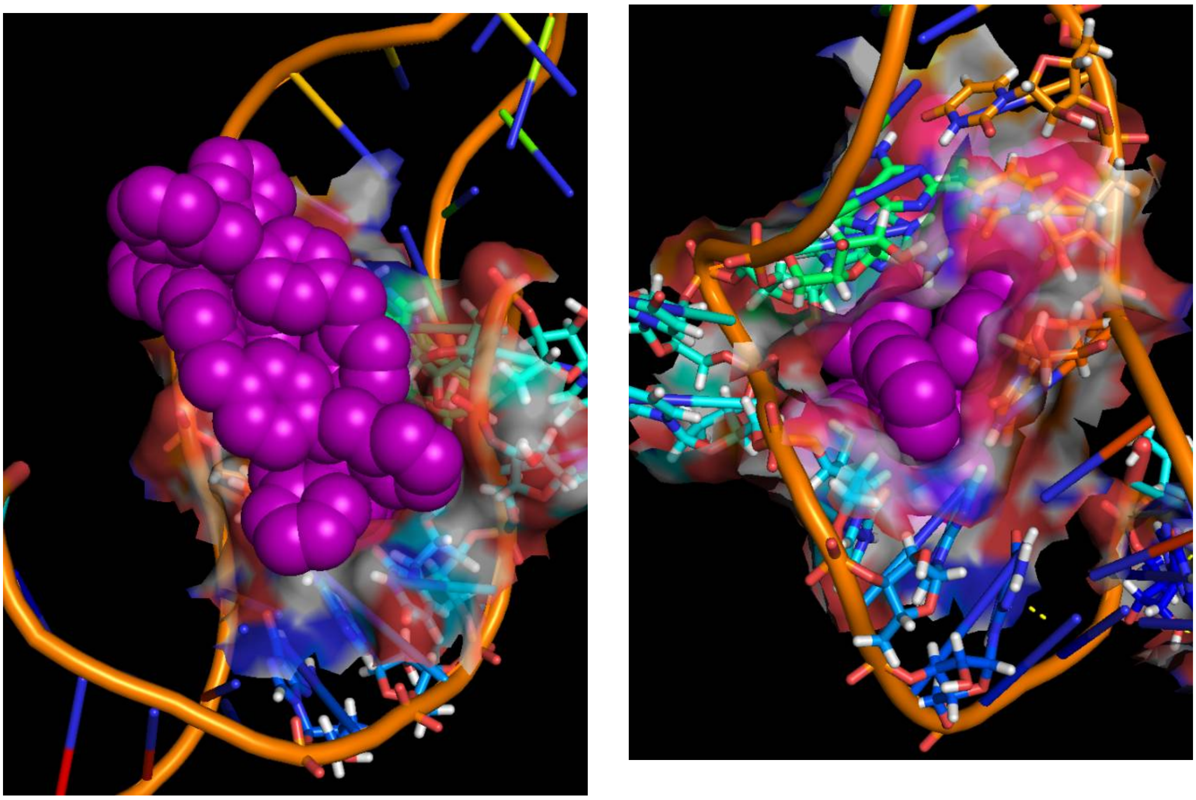

Figure S136 Extent of the overlapping surface between cylinder an opening of the bulge.

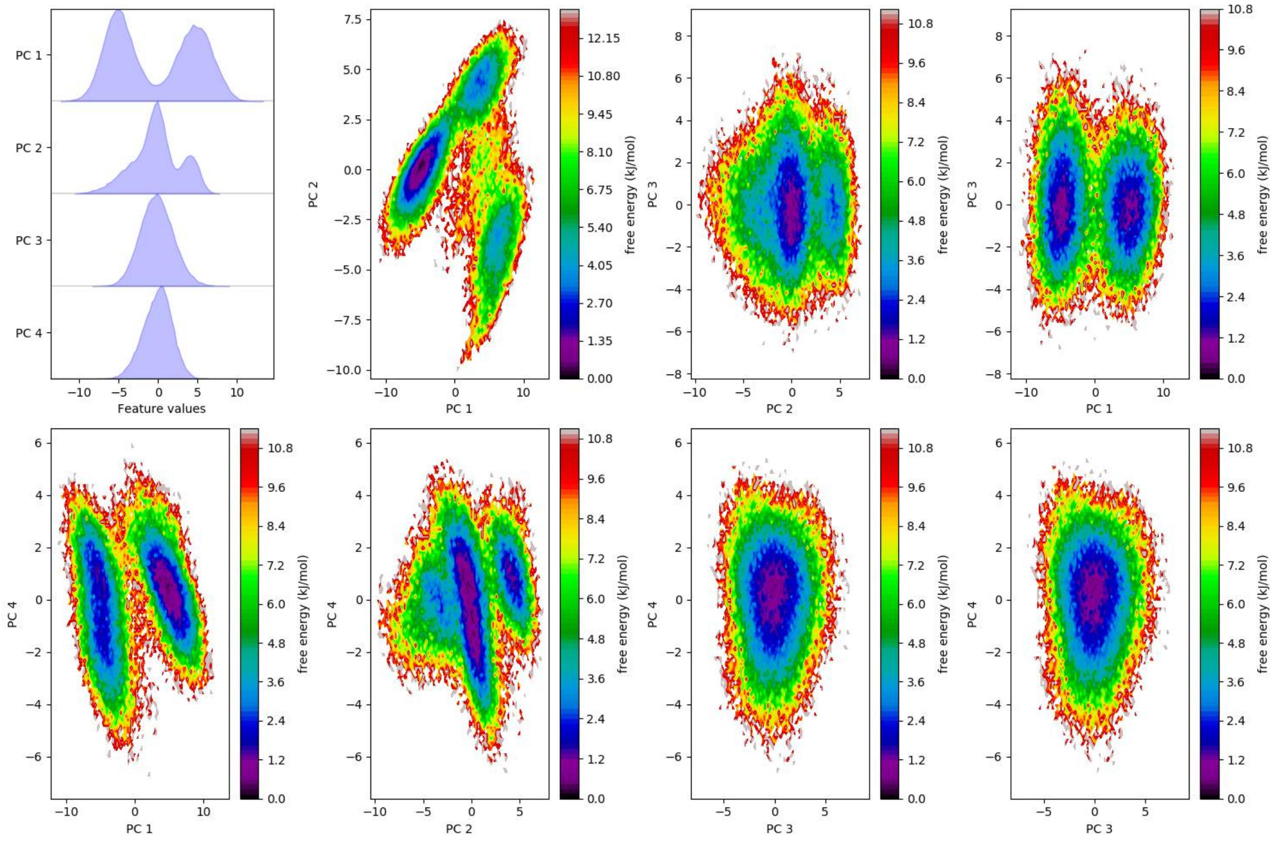

Figure S137 PCA projections between 1-4

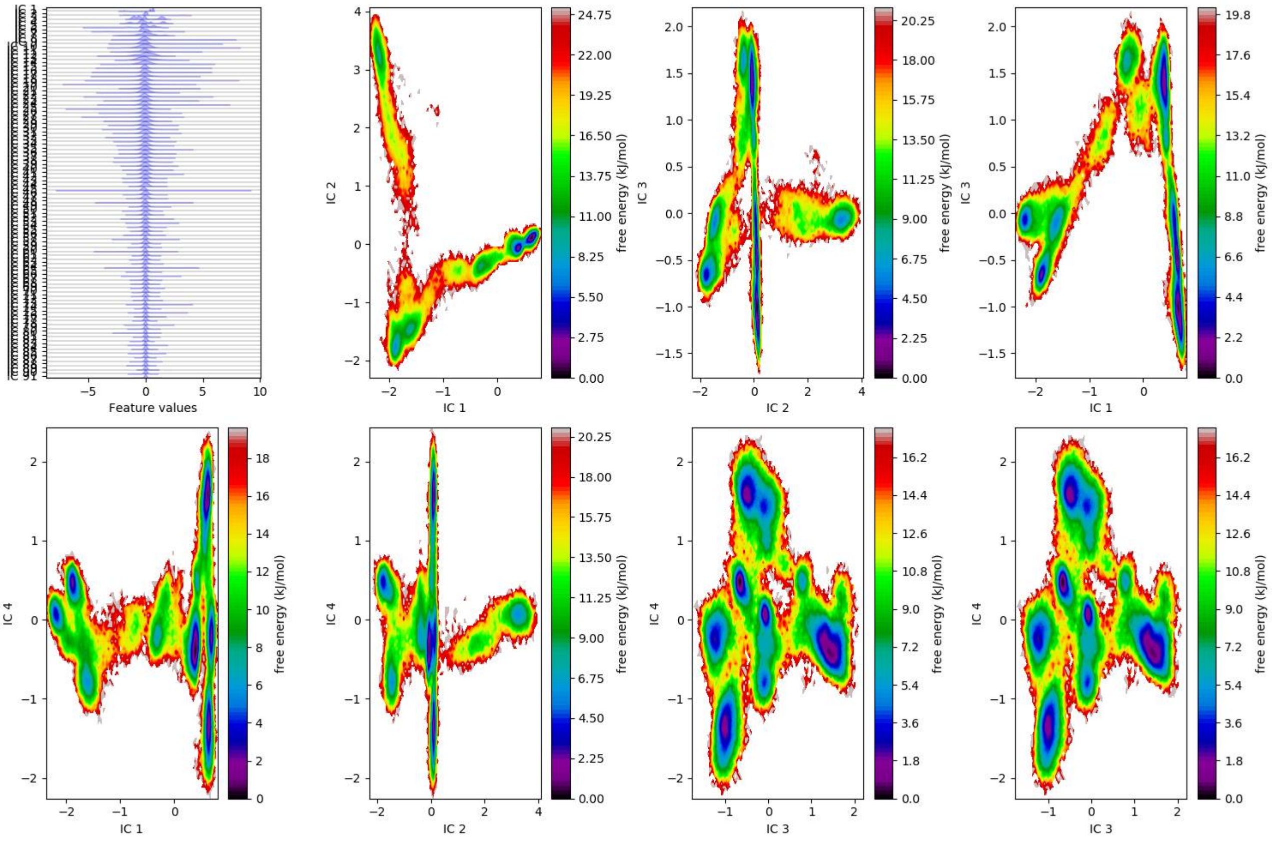

Figure S138 TICA projections between 1-4

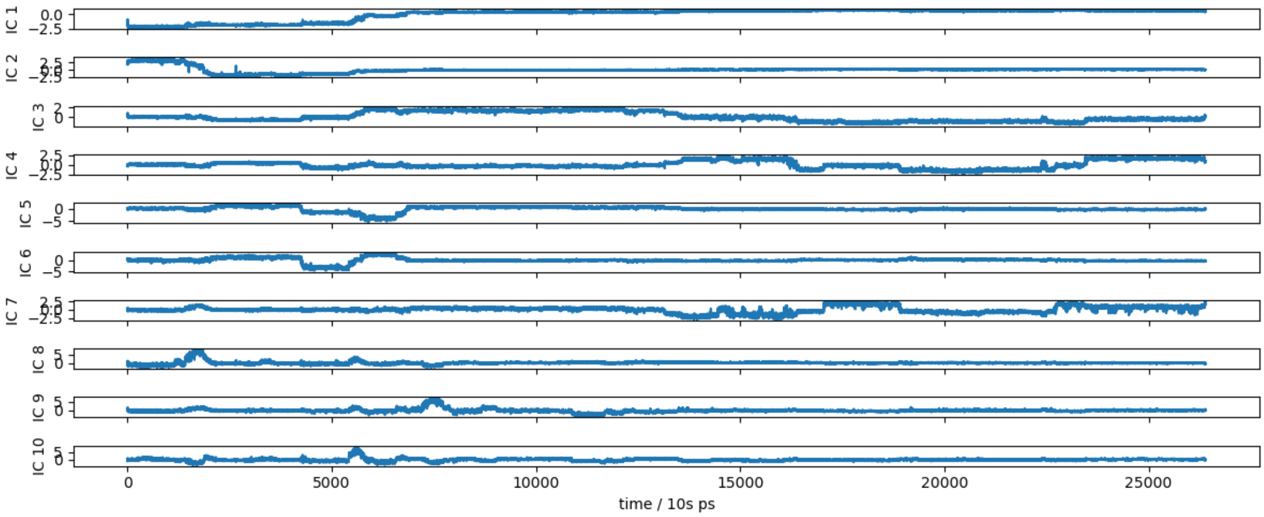

Figure S139 First 10 IC values over the simulation time

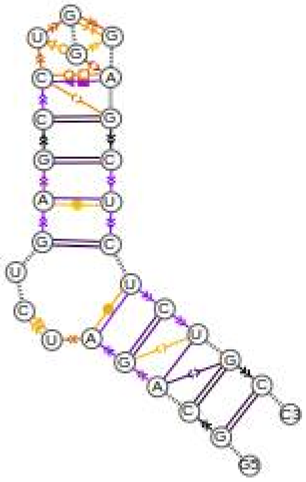

Figure S140 LW representation of the changes in base pairing and stacking in the presence of the cylinder in the bulge.

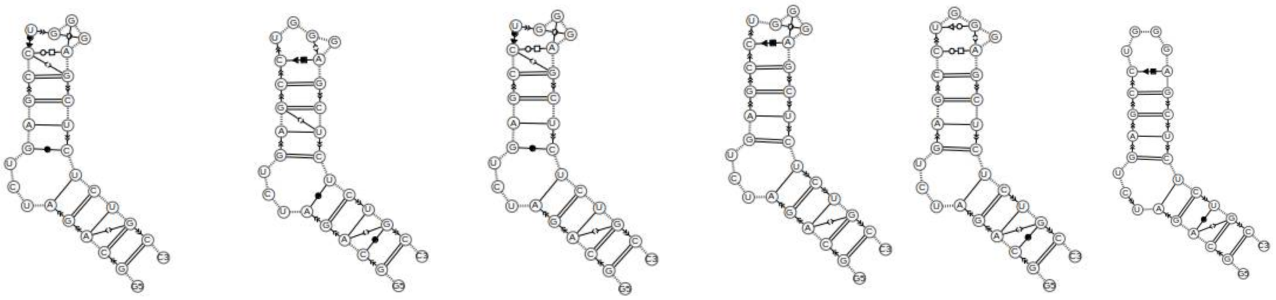

Figure S141 Regions of the TICA landscape and corresponding structure

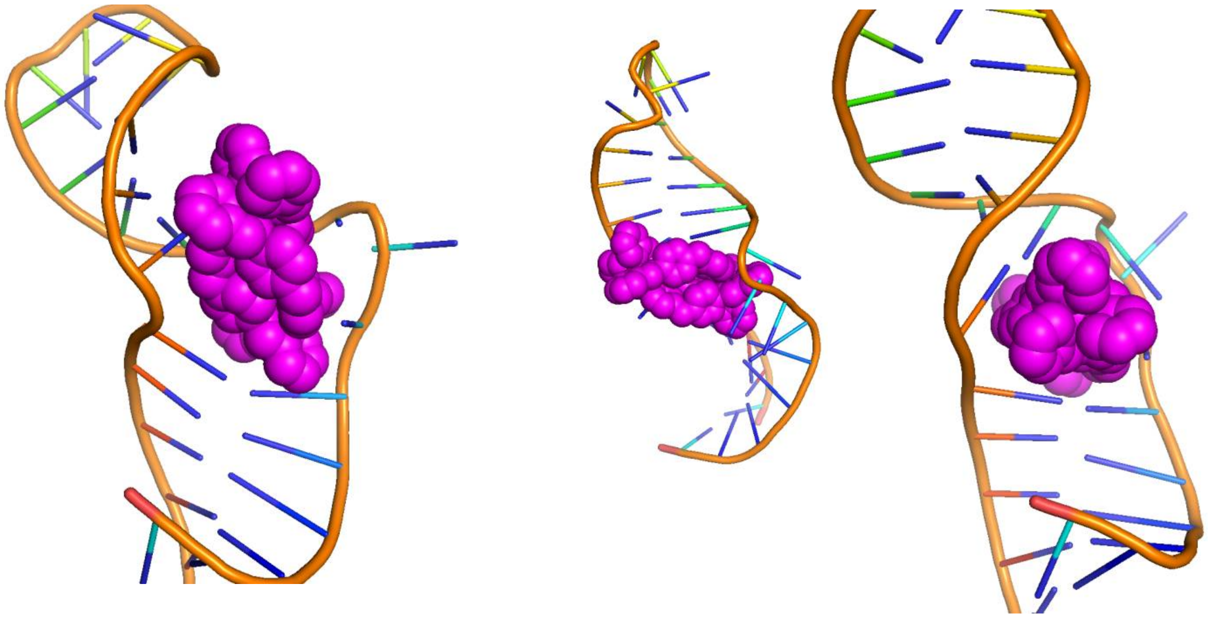

Figure S142 snapshots of the entering process

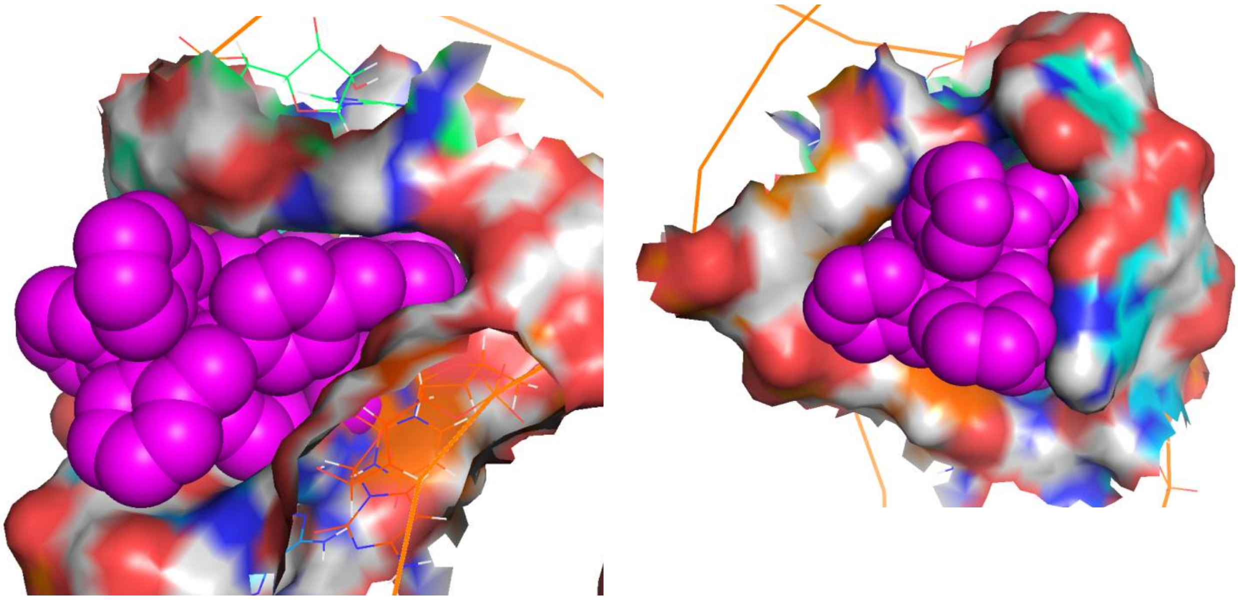

Figure S143 Surface of the created bulge pocket stabilised by the cylinder

state	π	G/kT
1	0.000025	10.592348
2	0.000039	10.164317
3	0.002197	6.120746
4	0.014848	4.209919
5	0.328236	1.114023
6	0.654656	0.423645

Table 8 Relative energy estimate of each state

M4

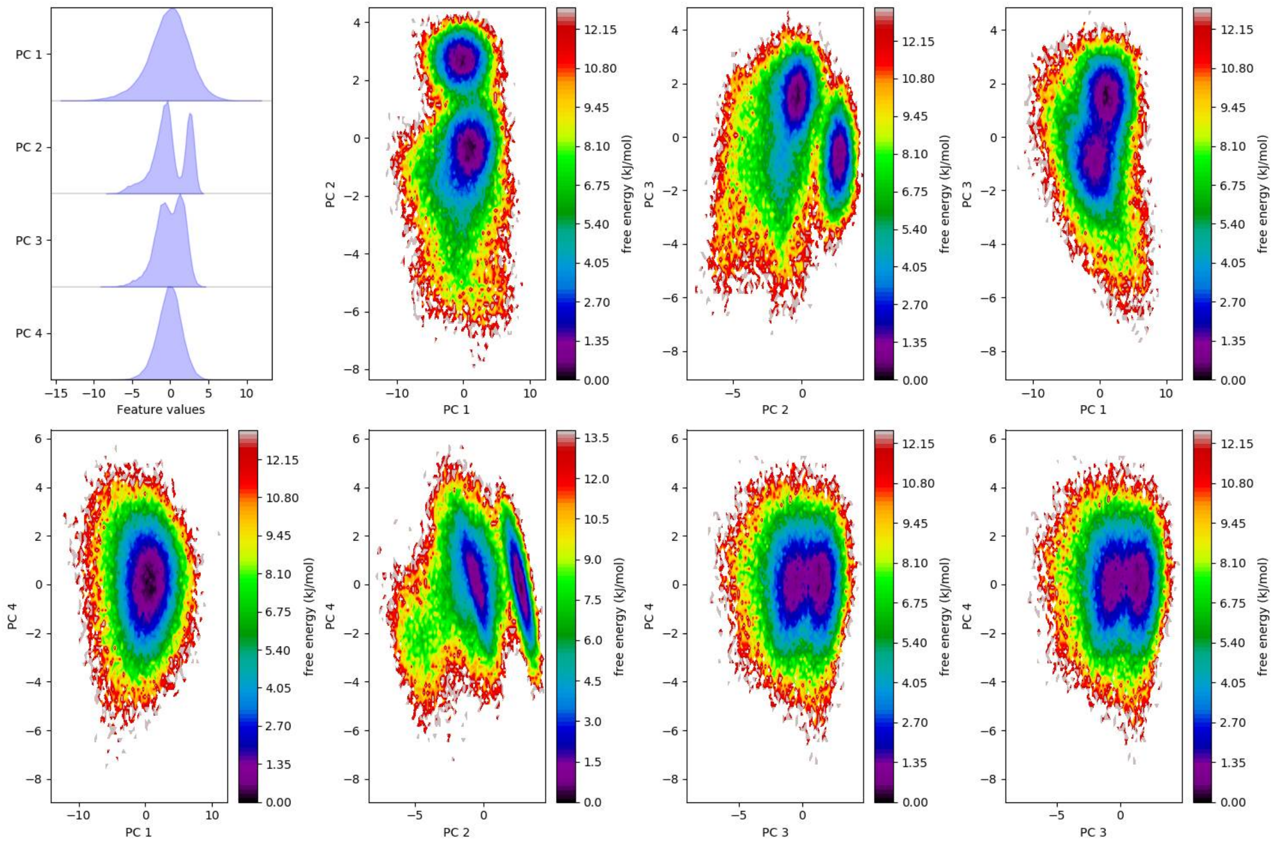

Figure S144 PCA projections between 1-4

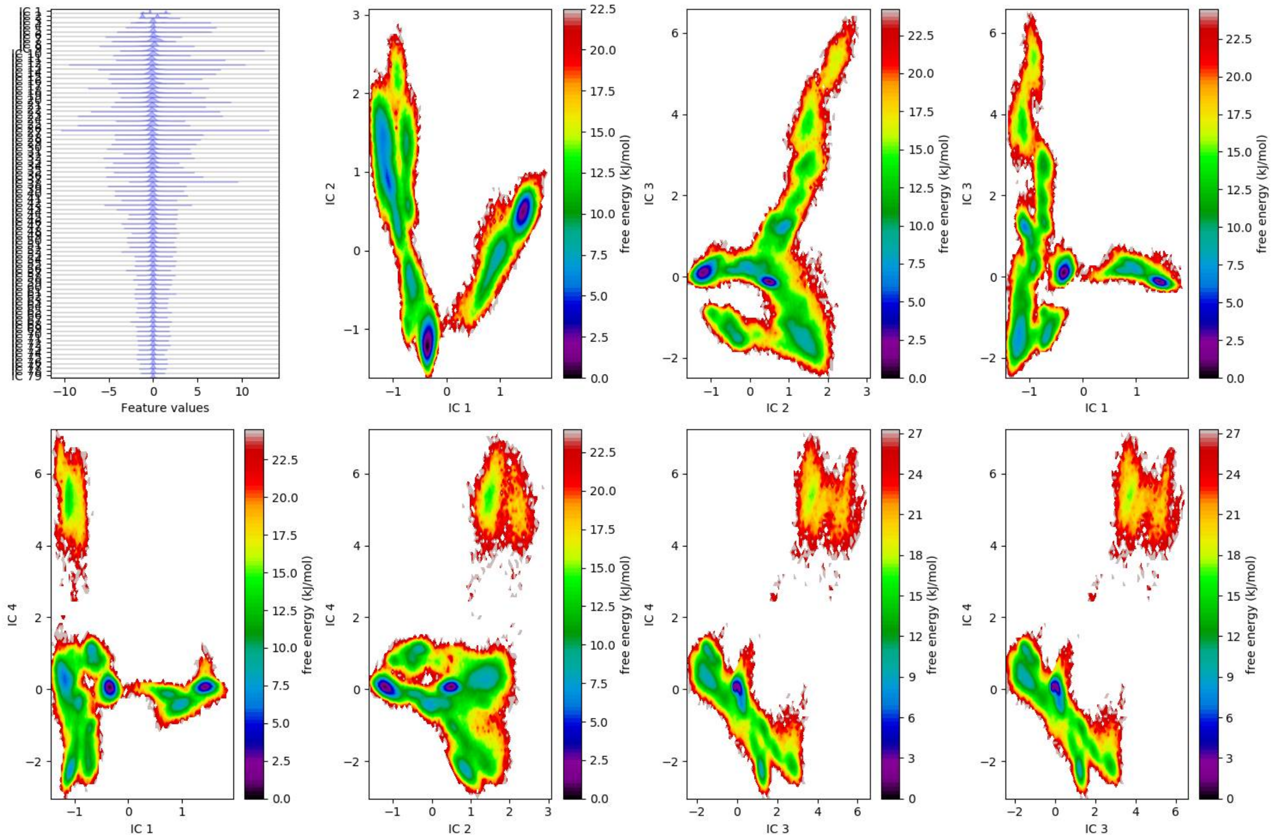

Figure S145 TICA projections between 1-4

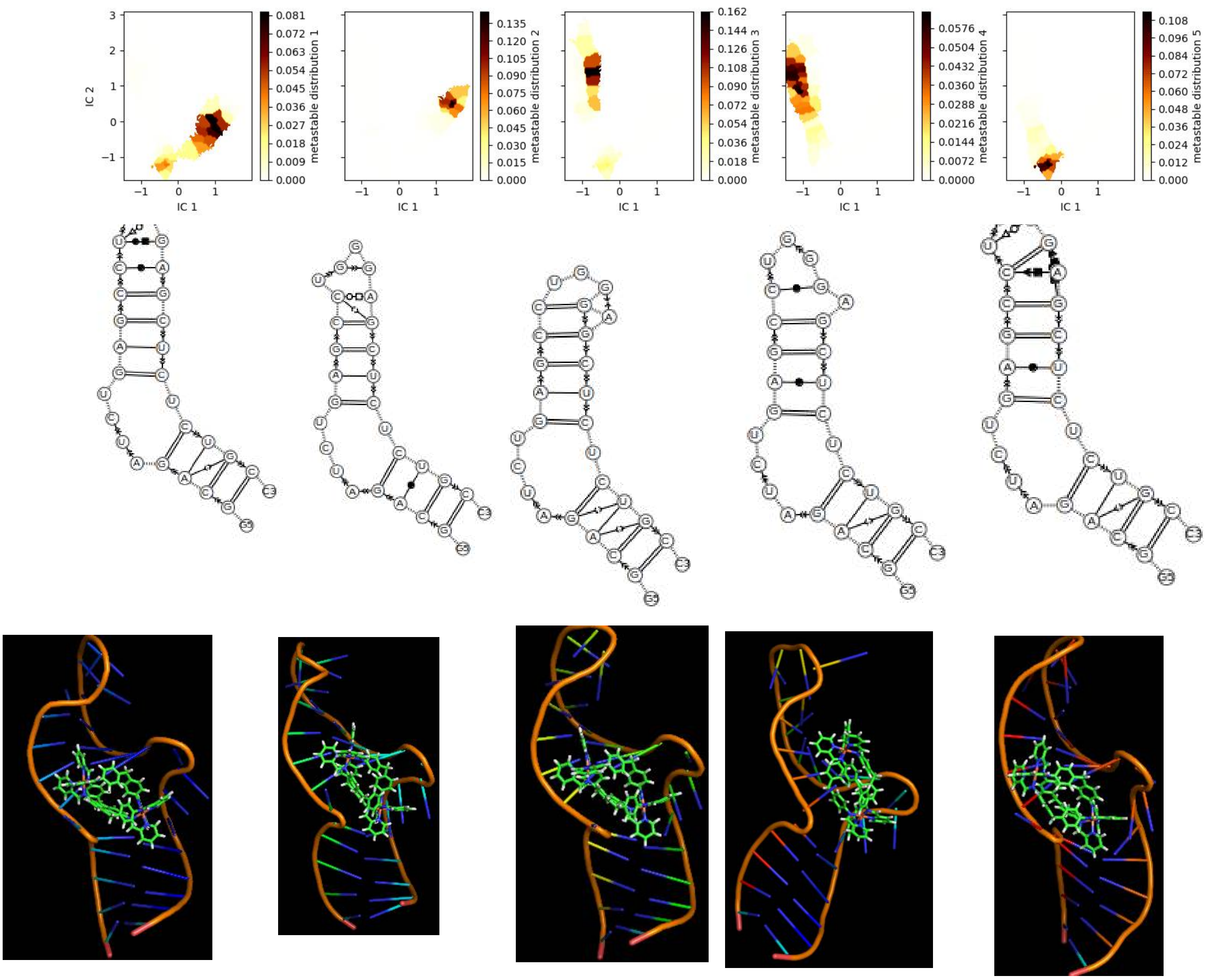

Figure S146 Regions of the TICA landscape and corresponding structure and 3D

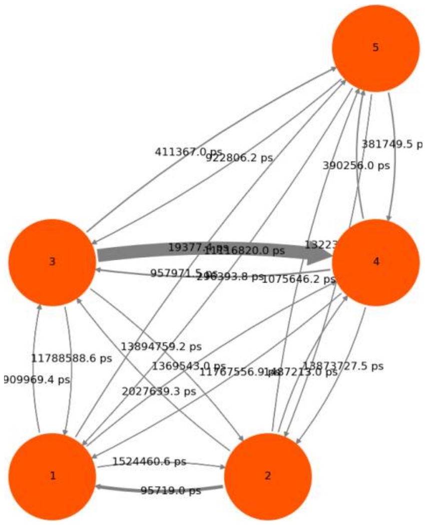

Figure S147 Representing network of transition probability between the above states.

P Collective

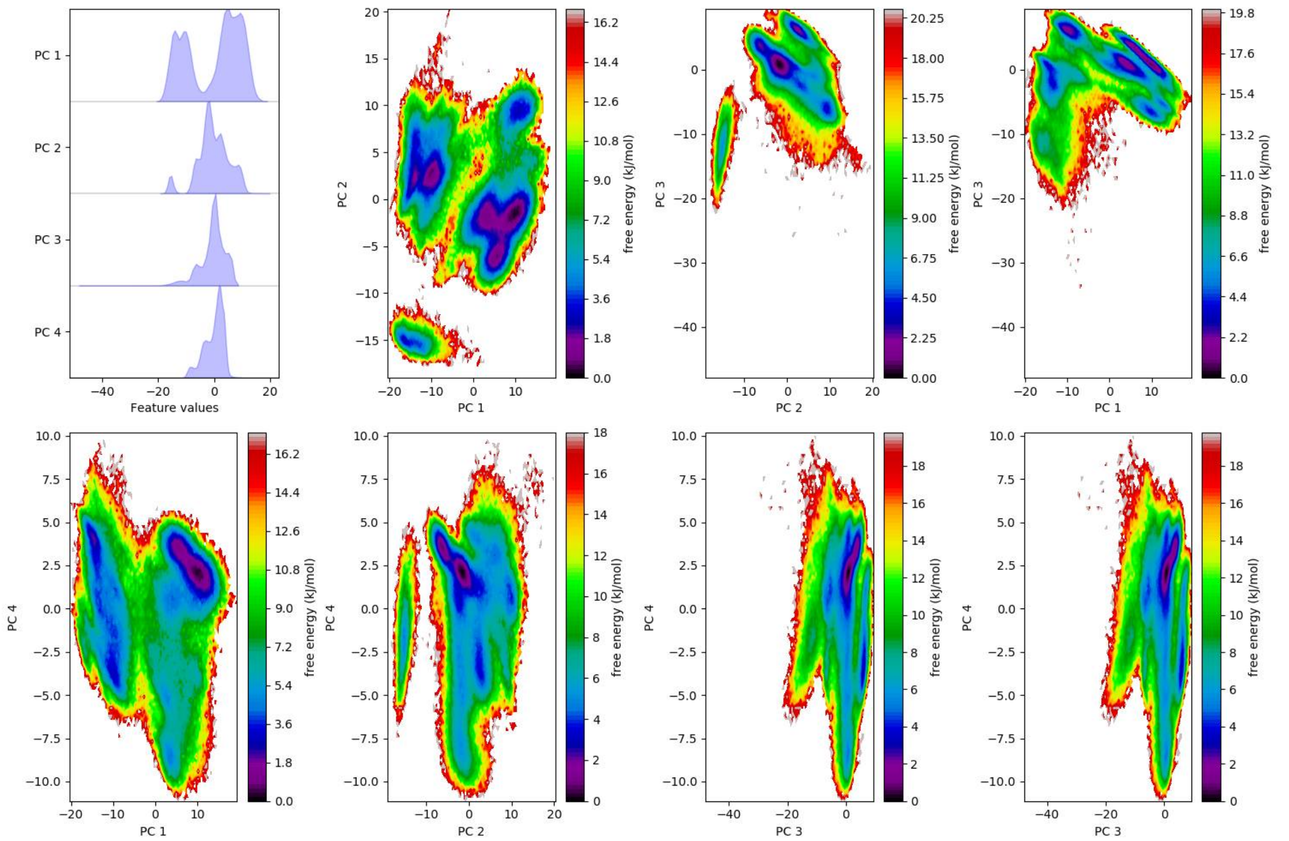

Figure S148 PCA projections between 1-4

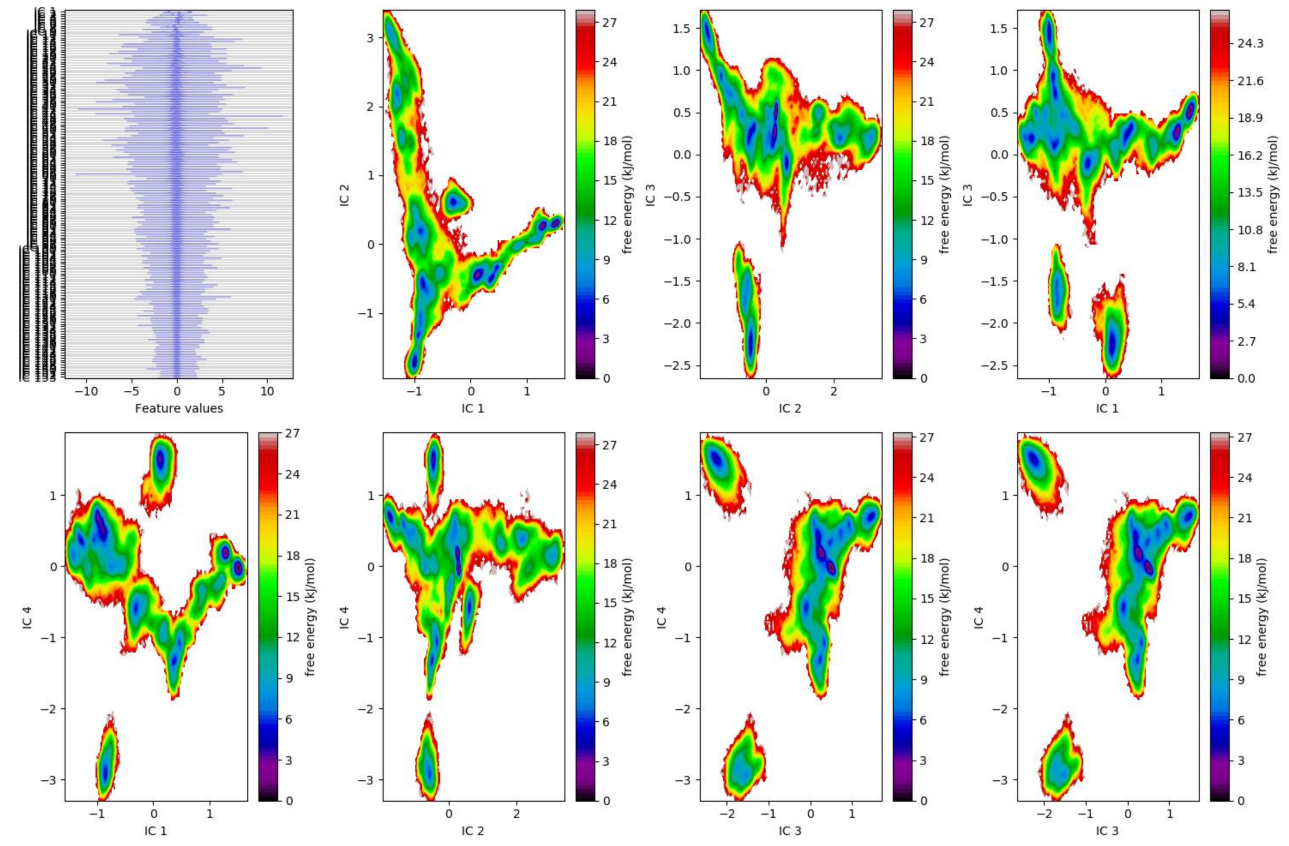

Figure S149 TICA projections between 1-4

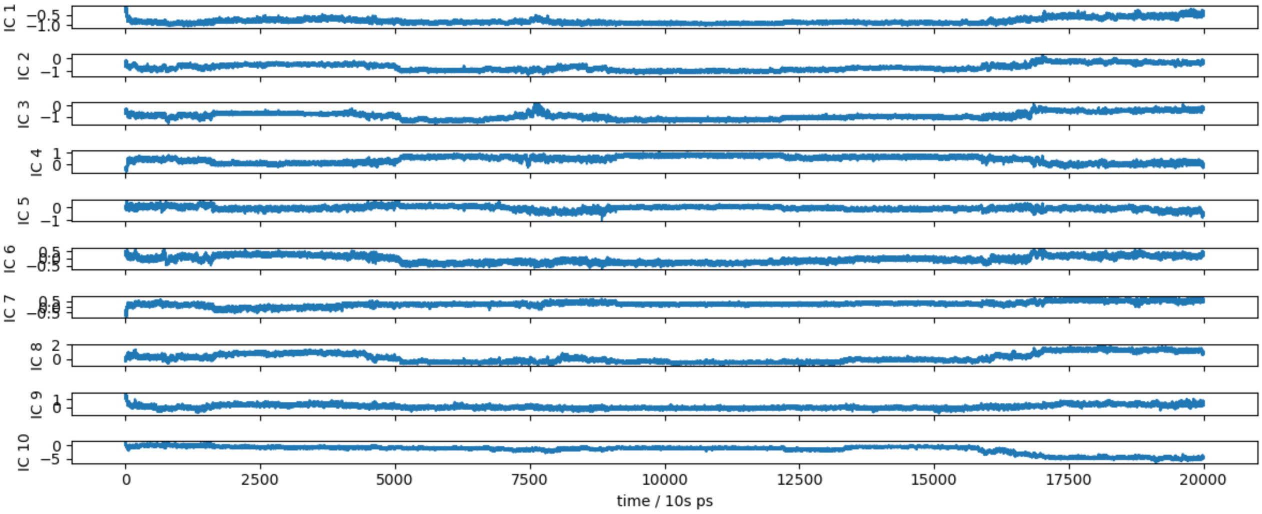

Figure S150 First 10 IC values over the simulation time

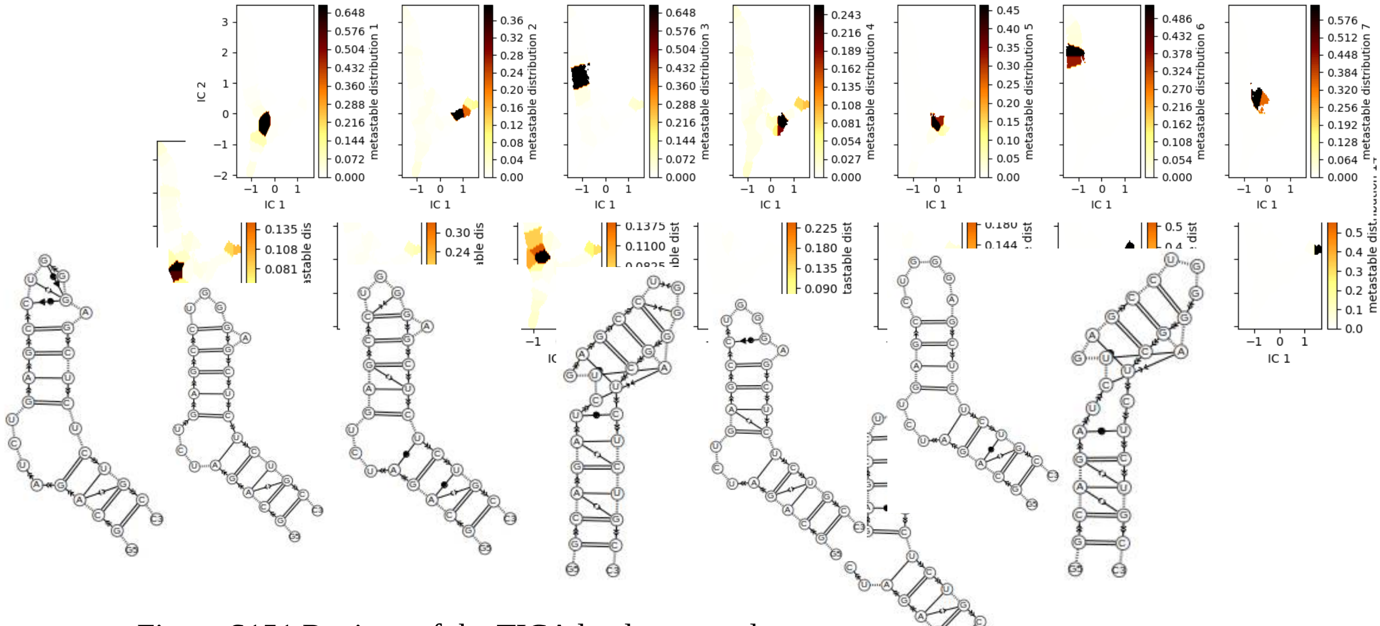

Figure S151 Regions of the TICA landscape and corresponding structure

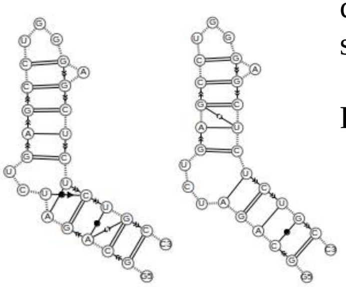

PC4

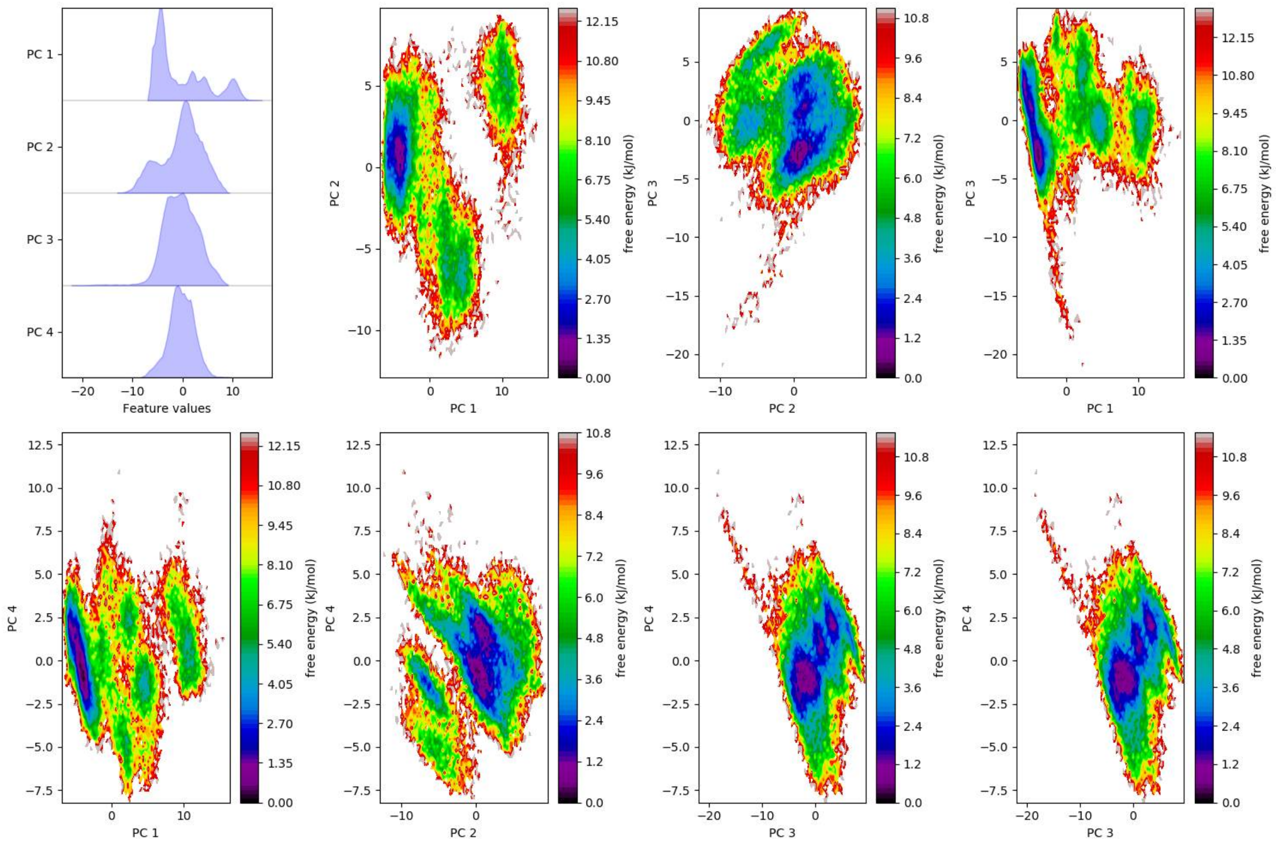

Figure S152 PCA projections between 1-4

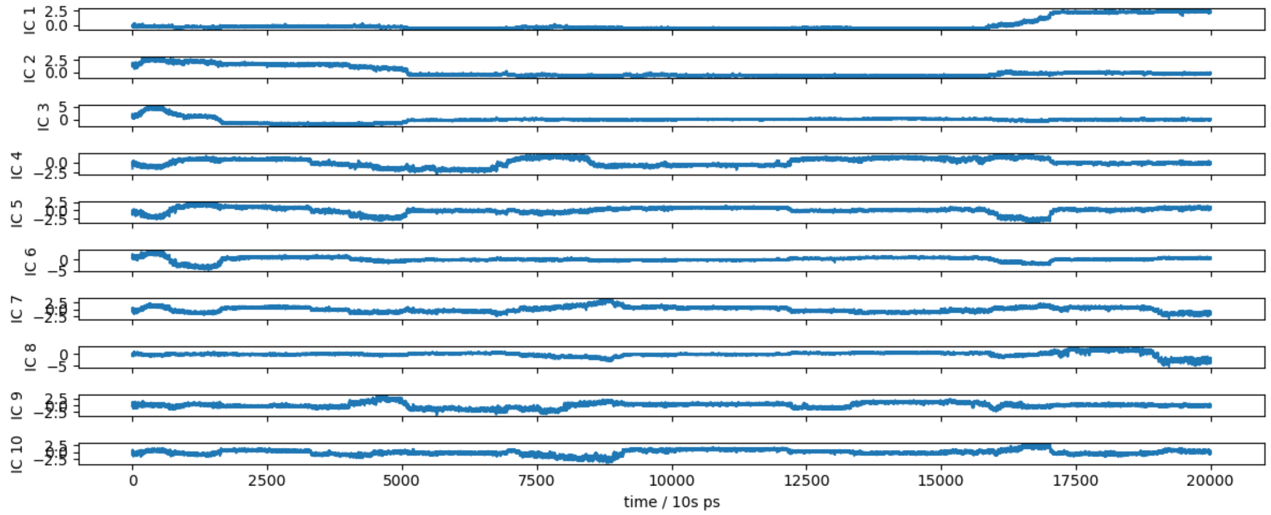

Figure S153 First 10 IC values over the simulation time

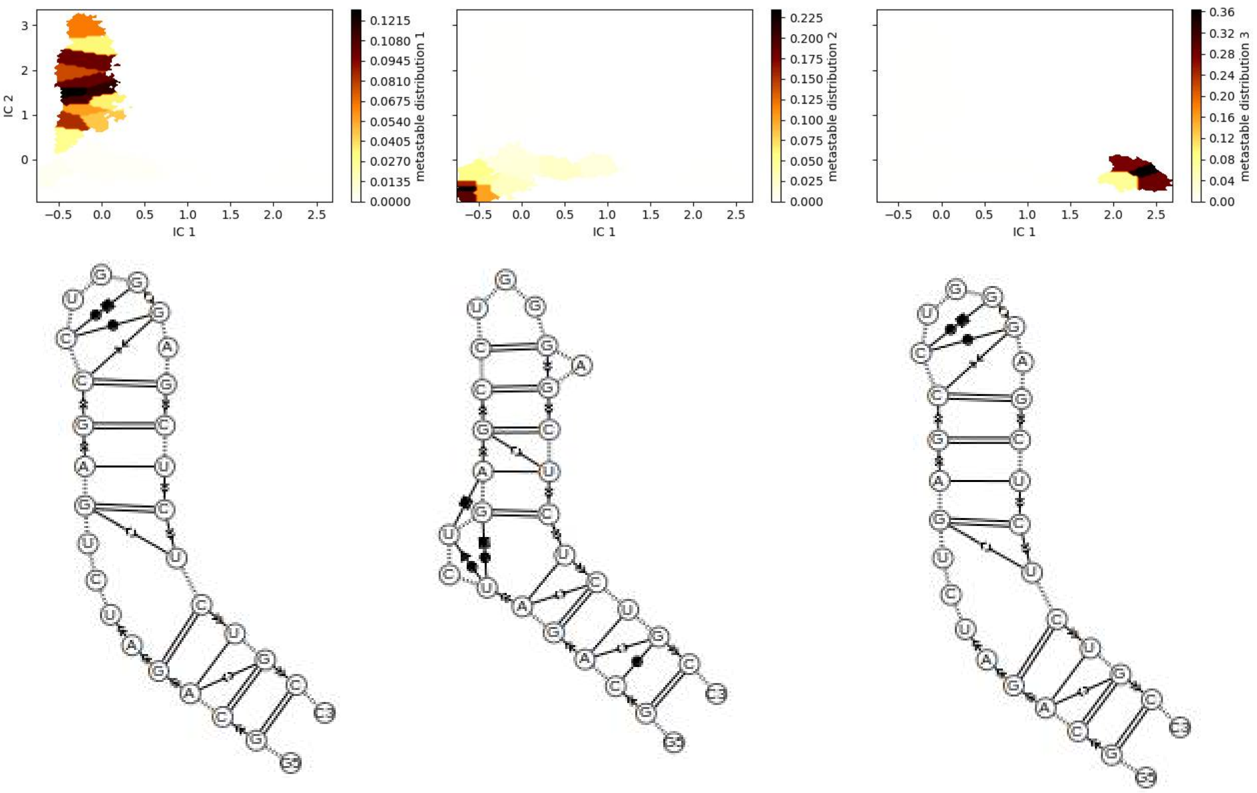

Figure S154 Regions of the TICA landscape and corresponding structure

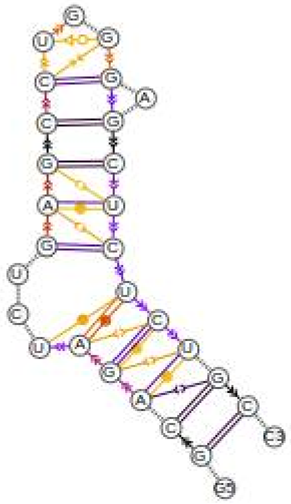

Figure S155 summary of base interaction with the P cylinder in the bulge pocket.

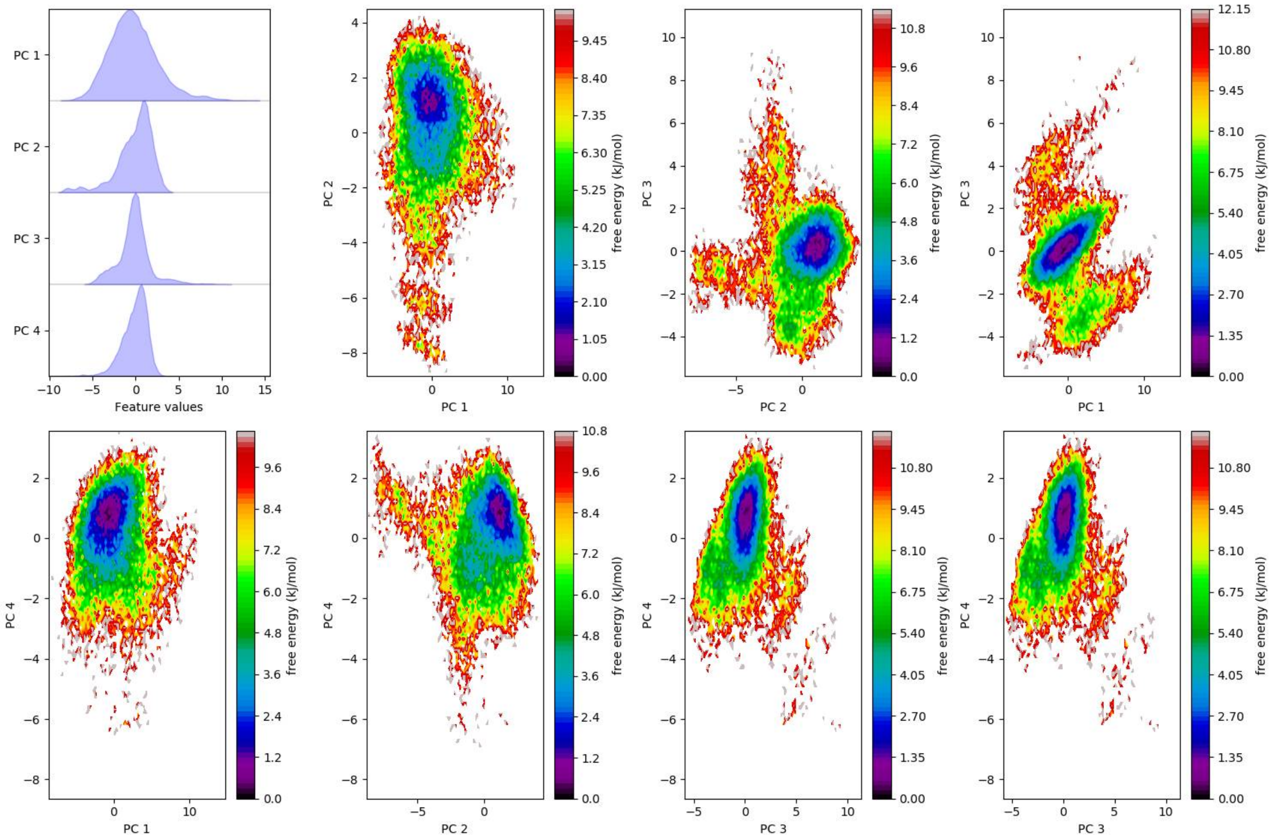

Figure S156 PCA projections between 1-4

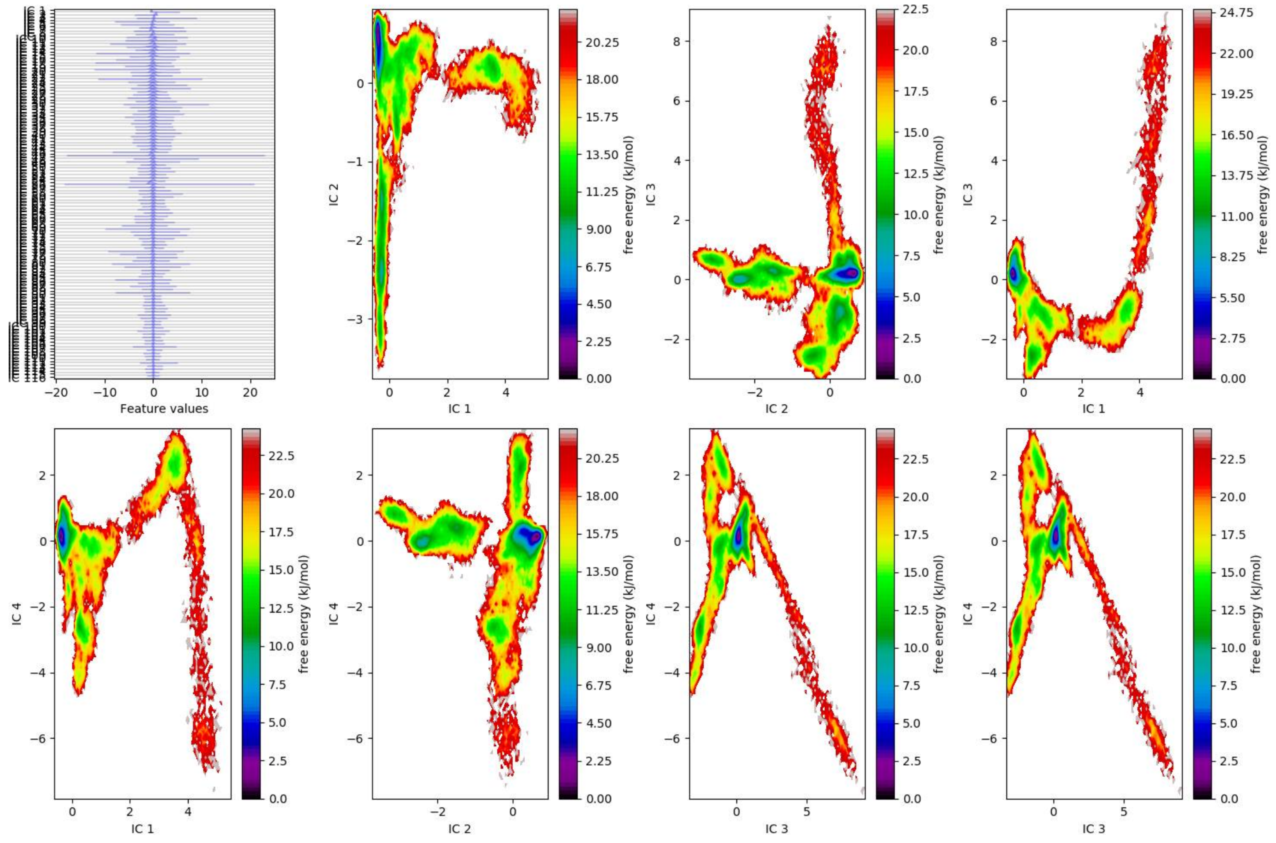

Figure S157 TICA projections between 1-4

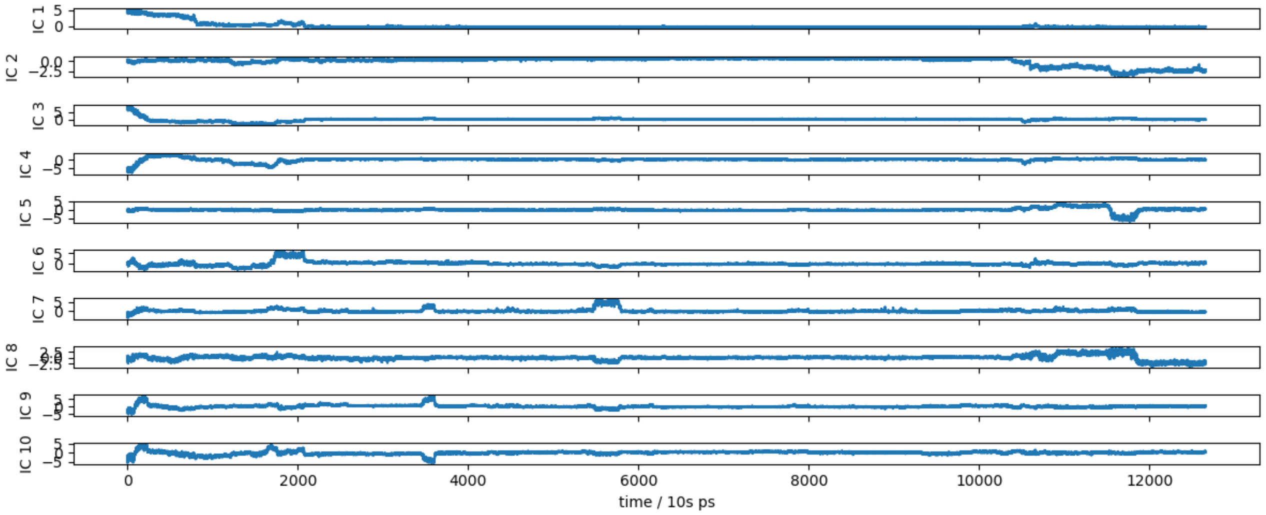

Figure S158 First 10 IC values over the simulation time

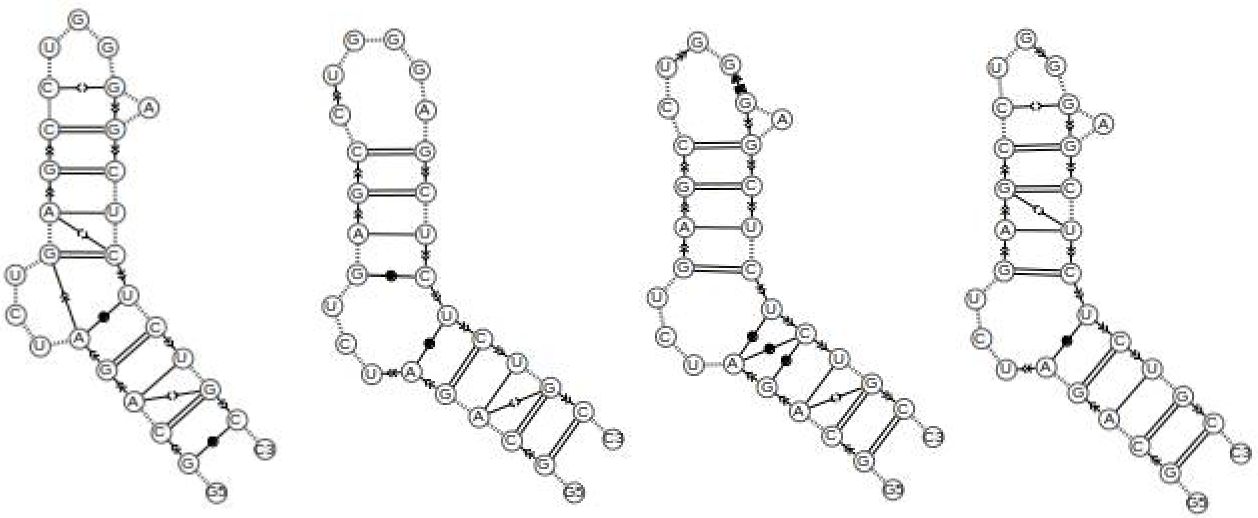

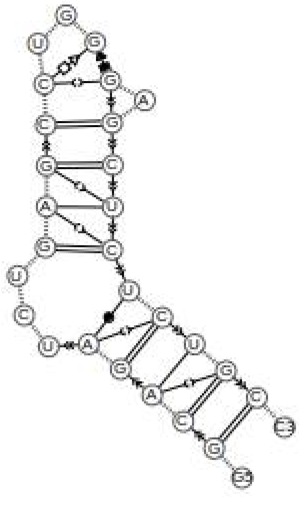

Figure S159 Regions of the TICA landscape and corresponding structure

Figure S160 summary of base interaction with the P cylinder in the bulge pocket

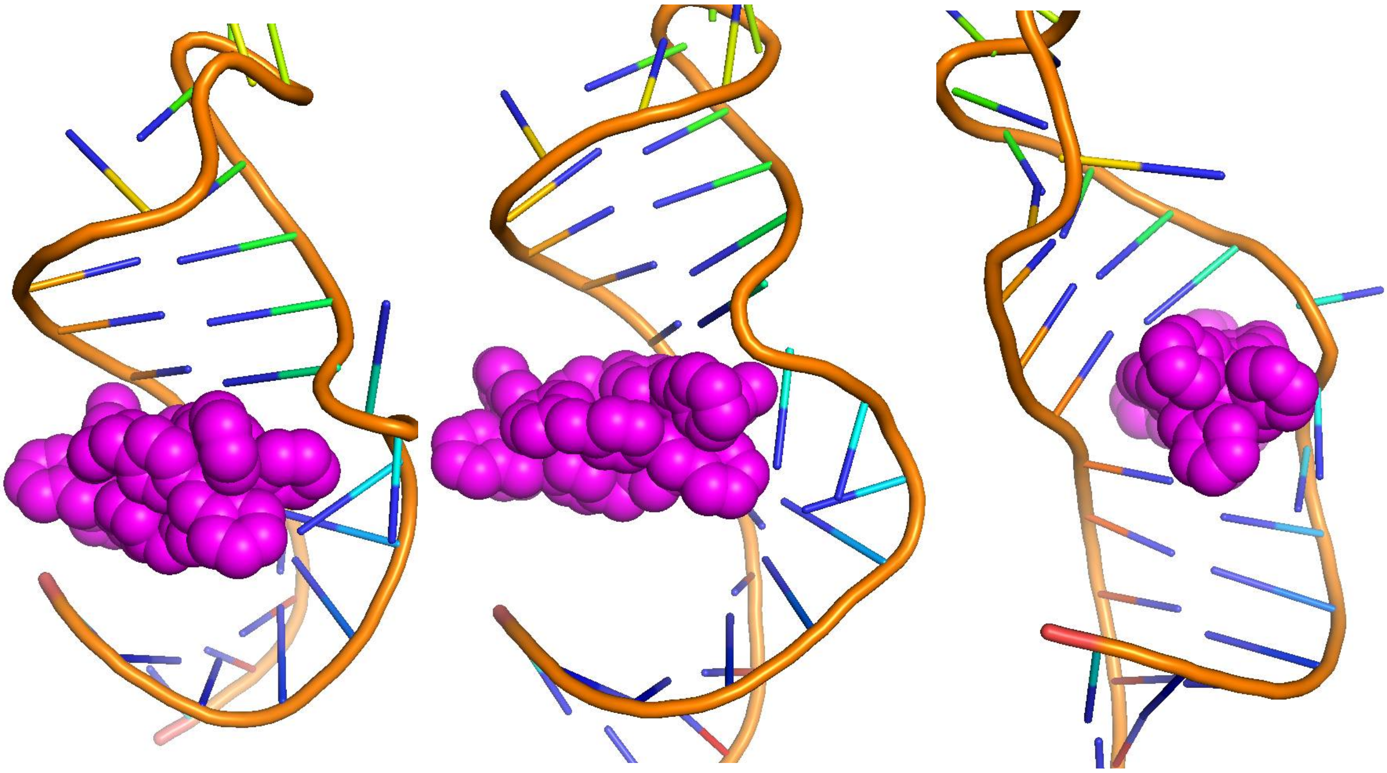

Figure S161 entering process for the P cylinder

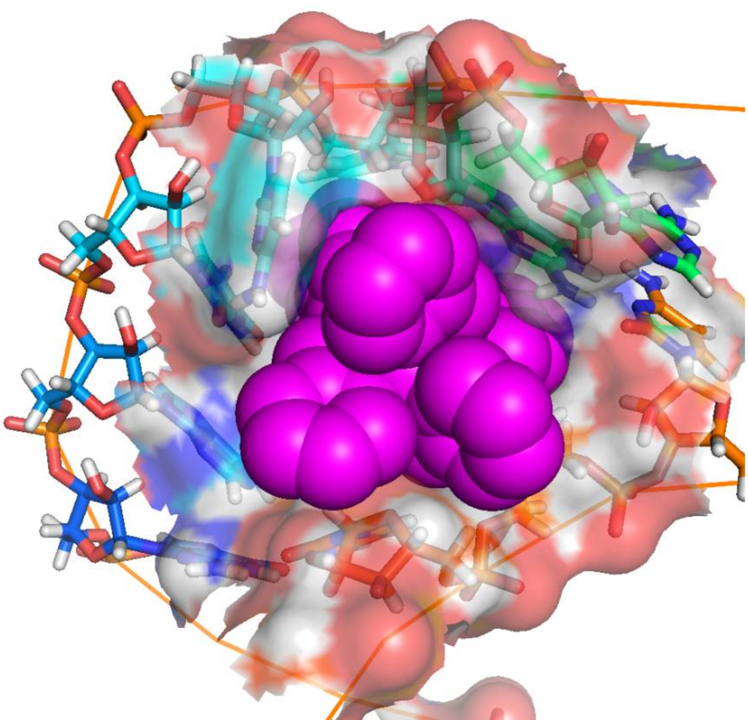

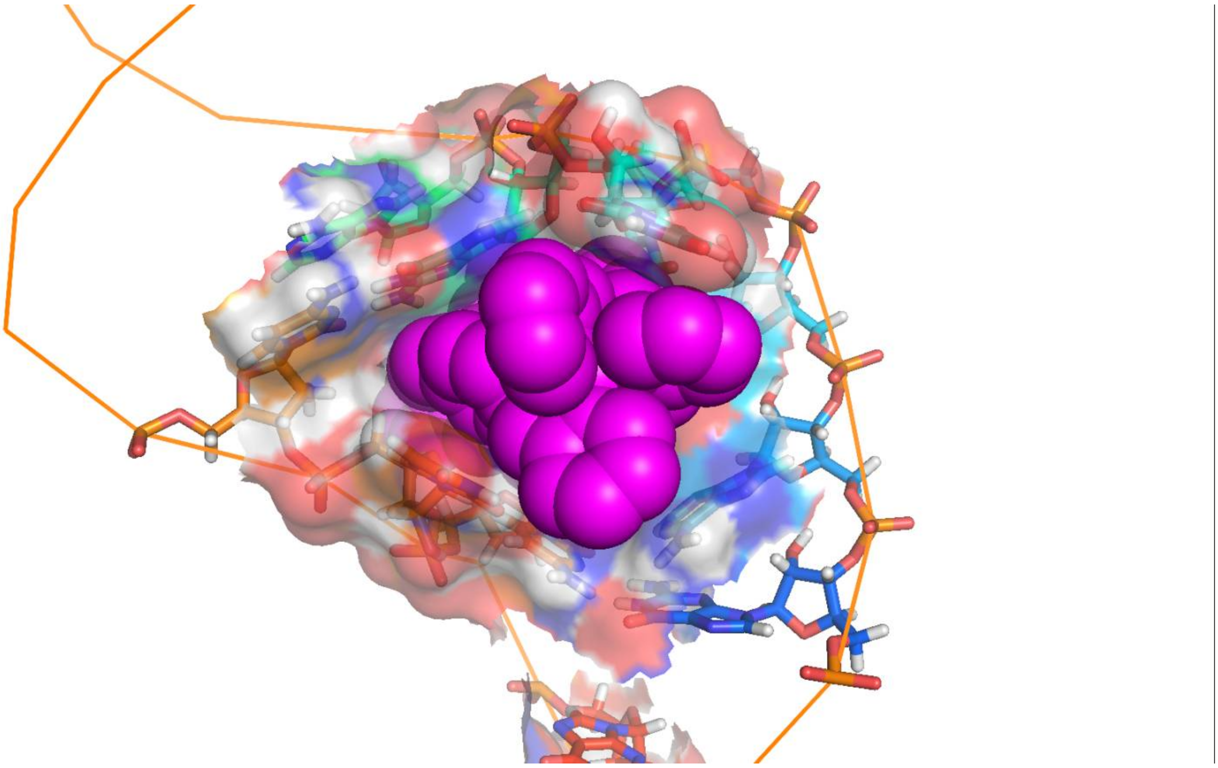

Figure S162 Surface and stacking of the bulge bases around the P cylinder.

P3

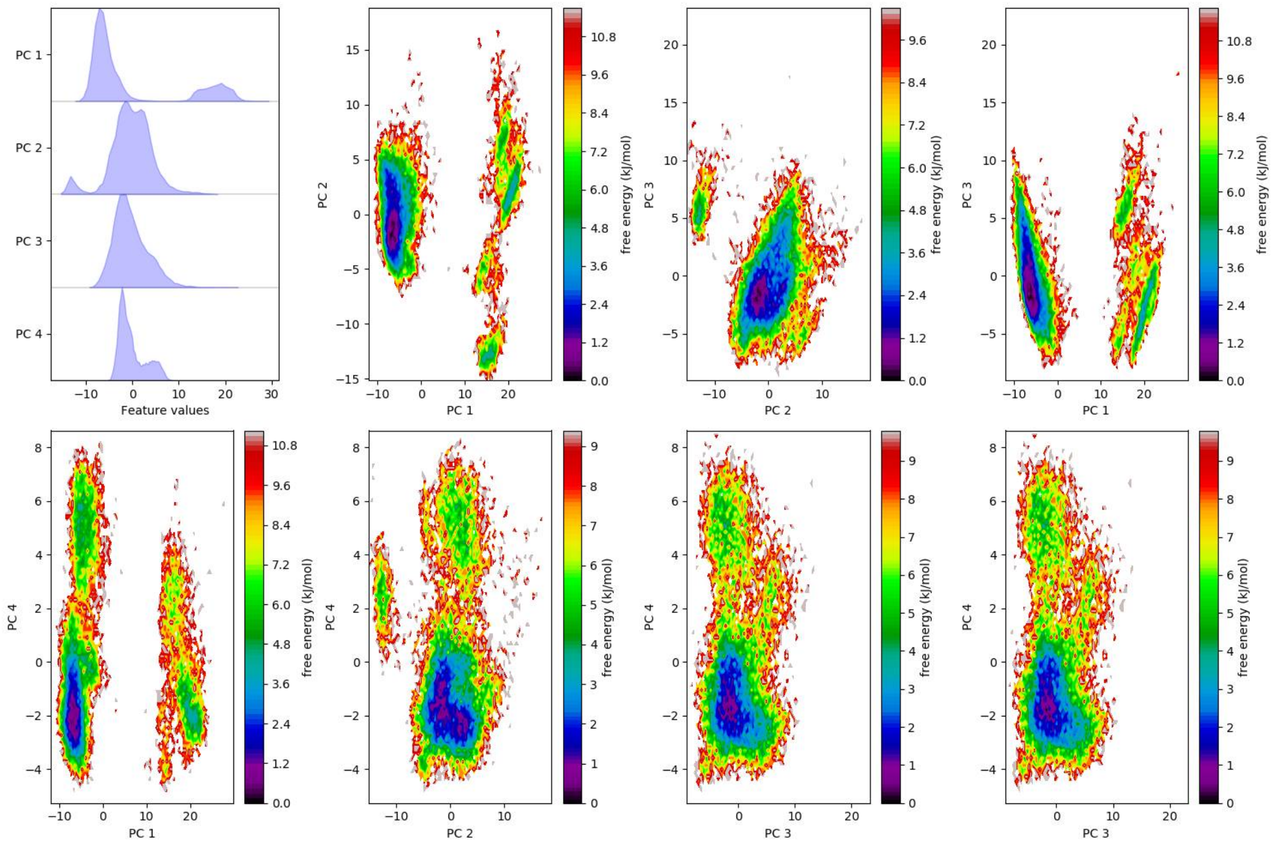

Figure S163 PCA projections between 1-4

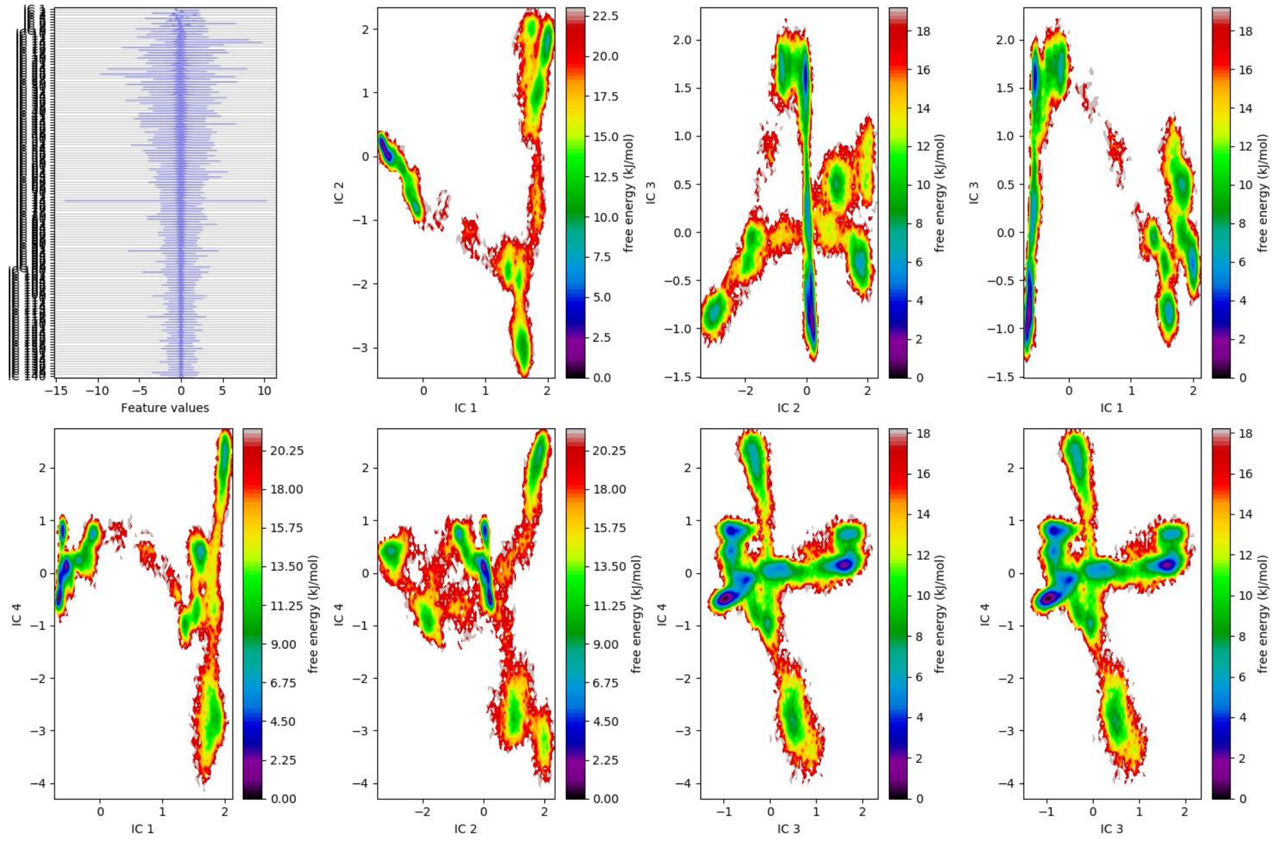

Figure S164 TICA projections between 1-4

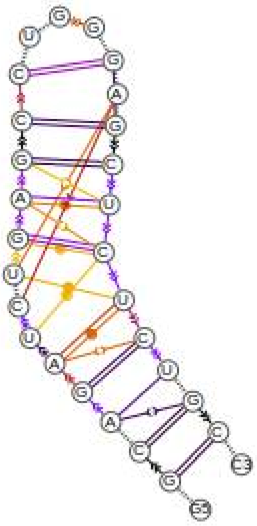

Figure S165 Stacking and base-pairings with the cylinder capping the terminal bases.

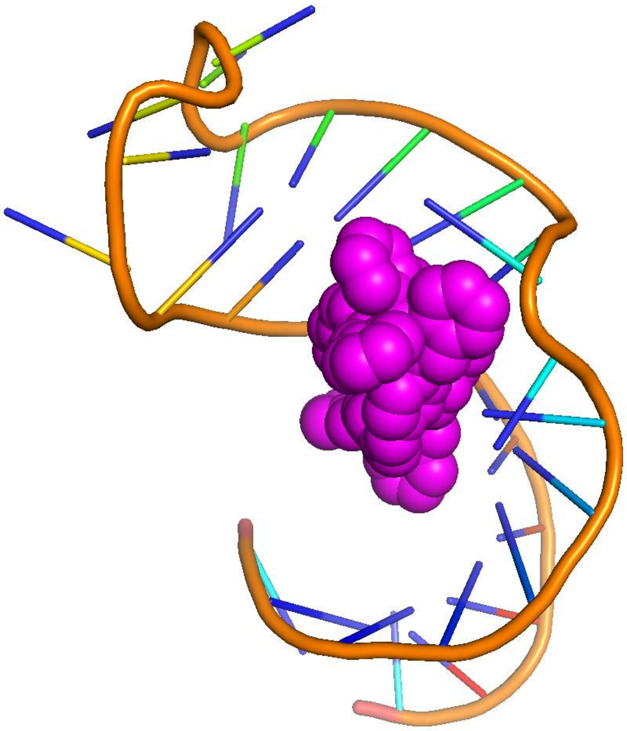

Figure S166 initial starting position of the simulation

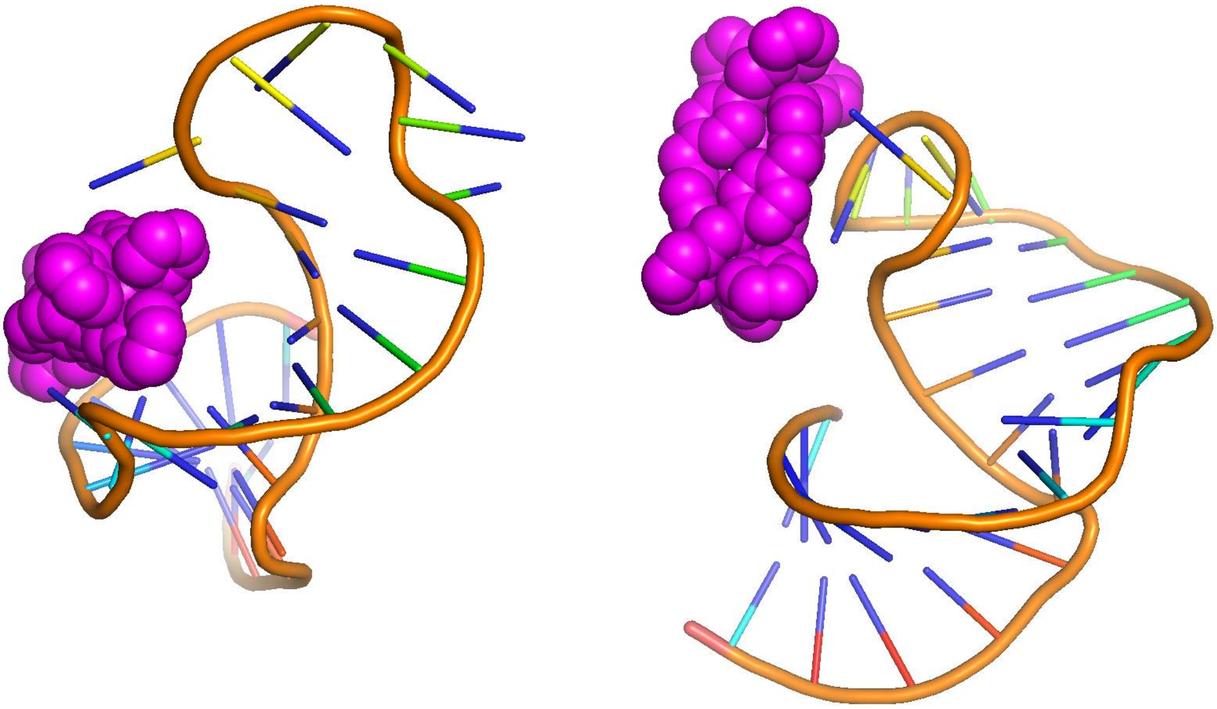

Figure S167 Intermediate binding events

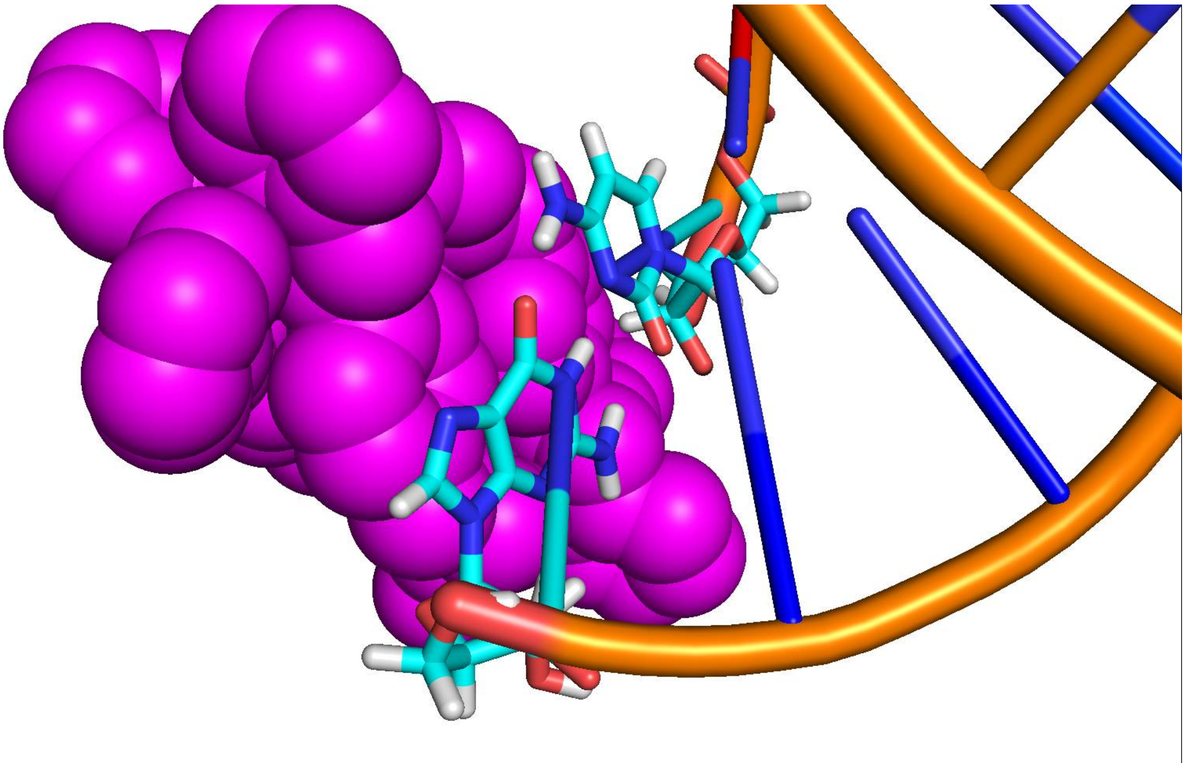

Figure S168 Stable binding of the P cylinder to the terminal bases.

P4

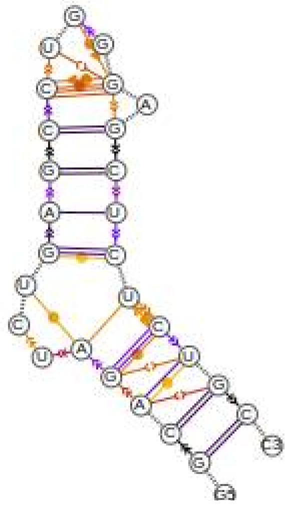

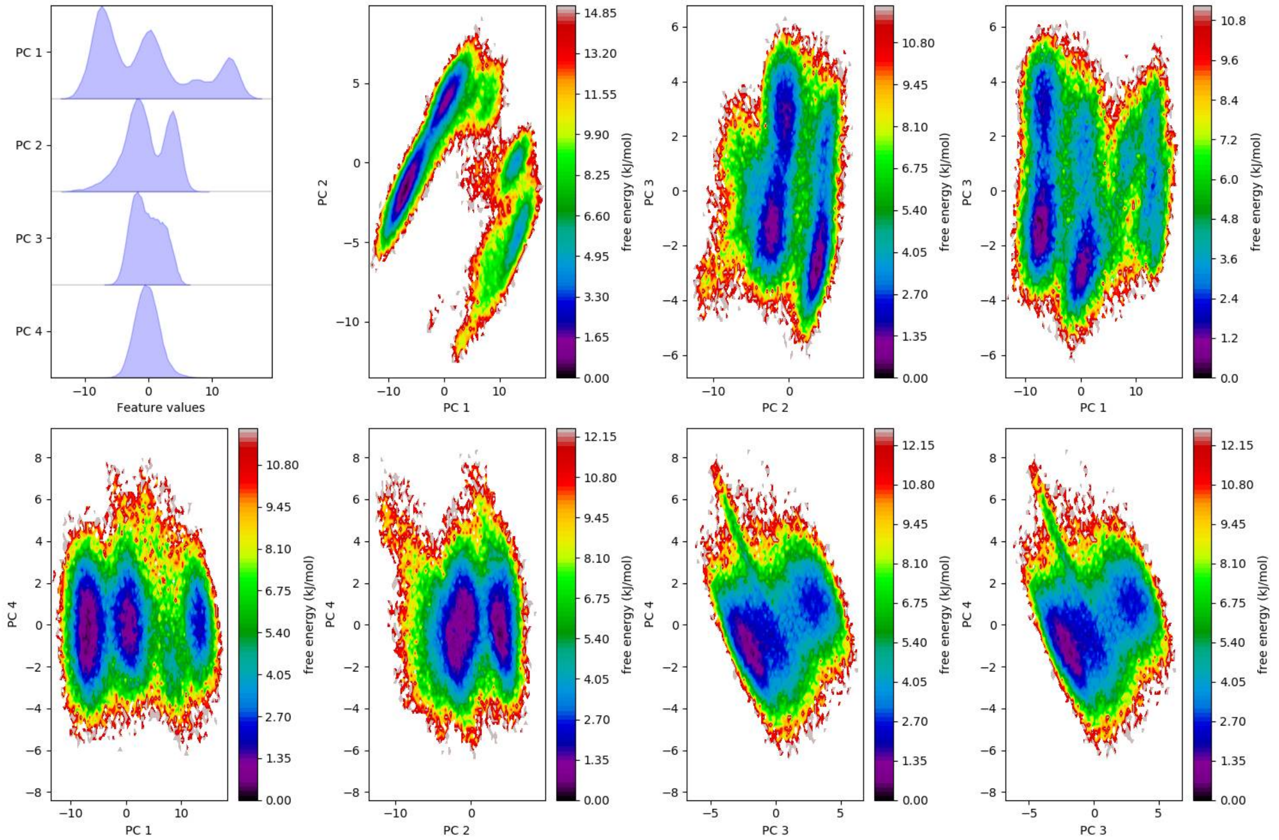

Figure S168 PCA projections between 1-4

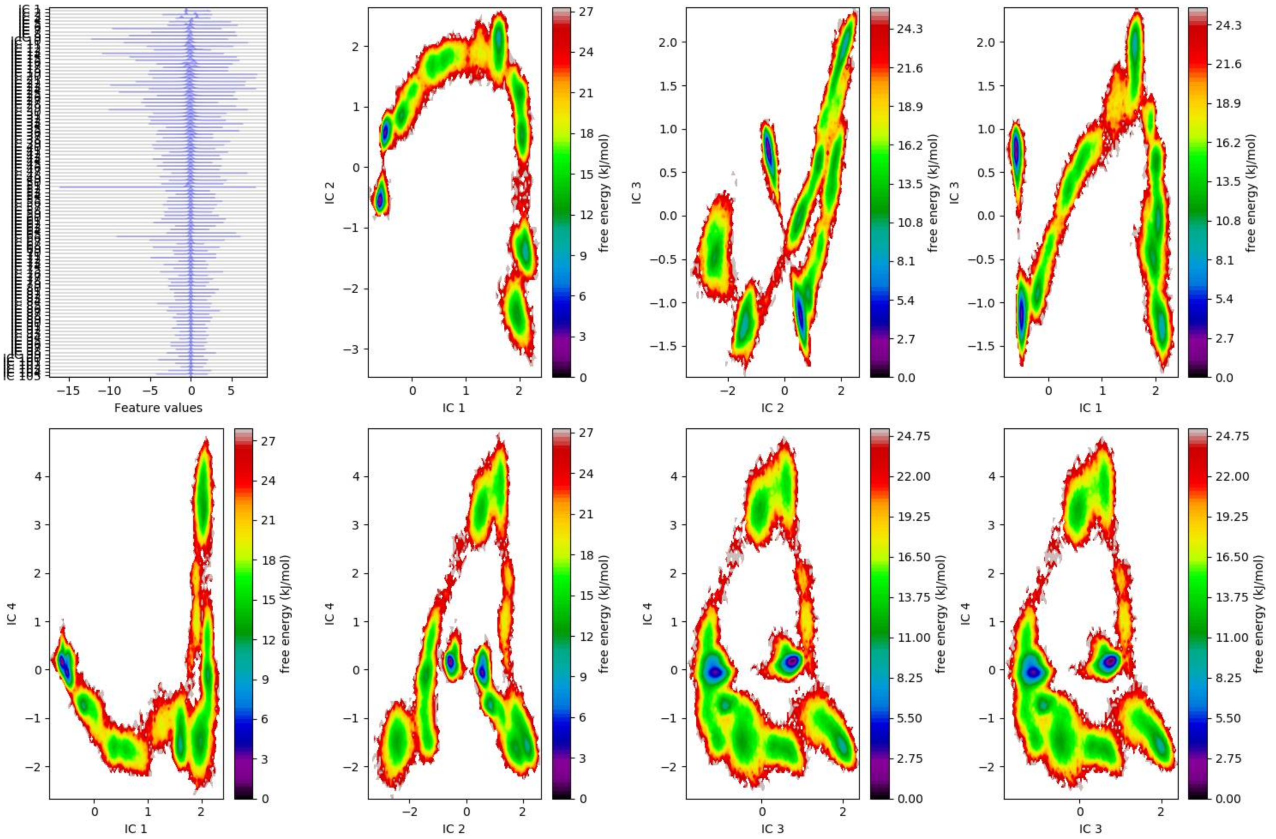

Figure S169 TICA projections between 1-4

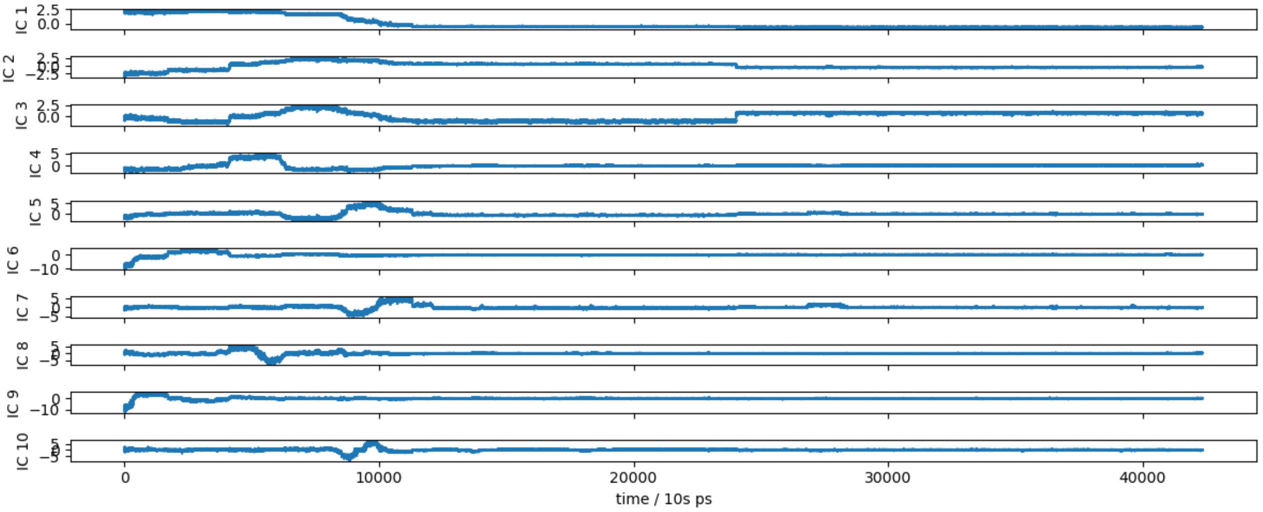

Figure S170 First 10 IC values over the simulation time

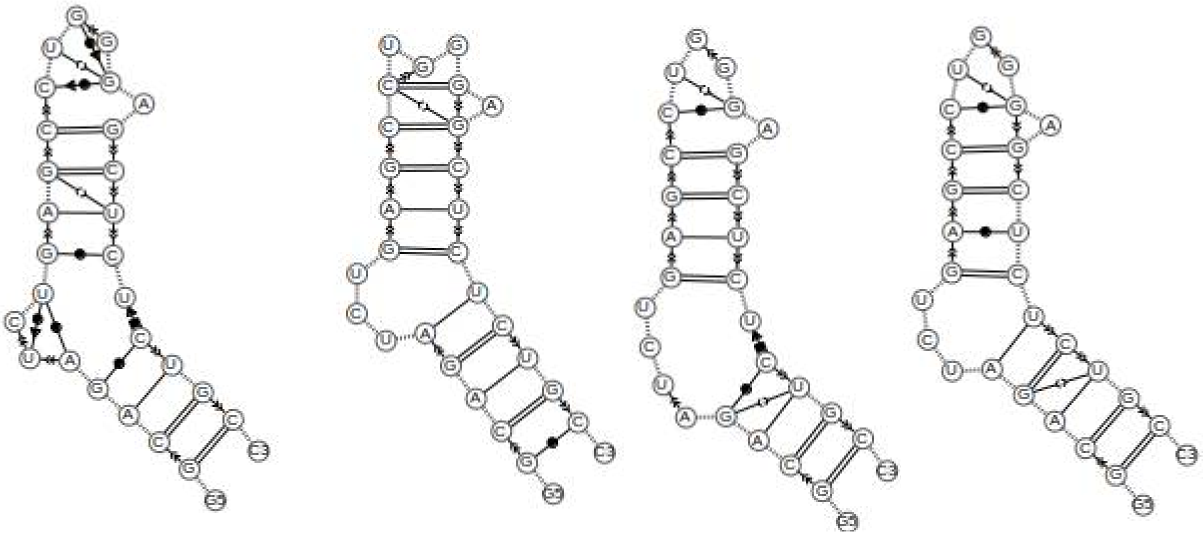

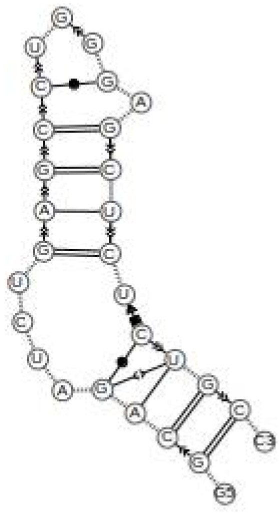

Figure S171 Local minima at the TICA landscape in LW and corresponding structures

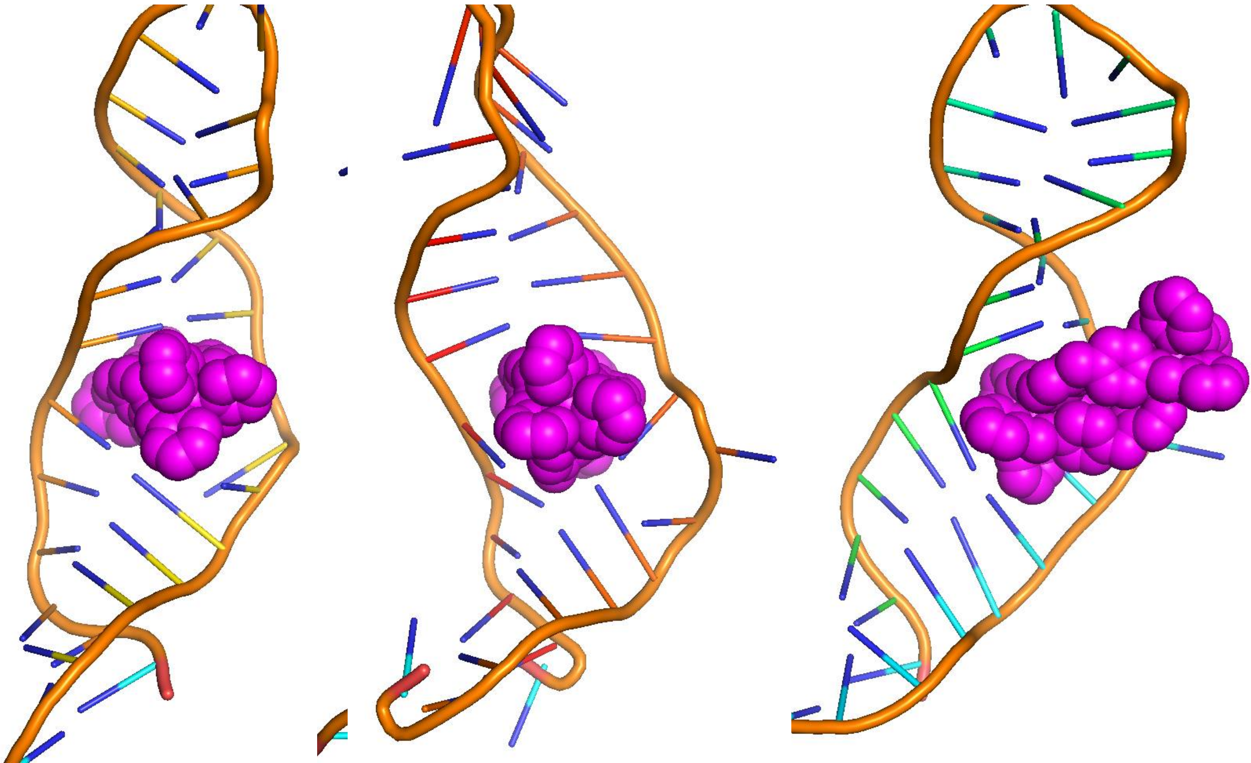

Figure S172 Snapshots of the entering process for the P cylinder.

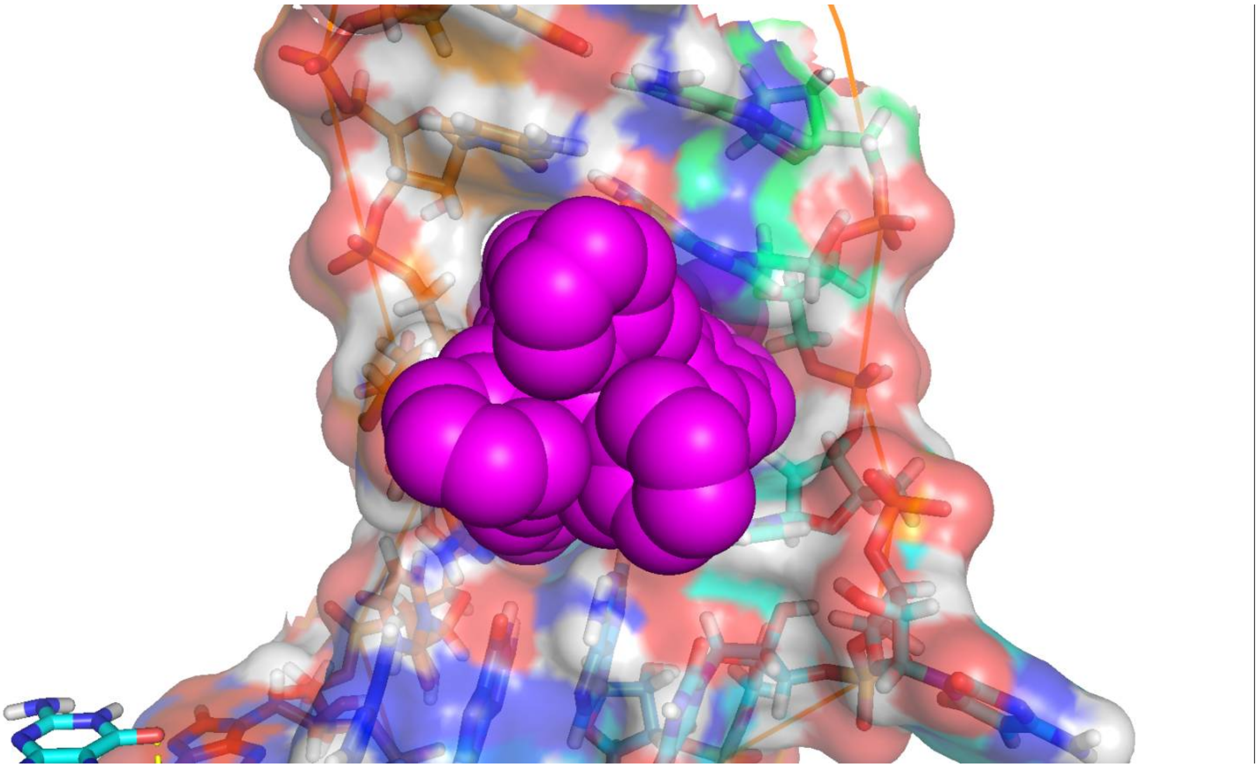

Figure S173 surface of the bulge pocket with the P cylinder bound

References for Supplementary Information

1. High-performance, D. NWChem Computational Chemistry to Science NWChem software.

2. Swart, M., Solà, M. & Bickelhaupt, F. M. A new all-round density functional based on spin states and SN2 barriers. *J. Chem. Phys.* **131**, (2009).

3. Grimme, S., Antony, J., Schwabe, T. & Mück-Lichtenfeld, C. Density functional theory with dispersion corrections for supramolecular structures, aggregates, and complexes of (bio)organic molecules. *Org. Biomol. Chem.* **5**, 741–758 (2007).

4. Staroverov, V. N., Scuseria, G. E., Tao, J. & Perdew, J. P. Comparative assessment of a new nonempirical density functional: Molecules and hydrogen-bonded complexes. *J. Chem. Phys.* **119**, 12129–12137 (2003).

5. Grimme, S., Antony, J., Ehrlich, S. & Krieg, H. A consistent and accurate ab initio parametrization of density functional dispersion correction (DFT-D) for the 94 elements H-Pu. *J. Chem. Phys.* **132**, (2010).

6. Humphrey, W., Dalke, A. & Schulten, K. Sartorius products. *J. Mol. Graph.* **14**, 33–38 (1996).

7. Trott,O., Olson, A. J. Autodock vina: improving the speed and accuracy of docking. *J. Comput. Chem.* **31**, 455–461 (2019).

8. Li, P. & Merz, K. M. MCPB.py: A Python Based Metal Center Parameter Builder. *J. Chem. Inf. Model.* **56**, 599–604 (2016).

9. Minenkov, Y., Singstad, Å., Occhipinti, G. & Jensen, V. R. The accuracy of DFT-optimized geometries of functional transition metal compounds: A validation study of catalysts for olefin metathesis and other reactions in the homogeneous phase. *Dalt. Trans.* **41**, 5526–5541 (2012).

10. Salomon-Ferrer, R., Case, D. A. & Walker, R. C. An overview of the Amber biomolecular simulation package. *Wiley Interdiscip. Rev. Comput. Mol. Sci.* **3**, 198–210 (2013).

11. Abraham, M. J. *et al.* Gromacs: High performance molecular simulations through multi-level parallelism from laptops to supercomputers. *SoftwareX* **1–2**, 19–25 (2015).

12. Aytenfisu, A. H., Spasic, A., Grossfield, A., Stern, H. A. & Mathews, D. H. Revised RNA Dihedral Parameters for the Amber Force Field Improve RNA Molecular Dynamics. *J. Chem. Theory Comput.* **13**, 900–915 (2017).

13. Scherer, M. K. *et al.* PyEMMA 2: A Software Package for Estimation, Validation, and Analysis of Markov Models. *J. Chem. Theory Comput.* **11**, 5525–5542 (2015).

14. Reißer, S. *et al.* Barnaba: software for analysis of nucleic acid structures and trajectories. *Rna* **25**, 219–231 (2018).

15. Kumari, R., Kumar, R., Lynn, A. & Lynn, A. *g_mmpbsa* —A GROMACS Tool for High-Throughput MM-PBSA Calculations. *J. Chem. Inf. Model.* **54**, 1951–1962 (2014).

16. Leontis, N. B. The non-Watson-Crick base pairs and their associated isostericity matrices. *Nucleic Acids Res.* **30**, 3497–3531 (2002).

17. Meistermann, I. *et al.* Intramolecular DNA coiling mediated by metallo-supramolecular cylinders: differential binding of P and M helical enantiomers. *Proc. Natl. Acad. Sci. U. S. A.* **99**, 5069–5074 (2002).

18. Disney, M. D., Dwyer, B. G. & Childs-Disney, J. L. Drugging the RNA world. *Cold Spring Harb. Perspect. Biol.* **10**, (2018).

19. Aytenfisu, A. H., Spasic, A., Grossfield, A., Stern, H. A. & Mathews, D. H. Revised RNA Dihedral Parameters for the Amber Force Field Improve RNA Molecular Dynamics. *J. Chem. Theory Comput.* **13**, 900–915 (2017).

20. Musiani, F. *et al.* Molecular dynamics simulations identify time scale of conformational changes responsible for conformational selection in molecular recognition of HIV-1 transactivation responsive RNA. *J. Am. Chem. Soc.* **136**, 15631–15637 (2014).

21. Phongtongpasuk, S. *et al.* Binding of a designed anti-cancer drug to the central cavity of an RNA three-way junction. *Angew. Chemie - Int. Ed.* **52**, 11513–11516 (2013).

22. Frisch, M. J.; Trucks, G. W.; Schlegel, H. B.; Scuseria, G. E.; Robb, M. A.; Cheeseman, J. R.; Scalmani,G.; Barone, V.; Mennucci, B.; Petersson, G. A.; Nakatsuji, H.; Caricato, M.; Li, X.; Hratchian, H. P.;Izmaylov, A. F.; Bloino, J.; Zheng, G.; Sonnenberg, J. L.; Hada, M.; Ehara, M.; Toyota, K.; Fukuda, R.;Hasegawa, J.; Ishida, M.; Nakajima, T.; Honda, Y.; Kitao, O.; Nakai, H.; Vreven, T.; Montgomery, J.A., J.; Peralta, J. E.; Ogliaro, F.; Bearpark, M.; Heyd, J. J.; Brothers, E.; Kudin, K. N.; Staroverov, V.N.; Kobayashi, R.; Normand, J.; Raghavachari, K.; Rendell, A.; Burant, J. C.; Iyengar, S. S.; Tomasi, J.;Cossi, M.; Rega, N.; Millam, J. M.; Klene, M.; Knox, J. E.; Cross, J. B.; Bakken, V.; Adamo, C.;Jaramillo, J.; Gomperts, R.; Stratmann, R. E.; Yazyev, O.; Austin, A. J.; Cammi, R.; Pomelli, C.;Ochterski, J. W.; Martin, R. L.; Morokuma, K.; Zakrzewski, V. G.; Voth, G. A.; Salvador, P.;Dannenberg, J. J.;Dapprich, S.; Daniels, A. D.; Farkas, Ö.; Foresman, J. B.; Ortiz, J. V.; Cioslowski, J.; Fox, D. J. Gaussian, Inc.: Wallingford CT 2009.
